# Organocatalytic enantio- and diastereoselective assembly of cyclopropane-incorporated polycyclic molecules *via* isobenzopyrylium ions†

**DOI:** 10.1039/d4sc03746d

**Published:** 2024-08-22

**Authors:** Shuxuan Liu, Chaoshen Zhang, Zhengyu Han, Hai Huang, Jianwei Sun

**Affiliations:** a Jiangsu Key Laboratory of Advanced Catalytic Materials & Technology, School of Petrochemical Engineering, Changzhou University Changzhou China; b Department of Chemistry and the Hong Kong Branch of Chinese National Engineering Research Centre for Tissue Restoration & Reconstruction, The Hong Kong University of Science and Technology Clear Water Bay Kowloon Hong Kong SAR China zhangcs@ust.hk sunjw@ust.hk

## Abstract

A highly enantio- and diastereoselective organocatalytic formation of cyclopropanes embedded in a complex bridged polycyclic architecture is disclosed. In the presence of a chiral phosphoric acid catalyst, this reaction generates four new stereogenic centers and three new C–C bonds efficiently from isochromene acetals and vinylboronic acids under mild conditions. Different from conventional asymmetric cyclopropanation strategies, this process does not involve carbenes or carbenoids. The complex products can serve as precursors to useful homoenolate equivalents. Mechanistically, DFT studies provided insights into the key transition states of the enantiodetermining [4 + 2] cycloaddition, in which the enantioselectivity is induced by the chiral phosphate counter anion of the isobenzopyrylium intermediate.

## Abstract 2

An organocatalytic asymmetric cycloaddition of isobenzopyrylium with vinylboronic acid was successfully developed, providing rapid access to cyclopropane-containing complex polycyclic structures with four chiral centers.

## Graphical abstract

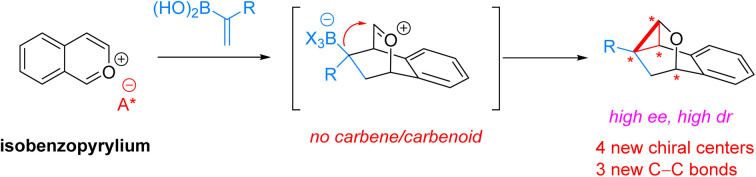

## Introduction

**Fig. 1:**
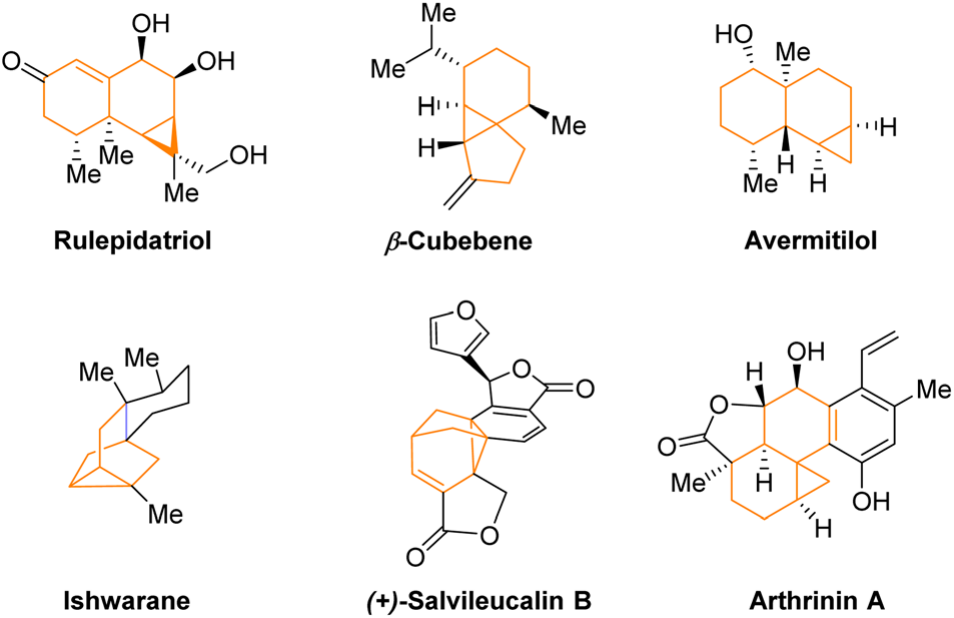
Selected cyclopropane-containing polycyclic natural molecules.

**Scheme 1:**
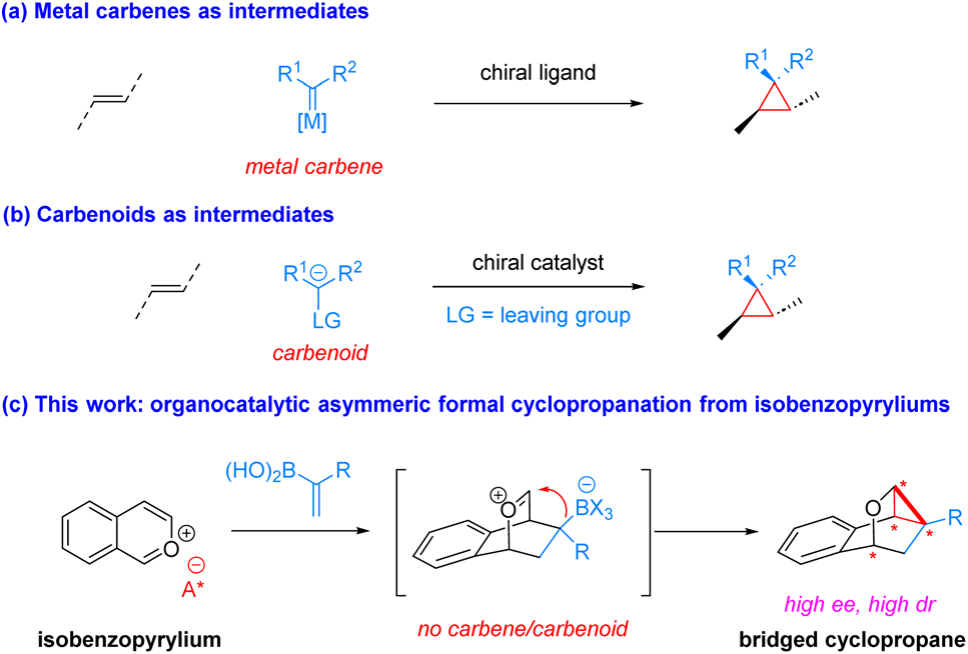
Introduction to catalytic asymmetric cyclopropanation strategies.

Cyclopropane is a ubiquitous structural unit in numerous natural products and drug molecules (Fig. 1).^1,2^ Incorporation of a cyclopropane unit into polycyclic bioactive molecules has been shown to enhance their rigidity, potency, and metabolic stability.^2^ Moreover, cyclopropanes can also serve as versatile building blocks in organic synthesis owing to their higher reactivity than ordinary alkanes driven by inherent ring strain and partial π-bond nature.^3^ Strategic utilization of their reactivity has facilitated expedient construction of many complex molecules.^3*a*–*c*^ Consequently, various methods have been developed for efficient cyclopropanation reactions, particularly in an enantioselective manner, since many of the above-mentioned useful cyclopropane-containing molecules are chiral.^3–7^ Among known strategies, catalytic asymmetric cyclopropanation of olefins by metal carbenes using a chiral ligand has been well established (Scheme 1a).^5^ Carbenoids bearing a nucleophilic carbanion and a leaving group in the geminal position have also been utilized as versatile species to provide efficient access to diverse enantioenriched cyclopropanes in the presence of a chiral catalyst (Scheme 1b).^6^ However, other methods that do not require carbenes or carbenoids for highly enantioselective formation of cyclopropanes remain under developed.^7^ Moreover, there has been limited demonstration of asymmetric cyclopropanations for concomitant assembly of strained bridged polycycles. In this context, here we report such a highly enantioselective organocatalytic example without involving carbenes or carbenoids.

Isobenzopyrylium ions are versatile species in organic synthesis.^8^ Their propensity toward cycloaddition with alkenes and alkynes as well as nucleophilic addition has permitted rapid access to diverse useful molecules, including (dihydro)naphthalenes and strained polycyclic structures.^8–11^ However, the lack of obvious ligation sites for metal coordination in such planar aromatic structures rendered it difficult for them to be utilized in catalytic asymmetric synthesis,^9^ unless they were generated *in situ* with a metal already attached.^10^ In 2015, we demonstrated the chiral counteranion strategy for successful asymmetric induction on isobenzopyryliums when reacting with 1,2-disubstituted vinylboronic acids to deliver enantioenriched dihydronaphthalenes.^9*a*^ In contrast, we have recently discovered that slight variation of the vinylboronic acids to 1,1-disubstituted ones led to completely different product topology.^11^ As shown in Scheme 1c, a type of highly strained cyclopropane-incorporated polycyclic structures, likely *via* [4 + 2] cycloaddition followed by intramolecular nucleophilic attack onto the oxonium by the well-positioned nucleophilic C–B motif. While this intriguing process has been developed with high chemical efficiency, unfortunately, a highly enantioselective variant has not been achieved due to the generally challenging stereocontrol on isobenzopyrylium ions.

## Results and discussion

We commenced our study with isochromene acetal 1a as the isobenzopyrylium precursor and the freshly prepared (1-phenylvinyl)boronic acid 2a as the model reaction partner (Table 1). Based on our previous success of chiral phosphoric acid (CPA) catalysis with isochromene acetals, we initially evaluated different CPAs as catalyst. Indeed, common CPAs A1–A4 were all able to catalyze the desired reaction at 0 °C in DCM in the presence of 3 Å molecular sieves, forming the cyclopropane-containing bridged product 3a in moderate to good yield (entries 1–4).^12^ While the product was formed as a single diastereomer, the enantioselectivity was moderate. Of note, although A3 proved promising for further improvement of enantioselectivity, unfortunately, considerable efforts devoted to its structural modification and other reaction condition examinations proved fruitless. Next, we screened a range of other CPA structures. To our delight, after exhaustive screenings we found that adding substituents to the anthracene ring of A4 led to improvement in enantioselectivity (entries 5–8). CPAs A5–A8 all gave better enantiocontrol, with the bulkiest substituent (A8) providing the best enantioselectivity (entry 8).

**Table 1:** Evaluation of catalysts^a^

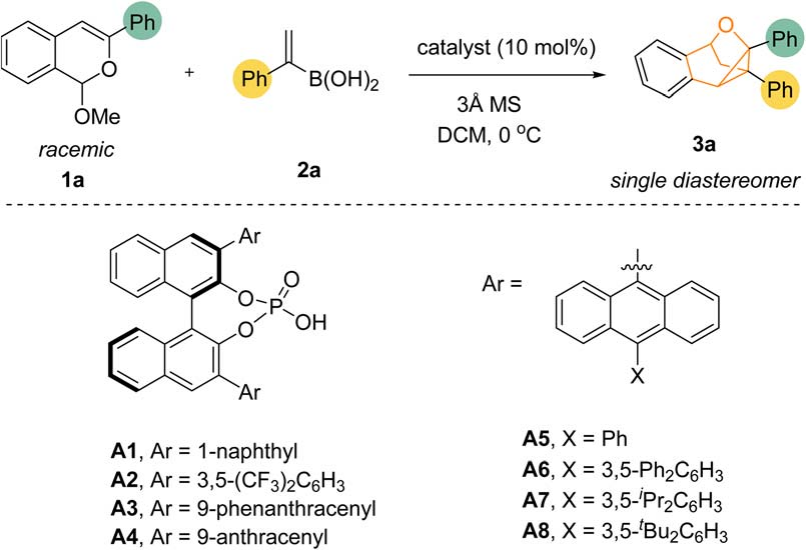			
Entry	CPA	Yield^b^ (%)	ee^b^ (%)
1	A1	60	33
2	A2	84	46
3	A3	55	69
4	A4	65	43
5	A5	60	76
6	A6	53	71
7	A7	44	83
8	A8	68	86

a Reaction scale: 1a (50 μmol), 2a (125 μmol), catalyst (5.0 μmol), solvent (0.5 mL), 0 °C, 24 h.

b Yield was determined by analysis of the ^1^H NMR spectrum of the crude reaction mixture with CH_2_Br_2_ as the internal standard. ee was determined by HPLC analysis on a chiral stationary phase.

Aiming to further enhance the reaction enantioselectivity, we resorted to other tactics (Table 2). According to our previous study,^9*a*^ the leaving group in acetal 1 may play a role in the enantiodetermining step and thus can influence enantioselectivity. Therefore, substrates with different leaving groups (**1b–1f**) were examined (Table 2, entries 1–5). Interestingly, the use of ^*i*^PrO, ^*t*^BuO, and BnO as leaving group all led to improvement on enantioselectivity. Among them, substrate 1b bearing the ^*i*^PrO group provided the best enantioselectivity (92% ee, entry 1). With this substrate, we further evaluated other reaction parameters. Unfortunately, other solvents, such as PhCl, THF, and Et_2_O, did not lead to improvement (entries 6–8). Indeed, significant decrease in both yield and enantioselectivity was observed. Similarly, dramatic decrease in yield was also observed when the reaction as run in the absence of molecular sieves (entry 9). It was found that unidentifiable byproducts were formed in these cases. Nevertheless, increasing the concentration led to slight increase in yield without compromising enantioselectivity (entry 10). Finally, decreasing the temperature to −50 °C further improved this reaction, which was equally efficient with 5 mol% of catalyst, giving both excellent yield and enantioselectivity (78% yield and 97% ee, entry 11). Notably, four new stereogenic centers and three new C–C bonds were formed in this reaction.

**Table 2:** Further optimization^a^

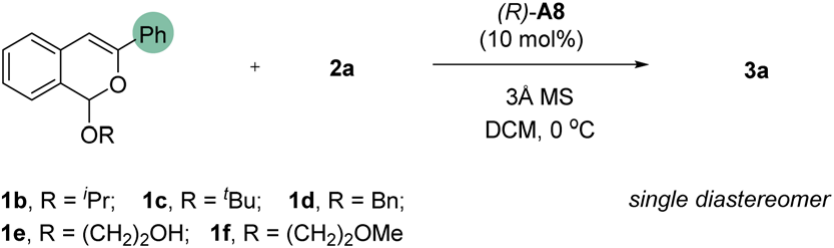				
Entry	1	Solvent	Yield^b^ (%)	ee^b^ (%)
1	1b	DCM	70	92
2	1c	DCM	68	91
3	1d	DCM	68	90
4	1e	DCM	36	78
5	1f	DCM	76	86
6	1b	PhCl	16	73
7	1b	THF	12	12
8	1b	Et_2_O	4	8
9^c^	1b	DCM	40	80
10^d^	1b	DCM	74	92
11^d^^,^^e^	1b	DCM	78	97

a Reaction scale: 1 (50 μmol), 2a (125 μmol), catalyst (5.0 μmol), solvent (0.5 mL), 0 °C, 24 h.

b Yield was determined by analysis of the ^1^H NMR spectrum of the crude reaction mixture with CH_2_Br_2_ as the internal standard. ee was determined by HPLC analysis on a chiral stationary phase.

c without 3 Å MS.

d *c* = 0.2 M.

e Run with 5 mol% of catalyst at −50 °C for 72 h.

**Scheme 2:**
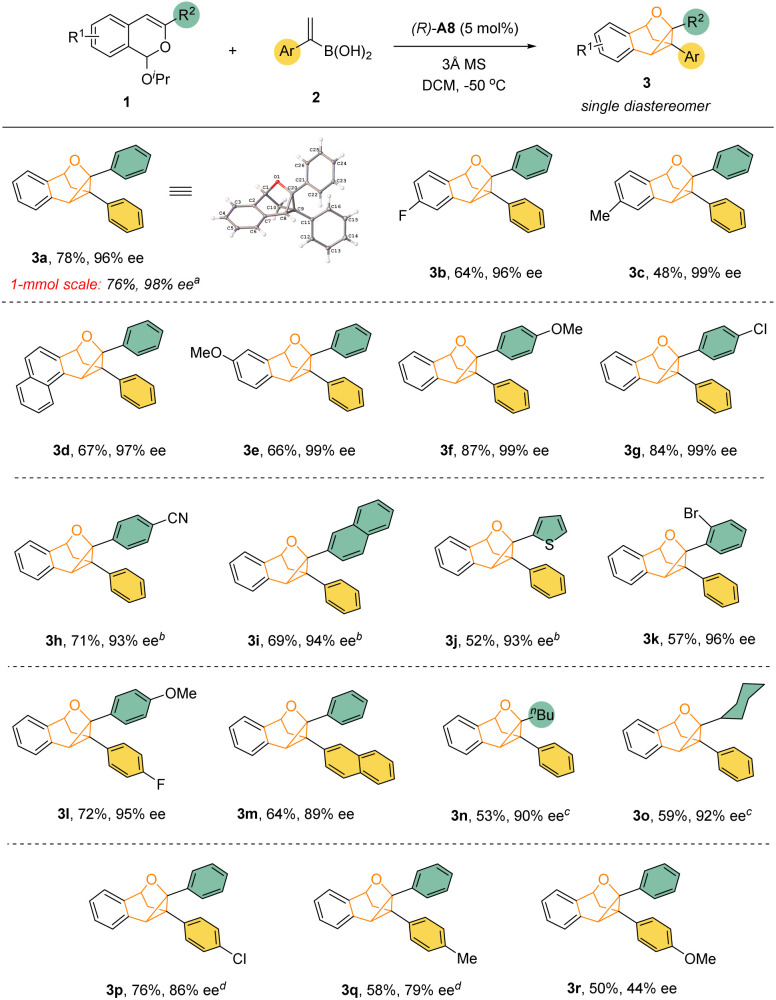
Reaction scope. Reaction conditions: 1 (0.30 mmol), 2a (0.75 mmol), (*R*)-A8 (0.015 mmol), 3 Å MS (30 mg), solvent (1.5 mL), −50 °C, 96 h. Isolated yield is provided. The ee value was determined by chiral HPLC analysis. ^*a*^ 168 h. ^*b*^ 10 mol% of catalyst at −35 °C. ^*c*^ 10 mol% of catalyst at room temperature for 48 h. ^*d*^ Room temperature.

With the optimized conditions, we explored the reaction substrate generality (Scheme 2). With isopropoxy group as the leaving group, a range of differently substituted isochromene acetals reacted smoothly with vinylboronic acids 2 to afford the desired cyclopropane-containing polycyclic products 3 with excellent enantioselectivity and diastereoselectivity (Scheme 2). Both electron-donating and electron-withdrawing groups on the isobenzopyrylium ions did not affect the desired reactivity and enantioselectivity. Substrates with an aliphatic substituent at the 3-position (3n and 3o) showed relatively low reactivity, thus requiring slightly higher temperature. Nevertheless, the desired products could still be obtained with high enantioselectivity. The influence of the substituents on the vinylboronic acids were also explored. Minor change of electronic properties of the aromatic ring still led to successful formation of the desired product. However, aliphatic substitution on the vinyl group resulted in no desired product. Finally, the standard protocol was also demonstrated to be equally efficient in a 1 mmol scale reaction with 1a. The product structure and absolute stereochemistry of 3a were also confirmed by X-ray crystallography.

**Scheme 3:**
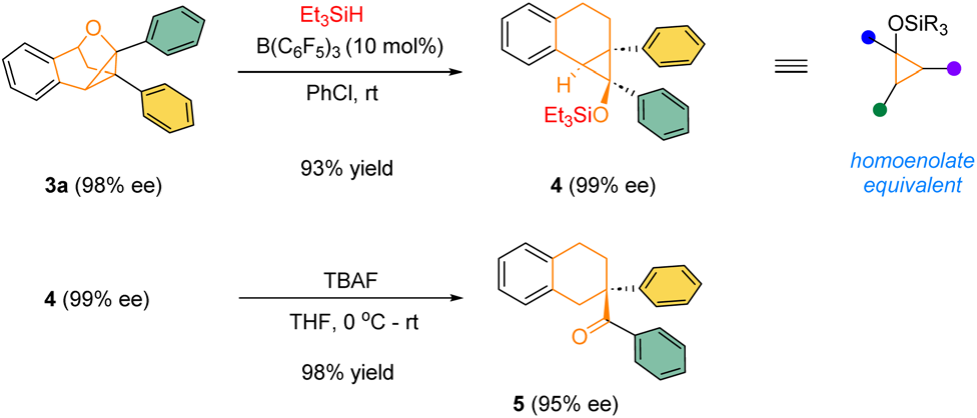
Product transformations.

The highly enantioenriched bridged polycyclic products are not only architecturally intriguing, but also synthetically useful. For example, in the presence of a catalytic amount of B(C_6_F_5_)_3_, the product 3a could react with Et_3_SiH to form densely-substituted siloxy cyclopropane 4, a type of synthetically versatile homoenolate equivalents.^13^ In the presence of TBAF, the silyl ether 4 underwent smooth desilylation followed by spontaneous ring-opening to form the corresponding homoenolate, which was immediately protonated by adventitious water to form the corresponding ketone 5 bearing an all-carbon quaternary stereogenic center in excellent yield and enantiomeric excess (Scheme 3).

To gain some insights of the reaction mechanism, we carried out a control experiment with vinyl boronic ester 2a′. Under the standard conditions, the reaction between 1b and 2a′ did not proceed to form the product 3a (eqn (1)), indicating that the boronic acid functionality is crucial for the desired reactivity.1
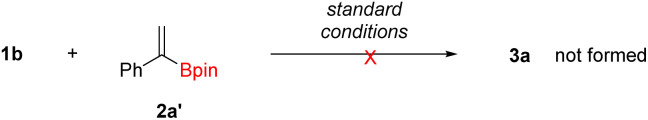


**Scheme 4:**
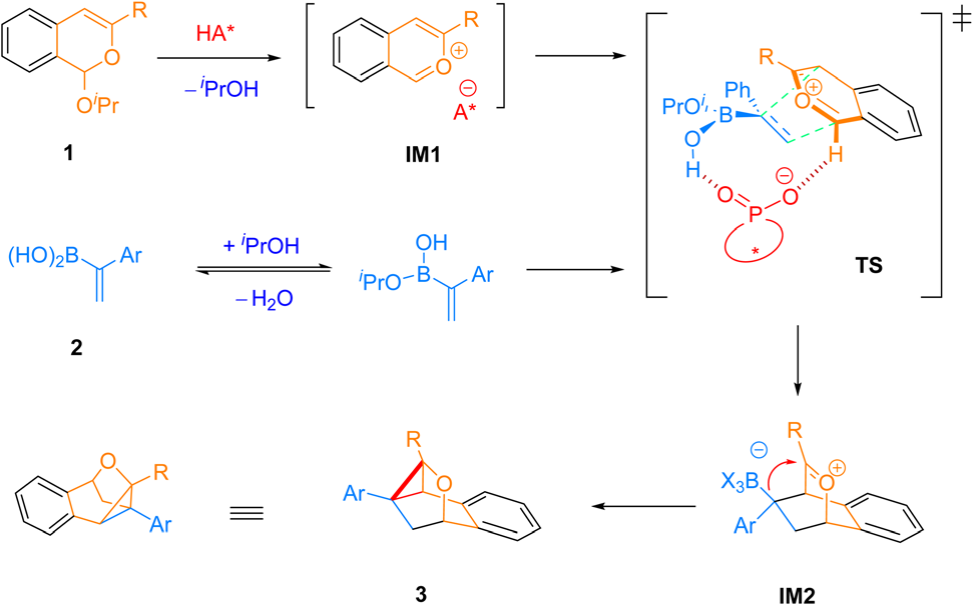
Proposed mechanism.

A possible reaction pathway is depicted in Scheme 4. The reaction begins with acid activation of substrate 1 to generate the key intermediate isobenzopyrylium IM1, which is a chiral ion pair with chiral phosphate as the counteranion. This step generates a molecule of isopropanol, which can potentially exchange with water in vinylboronic acid 2, perhaps assisted by molecular sieves. Next, the chiral phosphate may interact with the vinylboronic acid by forming a borate complex, which not only enhances the nucleophilicity of the double bond, but also relays chirality to the whole vinyl borate nucleophile. The latter is critical for facial discrimination when approaching the isobenzopyrylium. The subsequent stereocontrolled [4 + 2] cycloaddition generates intermediate IM2, with essential stereogenic centers established in this step. While the detailed coordination environment on boron is unknown, it is hypothesized that the isopropoxy group is likely involved in this key transition state TS since the leaving group in substrate 1 showed obvious influence on enantioselectivity. Moreover, the regioselectivity of this step is probably controlled by the electronic properties of the reaction partners, with the electron-rich terminal position of the styrene motif attacking the electron-deficient oxonium carbon. In IM2, the borate unit is nucleophilic and well-positioned for nucleophilic attack onto the oxonium. Thus, facile C–C bond formation takes place to close the cyclopropane ring and give product 3. In the whole process, no carbene or carbenoid species is involved, thus distinct from conventional cyclopropanation strategies.

**Fig. 2:**
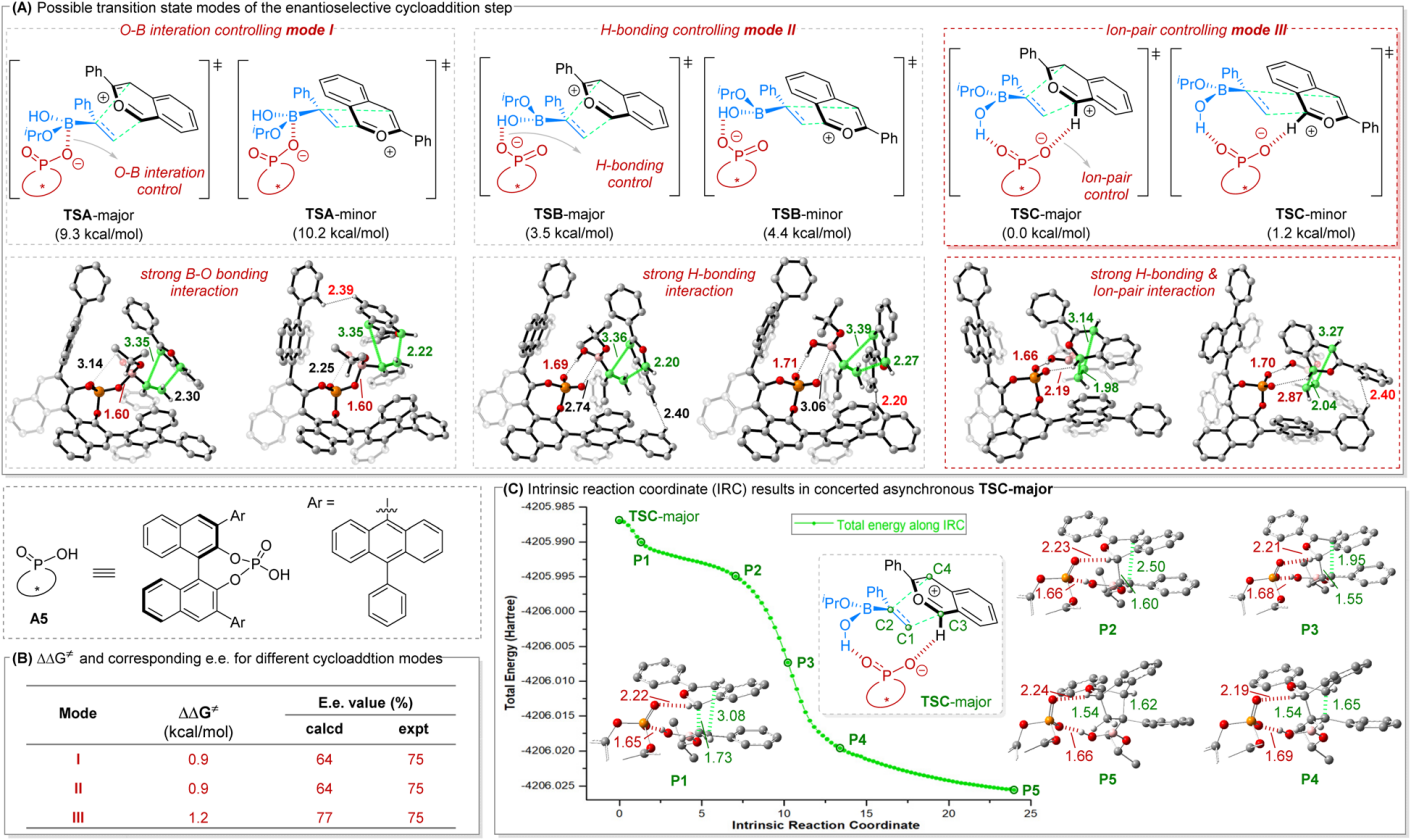
DFT calculation results on the enantioselective cycloaddition step. All transition state energies are relative to TSC-major. ΔΔ*G*^‡^ values are relative activation energies (TS-minor − TS-major). The key bond lengths in the enantioselective transition states are given in angstroms.

To further disclose how the chiral phosphoric acid (CPA) catalyst dictates the stereochemical course of the enantiodetermining [4 + 2] cycloaddition step, density functional theory (DFT) calculations were performed on the model reaction between 1b and 2a using CPA A5 as catalyst (Fig. 2). The calculated enantiodetermining transition states were optimized and characterized in dichloromethane with the SMD solvent model (SCRF = SMD) at M06-2X/6-31G(d) level, and single point energies were further calculated at M06-2X/6-311+G(d,p)//M06-2X/6-31G(d) level with solvent effects accounted by the SMD solvent model, using the experimental solvent (dichloromethane). Many possible interactions might be present in this system, including ion-pairing between the CPA anion and isobenzopyrylium cation as well as those other interactions that the vinylboronic acid can participate in (*e.g.* O⋯B interaction; hydrogen bonding; ion pairing). This may result in multiple possible transition states for the enantiodetermining [4 + 2] cycloaddition step. Here we focused on three feasible cycloaddition modes, namely sandwich-type modes I and II with the anion and cation motifs relatively separated, and the ion-pair mode III with the anion and cation motifs closer to each other (Fig. 2A).

Considering the possible formation of an anionic tetrahedral tetracoordinate boron moiety between the tricoordinate boron in 2a and the oxygen atom of the CPA anion, we first examined mode I that involves strong O⋯B interaction, reflected by the short O⋯B distance of 1.60 Å. While the energy gap (0.9 kcal mol^−1^) in this mode I (TSA-minor relative to TSA-major) could explain the experimental enantioselectivity (Fig. 2B), transition states in mode II were found to be 5.8 kcal mol^−1^ more favorable that those in mode I. Indeed, stronger hydrogen bonding interaction was found in mode II (hydrogen bond distance of 1.69 Å in TSB-major *versus* 3.14 Å in TSA-major).

More importantly, it was further found that the transition states in mode III show both strong H-bonding interaction and ionic interaction, leading to better organized transition states and further decreasing the cycloaddition barrier. The calculated energy gap is also most consistent with the observed enantioselectivity (Fig. 2B). Further structural analyses in the above three cycloaddition modes indicate that TS-minors are unfavorable mainly because they suffer from larger steric repulsions between the CPA catalyst and the substrates, as mirrored in the sum of H⋯H van der Waals radius distance of 2.39 Å in TSA-minor, 2.20 Å in TSB-minor and 2.40 Å in TSC-minor, respectively. It is worth noting that intrinsic reaction coordinate (IRC) calculations for TSC-major in Fig. 2C indicate that the two carbon–carbon bonds are not formed synchronously. Indeed, the C1–C3 bond is formed first but unstable at conformation P2, followed by C2–C4 bond formation to afford the stable cycloaddition product (P5). Therefore, this [4 + 2] cycloaddition step is concerted asynchronous in nature.

## Conclusions

In summary, we have developed a highly enantio- and diastereoselective organocatalytic strategy for the formation of cyclopropanes. This is a rare demonstration of asymmetric cyclopropanation with concomitant formation of a complex bridged polycyclic structure, with four new stereogenic centers and three new C–C bonds formed under mild conditions. Different from conventional strategies, this process does not involve carbenes or carbenoids as intermediates. Instead, the proper choice of a suitable chiral phosphoric acid together with other parameters, including the use of molecular sieves and a suitable leaving group, proved essential to the success. The complex products generated in this process can also serve as precursors to enantioenriched siloxy cyclopropanes, useful homoenolate equivalents. Mechanistically, this is another demonstration of the rarely developed asymmetric processes of isobenzopyrylium ions, a family of versatile synthetic intermediates but challenging for asymmetric control due to the lack of coordination sites for metal catalysis. In this process, the chiral counteranion, likely formed by association of the chiral phosphate and the vinylboronic acid, provides outstanding facial differentiation during bond formation. It is expected that this mechanistic scenario can be extended to other asymmetric processes involving isobenzopyrylium ions.

## Author contributions

J. S. conceived and designed the experiments. S. L. performed experiments and analyzed data. C. Z. performed DFT calculations. J. S., C. Z. and S. L. wrote the manuscript. Z. H. and H. H. assisted the experiments and commented on the manuscript.

## Conflicts of interest

There are no conflicts to declare.

## Supplementary Material

SC-015-D4SC03746D-s001

SC-015-D4SC03746D-s002

## Data Availability

All data, including experimental details, characterization data, NMR spectra and HLPC traces are available in ESI.†
