# Supplementary material for: Organocatalytic enantio- and diastereoselective assembly of cyclopropane-incorporated polycyclic molecules via isobenzopyrylium ions
Source: Chem Sci. 2024 Aug 22;15(37):15274–9. doi: 10.1039/d4sc03746d (PMC11378009; doi:10.1039/d4sc03746d)
Supplement: SC-015-D4SC03746D-s001 [file SC-015-D4SC03746D-s001.pdf]

Supplementary Information (SI) for Chemical Science.
This journal is © The Royal Society of Chemistry 2024

Supporting Information

Organocatalytic Enantio- and Diastereoselective Assembly of Cyclopropane-Incorporated Polycyclic Molecules via Isobenzopyrylium Ions

Shuxuan Liu,[ab] Chaoshen Zhang,*[b] Zhengyu Han,[a]
Hai Huang[a] and Jianwei Sun*[b]

[a]*Jiangsu Key Laboratory of Advanced Catalytic Materials & Technology, School of Petrochemical Engineering, Changzhou University, Changzhou (China)*

[b]*Department of Chemistry and the Hong Kong Branch of Chinese National Engineering Research Centre for Tissue Restoration & Reconstruction, The Hong Kong University of Science and Technology, Clear Water Bay, Kowloon, Hong Kong SAR (China)*

Table of Contents

I. General Information S-2
II. Preparation of Substrates S-4
IV. CPA-Catalyzed Synthesis of Bridged Cyclopropanes S-13
IV Large-Scale Reaction S-27
V. Product Derivatizations S-28
VI. Product Structure Determination S-30
VII. Computational Details S-39
NMR Spectra and HPLC Traces

I. General Information

Flash column chromatography was performed over silica gel (200-300 or 300-400 mesh) purchased from Qindao Haiyang Co., China or SiliCycle® Inc., Canada. All air or moisture sensitive reactions were conducted in oven-dried glassware under nitrogen atmosphere using anhydrous solvents. TLC was visualized by UV fluorescence (254 nm) then phosphomolybdic acid. Tetrahydrofuran was distilled from sodium/benzophenone. Anhydrous *N,N*-dimethyl formamide, dichloromethane, methanol, toluene, diethyl ether, acetonitrile, and *n*-hexane were purified by the Innovative® solvent purification system. Other anhydrous solvents were purchased from Sigma-Aldrich®, J&K® and Energy® and used as received. Chemicals were purchased from commercial suppliers, such as Sigma-Aldrich®, J&K®, Energy® and used without further purification unless otherwise stated. All the molecular sieves were purchased from Strem® Chemicals, and vacuum dry at 200 ^{o}C for 30 minutes. ^{1}H, ^{13}C, ^{31}P and ^{19}F NMR spectra were collected on a Bruker AV 400 and 300 MHz NMR spectrometers using residue solvent peaks as an internal standard (^{1}H NMR: $CDCl_3$ at 7.26 ppm; ^{13}C NMR: $CDCl_3$ at 77.0 ppm) Data for ^{1}H NMR were recorded as follows: chemical shift (δ, ppm), multiplicity (s = singlet; d = doublet; t = triplet; q = quarter; p = pentet; sept = septet; m = multiplet; br = broad), coupling constant (Hz), integration. Mass spectra were collected on an Agilent GC/MS 5975C system, or a MALDI Micro MX mass spectrometer, or an API QSTAR XL System. Optical rotations were measured on Rudolph Research Analytical Autopol I automatic polarimeter with $[\alpha]_D$ values reported in degrees; concentration (c) is in 10 mg/mL. The enantiomeric excess values were determined by chiral HPLC using an Agilent 1200 LC instrument or Agilent 1260 LC instrument with Daicel CHIRALPAK® AD-H, IC, IC-3 or CHIRALCEL® OD-H, OD-3 columns. Unless otherwise noted, the racemic

samples in this study were prepared using the racemic phosphoric acid catalyst 1,1'-binaphthyl-2,2'-diyl hydrogenphosphate.

II. Preparation of Substrates

The isochromene substrates **1a, 1b, 1c, 1d, 1e, 1f, 1j, 1p** are known compounds.[1] The procedure for the synthesis of other substrates is shown below. All vinylboronic acids used here are known compounds.[2]

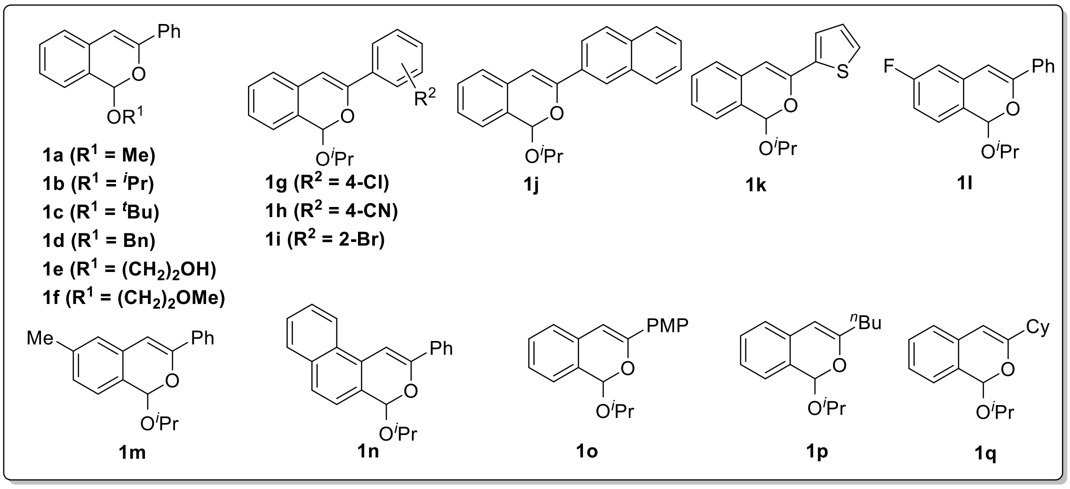

---

1. (a) H. Qian, W. Zhao, Z. Wang and J. Sun, *J. Am. Chem. Soc.,* 2015, **137**, 560–563. (b) Y. Liu and K. Jørgensen, *J. Am. Chem. Soc.,* 2021**, 143**, 8208–8220. (c) M. Dell'Acqua, B. Castano, C. Cecchini, T. Pedrazzini, V. Pirovano, E. Rossi, A. Caselli and G. Abbiati, *J. Org. Chem.,* 2014, **79**, 3494–3505.
2. Y. Zhang, H. Liu, L. Tang and C. Feng, *J. Am. Chem. Soc.,* 2018, **140**, 10695–10699.

General Procedure A.

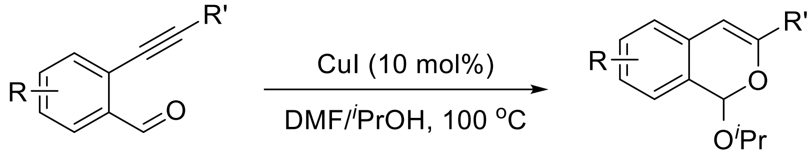

S1 **1**

To an oven-dried 4-mL vial was added the aldehyde **S1** (1.0 mmol), DMF (0.5 mL), isopropanol (0.5 mL), and CuI (19.0 mg, 10 mol%). The reaction mixture was stirred at 100 °C, and the reaction progress was monitored by thin layer chromatography. Upon completion (~72 h), water (3 mL) was added. The mixture was extracted with ethyl acetate (3 × 5 mL). The combined organic layers were washed with water (10 mL) and then brine (10 mL), dried with $NaSO_4$, filtered, and concentrated. The residue was purified by silica gel (pre-treated with Et_3N) flash chromatography to afford the acetal (eluent for all the cases: *n*-hexane/EtOAc = 100:1).

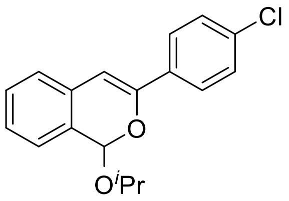

1g

3-(4-Chlorophenyl)-1-isopropoxy-1*H*-isochromene (1g) was prepared according to the General Procedure A as a yellow solid (chromatography eluent: *n*-hexane/EtOAc = 50:1) in 49% yield (0.7 g).

^{1}H NMR (400 MHz, $CDCl_3$) δ 7.72 (d, *J* = 8.4 Hz, 1H), 7.38 – 7.32 (m, 3H), 7.28 – 7.19 (m, 3H), 6.58 (s, 1H), 6.29 (s, 1H), 4.34 (hept, *J* = 6.0 Hz, 1H), 1.31 (d, *J* = 6.0 Hz, 3H), 1.16 (d, *J* = 6.0 Hz, 3H).

^{13}C NMR (101 MHz, $CDCl_3$) δ 148.3, 134.3, 133.2, 130.0, 129.2, 128.6, 127.5, 126.9, 126.0, 125.5, 124.6, 100.7, 96.9, 70.0, 23.5, 21.9.

HRMS (CI+) Calcd for $C_{18}H_{17}ClO_2$ [M]: 300.0917, Found: 300.0918.

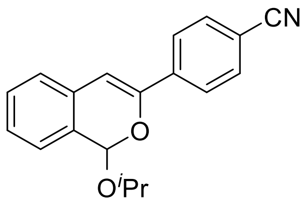

1h

4-(1-Isopropoxy-1*H*-isochromen-3-yl)benzonitrile (1h) was prepared according to the General Procedure A as a yellow solid (chromatography eluent: *n*-hexane/EtOAc = 50:1) in 62% yield (0.9 g).

^{1}H NMR (400 MHz, $CDCl_3$) δ 7.86 (d, *J* = 8.4 Hz, 2H), 7.66 (d, *J* = 8.4 Hz, 2H), 7.38 – 7.28 (m, 2H), 7.25 – 7.21 (m, 2H), 6.71 (s, 1H), 6.30 (s, 1H), 4.32 (hept, *J* = 6.0 Hz, 1H), 1.30 (d, *J* = 6.0 Hz, 3H), 1.14 (d, *J* = 6.0 Hz, 3H).

^{13}C NMR (101 MHz, $CDCl_3$) δ 147.3, 138.9, 132.2, 129.4, 129.3, 127.8, 127.6, 125.6, 125.0, 124.9, 118.8, 111.5, 103.3, 97.0, 70.2, 23.5, 21.9.

HRMS (CI+) Calcd for $C_{19}H_{17}NO_2^+$ $[M]^+$: 291.1254, Found: 291.1251.

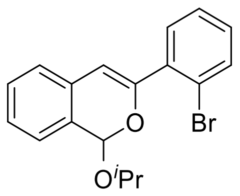

1i

3-(2-Bromophenyl)-1-isopropoxy-1*H*-isochromene (1i) was prepared according to the General Procedure A as a yellow solid (chromatography eluent: *n*-hexane/EtOAc = 50:1) in 67% yield (2.3 g).

^{1}H NMR (400 MHz, $CDCl_3$) δ 7.60 (dd, J_1 = 8.0 Hz, J_2 = 0.8 Hz, 1H), 7.51 (dd, J_1 = 7.6 Hz, J_2 = 1.6 Hz, 1H), 7.32 – 7.20 (m, 4H), 7.17 – 7.13 (m, 2H), 6.33 (s, 1H), 6.28 (s, 1H), 4.30 (hept, *J* = 6.0 Hz, 1H), 1.27 (d, *J* = 6.0 Hz, 3H), 1.21 (d, *J* = 6.0 Hz, 3H).

^{13}C NMR (101 MHz, $CDCl_3$) δ 149.5, 136.6, 133.4, 130.5, 129.8, 129.7, 129.0, 127.2, 127.1, 126.9, 125.4, 124.4, 121.8, 105.5, 97.1, 69.8, 23.4, 21.7.

HRMS (CI+) Calcd for $C_{18}H_{17}BrO_2$ [M]: 344.0412, Found: 344.0414.

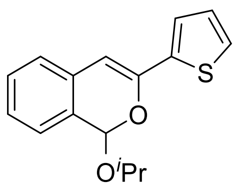

1k

1-Isopropoxy-3-(thiophen-2-yl)-1*H*-isochromene (1k) was prepared according to the General Procedure A as a brown solid (chromatography eluent: *n*-hexane/EtOAc = 50:1) in 40% yield (1.1 g).

^{1}H NMR (300 MHz, $CDCl_3$) δ 7.40 (dd, J_1 = 3.6 Hz, J_2 = 1.2 Hz, 1H), 7.34 – 7.15 (m, 5H), 7.06 – 7.03 (m, 1H), 6.46 (s, 1H), 6.24 (s, 1H), 4.36 (hept, *J* = 6.3 Hz, 1H), 1.30 (d, *J* = 6.0 Hz, 3H), 1.21 (d, *J* = 6.3 Hz, 3H).

^{13}C NMR (101 MHz, $CDCl_3$) δ 145.3, 139.2, 130.0, 129.1, 127.6, 127.5, 126.4, 125.53, 125.48, 124.3, 123.9, 99.6, 97.1, 70.0, 23.6, 21.9.

HRMS (CI+) Calcd for $C_{16}H_{16}O_2S$ [M]: 272.0871, Found: 272.0872.

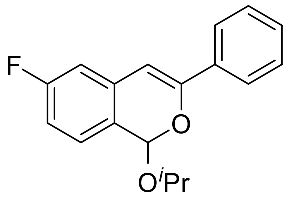

1l

6-Fluoro-1-isopropoxy-3-phenyl-1*H*-isochromene (1l) was prepared according to the General Procedure A as a yellow solid (chromatography eluent: *n*-hexane/EtOAc = 50:1) in 65% yield (0.9 g).

^{1}H NMR (400 MHz, $CDCl_3$) δ 7.78 (d, *J* = 7.6 Hz, 2H), 7.43 – 7.34 (m, 3H), 7.21 – 7.18 (m, 1H), 6.95 – 6.87 (m, 2H), 6.54 (s, 1H), 6.29 (s, 1H), 4.36 (hept, *J* = 6.4 Hz, 1H), 1.31 (d, *J* = 6.0 Hz, 3H), 1.17 (d, *J* = 6.4 Hz, 3H).

^{13}C **NMR** (101 MHz, $CDCl_3$) δ 163.2 (d, *J* = 244.3 Hz), 150.4, 134.3, 132.6 (d, *J* = 9.2 Hz), 129.0, 128.4, 127.4 (d, *J* = 9.2 Hz), 125.0, 123.5 (d, *J* = 2.6 Hz), 113.4 (d, *J* = 22.4 Hz), 110.7 (d, *J* = 22.3 Hz), 99.7 (d, *J* = 2.5 Hz), 96.5, 69.9, 23.5, 21.9.

^{19}F **NMR** (376 MHz, $CDCl_3$) δ -112.9.

HRMS (CI+) Calcd for $C_{18}H_{17}FO_2$ [M]: 284.1213, Found: 284.1218.

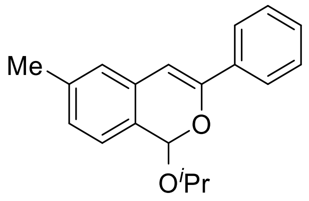

1m

1-Isopropoxy-6-methyl-3-phenyl-1*H*-isochromene (1m) was prepared according to the General Procedure A as a white solid (chromatography eluent: *n*-hexane/EtOAc = 50:1) in 75% yield (2.1 g).

^{1}H **NMR** (400 MHz, $CDCl_3$) δ 7.79 – 7.76 (m, 2H), 7.39 – 7.29 (m, 3H), 7.11 – 7.00 (m, 3H), 6.54 (s, 1H), 6.26 (s, 1H), 4.33 (hept, *J* = 6.4 Hz, 1H), 2.33 (s, 3H), 1.28 (d, *J* = 6.0 Hz, 3H), 1.15 (d, *J* = 6.4 Hz, 3H).

^{13}C **NMR** (101 MHz, $CDCl_3$) δ 149.3, 138.8, 134.8, 130.2, 128.5, 128.3, 127.4, 125.3, 124.98, 124.96, 124.8, 100.3, 96.8, 69.6, 23.5, 21.9, 21.3.

HRMS (CI+) Calcd for $C_{19}H_{20}O_2$ [M]: 280.1463, Found: 280.1467.

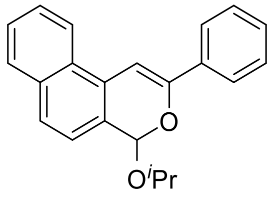

1n

4-Isopropoxy-2-phenyl-4*H*-benzo[*f*]isochromene (1n) was prepared according to the General Procedure A as a yellow solid (chromatography eluent: *n*-hexane/EtOAc = 50:1) in 66% yield (1.8 g).

^{1}H NMR (400 MHz, $CDCl_3$) δ 8.24 (d, *J* = 8.4 Hz, 1H), 7.91 – 7.89 (m, 2H), 7.81 – 7.79 (m, 1H), 7.71 (d, *J* = 8.4 Hz, 1H), 7.55 – 7.27 (m, 7H), 6.42 (s, 1H), 4.39 (hept, *J* = 6.0 Hz, 1H), 1.31 (d, *J* = 6.0 Hz, 3H), 1.18 (d, *J* = 6.4 Hz, 3H).

^{13}C NMR (101 MHz, $CDCl_3$) δ 150.3, 134.9, 133.9, 128.7, 128.5, 128.4, 128.0, 126.8, 126.5, 126.2, 126.1, 125.0, 123.6, 123.4, 122.9, 97.4, 96.0, 69.8, 23.6, 22.0.

HRMS (CI+) Calcd for $C_{22}H_{20}O_2$ [M]: 316.1463, Found: 316.1463.

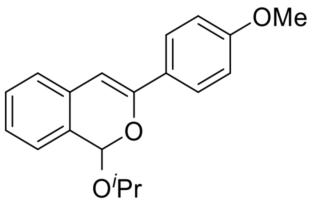

1o

1-Isopropoxy-3-(4-methoxyphenyl)-1*H*-isochromene (1o) was prepared according to the General Procedure A as a white solid (chromatography eluent: *n*-hexane/EtOAc = 50:1) in 52% yield (0.4 g).

^{1}H NMR (400 MHz, $CDCl_3$) δ 7.76 (d, *J* = 7.6 Hz, 2H), 7.39 (t, *J* = 7.6 Hz, 2H), 7.32 (d, *J* = 7.2 Hz, 1H), 7.15 (d, *J* = 8.4 Hz, 1H), 6.90 (dd, J_1 = 8.4 Hz, J_2 = 2.4 Hz, 1H), 6.78 (d, *J* = 2.4 Hz, 1H), 6.57 (s, 1H), 6.25 (s, 1H), 4.36 (hept, *J* = 6.0 Hz, 1H), 3.83 (s, 3H), 1.32 (d, *J* = 6.4 Hz, 3H), 1.17 (d, *J* = 6.0 Hz, 3H).

^{13}C NMR (101 MHz, $CDCl_3$) δ 147.5, 134.9, 129.0, 128.4 (2C), 128.2, 125.9, 124.5, 123.5, 115.2, 110.8, 100.1, 96.8, 69.7, 55.5, 23.5, 21.9.

HRMS (CI+) Calcd for $C_{22}H_{20}O_2$ [M]: 296.1412, Found: 296.1407.

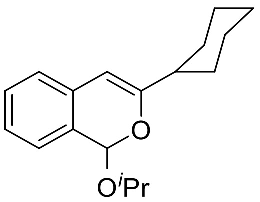

1q

3-Cyclohexyl-1-isopropoxy-1*H*-isochromene (1q) was prepared according to the General Procedure A as a yellow solid (chromatography eluent: *n*-hexane/EtOAc = 50:1) in 88% yield (1.2 g).

^{1}H NMR (400 MHz, $CDCl_3$) δ 7.27 – 7.23 (m, 1H), 7.18 – 7.11 (m, 2H), 7.03 (d, *J* = 7.6 Hz, 1H), 6.10 (s, 1H), 5.74 (s, 1H), 4.26 (hept, *J* = 6.4 Hz, 1H), 2.19 – 2.13 (m, 1H), 1.97 – 1.94 (m, 2H), 1.82 – 1.80 (m, 2H), 1.72 – 1.69 (m, 1H), 1.40 – 1.27 (m, 8H), 1.20 (d, *J* = 6.4 Hz, 3H).

^{13}C NMR (101 MHz, $CDCl_3$) δ 158.4, 130.5, 128.9, 126.8, 125.7, 125.5, 123.7, 97.9, 96.1, 68.8, 42.3, 30.8, 30.5, 26.23, 26.16, 23.4, 21.6.

HRMS (CI+) Calcd for $C_{18}H_{24}O_2$ [M]: 272.1776, Found: 272.1773.

III. Synthesis of Catalyst (*R*)-A8

The CPA catalyst (*R*)-**A8** was prepared by following the literature procedure.[3]

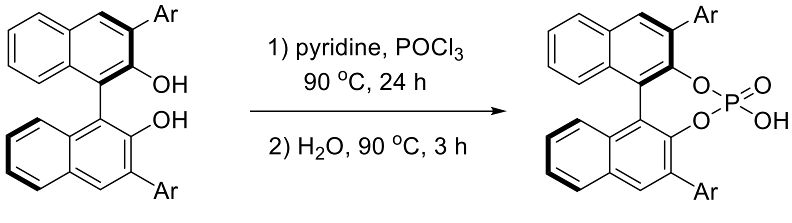

SA8 (*R*)-**A8**

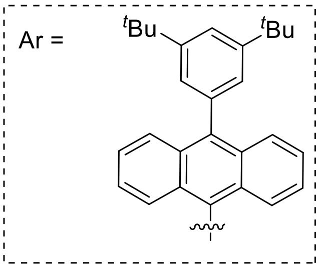

(*R*)-2,6-Bis(10-(3,5-di-*tert*-butylphenyl)anthracen-9-yl)-4-hydroxydinaphtho [2,1-d:1',2'-f][1,3,2]dioxaphosphepine 4-oxide (A8). At room temperature, to a 100-mL round-bottom flask charged with the substituted binaphthol **SA8** (1.2 g, 1.2 mmol) was added anhydrous pyridine (20 mL). The flask was cooled to 0 °C, and $POCl_3$ (1.8 g, 12.0 mmol, 10.0 equiv) was slowly added. The mixture was heated at 90 °C for 24 h and then cooled to room temperature. Water (5.0 mL) was cautiously added and then the mixture was heated again at 90 °C for 3 h. The mixture was cooled to room temperature and then poured into an aqueous HCl solution (1 N, 50 mL) and extracted with DCM (50 mL × 3). The combined organic layers were sequentially washed with an aqueous HCl solution (1 N, 50 mL × 3), brine (50 mL), dried with anhydrous Na_2SO_4, filtered, and concentrated *in vacuo*. The residue was purified by silica gel column

3. W. Guo, Y. Luo, H. Sung, I. D. Williams, P. Li and J. Sun, *J. Am. Chem. Soc.*, 2020, **142**, 14384–14390.

chromatography (eluent: DCM/MeOH = 50:1 → 20:1) to afford the desired product. The pure product was redissolved in DCM (50 mL), and vigorously washed with an aqueous HCl solution (4 N, 50 mL × 3) to acidify the phosphoric acid and remove salt impurities. Finally, the organic layer was dried over Na_2SO_4, filtered, and concentrated *in vacuo* to furnish the phosphoric acid **A8** as a light yellow solid (1.5 g, 85% yield).

$[\alpha]_D^{23}$: +0.9 (c = 1.0, CH_2Cl_2).

^{1}H NMR (400 MHz, $CDCl_3$) δ 8.08 (s, 2H), 8.00 (d, *J* = 8.0 Hz, 2H), 7.80 – 7.74 (m, 4H), 7.69 – 7.60 (m, 8H), 7.54 – 7.50 (m, 4H), 7.28 – 7.23 (m, 8H), 7.17 – 7.10 (m, 4H), 4.09 (s, 6H), 1.38 (s, 18H), 1.26 (s, 18H).

^{13}C NMR (101 MHz, $CDCl_3$) δ 150.7, 150.6, 146.8, 146.7, 139.6, 138.0, 134.3, 132.9, 131.7, 131.44, 131.41, 131.1, 130.8, 130.3, 130.1, 129.8, 128.7, 128.1, 127.63, 127.55, 127.1, 126.9, 126.4, 126.3, 125.8, 124.9, 124.7, 122.6, 121.0, 35.1, 35.0, 31.8, 31.7.

^{31}P NMR (162 MHz, $CDCl_3$) δ 2.2.

HRMS (ES-) Calcd for $C_{76}H_{68}O_4P^-$ [M - H]$^-$: 1075.4860, Found: 1075.4854.

IV. CPA-Catalyzed Synthesis of Bridged Cyclopropanes

General Procedure B.

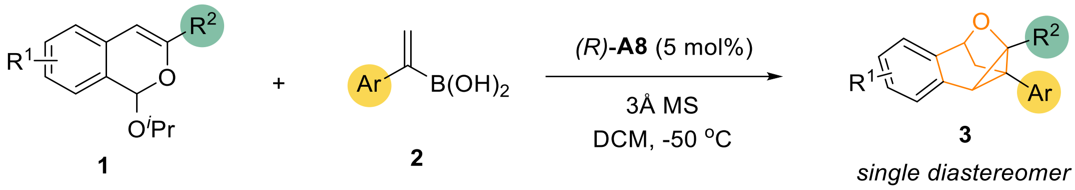

At –50 °C, to a solution of 3Å molecular sieves (30.0 mg), (*R*)-**A8** (16.1 mg, 0.015 mmol, 5 mol%) and vinylboronic acid **2** (0.75 mmol) in DCM (1.5 mL) in a 4-mL vial was added acetal **1** (0.30 mmol). The reaction mixture was stirred at –50 °C under N_2 atmosphere for 96 h. The reaction mixture was quenched by Et_3N (1 drop) and transferred to a flask with DCM (10 mL) and concentrated. Then the residue was directly purified by silica gel column chromatography to afford the desired product **3**.

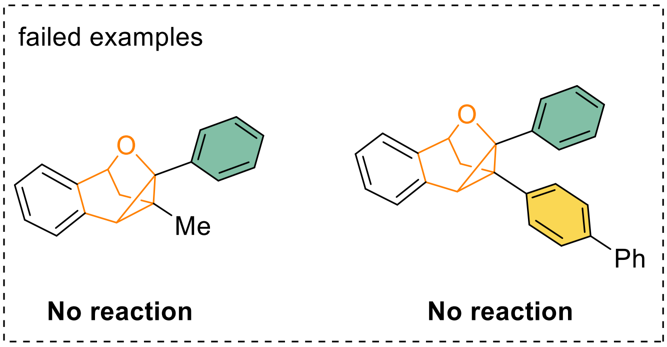

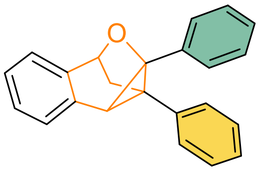

3a

(1*S*,3*S*,7b*S*)-1,1a-Diphenyl-1a,2,3,7b-tetrahydro-1*H*-1,3-epoxycyclopropa[*a*]naphthalene (3a) was prepared according to the General Procedure B as a white solid (chromatography eluent: *n*-hexane/EtOAc = 100:1) in 78% yield (72.3 mg, 96% ee).

$[\alpha]_D^{23}$: +129.2 (c = 1.0, CH_2Cl_2). HPLC analysis of the product: Daicel CHIRALPAK® IC column; 2% ^{i}PrOH in *n*-hexane; 1.0 mL/min; retention times: 8.1 min (major), 7.0 min (minor).

^{1}H NMR (400 MHz, $CDCl_3$) δ 7.46 (d, *J* = 7.2 Hz, 1H), 7.36 – 7.32 (m, 1H), 7.27 – 7.16 (m, 12H), 5.32 (d, *J* = 6.0 Hz, 1H), 3.22 (s, 1H), 2.66 – 2.61 (m, 1H), 1.73 (d, *J* = 11.6 Hz, 1H).

^{13}C NMR (101 MHz, $CDCl_3$) δ 137.4, 135.6, 135.3, 132.2, 128.9, 128.4, 127.91, 127.85, 126.9, 126.8, 126.7, 126.3, 125.3, 122.3, 74.8, 68.5, 37.1, 35.7, 31.8.

HRMS (CI+) Calcd for $C_{23}H_{18}O$ [M]: 310.1358, Found: 310.1362.

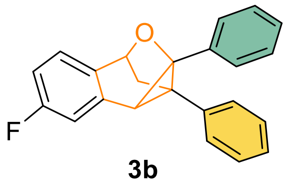

3b

(1*S*,1a*R*,3*R*)-6-Fluoro-1,1a-diphenyl-1a,2,3,7b-tetrahydro-1*H*-1,3-epoxycyclo propa[*a*]naphthalene (3b) was prepared according to the General Procedure B as a white solid (chromatography eluent: *n*-hexane/EtOAc = 100:1) in 64% yield (61.5 mg, 96% ee).

$[\alpha]_D^{23}$: +45.0 (c = 1.0, CH_2Cl_2). HPLC analysis of the product: Daicel CHIRALPAK® IC column; 2% ^{i}PrOH in *n*-hexane; 1.0 mL/min; retention times: 6.3 min (major), 5.8 min (minor).

^{1}H NMR (400 MHz, $CDCl_3$) δ 7.26 – 7.15 (m, 12H), 6.95 – 6.90 (m, 1H), 5.31 (d, *J* = 6.0 Hz, 1H), 3.18 (s, 1H), 2.65 (dd, J_1 = 11.6 Hz, J_2 = 6.0 Hz, 1H), 1.72 (d, *J* = 12.0 Hz, 1H).

^{13}C NMR (101 MHz, $CDCl_3$) δ 162.6 (d, *J* = 243.0 Hz), 136.9, 134.9, 134.4 (d, *J* = 9.0 Hz), 131.5 (d, *J* = 3.0 Hz), 128.8, 128.4, 127.9, 127.1, 126.8, 126.3, 123.9 (d, *J* = 8.0 Hz), 113.8 (d, *J* = 22.4 Hz), 111.9 (d, *J* = 21.0 Hz), 74.2, 68.5, 37.3, 35.9, 31.9 (d, *J* = 2.0 Hz).

HRMS (CI+) Calcd for $C_{23}H_{17}FO$ [M]: 328.1263, Found: 328.1264.

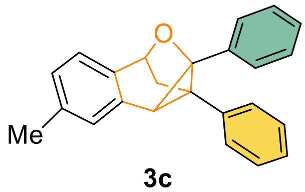

3c

(1*S*,3*S*,7b*S*)-6-Methyl-1,1a-diphenyl-1a,2,3,7b-tetrahydro-1*H*-1,3-epoxycyclo propa[*a*]naphthalene (3c) was prepared according to the General Procedure B as a yellow solid (chromatography eluent: *n*-hexane/EtOAc = 100:1) in 48% yield (40.3 mg, 99% ee).

$[\alpha]_D^{23}$: +78.8 (c = 1.0, CH_2Cl_2). HPLC analysis of the product: Daicel CHIRALPAK® IC column; 2% ^{i}PrOH in *n*-hexane; 1.0 mL/min; retention times: 8.6 min (major), 8.0 min (minor).

^{1}H NMR (400 MHz, $CDCl_3$) δ 7.27 (s, 1H), 7.26 – 7.13 (m, 11H), 7.06 – 7.04 (m, 1H), 5.29 (d, *J* = 6.0 Hz, 1H), 3.16 (s, 1H), 2.64 – 2.60 (m, 1H), 2.39 (s, 1H), 1.73 (d, *J* = 12.0 Hz, 1H).

^{13}C NMR (101 MHz, $CDCl_3$) δ 137.6, 137.5, 135.4, 132.9, 132.1, 128.9, 128.4, 127.8, 127.4, 126.8, 126.6, 126.3, 125.9, 122.2, 74.6, 68.5, 37.5, 35.8, 31.8, 21.5.

HRMS (CI+) Calcd for $C_{24}H_{20}O$ [M]: 324.1514, Found: 324.1507.

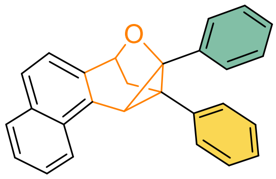

3d

1,1a-Diphenyl-1a,2,3,9c-tetrahydro-1*H*-1,3-epoxycyclopropa[*c*]phenanthrene (3d) was prepared according to the General Procedure B as a yellow solid (chromatography eluent: *n*-hexane/EtOAc = 100:1) in 67% yield (72.4 mg, 97% ee).

$[\alpha]_D^{23}$: -4.9 (c = 1.0, CH_2Cl_2). HPLC analysis of the product: Daicel CHIRALPAK® IC column; 2% ^{i}PrOH in *n*-hexane; 1.0 mL/min; retention times: 7.5 min (major), 6.0 min (minor).

^{1}H NMR (400 MHz, $CDCl_3$) δ 8.32 (d, *J* = 8.4 Hz, 1H), 7.89 (d, *J* = 8.4 Hz, 1H), 7.73 (d, *J* = 8.0 Hz, 1H), 7.57 (t, *J* = 7.2 Hz, 1H), 7.49 (t, *J* = 7.2 Hz, 1H), 7.41 (d, *J* = 8.0 Hz, 1H), 7.29 – 7.16 (m, 10H), 5.46 (d, *J* = 6.0 Hz, 1H), 3.92 (s, 1H), 2.68 (dd, J_1 = 12.0 Hz, J_2 = 6.0 Hz, 1H), 1.73 (d, *J* = 11.6 Hz, 1H).

^{13}C NMR (75 MHz, $CDCl_3$) δ 137.4, 135.3, 133.5, 132.4, 130.9, 129.0 (2C), 128.5, 127.9, 127.8, 127.0, 126.8, 126.5, 126.1, 125.4, 125.2, 122.1, 121.6, 75.1, 68.7, 36.9, 35.7, 28.2.

HRMS (CI+) Calcd for $C_{27}H_{20}NaO^+$ [M + Na]$^+$: 383.1406, Found: 383.1411.

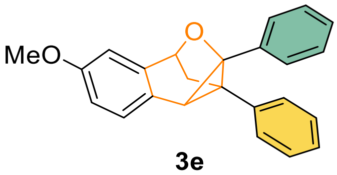

3e

(1*S*,3*S*,7b*S*)-5-Methoxy-1,1a-diphenyl-1a,2,3,7b-tetrahydro-1*H*-1,3-epoxycyclopropa[*a*]naphthalene (3e) was prepared according to the General Procedure B as a white solid (chromatography eluent: *n*-hexane/EtOAc = 100:1) in 66% yield (58.4 mg, 99% ee).

$[\alpha]_D^{23}$: +8.9 (c = 1.0, CH_2Cl_2). HPLC analysis of the product: Daicel CHIRALPAK® IC column; 2% ^{i}PrOH in *n*-hexane; 1.0 mL/min; retention times: 14.2 min (major).

^{1}H NMR (400 MHz, $CDCl_3$) δ 7.34 (d, *J* = 8.0 Hz, 1H), 7.25 – 7.15 (m, 10H), 6.90 – 6.88 (m, 1H), 6.83 (d, *J* = 2.4 Hz, 1H), 5.26 (d, *J* = 6.0 Hz, 1H), 3.81 (s, 3H), 3.16 (s, 1H), 2.62 (ddd, J_1 = 12.0 Hz, J_2 = 6.4 Hz, J_3 = 0.8 Hz, 1H), 1.73 (d, *J* = 12.0 Hz, 1H).

^{13}C NMR (101 MHz, $CDCl_3$) δ 157.8, 137.5, 136.7, 135.4, 128.9, 128.3, 127.8, 127.6, 126.8, 126.6, 126.2, 124.3, 113.3, 108.6, 74.9, 68.4, 55.5, 37.2, 35.6, 31.0.

HRMS (CI+) Calcd for $C_{24}H_{20}NaO_2^+$ [M + Na]$^+$: 363.1356, Found: 363.1362.

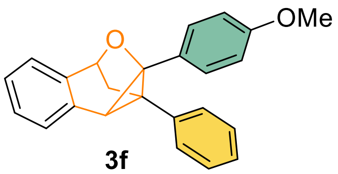

3f

(1*S*,3*S*,7b*S*)-1-(4-Methoxyphenyl)-1a-phenyl-1a,2,3,7b-tetrahydro-1*H*-1,3-epoxycyclopropa[*a*]naphthalene (3f) was prepared according to the General Procedure B as a white solid (chromatography eluent: *n*-hexane/EtOAc = 100:1) in 87% yield (77.1 mg, 99% ee).

$[\alpha]_D^{23}$: +138.6 (c = 1.0, CH_2Cl_2). HPLC analysis of the product: Daicel CHIRALPAK® IC column; 2% ^{i}PrOH in *n*-hexane; 1.0 mL/min; retention times: 26.1 min (major), 16.0 min (minor).

^{1}H NMR (400 MHz, $CDCl_3$) δ 7.45 – 7.43 (m, 1H), 7.35 – 7.30 (m, 1H), 7.25 – 7.20 (m, 4H), 7.18 – 7.14 (m, 5H), 6.76 – 6.73 (m, 2H), 5.28 (d, *J* = 6.0 Hz, 1H), 3.71 (s, 3H), 3.16 (s, 1H), 2.66 (ddd, J_1 = 12.0 Hz, J_2 = 6.0 Hz, J_3 = 0.8 Hz, 1H), 1.71 (d, *J* = 11.6 Hz, 1H).

^{13}C NMR (101 MHz, $CDCl_3$) δ 158.7, 137.7, 135.7, 134.0, 132.3, 128.5, 128.3, 127.8, 126.9, 126.7, 126.4, 125.2, 122.3, 113.4, 74.7, 68.7, 55.1, 36.5, 34.7, 31.3.

HRMS (CI+) Calcd for $C_{24}H_{20}O_2$ [M]: 340.1463, Found: 340.1463.

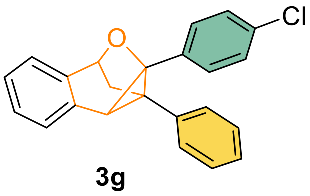

3g

(1*S*,3*S*,7b*S*)-1-(4-Chlorophenyl)-1a-phenyl-1a,2,3,7b-tetrahydro-1*H*-1,3-epoxycyclopropa[*a*]naphthalene (3g) was prepared according to the General Procedure B as a yellow solid (chromatography eluent: *n*-hexane/EtOAc = 100:1) in 84% yield (75.7 mg, 99% ee).

$[\alpha]_D^{23}$: +147.4 (c = 1.0, CH_2Cl_2). HPLC analysis of the product: Daicel CHIRALPAK® IC column; 2% ^{i}PrOH in *n*-hexane; 1.0 mL/min; retention times: 6.1 min (major), 5.8 min (minor).

^{1}H NMR (400 MHz, $CDCl_3$) δ 7.45 (d, *J* = 7.6 Hz, 1H), 7.36 – 7.33 (m, 1H), 7.28 – 7.23 (m, 4H), 7.22 – 7.14 (m, 5H), 7.11 – 7.08 (m, 2H), 5.31 (d, *J* = 6.0 Hz, 1H), 3.18 (s, 1H), 2.61 (ddd, J_1 = 12.0 Hz, J_2 = 6.0 Hz, J_3 = 0.8 Hz, 1H), 1.73 (d, *J* = 12.0 Hz, 1H).

^{13}C NMR (101 MHz, $CDCl_3$) δ 136.9, 135.5, 134.1, 132.6, 131.9, 129.0, 128.5, 128.0 (2C), 127.4, 126.9, 126.8, 125.5, 122.4, 74.9, 68.0, 37.2, 36.0, 31.8.

HRMS (CI+) Calcd for $C_{23}H_{17}ClO$ [M]: 344.0968, Found: 344.0965.

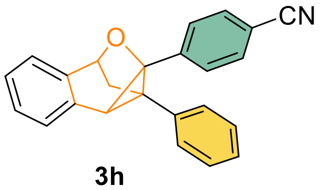

3h

4-((1*S*,3*S*,7b*S*)-1a-Phenyl-1a,2,3,7b-tetrahydro-1*H*-1,3-epoxycyclopropa[*a*] naphthalen-1-yl)benzonitrile (3h) was prepared according to the General Procedure B (temperature: –35 °C, catalyst loading: 10 mol%) as a white solid (chromatography eluent: *n*-hexane/EtOAc = 100:1) in 71% yield (71.1 mg, 93% ee).

$[\alpha]_D^{23}$: +252.0 (c = 1.0, CH_2Cl_2). HPLC analysis of the product: Daicel CHIRALPAK® IC column; 5% ^{i}PrOH in *n*-hexane; 1.0 mL/min; retention times: 42.5 min (major), 19.9 min (minor).

^{1}H NMR (400 MHz, $CDCl_3$) δ 7.47 – 7.43 (m, 3H), 7.37 (td, J_1 = 6.8 Hz, J_2 = 2.4 Hz, 1H), 7.31 – 7.25 (m, 5H), 7.21 – 7.18 (m, 4H), 5.37 (d, *J* = 6.0 Hz, 1H), 3.26 (s, 1H), 2.61 (ddd, J_1 = 12.0 Hz, J_2 = 6.0 Hz, J_3 = 0.4 Hz, 1H), 1.77 (d, *J* = 12.0 Hz, 1H).

^{13}C NMR (101 MHz, $CDCl_3$) δ 141.9, 136.2, 135.3, 131.5, 131.4, 129.3, 128.7, 128.2, 127.3, 126.9, 125.8 (2C), 122.5, 119.0, 110.0, 75.1, 67.8, 37.8, 37.6, 33.0.

HRMS (CI+) Calcd for $C_{24}H_{18}NO^+$ $[M + H]^+$: 336.1383, Found: 336.1397.

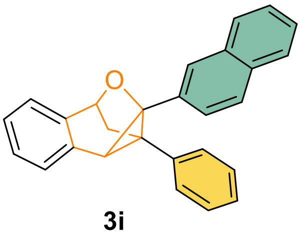

3i

(1*S*,1a*R*,3*R*)-1-(Naphthalen-2-yl)-1a-phenyl-1a,2,3,7b-tetrahydro-1*H*-1,3-epoxycyclopropa[*a*]naphthalene (3i) was prepared according to the General Procedure B (temperature: –35 °C, catalyst loading: 10 mol%) as a yellow solid (chromatography eluent: *n*-hexane/EtOAc = 100:1) in 69% yield (75.0 mg, 94% ee).

$[\alpha]_D^{23}$: +139.9 (c = 1.0, CH_2Cl_2). HPLC analysis of the product: Daicel CHIRALPAK® IC column; 2% ^{i}PrOH in *n*-hexane; 1.0 mL/min; retention times: 13.5 min (major), 11.4 min (minor).

^{1}H NMR (400 MHz, $CDCl_3$) δ 7.87 (d, *J* = 0.8 Hz, 1H), 7.74 – 7.70 (m, 2H), 7.63 (d, *J* = 8.8 Hz, 1H), 7.50 (d, *J* = 7.2 Hz, 1H), 7.43 – 7.34 (m, 3H), 7.29 – 7.26 (m, 2H), 7.24 – 7.21 (m, 4H), 7.20 – 7.16 (m, 1H), 7.10 (dd, J_1 = 8.4 Hz, J_2 = 1.6 Hz, 1H), 5.39 (d, *J* = 6.0 Hz, 1H), 3.36 (s, 1H), 2.70 (ddd, J_1 = 12.0 Hz, J_2 = 6.0 Hz, J_3 = 0.8 Hz, 1H), 1.79 (d, *J* = 12.0 Hz, 1H).

^{13}C NMR (101 MHz, $CDCl_3$) δ 137.3, 135.7, 133.0, 132.9, 132.4, 132.2, 128.9, 128.4, 128.0, 127.8, 127.5, 127.4, 126.9, 126.7, 125.9, 125.6, 125.4 (2C), 124.2, 122.4, 75.0, 68.8, 37.3, 35.9, 32.0.

HRMS (CI+) Calcd for $C_{27}H_{20}O$ [M]: 360.1514, Found: 360.1527.

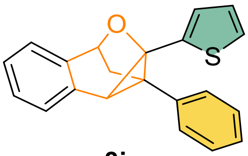

3j

(1*R*,1a*R*,3*R*)-1a-Phenyl-1-(thiophen-2-yl)-1a,2,3,7b-tetrahydro-1*H*-1,3-epoxycyclopropa[*a*]naphthalene (3j) was prepared according to the General

Procedure B (temperature: –35 °C, catalyst loading: 10 mol%) as a yellow solid (chromatography eluent: *n*-hexane/EtOAc = 100:1) in 52% yield (49.7 mg, 93% ee).

$[\alpha]_D^{23}$: +132.9 (c = 1.0, CH_2Cl_2). HPLC analysis of the product: Daicel CHIRALPAK® IC column; 2% ^{i}PrOH in *n*-hexane; 1.0 mL/min; retention times: 13.3 min (major), 8.7 min (minor).

^{1}H NMR (400 MHz, $CDCl_3$) δ 7.47 (d, J = 7.2 Hz, 1H), 7.36 (td, J_1 = 7.2 Hz, J_2 = 1.6 Hz, 1H), 7.28 – 7.20 (m, 7H), 7.15 (d, J = 5.2 Hz, 1H), 6.84 (dd, J_1 = 4.8 Hz, J_2 = 3.6 Hz, 1H), 6.68 – 6.67 (m, 1H), 5.31 (d, J = 6.0 Hz, 1H), 3.22 (s, 1H), 2.69 (dd, J_1 = 12.0 Hz, J_2 = 6.0 Hz, 1H), 1.72 (d, J = 11.6 Hz, 1H).

^{13}C NMR (101 MHz, $CDCl_3$) δ 138.4, 136.9, 135.4, 131.7, 128.8, 128.4 (2C), 128.0, 126.9, 126.5, 125.5, 124.8, 124.3, 122.5, 75.3, 66.6, 36.5, 35.8, 32.4.

HRMS (CI+) Calcd for $C_{21}H_{16}OS$ [M]: 316.0922, Found: 316.0924.

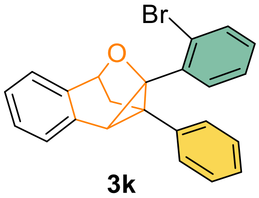

3k

(1*R*,1a*R*,3*R*)-1-(2-Bromophenyl)-1a-phenyl-1a,2,3,7b-tetrahydro-1*H*-1,3-epoxycyclopropa[*a*]naphthalene (3k) was prepared according to the General Procedure B as a yellow oil (chromatography eluent: *n*-hexane/EtOAc = 100:1) in 57% yield (67.0 mg, 96% ee).

$[\alpha]_D^{23}$: -41.9 (c = 1.0, CH_2Cl_2). HPLC analysis of the product: Daicel CHIRALPAK® IC column; 2% ^{i}PrOH in *n*-hexane; 1.0 mL/min; retention times: 14.5 min (major), 9.2 min (minor).

^{1}H NMR (400 MHz, $CDCl_3$) δ 7.52 (dd, J_1 = 8.0 Hz, J_2 = 1.2 Hz, 1H), 7.47 – 7.44 (m, 2H), 7.34 (td, J_1 = 7.2 Hz, J_2 = 2.0 Hz, 1H), 7.29 – 7.13 (m, 7H), 6.98 – 6.96 (m, 2H), 5.32 (d, J = 6.0 Hz, 1H), 3.17 (s, 1H), 3.05 – 3.01 (m, 1H), 1.78 (d, J = 11.6 Hz, 1H).

^{13}**C NMR** (101 MHz, $CDCl_3$) δ 138.4, 135.8, 133.53, 133.46, 132.9, 132.2, 130.4, 128.0, 127.9, 127.4, 127.2, 126.8, 125.8, 125.6, 125.4, 122.3, 75.3, 71.2, 33.1, 33.0, 32.4.

HRMS (CI+) Calcd for $C_{23}H_{17}BrO$ [M]: 388.0463, Found: 388.0367.

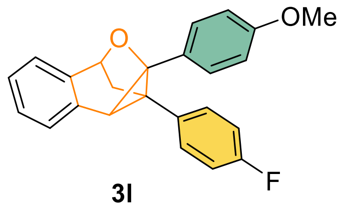

3l

(1*S*,3*S*,7b*S*)-1a-(4-Fluorophenyl)-1-(4-methoxyphenyl)-1a,2,3,7b-tetrahydro-1*H*-1,3-epoxycyclopropa[*a*]naphthalene (3l) was prepared according to the General Procedure B as a white solid (chromatography eluent: *n*-hexane/EtOAc = 100:1) in 72% yield (77.5 mg, 95% ee).

$[\alpha]_D^{23}$: -0.2 (c = 1.0, CH_2Cl_2). HPLC analysis of the product: Daicel CHIRALPAK® IC column; 2% ^{i}PrOH in *n*-hexane; 1.0 mL/min; retention times: 25.6 min (major), 16.7 min (minor).

^{1}**H NMR** (400 MHz, $CDCl_3$) δ 7.44 (d, *J* = 7.2 Hz, 1H), 7.36 – 7.32 (m, 1H), 7.27 – 7.22 (m, 2H), 7.17 – 7.11 (m, 4H), 6.94 – 6.90 (m, 2H), 6.79 – 6.75 (m, 2H), 5.28 (d, *J* = 6.0 Hz, 1H), 3.75 (s, 3H), 3.12 (s, 1H), 2.63 – 2.58 (m, 1H), 1.70 (d, *J* = 11.6 Hz, 1H).

^{13}**C NMR** (101 MHz, $CDCl_3$) δ 161.5 (d, *J* = 244.0 Hz), 158.8, 135.6, 133.5 (d, *J* = 3.0 Hz), 132.1, 130.2 (d, *J* = 7.0 Hz), 128.1, 127.9, 126.7, 125.3, 122.4, 115.4, 115.1, 113.5, 74.7, 68.5, 55.2, 36.8, 34.3, 31.2.

HRMS (ES+) Calcd for $C_{24}H_{19}FNaO_2^+$ $[M + Na]^+$: 381.1261, Found: 381.1269.

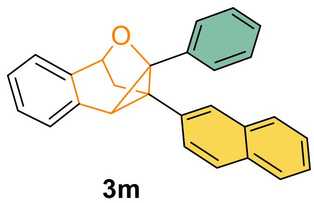

3m

(1*S*,3*S*,7b*S*)-1a-(Naphthalen-2-yl)-1-phenyl-1a,2,3,7b-tetrahydro-1*H*-1,3-epoxycyclopropa[*a*]naphthalene (3m) was prepared according to the General Procedure B as a white solid (chromatography eluent: *n*-hexane/EtOAc = 100:1) in 64% yield (68.8 mg, 89% ee).

$[\alpha]_D^{23}$: +216.5 (c = 1.0, CH_2Cl_2). HPLC analysis of the product: Daicel CHIRALPAK® IC column; 2% ^{i}PrOH in *n*-hexane; 1.0 mL/min; retention times: 9.6 min (major), 8.0 min (minor).

^{1}H NMR (400 MHz, $CDCl_3$) δ 7.74 – 7.64 (m, 4H), 7.49 (d, *J* = 7.2 Hz, 1H), 7.43 – 7.32 (m, 3H), 7.27 – 7.20 (m, 5H), 7.16 – 7.11 (m, 3H), 5.34 (d, *J* = 6.0 Hz, 1H), 3.34 (s, 1H), 2.73 – 2.67 (m, 1H), 1.75 (d, *J* = 12.0 Hz, 1H).

^{13}C NMR (101 MHz, $CDCl_3$) δ 135.6, 135.3, 135.0, 133.3, 132.2, 132.1, 128.0, 127.93, 127.87, 127.6, 127.5, 127.0, 126.9, 126.8, 126.2, 126.0, 125.7, 125.4, 122.3, 74.9, 68.6, 37.3, 36.0, 31.9.

HRMS (ES+) Calcd for $C_{27}H_{20}NaO^+$ [M + Na]$^+$: 383.1406, Found: 383.1411.

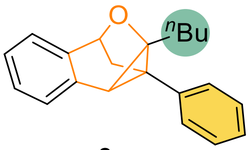

3n

(1*R*,3*S*,7b*S*)-1-Butyl-1a-phenyl-1a,2,3,7b-tetrahydro-1*H*-1,3-epoxycyclopropa [*a*]naphthalene (3n) was prepared according to the General Procedure B (room temperature, catalyst loading: 10 mol%, reaction time: 48 h) as a colorless oil (chromatography eluent: *n*-hexane/EtOAc = 100:1) in 53% yield (46.0 mg, 90% ee).

$[\alpha]_D^{23}$: -9.4 (c = 1.0, CH_2Cl_2). HPLC analysis of the product: Daicel CHIRALPAK® AD-H column; 3% ^{i}PrOH in *n*-hexane; 0.5 mL/min; retention times: 12.9 min (major), 10.0 min (minor).

^{1}H NMR (400 MHz, $CDCl_3$) δ 7.40 – 7.29 (m, 6H), 7.26 – 7.15 (m, 3H), 5.12 (d, *J* = 6.0 Hz, 1H), 2.61 (s, 1H), 2.52 (ddd, J_1 = 11.6 Hz, J_2 = 6.0 Hz, J_3 = 0.8 Hz, 1H), 2.03 – 1.96 (m, 1H), 1.53 – 1.36 (m, 4H), 1.26 – 1.18 (m, 2H), 0.78 (t, *J* = 7.2 Hz, 3H).

^{13}C NMR (101 MHz, $CDCl_3$) δ 138.6, 135.4, 133.0, 128.48, 128.45, 127.7, 126.5, 126.4, 124.8, 122.2, 74.7, 67.5, 36.3, 32.2, 29.0, 28.52, 28.47, 22.6, 13.9.

HRMS (ES+) Calcd for $C_{21}H_{22}NaO^+$ $[M + H]^+$: 313.1563, Found: 313.1568

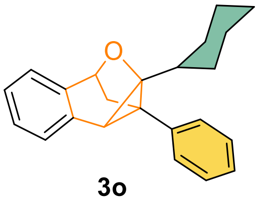

3o

(1*R*,3*S*,7b*S*)-1-Cyclohexyl-1a-phenyl-1a,2,3,7b-tetrahydro-1*H*-1,3-epoxycyclo propa[*a*]naphthalene (3o) was prepared according to the General Procedure B (room temperature, catalyst loading: 10 mol%, reaction time: 48 h) as a white solid (chromatography eluent: *n*-hexane/EtOAc = 100:1) in 59% yield (55.6 mg, 92% ee).

$[\alpha]_D^{23}$: -3.0 (c = 1.0, CH_2Cl_2). HPLC analysis of the product: Daicel CHIRALPAK® AD-H column; 2% ^{i}PrOH in *n*-hexane; 1.0 mL/min; retention times: 5.3 min (major), 4.6 min (minor).

^{1}H NMR (400 MHz, $CDCl_3$) δ 7.43 – 7.40 (m, 2H), 7.38 – 7.20 (m, 5H), 7.18 – 7.14 (m, 2H), 5.11 (d, *J* = 6.0 Hz, 1H), 2.66 (s, 1H), 2.60 – 2.55 (m, 1H), 1.75 (d, *J* = 12.4 Hz, 1H), 1.67 – 1.56 (m, 4H), 1.49 – 1.41 (m, 2H), 1.36 – 1.21 (m, 2H), 1.16 – 1.01 (m, 3H).

^{13}C NMR (101 MHz, $CDCl_3$) δ 138.3, 135.6, 133.0, 128.4, 128.3, 127.6, 126.34, 126.27, 124.7, 122.1, 74.3, 71.2, 37.9, 36.1, 33.0, 30.0, 28.9, 27.6, 26.4, 26.3, 26.2.

HRMS (ES+) Calcd for $C_{23}H_{24}NaO^+$ $[M + Na]^+$: 339.1719, Found: 339.1725.

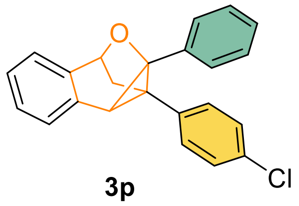

3p

(1*S*,3*R*,7b*R*)-1a-(4-Chlorophenyl)-1-phenyl-1a,2,3,7b-tetrahydro-1*H*-1,3-epoxycyclopropa[*a*]naphthalene (3p) was prepared according to the General Procedure B (room temperature, catalyst loading: 5 mol%, reaction time: 4 h) as a white solid (chromatography eluent: *n*-hexane/DCM/EtOAc = 100:25:1) in 76% yield (78.6 mg, 86% ee).

$[\alpha]_D^{23}$: +178.9 (c = 1.0, CH_2Cl_2). HPLC analysis of the product: Daicel CHIRALPAK® IC-3 column; 2% ^{i}PrOH in *n*-hexane; 1.0 mL/min; retention times: 9.1 min (major), 7.6 min (minor).

^{1}H NMR (400 MHz, $CDCl_3$) δ 7.45 – 7.43 (m, 1H), 7.36 – 7.32 (m, 1H), 7.25 – 7.18 (m, 9H), 7.12 – 7.10 (m, 2H), 5.31 (d, *J* = 6.0 Hz, 1H), 3.19 (s, 1H), 2.61 – 2.56 (m, 1H), 1.71 (d, *J* = 11.6 Hz, 1H).

^{13}C NMR (101 MHz, $CDCl_3$) δ 136.2, 135.7, 135.0, 132.6, 132.0, 130.3, 128.7,128.1(2C), 127.3, 126.9, 126.5, 125.6, 122.5, 77.5, 68.8, 37.2, 35.3, 31.7.

HRMS (ES+) Calcd for $C_{23}H_{18}ClO^+$ $[M + H]^+$: 345.1041, Found: 345.1025.

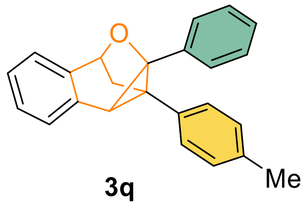

3q

(1*S*,3*R*,7b*R*)-1-Phenyl-1a-(*p*-tolyl)-1a,2,3,7b-tetrahydro-1*H*-1,3-epoxycyclo propa[*a*]naphthalene (3q) was prepared according to the General Procedure B (room temperature, catalyst loading: 5 mol%, reaction time: 12 h) as a white

solid (chromatography eluent: *n*-hexane/DCM/EtOAc = 100:25:1) in 58% yield (52.8 mg, 79% ee).

$[\alpha]_D^{23}$: +101.8 (c = 1.0, CH_2Cl_2). HPLC analysis of the product: Daicel CHIRALPAK® IC-3 column; 2% ^{i}PrOH in *n*-hexane; 1.0 mL/min; retention times: 8.9 min (major), 7.6 min (minor).

^{1}H NMR (400 MHz, $CDCl_3$) δ 7.45 (d, *J* = 7.6 Hz, 1H), 7.36 – 7.32 (m, 1H), 7.25 – 7.24 (m, 2H), 7.21 – 7.17 (m, 5H), 7.10 – 7.04 (m, 4H), 5.31 (d, *J* = 6.0 Hz, 1H), 3.18 (s, 1H), 2.62 (ddd, J_1 = 11.6 Hz, J_2 = 6.0 Hz, J_3 = 0.8 Hz, 1H), 2.29 (s, 3H), 1.71 (d, *J* = 11.6 Hz, 1H).

^{13}C NMR (101 MHz, $CDCl_3$) δ 136.4, 135.8, 135.6, 134.3, 132.4, 129.3, 128.9, 128.03, 127.98, 127.0, 126.9, 126.5, 125.4, 122.5, 75.0, 68.6, 37.4, 35.5, 31.9, 21.2.

HRMS (ES+) Calcd for $C_{24}H_{21}O^+$ $[M + H]^+$: 325.1587, Found: 325.1581.

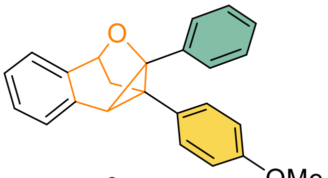

3r

(1*S*,3*R*,7b*R*)-1a-(4-Methoxyphenyl)-1-phenyl-1a,2,3,7b-tetrahydro-1*H*-1,3-epoxycyclopropa[*a*]naphthalene (3r) was prepared according to the General Procedure B as a white solid (chromatography eluent: *n*-hexane/DCM/EtOAc = 100:25:1) in 50% yield (51.0 mg, 44% ee).

$[\alpha]_D^{23}$: +43.1 (c = 1.0, CH_2Cl_2). HPLC analysis of the product: Daicel CHIRALPAK® IC-3 column; 2% ^{i}PrOH in *n*-hexane; 1.0 mL/min; retention times: 13.4 min (major), 11.3 min (minor).

^{1}H NMR (400 MHz, $CDCl_3$) δ 7.44 (d, *J* = 7.6 Hz, 1H), 7.36 – 7.32 (m, 1H), 7.27 – 7.16 (m, 7H), 7.15 – 7.10 (m, 2H), 6.81 – 6.77 (m, 2H), 5.30 (d, *J* = 6.0 Hz, 1H), 3.76 (s, 3H), 3.15 (s, 1H), 2.61 – 2.56 (m, 1H), 1.70 (d, *J* = 11.6 Hz, 1H).

^{13}C NMR (101 MHz, $CDCl_3$) δ 158.5, 135.8, 135.7, 132.5, 130.2, 129.3, 128.03, 127.99, 126.93, 126.91, 126.3, 125.4, 122.5, 114.0, 75.0, 68.4, 55.3, 37.5, 35.3, 32.0.

HRMS (ES+) Calcd for $C_{24}H_{21}O_2^+$ [M + H]$^+$: 341.1537, Found: 341.1531.

IV Large-Scale Reaction

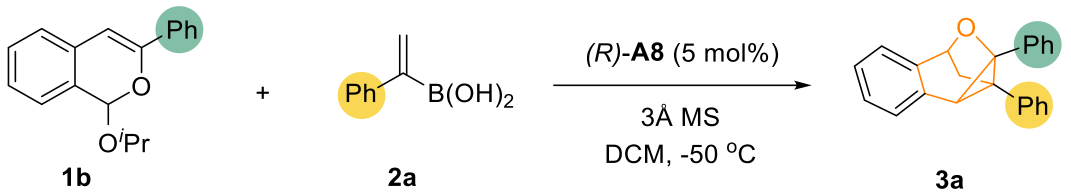

At –50 °C, to a solution of 3Å molecular sieves (300.0 mg), (*R*)-**A8** (0.15 mmol, 5 mol%) and vinylboronic acid **2a** (7.5 mmol) in DCM (15 mL) in a 50-mL flask was added acetal **1a** (3.0 mmol). The reaction mixture was stirred at –50 °C under N_2 atmosphere for 168 h before it was quenched by Et_3N (1 drop), transferred to a flask with DCM (50 mL) and concentrated. Then the residue was directly purified by silica gel column chromatography to afford the desired product **3a** (706.4 mg) in 76% yield and 98% ee.

V. Product Derivatizations

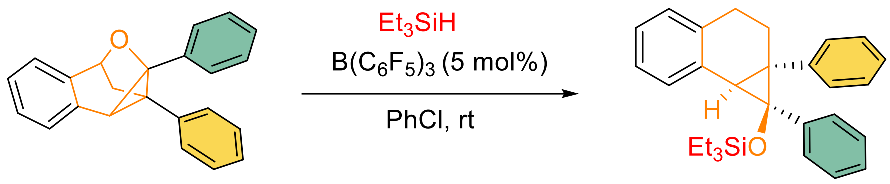

3a 4

(((1*S*,1a*R*)-1,1a-Diphenyl-1a,2,3,7b-tetrahydro-1*H*-cyclopropa[*a*]naphthalen-1-yl)oxy)triethylsilane (4). At room temperature, to a solution of **3a** (31.0 mg, 0.1 mmol) and $B(C_6F_5)_3$ (2.5 mg, 5 μmol) in anhydrous DCM (1.0 mL) was added triethylsilane (14.0 mg, 0.12 mmol). The mixture was kept stirring at room temperature for 1 h before it was quenched by Et_3N (1 drop). The mixture was concentrated under reduced pressure, and the residue was directly purified by silica gel column chromatography (eluent: *n*-hexane) to afford the desired product **4** in 93% yield, 99% ee (39.7 mg).

$[\alpha]_D^{23}$: +128.4 (c = 1.0, CH_2Cl_2). HPLC analysis of the product: Daicel CHIRALDICEL® OD-H column; 0.5 % ^{i}PrOH in *n*-hexane; 0.5 mL/min; retention times: 14.8 min (major), 12.7 min (minor).

^{1}H NMR (400 MHz, $CDCl_3$) δ 7.49 (d, *J* = 7.2 Hz, 1H), 7.30 – 7.28 (m, 2H), 7.24 – 6.99 (m, 10H), 6.96 – 6.92 (m, 1H), 3.22 (s, 1H), 3.07 – 3.01 (m, 1H), 2.85 – 2.74 (m, 2H), 2.07 – 2.00 (m, 1H), 0.61 (t, *J* = 8.0 Hz, 9H), 0.04 (q, *J* = 8.0 Hz, 6H).

^{13}C NMR (101 MHz, $CDCl_3$) δ 144.0, 141.5, 137.9, 133.2, 130.7, 128.7, 128.5, 128.4, 127.7, 127.4, 126.7, 125.9, 125.5, 125.4, 73.0, 38.0, 28.7, 28.6, 27.6, 6.8, 5.3.

HRMS (CI+) Calcd for $C_{29}H_{34}OSi$ [M]: 426.2379, Found: 426.2389.

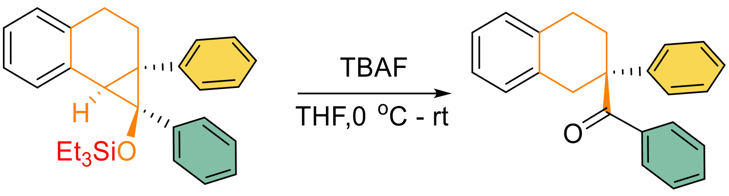

4 **5**

(*R*)-Phenyl(2-phenyl-1,2,3,4-tetrahydronaphthalen-2-yl)methanone (5). At 0 °C, to a solution of **4** (85.3 mg, 0.2 mmol) in THF (2.0 mL) was added TBAF (0.4 mL, 0.4 mmol, 1 M in THF). The mixture was kept stirring at room temperature for 1 h. The mixture was directly purified by silica gel column chromatography (eluent: *n*-hexane/EtOAc = 20:1) to afford the desired product **5** in 98% yield, 95% ee (61.2 mg).

$[\alpha]_D^{23}$: -3.3 (c = 1.0, CH_2Cl_2). HPLC analysis of the product: Daicel CHIRALPAK® IC-3 column; 2% ^{i}PrOH in *n*-hexane; 1.0 mL/min; retention times: 8.4 min (major), 7.4 min (minor).

^{1}H NMR (400 MHz, $CDCl_3$) δ 7.47 – 7.45 (m, 2H), 7.41 – 7.21 (m, 8H), 7.14 – 7.08 (m, 3H), 7.02 (d, *J* = 6.4 Hz, 1H), 3.42 (d, *J* = 16.8 Hz, 1H), 3.23 (d, *J* = 16.8 Hz, 1H), 2.69 – 2.63 (m, 1H), 2.57 – 2.45 (m, 3H).

^{13}C NMR (101 MHz, $CDCl_3$) δ 202.4, 142.9, 136.8, 135.6, 135.2, 131.9, 129.5, 129.3 (2C), 128.8, 128.2, 127.3, 126.4, 126.2, 126.0, 54.5, 39.6, 32.0, 26.2.

HRMS (ES+) Calcd for $C_{23}H_{20}NaO^+$ [M + Na]$^+$: 335.1407, Found: 335.1417.

VI. Product Structure Determination

The structure of product **3a** was determined by X-ray crystallography. The X-ray data have been deposited at the Cambridge Crystallographic Data Center (CCDC 2356608). The structures of other products were assumed by analogy.

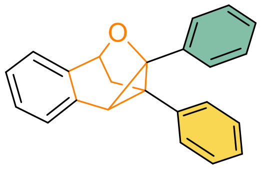

3a

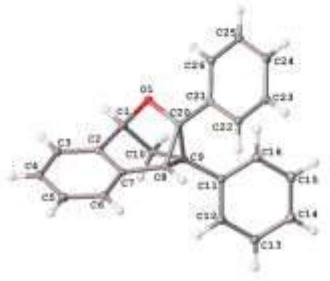

Table S1. Crystal Data and Structure Refinement for 3a.

Identification code	**3a**
Empirical formula	$C_{23}H_{18}O$
Formula weight	310.37
Temperature/K	100.00(10)
Crystal system	orthorhombic
Space group	$P2_12_12_1$
a/Å	10.33817(7)
b/Å	10.45428(8)
c/Å	14.51868(10)
α/°	90
β/°	90
γ/°	90
Volume/Å^3	1569.15(2)
Z	4
ρ_{calc}g/cm^3	1.314
μ/mm^{-1}	0.608
F(000)	656.0
Crystal size/mm^3	0.2 × 0.18 × 0.18

Radiation	Cu Kα (λ = 1.54184)
2Θ range for data collection/°	10.428 to 153.808
Index ranges	$-13 \leq h \leq 11$, $-12 \leq k \leq 12$, $-18 \leq l \leq 13$
Reflections collected	10133
Independent reflections	3252 [R_{int} = 0.0139, R_{sigma} = 0.0135]
Data/restraints/parameters	3252/0/218
Goodness-of-fit on F^2	1.174
Final R indexes [I>=2σ (I)]	R_1 = 0.0258, wR_2 = 0.0720
Final R indexes [all data]	R_1 = 0.0273, wR_2 = 0.0865
Largest diff. peak/hole / e $Å^{-3}$	0.24/-0.14
Flack parameter	0.01(6)

Table S2. Fractional Atomic Coordinates (×10^4) and Equivalent Isotropic Displacement Parameters (Å^2×10^3) for 3a. U_{eq} is Defined as 1/3 of the Trace of the Orthogonalised U_{IJ} Tensor.

Atom	*x*	*y*	*z*	U(eq)
O(1)	2270.1(11)	2588.2(11)	2858.8(8)	14.3(3)
C(1)	1417.8(16)	2320.2(16)	3638.2(11)	15.7(3)
C(2)	189.1(17)	3046.2(16)	3469.7(11)	15.5(3)
C(3)	-1048.4(16)	2528.6(17)	3402.7(11)	17.9(3)
C(4)	-2087.8(17)	3336.5(18)	3207.7(12)	20.0(4)
C(5)	-1891.0(17)	4644.0(18)	3084.3(12)	19.4(4)
C(6)	-654.0(16)	5159.8(17)	3155.0(11)	16.7(3)
C(7)	391.8(16)	4359.0(16)	3344.8(11)	14.2(3)
C(8)	1749.4(16)	4789.0(16)	3380.2(11)	14.1(3)
C(9)	2701.6(16)	4114.5(16)	4035.1(11)	14.6(3)
C(10)	2143.8(17)	2901.2(16)	4458.7(11)	16.3(3)
C(11)	3658.3(16)	4873.7(16)	4577.0(11)	15.3(3)
C(12)	3213.0(18)	5634.1(17)	5301.0(12)	19.1(4)
C(13)	4077.9(19)	6289.8(18)	5859.4(12)	22.2(4)
C(14)	5401.3(19)	6214.1(19)	5694.3(13)	22.8(4)
C(15)	5847.6(18)	5481.5(18)	4967.4(13)	22.6(4)
C(16)	4989.0(17)	4797.4(18)	4416.7(12)	19.1(4)
C(20)	2763.4(15)	3832.9(16)	3010.8(11)	13.5(3)
C(21)	3769.8(16)	4193.6(16)	2330.5(11)	14.2(3)
C(22)	4163.2(17)	5463.3(16)	2223.0(12)	16.6(3)
C(23)	5066.3(17)	5792.8(18)	1553.7(13)	19.6(4)
C(24)	5595.8(18)	4861.9(19)	985.6(13)	22.1(4)
C(25)	5232.3(17)	3589.7(18)	1101.4(13)	20.7(4)
C(26)	4319.6(17)	3259.9(16)	1763.8(12)	17.0(3)

Table S3. Anisotropic Displacement Parameters (Å^2 ×10^3) for 3a. The Anisotropic Displacement Factor Exponent Takes the Form: -2π^2[$h^2a^{*2}U_{11}$+2hka*b*U_{12}+…].

**Atom**	**U_{11}**	**U_{22}**	**U_{33}**	**U_{23}**	**U_{13}**	**U_{12}**
O(1)	17.3(6)	11.7(5)	13.9(5)	-0.3(4)	1.3(4)	-0.5(4)
C(1)	18.8(8)	14.1(7)	14.1(7)	0.7(6)	2.7(6)	0.7(6)
C(2)	18.1(8)	16.4(8)	12.0(7)	0.0(6)	2.3(6)	0.0(6)
C(3)	20.6(8)	16.5(8)	16.8(7)	-1.0(6)	3.5(6)	-2.7(7)
C(4)	14.9(7)	23.7(9)	21.6(8)	-2.0(7)	3.1(7)	-2.6(7)
C(5)	15.8(8)	22.6(9)	19.8(8)	-0.1(7)	1.6(6)	3.7(7)
C(6)	18.3(8)	16.2(8)	15.7(7)	1.1(6)	2.1(6)	1.0(6)
C(7)	15.2(8)	16.8(8)	10.6(7)	-0.2(6)	1.1(6)	-0.3(6)
C(8)	15.7(7)	11.9(7)	14.7(7)	0.0(6)	0.3(6)	1.3(6)
C(9)	16.2(8)	15.4(8)	12.1(7)	-0.5(6)	0.0(6)	1.3(6)
C(10)	19.5(8)	15.6(8)	13.7(7)	1.2(6)	-0.6(6)	1.3(6)
C(11)	18.7(8)	14.0(7)	13.3(7)	1.3(6)	-1.1(6)	-0.4(6)
C(12)	21.2(8)	19.3(8)	16.7(7)	-0.7(6)	2.7(7)	-1.5(7)
C(13)	33.0(10)	18.6(8)	15.0(8)	-3.0(6)	3.1(7)	-4.6(7)
C(14)	28.4(9)	21.8(9)	18.2(8)	1.0(7)	-7.1(7)	-4.3(7)
C(15)	19.0(8)	25.8(9)	23.1(9)	2.4(7)	-4.9(7)	1.2(7)
C(16)	18.4(8)	21.7(8)	17.3(8)	-1.1(7)	-2.1(6)	4.8(7)
C(20)	14.4(7)	12.6(8)	13.5(7)	-1.3(6)	-1.1(6)	-0.1(6)
C(21)	13.2(7)	17.7(8)	11.8(7)	-0.4(6)	-2.8(6)	1.3(6)
C(22)	15.3(7)	17.0(8)	17.6(8)	-2.3(6)	-0.2(6)	0.7(6)
C(23)	17.4(8)	20.0(8)	21.3(8)	-0.3(7)	0.1(7)	-2.5(6)
C(24)	16.8(8)	27.9(10)	21.5(8)	-0.9(7)	5.7(6)	-3.2(7)
C(25)	18.6(8)	22.7(9)	20.8(8)	-4.8(7)	3.8(7)	1.4(7)
C(26)	17.1(7)	16.6(8)	17.4(8)	-1.7(7)	-0.5(6)	1.4(6)

Table S4. Bond Lengths for 3a.

Atom	Atom	Length/Å	Atom	Atom	Length/Å
O(1)	C(1)	1.4613(19)	C(9)	C(20)	1.517(2)
O(1)	C(20)	1.415(2)	C(11)	C(12)	1.396(2)
C(1)	C(2)	1.500(2)	C(11)	C(16)	1.397(2)
C(1)	C(10)	1.533(2)	C(12)	C(13)	1.388(3)
C(2)	C(3)	1.392(2)	C(13)	C(14)	1.391(3)
C(2)	C(7)	1.400(2)	C(14)	C(15)	1.383(3)
C(3)	C(4)	1.396(3)	C(15)	C(16)	1.392(3)
C(4)	C(5)	1.394(3)	C(20)	C(21)	1.483(2)
C(5)	C(6)	1.392(2)	C(21)	C(22)	1.397(2)
C(6)	C(7)	1.395(2)	C(21)	C(26)	1.397(2)
C(7)	C(8)	1.475(2)	C(22)	C(23)	1.391(3)
C(8)	C(9)	1.540(2)	C(23)	C(24)	1.388(3)
C(8)	C(20)	1.545(2)	C(24)	C(25)	1.392(3)
C(9)	C(10)	1.523(2)	C(25)	C(26)	1.391(3)
C(9)	C(11)	1.492(2)			

Table S5. Bond Angles for 3a.

Atom	Atom	Atom	Angle/°	Atom	Atom	Atom	Angle/°
C(20)	O(1)	C(1)	105.84(12)	C(9)	C(10)	C(1)	101.62(13)
O(1)	C(1)	C(2)	106.70(13)	C(12)	C(11)	C(9)	118.77(15)
O(1)	C(1)	C(10)	103.33(13)	C(12)	C(11)	C(16)	118.85(16)
C(2)	C(1)	C(10)	109.93(13)	C(16)	C(11)	C(9)	122.30(15)
C(3)	C(2)	C(1)	126.36(15)	C(13)	C(12)	C(11)	120.57(16)
C(3)	C(2)	C(7)	120.62(16)	C(12)	C(13)	C(14)	120.31(17)
C(7)	C(2)	C(1)	112.98(15)	C(15)	C(14)	C(13)	119.40(17)
C(2)	C(3)	C(4)	119.10(16)	C(14)	C(15)	C(16)	120.66(17)
C(5)	C(4)	C(3)	120.48(16)	C(15)	C(16)	C(11)	120.18(17)
C(6)	C(5)	C(4)	120.32(16)	O(1)	C(20)	C(8)	113.87(13)
C(5)	C(6)	C(7)	119.62(16)	O(1)	C(20)	C(9)	108.43(13)
C(2)	C(7)	C(8)	115.90(15)	O(1)	C(20)	C(21)	112.50(13)
C(6)	C(7)	C(2)	119.86(16)	C(9)	C(20)	C(8)	60.37(10)
C(6)	C(7)	C(8)	124.16(16)	C(21)	C(20)	C(8)	122.87(14)
C(7)	C(8)	C(9)	119.37(14)	C(21)	C(20)	C(9)	129.26(14)
C(7)	C(8)	C(20)	115.88(14)	C(22)	C(21)	C(20)	121.34(15)
C(9)	C(8)	C(20)	58.94(10)	C(22)	C(21)	C(26)	118.65(15)
C(10)	C(9)	C(8)	112.88(14)	C(26)	C(21)	C(20)	119.99(15)
C(11)	C(9)	C(8)	120.38(14)	C(23)	C(22)	C(21)	120.58(16)
C(11)	C(9)	C(10)	118.75(14)	C(24)	C(23)	C(22)	120.40(17)
C(11)	C(9)	C(20)	126.30(14)	C(23)	C(24)	C(25)	119.44(17)
C(20)	C(9)	C(8)	60.69(10)	C(26)	C(25)	C(24)	120.22(17)
C(20)	C(9)	C(10)	104.49(13)	C(25)	C(26)	C(21)	120.67(16)

Table S6. Torsion Angles for 3a.

A	B	C	D	Angle/°	A	B	C	D	Angle/°
O(1)	C(1)	C(2)	C(3)	121.59(17)	C(9)	C(8)	C(20)	C(21)	-119.95(17)
O(1)	C(1)	C(2)	C(7)	-56.19(18)	C(9)	C(11)	C(12)	C(13)	175.81(16)
O(1)	C(1)	C(10)	C(9)	38.38(15)	C(9)	C(11)	C(16)	C(15)	-177.15(16)
O(1)	C(20)	C(21)	C(22)	165.02(14)	C(9)	C(20)	C(21)	C(22)	-53.7(2)
O(1)	C(20)	C(21)	C(26)	-13.3(2)	C(9)	C(20)	C(21)	C(26)	128.06(18)
C(1)	O(1)	C(20)	C(8)	-40.95(16)	C(10)	C(1)	C(2)	C(3)	-127.01(17)
C(1)	O(1)	C(20)	C(9)	24.06(16)	C(10)	C(1)	C(2)	C(7)	55.20(18)
C(1)	O(1)	C(20)	C(21)	173.39(13)	C(10)	C(9)	C(11)	C(12)	-76.0(2)
C(1)	C(2)	C(3)	C(4)	-177.54(15)	C(10)	C(9)	C(11)	C(16)	100.53(19)
C(1)	C(2)	C(7)	C(6)	178.18(15)	C(10)	C(9)	C(20)	O(1)	0.86(17)
C(1)	C(2)	C(7)	C(8)	1.2(2)	C(10)	C(9)	C(20)	C(8)	108.39(14)
C(2)	C(1)	C(10)	C(9)	-75.20(16)	C(10)	C(9)	C(20)	C(21)	-141.65(17)
C(2)	C(3)	C(4)	C(5)	-0.2(3)	C(11)	C(9)	C(10)	C(1)	-170.79(14)
C(2)	C(7)	C(8)	C(9)	-34.3(2)	C(11)	C(9)	C(20)	O(1)	144.57(15)
C(2)	C(7)	C(8)	C(20)	33.0(2)	C(11)	C(9)	C(20)	C(8)	-107.90(18)
C(3)	C(2)	C(7)	C(6)	0.3(2)	C(11)	C(9)	C(20)	C(21)	2.1(3)
C(3)	C(2)	C(7)	C(8)	-176.72(15)	C(11)	C(12)	C(13)	C(14)	1.1(3)
C(3)	C(4)	C(5)	C(6)	-0.1(3)	C(12)	C(11)	C(16)	C(15)	-0.6(3)
C(4)	C(5)	C(6)	C(7)	0.4(3)	C(12)	C(13)	C(14)	C(15)	0.1(3)
C(5)	C(6)	C(7)	C(2)	-0.5(2)	C(13)	C(14)	C(15)	C(16)	-1.6(3)
C(5)	C(6)	C(7)	C(8)	176.22(16)	C(14)	C(15)	C(16)	C(11)	1.9(3)
C(6)	C(7)	C(8)	C(9)	148.84(16)	C(16)	C(11)	C(12)	C(13)	-0.8(3)
C(6)	C(7)	C(8)	C(20)	-143.80(16)	C(20)	O(1)	C(1)	C(2)	76.63(15)
C(7)	C(2)	C(3)	C(4)	0.1(2)	C(20)	O(1)	C(1)	C(10)	-39.27(15)
C(7)	C(8)	C(9)	C(10)	10.0(2)	C(20)	C(8)	C(9)	C(10)	-94.27(15)
C(7)	C(8)	C(9)	C(11)	-138.52(16)	C(20)	C(8)	C(9)	C(11)	117.25(17)
C(7)	C(8)	C(9)	C(20)	104.23(17)	C(20)	C(9)	C(10)	C(1)	-23.75(16)
C(7)	C(8)	C(20)	O(1)	-11.7(2)	C(20)	C(9)	C(11)	C(12)	144.83(17)
C(7)	C(8)	C(20)	C(9)	-110.12(16)	C(20)	C(9)	C(11)	C(16)	-38.7(3)

C(7)	C(8)	C(20)	C(21)	129.92(16)	C(20)	C(21)	C(22)	C(23)	-177.12(15)
C(8)	C(9)	C(10)	C(1)	40.17(17)	C(20)	C(21)	C(26)	C(25)	177.81(16)
C(8)	C(9)	C(11)	C(12)	70.7(2)	C(21)	C(22)	C(23)	C(24)	-0.4(3)
C(8)	C(9)	C(11)	C(16)	-112.80(19)	C(22)	C(21)	C(26)	C(25)	-0.5(2)
C(8)	C(9)	C(20)	O(1)	-107.53(14)	C(22)	C(23)	C(24)	C(25)	-1.1(3)
C(8)	C(9)	C(20)	C(21)	109.97(19)	C(23)	C(24)	C(25)	C(26)	1.8(3)
C(8)	C(20)	C(21)	C(22)	22.9(2)	C(24)	C(25)	C(26)	C(21)	-1.0(3)
C(8)	C(20)	C(21)	C(26)	-155.37(15)	C(26)	C(21)	C(22)	C(23)	1.2(3)
C(9)	C(8)	C(20)	O(1)	98.40(15)					

Table S7. Hydrogen Atom Coordinates (Å×10^4) and Isotropic Displacement Parameters (Å^2×10^3) for 3a.

Atom	*x*	*y*	*z*	U(eq)
H(1)	1260	1383	3719	19
H(3)	-1183	1638	3489	22
H(4)	-2936	2993	3159	24
H(5)	-2605	5186	2951	23
H(6)	-523	6052	3074	20
H(8)	1912	5716	3254	17
H(10A)	1549	3100	4974	20
H(10B)	2836	2324	4681	20
H(12)	2310	5703	5413	23
H(13)	3765	6793	6357	27
H(14)	5993	6661	6077	27
H(15)	6749	5445	4843	27
H(16)	5309	4277	3931	23
H(22)	3811	6107	2611	20
H(23)	5322	6660	1485	23
H(24)	6201	5091	522	26
H(25)	5609	2945	727	25
H(26)	4067	2391	1832	20

VII. Computational Details

All structures were optimized and characterized in dichloromethane with the SMD[4] solvent model (SCRF = SMD) at M06-2X[5]/6-31G(d) level. Harmonic frequency analysis calculations at the same level were performed to verify the optimized transition states (TSs, having unique one imaginary frequency). The energies were further improved by M06-2X/6-311+G(d,p) //M06-2X/6-31G(d) single-point calculations with solvent effects accounted by the SMD solvent model, using the experimental solvent (dichloromethane). Intrinsic reaction coordinate (IRC) calculations for cycloaddition transition states were carried out at the M06-2X/6-31G(d) level to verify the transition state correctly connecting with its nearby minima.[6] All DFT calculations were carried out using Gaussian 09 program.[7] Computed structures are illustrated using the CYLview.[8]

---

4. A. V. Marenich, C. J. Cramer, D. G. Truhlar, *J. Phys. Chem. B* 2009, **113**, 6378–6396.
5. (a) Y. Zhao, D. G. Truhlar, *Theor. Chem. Acc.* 2008, **120**, 215–241. (b) Y. Zhao, D. G. Truhlar, *Acc. Chem. Res.* 2008, **41**, 157–167. (c) Y. Zhao, D. G. Truhlar, *J. Chem. Theory Comput.* 2009, **5**, 324–333.
6 K. Fukui, Acc. *Chem. Res.* 1981, **14**, 363.
7 M. J. Frisch, G. W. Trucks, H. B. Schlegel, G. E. Scuseria, M. A. Robb, J. R. Cheeseman, G. Scalmani, V. Barone, B. Mennucci, G. A. Petersson, H. Nakatsuji, M. Caricato, X. Li, H. P. Hratchian, A. F. Izmaylov, J. Bloino, G. Zheng, J. L. Sonnenberg, M. Hada, M. Ehara, K. Toyota, R. Fukuda, J. Hasegawa, , M. Ishida, T.Nakajima, Y. Honda, O. Kitao, H. Nakai, T. Vreven, J. A. Montgomery, Jr.; J. E. Peralta, F. Ogliaro, M. Bearpark, J. J. Heyd, E. Brothers, K. N. Kudin, V. N. Staroverov, T. Keith, R. Kobayashi, J. Normand, K. Raghavachari, A. Rendell, J. C. Burant, S. S. Iyengar, J. Tomasi, M. Cossi, N. Rega, J. M. Millam, M. Klene, J. E. Knox, J. B. Cross, V. Bakken, C. Adamo, J. Jaramillo, R. Gomperts, R. E. Stratmann, O. Yazyev, A. J. Austin, R. Cammi, C. Pomelli, J. W. Ochterski, R. L. Martin, K. Morokuma, V. G. Zakrzewski, G. A. Voth, P. Salvador, J. J. Dannenberg, S. Dapprich, A. D. Daniels, O. Farkas, J. B. Foresman, J. V. Ortiz, J. Cioslowski, and D. J. Fox, Gaussian 09, Rev. A.01; Gaussian, Inc.: Wallingford, CT, 2010.
8 C. Y. Legault, *CYLView, version 1.0 b*; Université de Sherbrooke: Sherbrooke,

Cartesian Coordinates in Å, SCF Energies and Free Energies (in a.u.) at 298.15 K and 1 atm for the Optimized Structures [BSI= 6-31G(d), BSII = 6-311+G(d,p)

TSA-major

M06-2X/BSI SCF energy in dichloromethane: -4205.974053 a.u.

M06-2X/BSII SCF energy in dichloromethane: -4207.035873 a.u.

M06-2X/BSII free energy in dichloromethane: -4205.885975 a.u.

P	-0.54789000	1.04013600	0.90925600
O	-1.82968000	1.48518000	-0.02031300
O	0.26954000	2.45399500	0.99916900
O	0.26497800	0.14163000	-0.02141100
O	-0.93916100	0.63247900	2.27178500
C	0.14044100	3.43686900	0.04812300
C	-1.04449700	4.14058100	-0.05484500
C	-1.14090700	5.18719700	-1.03291900
C	0.01412400	5.52661300	-1.79367800
C	1.20396300	4.77500700	-1.63092500
C	1.27887400	3.72129500	-0.75216900
H	-3.25679200	5.61404800	-0.78027700
C	-2.34993400	5.88599100	-1.30817300
C	-0.05313000	6.57976300	-2.74549700
H	2.06494700	5.02030900	-2.24808900
C	-1.22531100	7.25623400	-2.96238400
C	-2.38784200	6.89202100	-2.24151700
H	0.84463300	6.82669600	-3.30612900
H	-1.27146000	8.05731900	-3.69366700
H	-3.32315300	7.40843200	-2.43524800

Quebec, Canada, 2009. http://www.cylview.org.

C	-2.23562600	3.71006800	0.72853500
C	-3.01035300	4.59552900	1.54883800
C	-2.64885700	2.39835400	0.61785400
C	-2.58784900	5.91718100	1.85539300
C	-4.23198300	4.12554100	2.10965600
C	-3.90100800	1.93378200	1.10413500
C	-3.34965300	6.72980100	2.65872000
H	-1.64335400	6.27618100	1.45858700
C	-5.00547000	4.99516800	2.92343700
C	-4.67288100	2.80408400	1.83073700
C	-4.57743200	6.27000900	3.19263600
H	-3.00651300	7.73341400	2.89105500
H	-5.94084900	4.62327600	3.33319800
H	-5.64385200	2.48405300	2.20116600
H	-5.17240100	6.92590500	3.82051500
C	-3.66275100	-1.52422000	3.65070800
C	-3.75401500	-0.43066300	2.84165700
C	-4.33437300	-0.52388100	1.53392900
C	-4.83015800	-1.79904700	1.08375300
C	-4.69271600	-2.92402900	1.96328400
C	-4.13519600	-2.79145200	3.20160300
C	-4.40270000	0.59127900	0.68367400
C	-5.40858900	-1.92085100	-0.19576100
C	-5.49320700	-0.79351300	-1.03885000
C	-4.96950600	0.47147800	-0.60052600
C	-5.04452900	1.58991300	-1.49598700
H	-4.64816100	2.54762900	-1.17510600
C	-5.60590300	1.47242100	-2.73335300
C	-6.14938600	0.22694200	-3.15828500

C	-6.09972900	-0.86189900	-2.33766700
H	-3.21238800	-1.43663200	4.63458800
H	-3.35546000	0.52424400	3.16354400
H	-5.03669200	-3.89530800	1.62517000
H	-4.04225900	-3.65813600	3.84990700
H	-5.64762900	2.33207800	-3.39510200
H	-6.61313100	0.14827000	-4.13687900
H	-6.52959400	-1.80108000	-2.66687300
C	2.10202200	0.99041400	-3.89642900
C	1.82507100	1.80000100	-2.83422300
C	2.79809200	2.04534100	-1.80860900
C	4.07948700	1.40142600	-1.90826300
C	4.32868300	0.56529700	-3.04887200
C	3.37978800	0.36923800	-4.00929300
C	2.51494100	2.88375800	-0.71411900
C	5.04088600	1.58492900	-0.89263300
C	4.71588100	2.34585300	0.25048600
C	3.43710300	2.99972800	0.33974400
C	3.14597200	3.78843500	1.50043100
H	2.18143100	4.28137500	1.56896000
C	4.05008400	3.91812300	2.51260400
C	5.32495100	3.28934400	2.41803300
C	5.64758100	2.54113400	1.32419700
H	1.34546700	0.81053900	-4.65425400
H	0.84502100	2.25567800	-2.74066200
H	5.28766500	0.06541200	-3.13171900
H	3.59115600	-0.27467800	-4.85798100
H	3.80523900	4.50914900	3.38963800
H	6.04663400	3.41644700	3.21912100

H	6.62808200	2.08361300	1.25742700
C	-5.91685200	-3.24029300	-0.67404500
C	-7.02069400	-3.85028400	-0.06644800
C	-5.30536100	-3.88110200	-1.75846700
C	-7.50191400	-5.07198900	-0.53215500
H	-7.50536600	-3.35939400	0.77358100
C	-5.79116000	-5.09904500	-2.22997600
H	-4.44365400	-3.41804300	-2.23337100
C	-6.88998400	-5.69860600	-1.61685900
H	-8.36062300	-5.53117800	-0.05149400
H	-5.30729700	-5.58115100	-3.07471700
H	-7.26896800	-6.64776100	-1.98357900
C	6.39972500	0.98642100	-1.04943900
C	7.23787700	1.40861600	-2.08921500
C	6.86103400	-0.00839100	-0.17982300
C	8.49900500	0.84310700	-2.26108600
H	6.89212900	2.18362200	-2.76830300
C	8.12180300	-0.57684100	-0.35166200
H	6.22650100	-0.32348200	0.64288600
C	8.94335800	-0.15483500	-1.39496300
H	9.13556100	1.18340600	-3.07250200
H	8.46234200	-1.34772100	0.33369500
H	9.92576700	-0.59730100	-1.52983500
C	1.38689600	-4.57938700	0.05500600
C	2.12516200	-4.67302400	1.19530100
C	3.54641700	-4.55181000	1.15060800
C	4.13387800	-4.15761300	-0.07364000
H	3.94738100	-5.13783500	3.19169200
H	1.63841600	-4.94190600	2.12537900

C	4.38880900	-4.83961300	2.24572000
C	5.53437000	-4.03567000	-0.20514200
C	6.33449200	-4.34009800	0.87317600
C	5.75583100	-4.74183100	2.09904600
H	5.95802800	-3.70928100	-1.15102700
H	7.41391200	-4.27071800	0.78737700
H	6.40218000	-4.97067900	2.94069300
O	1.99750400	-4.26035300	-1.11624000
C	-0.03074600	-4.93066000	-0.09751800
C	-0.87546000	-4.93771000	1.01956000
C	-0.53444500	-5.31208400	-1.34541900
C	-2.19836000	-5.34082700	0.88956500
H	-0.50814900	-4.59250400	1.97969000
C	-1.86209300	-5.71346400	-1.46979500
H	0.11315500	-5.30453100	-2.21573900
C	-2.69578300	-5.73555300	-0.35291700
H	-2.84524400	-5.34056300	1.76072100
H	-2.24192000	-6.01444800	-2.44178000
H	-3.72983000	-6.05324900	-0.44794600
C	2.82927500	-1.24349900	1.60885400
C	2.05363200	-0.76071700	2.68020000
C	2.62930900	-0.50260000	3.92052400
C	3.98464300	-0.74611600	4.14005300
C	4.76308100	-1.25262400	3.10121600
C	4.19140200	-1.49091600	1.85549800
H	0.99151700	-0.59419200	2.54080800
H	2.01038400	-0.11235600	4.72348500
H	4.42889300	-0.54812500	5.11131400
H	5.81808100	-1.46274300	3.25759900

H	4.81971900	-1.88262700	1.06315000
C	2.22214900	-1.45502100	0.27763100
C	3.01690600	-1.57383700	-0.82947500
H	4.09403400	-1.40604700	-0.81283100
H	2.53733700	-1.54702600	-1.80452500
B	0.59818400	-1.42006700	0.03949800
O	-0.09633000	-2.03674600	1.13024500
H	-1.02446800	-2.18944700	0.90019200
O	0.30550700	-1.93999200	-1.27212000
C	-0.76949700	-1.45986500	-2.04329400
H	-0.65624600	-0.37539700	-2.19583700
C	-0.70211800	-2.15174100	-3.39832200
H	-0.88633900	-3.22634600	-3.28322400
H	-1.45002500	-1.74432000	-4.08678900
H	0.28985600	-2.01927700	-3.84352500
C	-2.12271800	-1.70508800	-1.37766200
H	-2.22433200	-2.76624100	-1.10986300
H	-2.24574300	-1.09817000	-0.47288100
H	-2.93873300	-1.43588600	-2.05850400
C	3.24940500	-3.84156400	-1.13392600
H	3.60136000	-3.62904000	-2.13754300

TSA-minor

M06-2X/BSI SCF energy in dichloromethane: -4205.97731 a.u.

M06-2X/BSII SCF energy in dichloromethane: -4207.038216a.u.

M06-2X/BSII free energy in dichloromethane: -4205.884514 a.u.

P	0.00586600	1.10938700	-0.81365800
O	1.44404500	1.58281000	-0.19034100

O	-0.77315000	2.52990400	-0.95821000
O	-0.68327500	0.34798600	0.32949300
O	0.09010200	0.50059000	-2.15953300
C	-0.61713900	3.50736400	-0.00107300
C	0.56595300	4.21177700	0.04745300
C	0.72056500	5.21374800	1.06320600
C	-0.38957900	5.51958600	1.90203700
C	-1.59974200	4.79113000	1.76329600
C	-1.72381400	3.77528400	0.84807900
H	2.81681100	5.64520600	0.69699000
C	1.94679300	5.89607100	1.29428700
C	-0.25768800	6.52407600	2.89848200
H	-2.43306300	5.02929400	2.42007800
C	0.93111200	7.18289400	3.07933500
C	2.04784300	6.85131200	2.27520700
H	-1.11984500	6.75060200	3.52030800
H	1.02550500	7.94582200	3.84572900
H	2.99598200	7.35419200	2.43951400
C	1.67674700	3.85190900	-0.87636800
C	2.31797600	4.81309200	-1.72529500
C	2.13367500	2.54935800	-0.89816500
C	1.82615000	6.13706400	-1.88377700
C	3.47013800	4.42360800	-2.46493300
C	3.34108600	2.16853100	-1.54914900
C	2.45944500	7.02646400	-2.71612600
H	0.93008200	6.43638800	-1.34981300
C	4.10921600	5.36931300	-3.31002500
C	3.98320800	3.10736500	-2.31719900
C	3.61866200	6.64454000	-3.43290500

H	2.06361500	8.03083700	-2.83112800
H	4.99270100	5.05848700	-3.86136100
H	4.91857300	2.84969600	-2.80848200
H	4.10987500	7.36045000	-4.08460500
C	2.49190100	-1.45345600	-3.72297500
C	2.80995300	-0.30378300	-3.06197800
C	3.69233000	-0.31707900	-1.93404000
C	4.27608300	-1.56818900	-1.52479400
C	3.88570500	-2.75560400	-2.22950100
C	3.02659100	-2.70053700	-3.28899300
C	3.98106500	0.85750600	-1.22073700
C	5.18427300	-1.60235300	-0.44701800
C	5.50961300	-0.41262300	0.23566400
C	4.88287100	0.82648500	-0.13960900
C	5.18633700	2.00279800	0.62538600
H	4.69943800	2.93729900	0.36812500
C	6.06741100	1.96618000	1.66596000
C	6.72599200	0.75033500	2.00569200
C	6.45677600	-0.39388600	1.31388700
H	1.81566900	-1.42384300	-4.57193700
H	2.37676000	0.64114300	-3.36914200
H	4.28184800	-3.71174600	-1.90554500
H	2.74803000	-3.61303000	-3.80859100
H	6.27855400	2.86852800	2.23169900
H	7.44677200	0.73798900	2.81780600
H	6.97114300	-1.31196000	1.57442600
C	-2.67914100	0.85385700	3.81010900
C	-2.37411500	1.76843200	2.84573300
C	-3.24936200	1.99418600	1.73118000

C	-4.44248000	1.19642800	1.62977300
C	-4.71477600	0.23500500	2.65964400
C	-3.87176900	0.07863700	3.71980900
C	-2.96061400	2.94922700	0.73926600
C	-5.30079100	1.35644800	0.52815900
C	-5.01235900	2.31596700	-0.46040800
C	-3.82899200	3.12335300	-0.35372100
C	-3.57189600	4.09626300	-1.37511100
H	-2.68251800	4.71418100	-1.29859200
C	-4.42419000	4.25851700	-2.42786200
C	-5.60166600	3.46268800	-2.52988300
C	-5.88464900	2.52687900	-1.57916400
H	-2.00859300	0.70763200	4.65136900
H	-1.45522400	2.34104600	2.91359900
H	-5.61058600	-0.37221100	2.58153200
H	-4.09588400	-0.65053000	4.49315300
H	-4.21190300	5.00068500	-3.19139700
H	-6.27705500	3.60875000	-3.36739000
H	-6.78682900	1.92943300	-1.65608300
C	5.77796200	-2.89751600	-0.00348200
C	6.63379500	-3.63176300	-0.83230100
C	5.47948700	-3.40001900	1.26925200
C	7.16814300	-4.84502400	-0.40209200
H	6.87936200	-3.24823500	-1.81936600
C	6.01005800	-4.61376800	1.69891700
H	4.82248700	-2.82635200	1.91945800
C	6.85552700	-5.34065000	0.86217200
H	7.83122500	-5.40323200	-1.05640900
H	5.76421200	-4.99139700	2.68755500

H	7.26979700	-6.28731900	1.19577400
C	-6.49982800	0.48121600	0.37461000
C	-7.66960800	0.70909500	1.10566900
C	-6.45697200	-0.58617700	-0.52826200
C	-8.77914300	-0.11692000	0.93505400
H	-7.70858700	1.53754100	1.80826200
C	-7.56526200	-1.41248200	-0.69700500
H	-5.54760600	-0.76232700	-1.09806700
C	-8.72982400	-1.17842700	0.03303200
H	-9.68435000	0.07280700	1.50449300
H	-7.51782900	-2.23726300	-1.40278300
H	-9.59597000	-1.81978000	-0.10120500
C	0.76037600	-4.32477500	1.47700700
C	0.33346900	-4.47593600	0.11980600
C	-0.94535600	-4.81897500	-0.16287700
H	1.05746600	-4.39777700	-0.68175900
C	-2.70316400	-2.16625100	-0.39088100
C	-2.44011700	-1.53832300	-1.62238000
C	-3.21605200	-1.79480700	-2.74863500
C	-4.26393900	-2.71245700	-2.69007500
C	-4.54896300	-3.34140700	-1.47919200
C	-3.79241400	-3.06054400	-0.34701100
H	-1.62345700	-0.83273000	-1.71127100
H	-2.98617100	-1.28638400	-3.68064200
H	-4.85226600	-2.93423800	-3.57591500
H	-5.36114300	-4.06062200	-1.41502100
H	-4.04027900	-3.57518700	0.57504800
C	-1.83286200	-1.92648100	0.78244700
C	-2.26843800	-2.35559600	2.01399800

H	-3.30028600	-2.65218400	2.19142000
H	-1.69094400	-2.08091600	2.89054000
B	-0.34841400	-1.16482900	0.71175900
O	0.58941200	-1.67447400	-0.26293500
H	0.30400000	-1.51572600	-1.17523900
O	0.18665800	-1.18739000	2.03855700
C	1.16492800	-0.26025400	2.45713700
H	0.87796800	0.74499500	2.11516500
C	1.17360500	-0.26794900	3.98042900
H	1.44218300	-1.26574600	4.34785900
H	1.89702300	0.45351400	4.37509100
H	0.18210400	-0.01498800	4.37004700
C	2.55061000	-0.57622700	1.90112700
H	2.90101000	-1.53961700	2.29080200
H	2.52221100	-0.63007900	0.81004800
H	3.26661100	0.19973200	2.20030400
C	-1.58271400	-4.47033600	2.05554100
H	-2.37692300	-4.67762900	2.76485200
C	-1.48433000	-5.15177600	-1.48737600
C	-0.95341400	-4.53024600	-2.62303500
C	-2.52369200	-6.07985200	-1.62453100
C	-1.44592900	-4.84835400	-3.88451000
H	-0.18130900	-3.77373700	-2.51156600
C	-3.00952500	-6.39549100	-2.88830000
H	-2.94511500	-6.55384700	-0.74309300
C	-2.47222900	-5.78194200	-4.01996500
H	-1.03680400	-4.35437300	-4.76051000
H	-3.80970500	-7.12194200	-2.99090200
H	-2.85635400	-6.02776500	-5.00540400

O	-1.86136600	-4.94013700	0.84680700
C	-0.22782400	-4.34478800	2.48055800
C	0.11617600	-4.23495700	3.84011600
H	-0.66444200	-4.24042700	4.59603200
C	1.44751800	-4.14739600	4.18785400
C	2.44211700	-4.12846100	3.18741400
H	1.73442300	-4.08504900	5.23258500
C	2.10927100	-4.20241100	1.84946900
H	2.87333600	-4.16752800	1.07769100
H	3.48601600	-4.04593100	3.47802700

TSB-major

M06-2X/BSI SCF energy in dichloromethane: -4205.982433 a.u.

M06-2X/BSII SCF energy in dichloromethane: -4207.04699 a.u.

M06-2X/BSII free energy in dichloromethane: -4205.895196 a.u.

P	0.08882200	1.21610600	0.14559900
O	-0.86559200	2.40304400	-0.51464200
O	1.38613400	2.14216700	0.59700200
O	0.41202600	0.29989800	-0.97976200
O	-0.49051500	0.73938900	1.44261900
C	1.81819100	3.14472800	-0.22675700
C	1.10248400	4.32686300	-0.30750700
C	1.57680000	5.35846600	-1.18416000
C	2.81928000	5.17692900	-1.85713200
C	3.53061800	3.96070700	-1.69935300
C	3.03997100	2.94004400	-0.92399000
H	-0.12659500	6.68883800	-0.97993900
C	0.84409400	6.55051500	-1.44380000

C	3.31402600	6.20063100	-2.70887900
H	4.47074700	3.83051900	-2.23032100
C	2.59724600	7.35065800	-2.91503200
C	1.34095000	7.51690800	-2.28315000
H	4.26988200	6.04390600	-3.20229500
H	2.97966100	8.12608600	-3.57153100
H	0.76155100	8.41580000	-2.47166600
C	-0.17031400	4.45287200	0.45596300
C	-0.42999100	5.52039300	1.37648700
C	-1.13028000	3.47186500	0.29851600
C	0.54634400	6.49790300	1.71209500
C	-1.70183200	5.58896300	2.01460100
C	-2.40876900	3.53085100	0.92044600
C	0.26186900	7.50209000	2.60427200
H	1.53275700	6.43551300	1.26434700
C	-1.96952900	6.64649600	2.92495900
C	-2.67772800	4.59098700	1.75014600
C	-1.01369700	7.58724200	3.21199300
H	1.02469300	8.23455300	2.85088700
H	-2.94831900	6.68828800	3.39612300
H	-3.64911900	4.66764700	2.23347500
H	-1.22572200	8.38939800	3.91213900
C	-3.31664100	0.48745700	3.78048800
C	-3.04454900	1.45385700	2.85954200
C	-3.72458500	1.49368400	1.59738000
C	-4.71566400	0.48722200	1.31769400
C	-4.94865600	-0.52664500	2.30760100
C	-4.27940200	-0.52436000	3.49594700
C	-3.42119800	2.47053700	0.63311600

C	-5.40472600	0.49944600	0.08839500
C	-5.09598400	1.47792900	-0.87891000
C	-4.07954200	2.45987900	-0.61140200
C	-3.77423800	3.42333500	-1.62833200
H	-3.00407700	4.16300700	-1.43465100
C	-4.43071500	3.42627700	-2.82393100
C	-5.46258200	2.47763800	-3.07632300
C	-5.78730600	1.54516400	-2.13493100
H	-2.78283500	0.47126400	4.72586300
H	-2.27865700	2.19451600	3.05649100
H	-5.66493600	-1.31254800	2.09402300
H	-4.46951100	-1.30372200	4.22808500
H	-4.17885600	4.16111800	-3.58255500
H	-5.99850400	2.50561800	-4.02031000
H	-6.58663900	0.83960400	-2.33170000
C	2.56012000	-0.05806600	-3.91208000
C	2.70061400	0.87381200	-2.92758300
C	3.63719400	0.69090400	-1.85759200
C	4.43502300	-0.50576800	-1.83813400
C	4.24243000	-1.46692200	-2.88718100
C	3.34323400	-1.24863500	-3.89001600
C	3.77786400	1.64413800	-0.83334800
C	5.36003300	-0.71776400	-0.79644900
C	5.48993400	0.23339000	0.23548400
C	4.67233200	1.41639300	0.22713700
C	4.80574900	2.34975400	1.30673800
H	4.17673700	3.23439900	1.31161600
C	5.70137100	2.14306500	2.31482700
C	6.54414500	0.99372400	2.30150200

C	6.44192000	0.07445200	1.29769400
H	1.83555300	0.09398400	-4.70592100
H	2.08039500	1.76287800	-2.92652100
H	4.82468100	-2.38201600	-2.87363600
H	3.21404800	-1.98995700	-4.67309500
H	5.78386900	2.86025600	3.12566400
H	7.27428100	0.85430600	3.09293700
H	7.09276300	-0.79314900	1.29250200
C	-6.46775800	-0.51273000	-0.18301600
C	-7.63883900	-0.53273200	0.58500600
C	-6.32060600	-1.45671700	-1.20628300
C	-8.63086400	-1.48023600	0.34536900
H	-7.76700100	0.20130400	1.37616900
C	-7.31399000	-2.40335500	-1.45016600
H	-5.41948400	-1.44426300	-1.81403700
C	-8.47000200	-2.42086900	-0.67128700
H	-9.53196400	-1.48137200	0.95125100
H	-7.17940900	-3.12960100	-2.24685800
H	-9.24338600	-3.15998300	-0.85725500
C	6.18596000	-1.95966800	-0.77297400
C	7.18899200	-2.16727600	-1.72661900
C	5.96803500	-2.93921300	0.20371900
C	7.95844400	-3.32819300	-1.70601500
H	7.36324400	-1.41114300	-2.48749900
C	6.73868100	-4.10069700	0.22482000
H	5.18944500	-2.78443300	0.94904700
C	7.73560300	-4.29801100	-0.72995800
H	8.73423600	-3.47268200	-2.45179700
H	6.56259300	-4.85163300	0.99021600

H	8.33663900	-5.20198900	-0.71248100
C	-0.94085500	-5.18571000	0.12487200
C	-0.53461600	-5.10748600	1.41693400
C	0.85894300	-5.16517400	1.74781900
C	1.78360700	-5.12661100	0.68119200
H	0.63386900	-5.28397300	3.89018700
H	-1.28355400	-5.08920300	2.20195400
C	1.34076300	-5.25531600	3.06641500
C	3.17033000	-5.14405100	0.92544100
C	3.62018900	-5.23093200	2.22612900
C	2.70214300	-5.28804100	3.29514700
H	3.86577000	-5.09258500	0.09166100
H	4.68552600	-5.25289900	2.43315000
H	3.07442200	-5.35048200	4.31276400
O	-0.02497600	-5.25561200	-0.88062100
C	-2.33333300	-5.30769500	-0.33230900
C	-3.32112400	-4.49763200	0.23581700
C	-2.67333700	-6.25239200	-1.30690700
C	-4.64654200	-4.64061000	-0.16478100
H	-3.04412200	-3.72799600	0.95292800
C	-4.00217500	-6.39768400	-1.69335500
H	-1.90283100	-6.87805300	-1.74795400
C	-4.98915600	-5.59286800	-1.12375400
H	-5.40881900	-3.99812000	0.26593500
H	-4.26679900	-7.13837500	-2.44173300
H	-6.02482900	-5.70496300	-1.43068400
C	1.21624900	-1.94929200	1.24971600
C	0.50002900	-2.08850600	2.45119800
C	1.06679800	-1.72143300	3.66552100

C	2.35086900	-1.18045400	3.70130200
C	3.06585600	-1.00693000	2.51559200
C	2.50785200	-1.39684500	1.30315100
H	-0.50945400	-2.48899000	2.42506700
H	0.50185100	-1.84589700	4.58473600
H	2.78682800	-0.87473900	4.64796900
H	4.05377000	-0.55349700	2.53460900
H	3.04836600	-1.21675800	0.37782300
C	0.61353500	-2.30836900	-0.04121000
C	1.39186800	-2.83238700	-1.04023000
H	2.47801300	-2.87193100	-0.95105300
H	0.97918500	-2.92777500	-2.04146300
B	-0.92674100	-2.00944400	-0.34200300
O	-1.76336200	-1.38239500	0.52314400
H	-1.31285700	-0.69603500	1.08308900
O	-1.40122700	-2.49900500	-1.52691500
C	-2.68307400	-2.04281600	-1.98054200
H	-3.38886400	-2.10440200	-1.14332300
C	-2.58101200	-0.59784300	-2.44899300
H	-1.82963200	-0.51628100	-3.24264800
H	-3.54356900	-0.25344100	-2.84562000
H	-2.28631700	0.06257700	-1.62825500
C	-3.12703600	-2.97438200	-3.09256000
H	-2.44086700	-2.89980700	-3.94414000
H	-3.13751300	-4.01117900	-2.74609700
H	-4.13287500	-2.71029000	-3.43704400
C	1.25367100	-4.99075400	-0.63557500
H	1.87397100	-5.14205200	-1.51324100

TSB-minor

M06-2X/BSI SCF energy in dichloromethane: -4205.978583 a.u.

M06-2X/BSII SCF energy in dichloromethane: -4207.043656 a.u.

M06-2X/BSII free energy in dichloromethane: -4205.893742 a.u.

P	1.37472100	-0.29185900	-0.01865000
O	2.79805700	0.27766700	-0.65333800
O	1.93149300	-1.74117900	0.57145400
O	0.47557700	-0.48285100	-1.18535400
O	1.00887100	0.49828700	1.19930000
C	2.73920700	-2.48385900	-0.24537100
C	4.07445700	-2.14105000	-0.38295000
C	4.89428700	-2.92055300	-1.26453600
C	4.33765700	-4.07330000	-1.88978100
C	2.97283200	-4.39684000	-1.67825900
C	2.16321600	-3.61204800	-0.89480900
H	6.66605300	-1.67193500	-1.14993600
C	6.23779200	-2.57152100	-1.57871400
C	5.14538600	-4.86377200	-2.75146100
H	2.55854900	-5.27065600	-2.17620100
C	6.44548700	-4.51545600	-3.01128700
C	6.98959900	-3.34661500	-2.42642300
H	4.70279100	-5.74497200	-3.20889900
H	7.05503000	-5.12222100	-3.67371700
H	8.01075300	-3.05765300	-2.65665300
C	4.57953900	-0.93156400	0.32806000
C	5.70786000	-0.95124000	1.21214200
C	3.88909000	0.25545700	0.16842800
C	6.40586200	-2.14478700	1.54508500

C	6.13469500	0.26814800	1.81350800
C	4.27041500	1.47107500	0.80632300
C	7.48653600	-2.11893800	2.39158300
H	6.06497100	-3.08852300	1.13245900
C	7.26574200	0.26493900	2.67375400
C	5.40019300	1.46215000	1.58655800
C	7.93471300	-0.89924600	2.95315700
H	8.00124200	-3.04342000	2.63571800
H	7.58288700	1.20739700	3.11317200
H	5.72348900	2.37525400	2.08143600
H	8.79617300	-0.89254700	3.61373800
C	1.91035900	2.85970200	4.07603400
C	2.64975500	2.42482600	3.01706400
C	2.63015300	3.11184500	1.75757000
C	1.75971300	4.24780900	1.61736400
C	1.01422100	4.68243500	2.76489100
C	1.09002700	4.01819200	3.95328600
C	3.40303600	2.67854600	0.66266100
C	1.64609700	4.90026900	0.37519900
C	2.41909800	4.46651000	-0.71939300
C	3.31623300	3.35263500	-0.56884800
C	4.10199200	2.95125700	-1.69889800
H	4.79603800	2.12462200	-1.58253000
C	3.99596700	3.58779200	-2.90023300
C	3.08171000	4.66908700	-3.06025000
C	2.31986300	5.08909200	-2.00920600
H	1.93764200	2.31742000	5.01631400
H	3.25157800	1.52833300	3.11098200
H	0.38537900	5.56180400	2.67637500

H	0.51891800	4.36555600	4.80928500
H	4.60224300	3.26845300	-3.74238300
H	2.98894500	5.15518800	-4.02710700
H	1.61786700	5.90556500	-2.14280500
C	-0.58995900	-2.75262200	-4.03929500
C	0.26855400	-2.99951200	-3.01007700
C	-0.17311200	-3.64890800	-1.80934500
C	-1.56398000	-3.99818000	-1.69506300
C	-2.43201800	-3.72542200	-2.80692400
C	-1.96110200	-3.13139100	-3.94068900
C	0.70833900	-3.92166700	-0.74655600
C	-2.04699900	-4.58994600	-0.51400600
C	-1.16425100	-4.87274600	0.54533500
C	0.22690400	-4.52606500	0.43024600
C	1.09267700	-4.80826900	1.53716500
H	2.14306800	-4.54567600	1.45848600
C	0.62065200	-5.39870900	2.67314200
C	-0.75641500	-5.74831500	2.78445500
C	-1.61861900	-5.48745900	1.75921800
H	-0.23365700	-2.25679400	-4.93704900
H	1.30498700	-2.69075900	-3.08186600
H	-3.47643100	-4.01248800	-2.73657400
H	-2.63122400	-2.94149300	-4.77427500
H	1.29427300	-5.60357500	3.49958100
H	-1.11890300	-6.21685700	3.69455600
H	-2.66858800	-5.74603700	1.85156800
C	0.67636400	6.02341800	0.21150000
C	1.11215500	7.33851600	0.01708700
C	-0.70011700	5.76455800	0.24877400

C	0.19219200	8.37353500	-0.14270700
H	2.17835100	7.54864700	-0.00930800
C	-1.61870100	6.79918600	0.08593500
H	-1.03379800	4.74090600	0.40324000
C	-1.17523200	8.10631400	-0.11089900
H	0.54551300	9.38987800	-0.29031100
H	-2.68330100	6.58306200	0.11260500
H	-1.89204400	8.91226100	-0.23679300
C	-3.51204400	-4.83710200	-0.36277100
C	-4.07526300	-6.10333300	-0.54186100
C	-4.34986200	-3.76068700	-0.04779300
C	-5.45158200	-6.28678800	-0.40869300
H	-3.43250600	-6.94428500	-0.78838200
C	-5.72387200	-3.94185400	0.08535600
H	-3.90757900	-2.77680800	0.09799800
C	-6.27799200	-5.20890700	-0.09638400
H	-5.87899400	-7.27491200	-0.55047600
H	-6.35765000	-3.09476000	0.33436500
H	-7.34887900	-5.35606300	0.00634200
C	-5.12875200	0.82196600	0.70877600
C	-5.04154600	2.07962700	0.19318800
C	-5.05026700	2.28905300	-1.22010200
C	-4.93039300	1.15272500	-2.05077300
H	-5.27419900	4.43736200	-1.18861600
H	-5.04395800	2.93408600	0.86024800
C	-5.18544900	3.55658600	-1.81754100
C	-4.92621200	1.27235300	-3.45541800
C	-5.08103500	2.51892300	-4.01727100
C	-5.21265700	3.65903800	-3.19283200

H	-4.81548100	0.38240900	-4.06852000
H	-5.09754500	2.63355400	-5.09583400
H	-5.32539800	4.63502100	-3.65495900
O	-5.09628700	-0.24978000	-0.13202400
C	-5.29508000	0.43889600	2.11556400
C	-5.63168400	1.39976800	3.07811400
C	-5.08855600	-0.88886300	2.51107600
C	-5.74320000	1.03752800	4.41504100
H	-5.81924300	2.42887900	2.78891700
C	-5.21008000	-1.24609000	3.84860800
H	-4.80676800	-1.63917600	1.78096400
C	-5.53223800	-0.28486100	4.80463200
H	-6.00229800	1.78952700	5.15367100
H	-5.03585300	-2.27619700	4.14372900
H	-5.61946500	-0.56398800	5.85019300
C	-2.01554100	-0.10097700	1.05503300
C	-2.12474700	0.75512300	2.16281200
C	-1.94537100	0.27799800	3.45464400
C	-1.63145700	-1.06590500	3.66282800
C	-1.49795100	-1.92497200	2.57305100
C	-1.68570700	-1.44589200	1.27982200
H	-2.35788000	1.80516100	2.00018300
H	-2.04260700	0.95402100	4.29930100
H	-1.47639200	-1.43865300	4.67121900
H	-1.21560800	-2.96300500	2.72663700
H	-1.50192700	-2.09809600	0.42948900
C	-2.12464800	0.42714700	-0.31536200
C	-2.53392400	-0.36466700	-1.34801700
H	-2.70269400	-1.43205800	-1.20772100

H	-2.40293300	-0.02714200	-2.37270800
B	-1.46613100	1.83105600	-0.67312400
O	-0.49467400	2.39196400	0.08695900
H	-0.02617400	1.75447900	0.69001500
O	-1.88299800	2.44286100	-1.82518300
C	-0.97288200	3.35021900	-2.46827800
H	-0.40717600	3.89081900	-1.70160300
C	-0.00840800	2.52957100	-3.31106000
H	-0.56737400	1.92820600	-4.03827200
H	0.68461100	3.17961100	-3.85427600
H	0.57240500	1.85210600	-2.67451000
C	-1.77681800	4.33995000	-3.28847400
H	-2.35081500	3.82337000	-4.06598400
H	-2.47283800	4.89689300	-2.65307800
H	-1.10316800	5.05686600	-3.76989600
C	-4.78423700	-0.10139600	-1.40947600
H	-4.82688700	-1.03796100	-1.95657100

TSC-major

M06-2X/BSI SCF energy in dichloromethane: -4205.987003 a.u.

M06-2X/BSII SCF energy in dichloromethane: -4207.05046 a.u.

M06-2X/BSII free energy in dichloromethane: -4205.900742 a.u.

P	-0.55880900	1.07624500	-0.57469700
O	-1.79603100	2.14796500	-0.78827000
O	0.40912700	1.95050400	0.45257200
O	0.10999400	0.89530900	-1.88964800
O	-1.04087000	-0.07152800	0.25808300
C	0.67096300	3.25887100	0.12988100

C	-0.31624300	4.20771900	0.32447300
C	-0.06922100	5.56182300	-0.07166900
C	1.22382800	5.91319000	-0.54923800
C	2.22524100	4.91572100	-0.64228700
C	1.97556000	3.60083000	-0.32283600
H	-2.07262300	6.31342300	0.29354100
C	-1.07053000	6.57175800	-0.03220600
C	1.49089100	7.25597000	-0.93086100
H	3.21746100	5.20414200	-0.98142200
C	0.51036000	8.21146700	-0.86216600
C	-0.78703000	7.85821600	-0.41771400
H	2.48864400	7.50519100	-1.28285700
H	0.71984300	9.23523100	-1.15650800
H	-1.56696600	8.61326300	-0.38593600
C	-1.61031200	3.78437100	0.92184300
C	-2.11815700	4.37817100	2.12404100
C	-2.31279800	2.74906400	0.33508700
C	-1.39817600	5.35056300	2.87301900
C	-3.38331700	3.95327100	2.61684300
C	-3.57718300	2.31351500	0.81903700
C	-1.92688200	5.88899100	4.02018200
H	-0.41314500	5.65910200	2.53962800
C	-3.91025900	4.53703600	3.80027500
C	-4.09147300	2.93550700	1.93246300
C	-3.20217900	5.48871400	4.48719900
H	-1.35811900	6.62572400	4.57951500
H	-4.88255600	4.20298400	4.15273300
H	-5.06077400	2.62642300	2.31651700
H	-3.60825700	5.92692400	5.39359300

C	-4.30520900	-1.38756900	2.81069700
C	-4.00859600	-0.25113200	2.11740800
C	-4.66027200	0.05748200	0.87675700
C	-5.60985400	-0.88628900	0.35318100
C	-5.90959900	-2.05685900	1.12940500
C	-5.28406200	-2.29798200	2.31677400
C	-4.37499700	1.23813300	0.16255000
C	-6.19028900	-0.67078800	-0.91115000
C	-5.85804300	0.47949900	-1.65274000
C	-4.95466300	1.45394200	-1.10287000
C	-4.69399000	2.64343300	-1.85968800
H	-4.02956300	3.39490100	-1.44678900
C	-5.26410300	2.84518400	-3.08213900
C	-6.15087700	1.87476900	-3.63181000
C	-6.43996300	0.73639500	-2.93849900
H	-3.78863900	-1.60606100	3.74042300
H	-3.24690000	0.42599400	2.48698100
H	-6.62991500	-2.76986100	0.74178500
H	-5.51638100	-3.19578700	2.88183600
H	-5.04707100	3.75122200	-3.63956600
H	-6.60416300	2.04987000	-4.60283000
H	-7.12847900	0.00863500	-3.35427800
C	3.71629700	2.55691200	-4.08352500
C	3.11813200	2.80528100	-2.88335300
C	3.70857500	2.36575000	-1.65106200
C	4.94971400	1.64040400	-1.70776900
C	5.54617000	1.40956900	-2.99343100
C	4.95359800	1.85315500	-4.13898500
C	3.09604700	2.61796200	-0.40684900

C	5.53425800	1.14629500	-0.52495800
C	4.86626800	1.30618100	0.70627300
C	3.63993100	2.05907800	0.76776100
C	3.02124100	2.24538200	2.04865400
H	2.11331700	2.83386500	2.11480100
C	3.54686900	1.69315200	3.17934100
C	4.75481400	0.94173100	3.11830800
C	5.39959100	0.77204300	1.92810200
H	3.24286700	2.88387800	-5.00412300
H	2.16284700	3.31695600	-2.84827300
H	6.47791600	0.85610200	-3.04483000
H	5.41752900	1.65846700	-5.10118600
H	3.05103500	1.83623300	4.13434400
H	5.17075000	0.51835300	4.02746100
H	6.33297000	0.22033700	1.89431500
C	-7.14375400	-1.67555700	-1.46902900
C	-8.45459100	-1.75990100	-0.98789300
C	-6.73199200	-2.55744800	-2.47477300
C	-9.33573200	-2.71076500	-1.49868400
H	-8.78017200	-1.07729500	-0.20714700
C	-7.61308700	-3.50948200	-2.98462900
H	-5.71585100	-2.49347000	-2.85605300
C	-8.91653600	-3.58805000	-2.49748500
H	-10.35077800	-2.76543200	-1.11648200
H	-7.27926200	-4.18875700	-3.76346700
H	-9.60375400	-4.32885700	-2.89492100
C	6.87213700	0.48602600	-0.58137900
C	8.01616900	1.23777200	-0.87510200
C	7.01104300	-0.88576200	-0.35192800

C	9.26758700	0.62884200	-0.93535100
H	7.91945600	2.30508700	-1.05691500
C	8.26104000	-1.49812900	-0.41612600
H	6.12725500	-1.47732000	-0.13383900
C	9.39392700	-0.74076900	-0.70741500
H	10.14563300	1.22563000	-1.16339600
H	8.34547500	-2.56799600	-0.24480400
H	10.36960600	-1.21442400	-0.75921700
C	0.28103600	-3.62395200	-1.05955800
C	1.37687700	-4.43188100	-1.06527200
C	2.65865700	-3.91729200	-1.44597400
C	2.78275700	-2.51905100	-1.58132300
H	3.68530800	-5.81073400	-1.58996200
H	1.26443100	-5.49420300	-0.88223400
C	3.77164400	-4.73519800	-1.71447200
C	3.98772000	-1.94359700	-2.01312000
C	5.05633900	-2.76665600	-2.31645100
C	4.95220300	-4.16037200	-2.14561000
H	4.06091200	-0.86399900	-2.12006900
H	5.98672500	-2.33575000	-2.67249500
H	5.80877200	-4.78979000	-2.36651900
O	0.42352000	-2.28757100	-1.24178900
C	-1.12407600	-4.04555100	-0.97374000
C	-1.46888300	-5.40403800	-1.02126700
C	-2.13354300	-3.07915700	-0.87575200
C	-2.80211800	-5.79038200	-0.95437200
H	-0.70281600	-6.16585400	-1.12292700
C	-3.46537900	-3.47523200	-0.83456000
H	-1.87803500	-2.02451900	-0.82386600

C	-3.80569800	-4.82596200	-0.86403400
H	-3.05704800	-6.84512200	-0.98714900
H	-4.24011900	-2.71988000	-0.77214200
H	-4.84996800	-5.12356300	-0.82509000
C	3.07598300	-3.36929400	1.54808200
C	2.97230000	-4.76160700	1.75571600
C	4.10505200	-5.55756000	1.84201100
C	5.37519800	-4.98680700	1.73405000
C	5.50120800	-3.61210700	1.54714900
C	4.36611100	-2.81437000	1.45198900
H	1.98906100	-5.21188600	1.82462900
H	4.00053500	-6.62813500	1.98900100
H	6.26129100	-5.61052300	1.80454200
H	6.48530100	-3.15516500	1.48885500
H	4.48883700	-1.74006200	1.35141400
C	1.87925200	-2.54007200	1.38560200
C	1.96718300	-1.31319800	0.73132900
H	2.93449300	-0.84545100	0.55066600
H	1.14502800	-0.60915900	0.83563900
B	0.44116700	-2.93750800	1.97419900
O	-0.67517400	-2.23778700	1.69972600
H	-0.67550100	-1.44705700	1.10627700
O	0.38138900	-3.98569700	2.84639800
C	-0.85200800	-4.30154700	3.50904900
H	-1.30934900	-3.36389000	3.84569600
C	-1.80279400	-5.00771000	2.55417600
H	-1.32936600	-5.91142600	2.15246400
H	-2.71511700	-5.30417300	3.08313400
H	-2.08272300	-4.35042900	1.72578100

C	-0.49424900	-5.16160500	4.70817800
H	-0.03084400	-6.09771600	4.37691300
H	0.21024000	-4.63943400	5.36222400
H	-1.39116900	-5.40454000	5.28578700
C	1.64693400	-1.72383200	-1.18271700
H	1.57862600	-0.67366000	-1.46669600

TSC-minor

M06-2X/BSI SCF energy in dichloromethane: -4205.983129 a.u.

M06-2X/BSII SCF energy in dichloromethane: -4207.047277a.u.

M06-2X/BSII free energy in dichloromethane: -4205.898863 a.u.

P	-1.20184600	0.82095500	-0.31977400
O	-2.76221500	1.29796300	-0.57441200
O	-0.67049500	2.09756900	0.58965700
O	-0.52538300	0.78615900	-1.64429500
O	-1.18097900	-0.34445000	0.62214600
C	-1.03886000	3.36539100	0.22348500
C	-2.31878900	3.80546800	0.50376800
C	-2.69523600	5.12967100	0.10219500
C	-1.71176900	5.98065900	-0.47822300
C	-0.40262600	5.48502500	-0.70590300
C	-0.05942600	4.19269900	-0.39172700
H	-4.79763000	4.97533800	0.61879200
C	-4.02368500	5.62445600	0.22375000
C	-2.06550100	7.30343100	-0.85916700
H	0.33194100	6.14047200	-1.16846100
C	-3.34849200	7.76018100	-0.70187700
C	-4.33925600	6.90194500	-0.16723000

H	-1.29674000	7.94031800	-1.28920100
H	-3.61227700	8.77052900	-0.99869500
H	-5.36092100	7.25686600	-0.07114800
C	-3.28062000	2.86210500	1.13703900
C	-3.97888900	3.16346800	2.35174700
C	-3.48104300	1.62832700	0.54870200
C	-3.72725200	4.34141300	3.10718200
C	-4.93292300	2.23178000	2.85079000
C	-4.43519500	0.68860000	1.03119100
C	-4.41358600	4.59336600	4.26934200
H	-2.97156700	5.03994100	2.76322800
C	-5.63813100	2.52739900	4.04819800
C	-5.15364800	1.01403200	2.15575900
C	-5.39027100	3.68421500	4.74229100
H	-4.20047600	5.49553700	4.83477200
H	-6.37073900	1.80965700	4.40816300
H	-5.89604000	0.31869200	2.54085900
H	-5.93011300	3.90025600	5.65905900
C	-3.16270200	-3.06036700	2.66382200
C	-3.55814000	-1.87187300	2.12559300
C	-4.26417600	-1.81722000	0.87817300
C	-4.53643000	-3.05245000	0.19231000
C	-4.09999700	-4.27943000	0.79594000
C	-3.44024500	-4.28475500	1.98977400
C	-4.66256200	-0.59551200	0.30566300
C	-5.18027300	-3.03659400	-1.05908100
C	-5.56145700	-1.81198700	-1.64033200
C	-5.30363300	-0.57855500	-0.94814200
C	-5.72710900	0.64696600	-1.56038600

H	-5.54696100	1.58287400	-1.04158700
C	-6.35325100	0.65517500	-2.77205100
C	-6.61062900	-0.56718800	-3.45682700
C	-6.23158100	-1.75672500	-2.90776200
H	-2.61660900	-3.07683800	3.60196000
H	-3.31437500	-0.94133000	2.62533000
H	-4.29511700	-5.21369300	0.27973300
H	-3.11030100	-5.22368700	2.42519100
H	-6.66457200	1.59547200	-3.21685400
H	-7.11806400	-0.54767500	-4.41660900
H	-6.44156500	-2.68519000	-3.42817300
C	1.02722900	3.32467000	-4.41004700
C	0.68154100	3.53602800	-3.10902800
C	1.64098200	3.39992000	-2.05030400
C	2.97498900	2.97284900	-2.38110800
C	3.30083200	2.79776700	-3.76939800
C	2.36560800	2.97047500	-4.74669300
C	1.29789500	3.66123000	-0.71149400
C	3.92751600	2.77487100	-1.35826200
C	3.56853700	3.00794600	-0.01212400
C	2.24196700	3.46116900	0.31142800
C	1.90970000	3.69149600	1.68675600
H	0.91299400	4.04525500	1.93061700
C	2.81448300	3.46693000	2.68205900
C	4.12193100	2.99944400	2.36752800
C	4.48461800	2.78503300	1.07000100
H	0.28359700	3.42723800	-5.19431000
H	-0.33926300	3.79619900	-2.85342100
H	4.31679300	2.53451200	-4.04065100

H	2.63935500	2.83606100	-5.78896200
H	2.53919400	3.63975300	3.71798600
H	4.83262700	2.80685900	3.16587600
H	5.48096100	2.42387000	0.84635900
C	-5.44480100	-4.31975400	-1.77561800
C	-6.47338500	-5.16959000	-1.35508400
C	-4.66711300	-4.69097900	-2.87841300
C	-6.72148300	-6.36589200	-2.02447400
H	-7.07961600	-4.88744300	-0.49828100
C	-4.91377400	-5.88863600	-3.54685400
H	-3.87108700	-4.03142600	-3.21549400
C	-5.94180800	-6.72814500	-3.12182500
H	-7.52570300	-7.01429000	-1.68940800
H	-4.30351800	-6.16418200	-4.40178700
H	-6.13561100	-7.66021200	-3.64416300
C	5.30530500	2.31185600	-1.69958700
C	6.42210300	3.10651400	-1.41305700
C	5.50864200	1.07116000	-2.31941000
C	7.70693600	2.67121400	-1.73059200
H	6.27786000	4.07369100	-0.93884500
C	6.79185900	0.63831900	-2.64583400
H	4.64935400	0.44384500	-2.54914900
C	7.89607900	1.43654400	-2.34954300
H	8.56061300	3.30142000	-1.49979200
H	6.92847900	-0.32191300	-3.13469900
H	8.89696800	1.10054000	-2.60356800
C	3.72506600	-3.12846000	-0.70811600
C	2.80081400	-4.02133300	-0.26406700
C	1.41695600	-3.86109400	-0.60185000

C	1.02861900	-2.62917900	-1.17018400
H	0.74920900	-5.81714700	0.01488300
H	3.13262700	-4.90960500	0.26247600
C	0.44973500	-4.86100400	-0.40388500
C	-0.31262300	-2.38014600	-1.50523200
C	-1.25009300	-3.37099200	-1.28751000
C	-0.86691200	-4.61119500	-0.74519500
H	-0.58702400	-1.41505800	-1.92092700
H	-2.29185100	-3.19053100	-1.53489100
H	-1.61912900	-5.37910900	-0.59134000
O	3.33531500	-2.03050900	-1.41407100
C	5.17961000	-3.23005300	-0.54401200
C	5.70482200	-3.82834700	0.60726200
C	6.03991700	-2.67341100	-1.49700100
C	7.08163500	-3.87491900	0.79551500
H	5.03548600	-4.20898400	1.37404800
C	7.41586900	-2.72932100	-1.30488000
H	5.62917800	-2.20209700	-2.38490600
C	7.93890600	-3.32682300	-0.15884800
H	7.48569000	-4.32109200	1.69885300
H	8.08225600	-2.30025300	-2.04710600
H	9.01315200	-3.35945100	-0.00524500
C	4.01372800	-1.00116400	1.69546800
C	4.50669600	-1.35513600	2.96718000
C	5.84073100	-1.14646300	3.29709700
C	6.72435500	-0.60490600	2.36243400
C	6.25800000	-0.25535400	1.09611800
C	4.91896800	-0.43809400	0.77496300
H	3.83223500	-1.78057500	3.69907300

H	6.19398000	-1.41281400	4.28876400
H	7.76879000	-0.45545400	2.62036800
H	6.93315400	0.16349700	0.35320700
H	4.58190600	-0.15447700	-0.21671800
C	2.59696700	-1.17860400	1.34590300
C	2.04143400	-0.44523000	0.30942600
H	2.58297600	0.39013300	-0.13193700
H	0.96248000	-0.36241600	0.26349600
B	1.60573100	-2.17863700	2.12210900
O	0.27200500	-2.18191400	1.92640400
H	-0.18048500	-1.48601800	1.38957800
O	2.16064000	-3.07529300	2.98942300
C	1.33503900	-3.86991000	3.85104900
H	0.47206700	-3.26716600	4.15619200
C	0.84394500	-5.10303200	3.11178200
H	1.69648700	-5.69088200	2.75107900
H	0.24801900	-5.73461600	3.77921400
H	0.22115500	-4.81429800	2.26116100
C	2.17552300	-4.21921800	5.06715700
H	3.06044700	-4.79006000	4.76453100
H	2.50673000	-3.31224100	5.58246900
H	1.59596400	-4.82422800	5.77107900
C	2.05189000	-1.63403700	-1.349668000
H	1.85896000	-0.77849600	-1.992442000

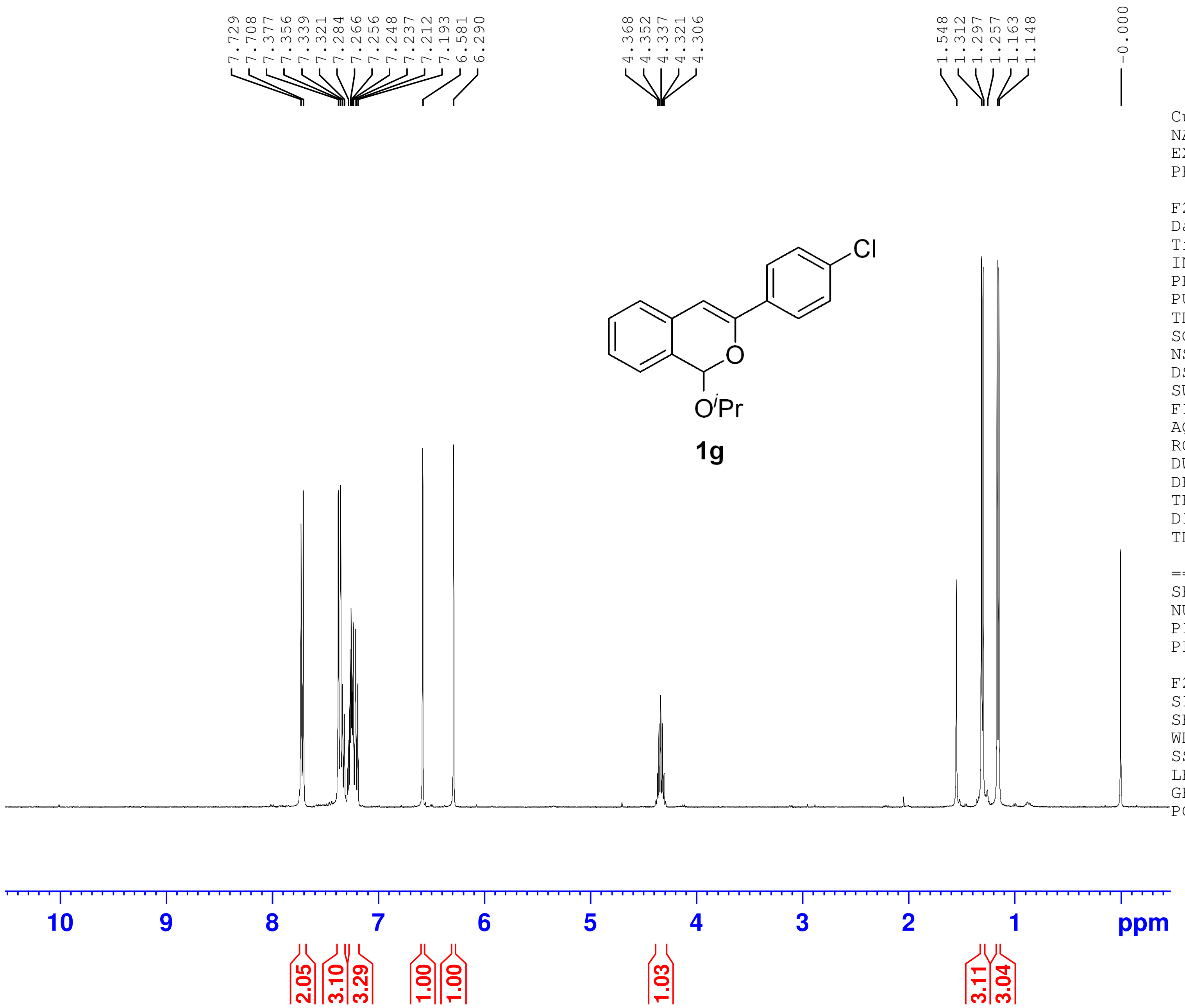


```
Current Data Parameters
NAME          lsx-1-P-Cl-H
EXPNO                    1
PROCNO                   1

F2 - Acquisition Parameters
Date_             20211228
Time                 19.52
INSTRUM              spect
PROBHD     5 mm PABBO BB/
PULPROG               zg30
TD                   65536
SOLVENT              CDCl3
NS                       6
DS                       2
SWH               8012.820 Hz
FIDRES            0.122266 Hz
AQ               4.0894465 sec
RG                  142.88
DW                  62.400 usec
DE                    6.50 usec
TE                   295.2 K
D1              1.00000000 sec
TD0                      1

======== CHANNEL f1 ========
SFO1           400.1324710 MHz
NUC1                    1H
P1                   14.50 usec
PLW1           11.99499989 W

F2 - Processing parameters
SI                   65536
SF             400.1300115 MHz
WDW                     EM
SSB                      0
LB                    0.30 Hz
GB                       0
PC                    1.00
```

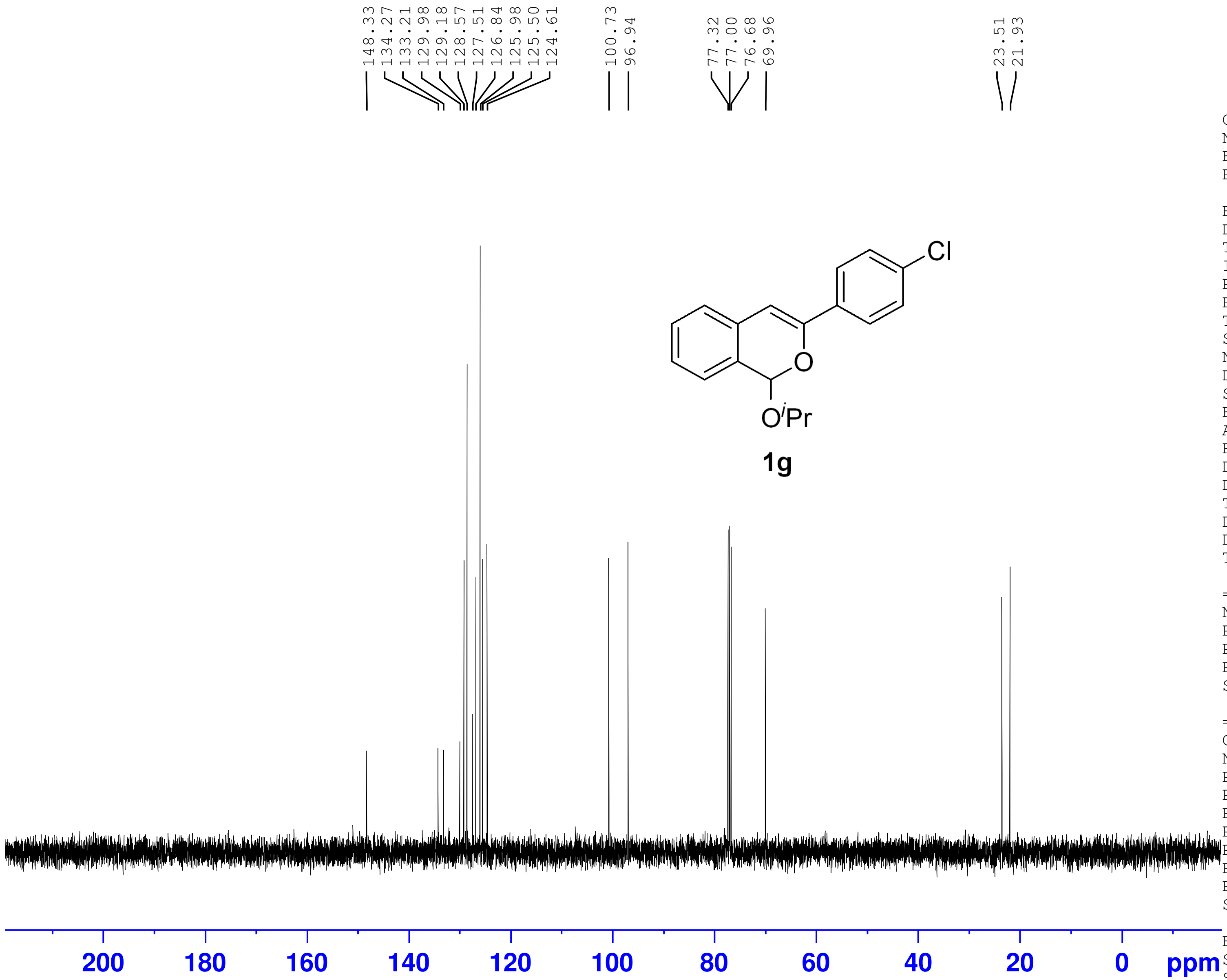


```
Current Data Parameters
NAME      lsx-project3-1c-c
EXPNO                     1
PROCNO                    1

F2 - Acquisition Parameters
Date_              20220722
Time                  20.04
INSTRUM               spect
PROBHD    5 mm DUL 13C-1
PULPROG             zgpg30
TD                   65536
SOLVENT              CDCl3
NS                      20
DS                       1
SWH             24038.461 Hz
FIDRES           0.366798 Hz
AQ              1.3631488 sec
RG                    2050
DW                  20.800 usec
DE                    6.00 usec
TE                   293.1 K
D1              2.00000000 sec
D11             0.03000000 sec
TD0                      1

======== CHANNEL f1 ========
NUC1                   13C
P1                   40.00 usec
PL1                  -3.00 dB
PL1W           60.64365387 W
SFO1           100.6228298 MHz

======== CHANNEL f2 ========
CPDPRG[2           waltz16
NUC2                    1H
PCPD2                80.00 usec
PL2                  -1.00 dB
PL12                 14.39 dB
PL13                 18.00 dB
PL2W           12.17476940 W
PL12W           0.35193357 W
PL13W           0.15327126 W
SFO2           400.1316005 MHz

F2 - Processing parameters
SI                   32768
SF             100.6127795 MHz
WDW                     EM
SSB                      0
LB                    1.00 Hz
GB                       0
PC                    1.40
```

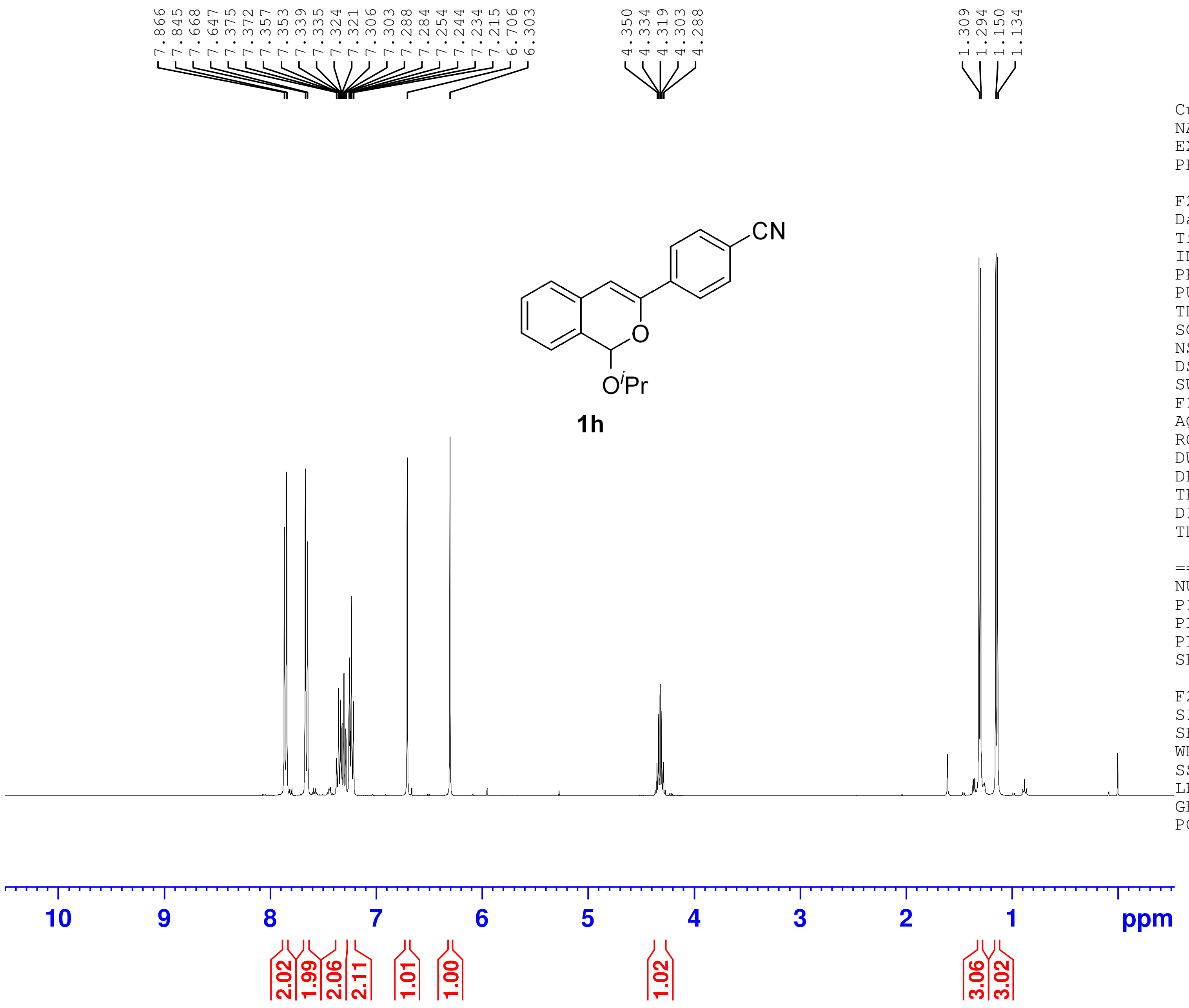


```
Current Data Parameters
NAME      lsx-project3-1d-h
EXPNO                     1
PROCNO                    1

F2 - Acquisition Parameters
Date_              20220722
Time                  20.09
INSTRUM               spect
PROBHD   5 mm DUL 13C-1
PULPROG                zg30
TD                    65536
SOLVENT               CDCl3
NS                        6
DS                        2
SWH                8223.685 Hz
FIDRES             0.125483 Hz
AQ                3.9845889 sec
RG                      161
DW                   60.800 usec
DE                     6.00 usec
TE                    292.9 K
D1               1.00000000 sec
TD0                       1

======== CHANNEL f1 ========
NUC1                     1H
P1                    15.80 usec
PL1                   -1.00 dB
PL1W            12.17476940 W
SFO1            400.1324710 MHz

F2 - Processing parameters
SI                    32768
SF              400.1300159 MHz
WDW                      EM
SSB                       0
LB                     0.30 Hz
GB                        0
PC                     1.00
```

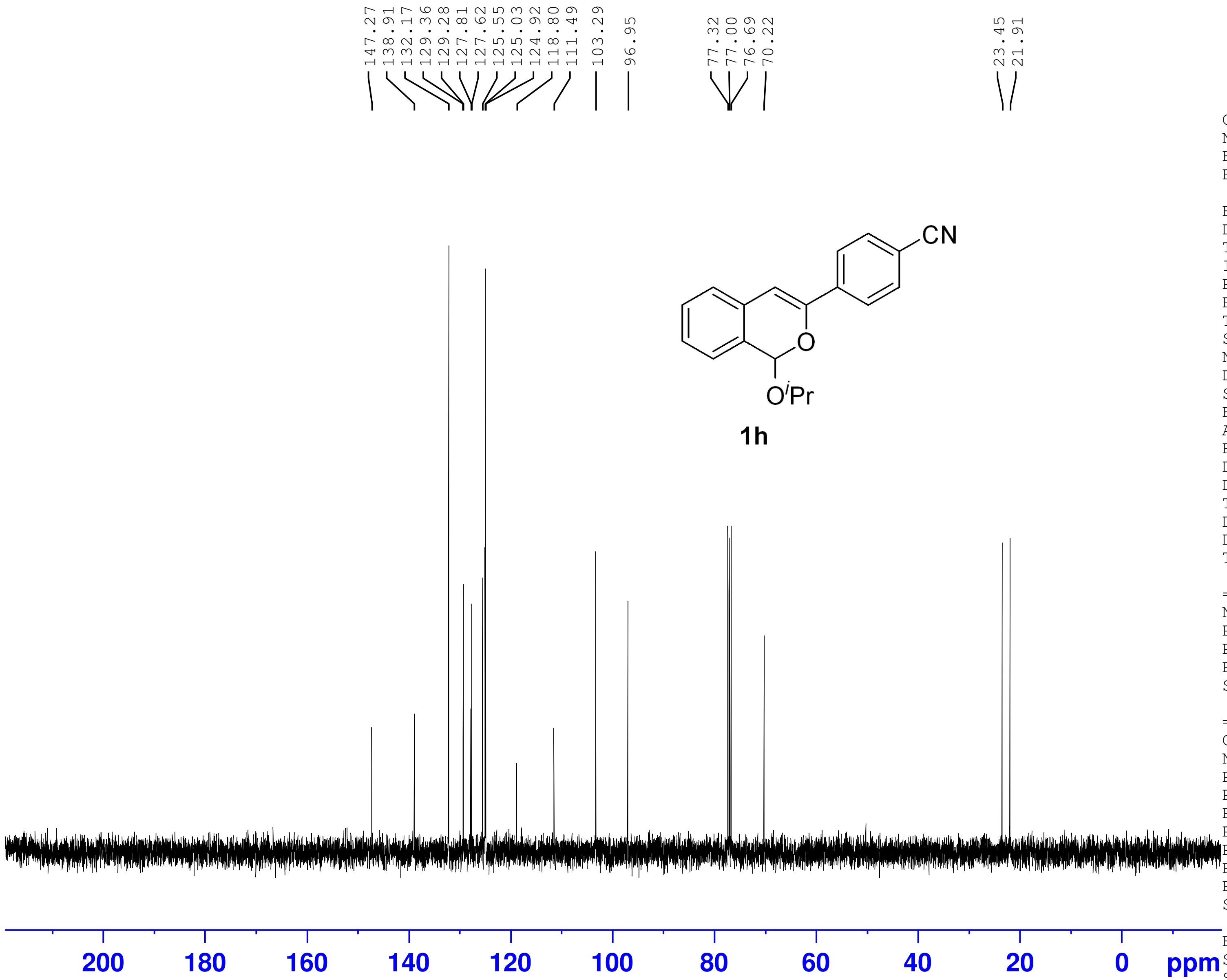


```
Current Data Parameters
NAME     lsx-project3-1d-c
EXPNO                    1
PROCNO                   1

F2 - Acquisition Parameters
Date_             20220722
Time                 20.07
INSTRUM              spect
PROBHD   5 mm DUL 13C-1
PULPROG            zgpg30
TD                   65536
SOLVENT              CDCl3
NS                      25
DS                       1
SWH             24038.461 Hz
FIDRES           0.366798 Hz
AQ              1.3631488 sec
RG                    2050
DW                  20.800 usec
DE                    6.00 usec
TE                   293.1 K
D1             2.00000000 sec
D11            0.03000000 sec
TD0                      1

======== CHANNEL f1 ========
NUC1                   13C
P1                   40.00 usec
PL1                  -3.00 dB
PL1W           60.64365387 W
SFO1           100.6228298 MHz

======== CHANNEL f2 ========
CPDPRG[2           waltz16
NUC2                    1H
PCPD2                80.00 usec
PL2                  -1.00 dB
PL12                 14.39 dB
PL13                 18.00 dB
PL2W           12.17476940 W
PL12W           0.35193357 W
PL13W           0.15327126 W
SFO2           400.1316005 MHz

F2 - Processing parameters
SI                   32768
SF             100.6127810 MHz
WDW                     EM
SSB                      0
LB                    1.00 Hz
GB                       0
PC                    1.40
```

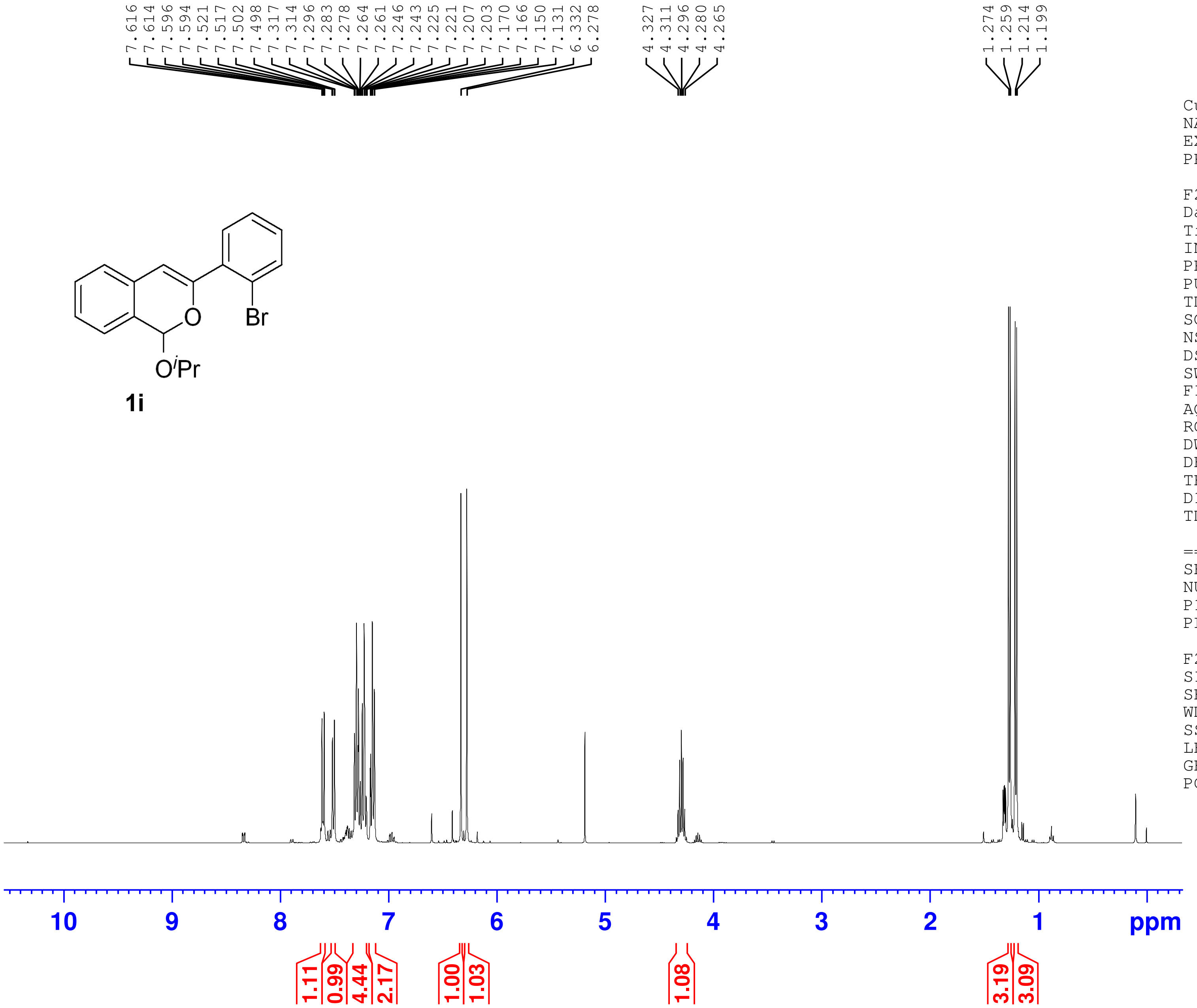


```
Current Data Parameters
NAME       lsx-project3-1e-h-fr
EXPNO                     1
PROCNO                    1

F2 - Acquisition Parameters
Date_              20220723
Time                  16.25
INSTRUM               spect
PROBHD     5 mm PABBO BB/
PULPROG                zg30
TD                    65536
SOLVENT               CDCl3
NS                       10
DS                        2
SWH             8012.820 Hz
FIDRES          0.122266 Hz
AQ             4.0894465 sec
RG                    15.71
DW               62.400 usec
DE                 6.50 usec
TE                  295.4 K
D1            1.00000000 sec
TD0                       1

======== CHANNEL f1 ========
SFO1         400.1324710 MHz
NUC1                     1H
P1                14.50 usec
PLW1          11.99499989 W

F2 - Processing parameters
SI                    65536
SF           400.1300481 MHz
WDW                      EM
SSB                       0
LB                  0.30 Hz
GB                        0
PC                     1.00
```

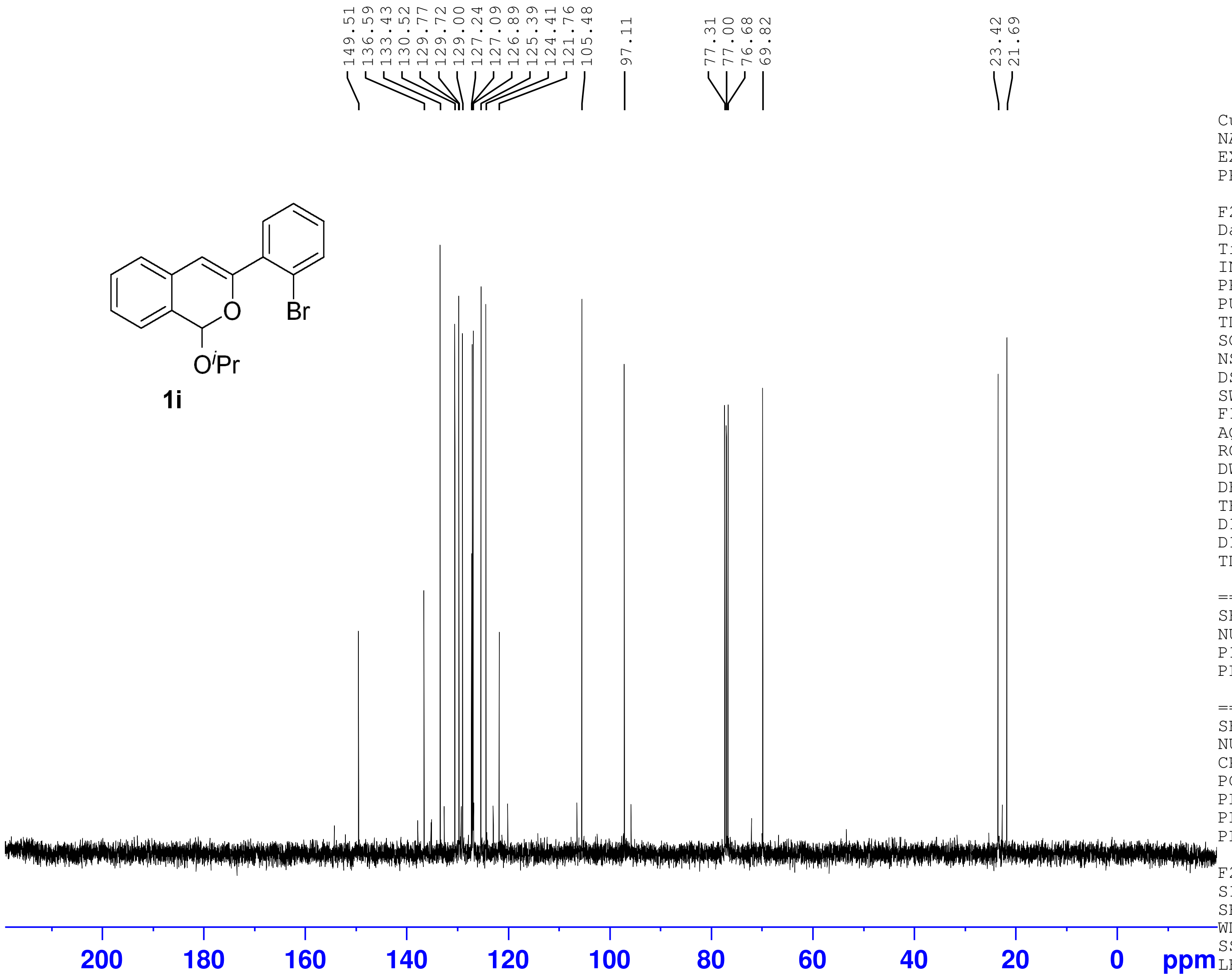

Current Data Parameters
NAME lsx-project3-1e-c-fr
EXPNO 1
PROCNO 1

F2 - Acquisition Parameters
Date_ 20220723
Time 16.27
INSTRUM spect
PROBHD 5 mm PABBO BB/
PULPROG zgpg30
TD 65536
SOLVENT CDCl3
NS 10
DS 2
SWH 24038.461 Hz
FIDRES 0.366798 Hz
AQ 1.3631488 sec
RG 196.92
DW 20.800 usec
DE 6.50 usec
TE 295.8 K
D1 2.00000000 sec
D11 0.03000000 sec
TD0 1

======== CHANNEL f1 ========
SFO1 100.6228298 MHz
NUC1 13C
P1 9.70 usec
PLW1 46.98899841 W

======== CHANNEL f2 ========
SFO2 400.1316005 MHz
NUC2 1H
CPDPRG[2 waltz16
PCPD2 90.00 usec
PLW2 11.99499989 W
PLW12 0.34213999 W
PLW13 0.27713001 W

F2 - Processing parameters
SI 32768
SF 100.6127927 MHz
WDW EM
SSB 0
LB 1.00 Hz
GB 0
PC 1.40

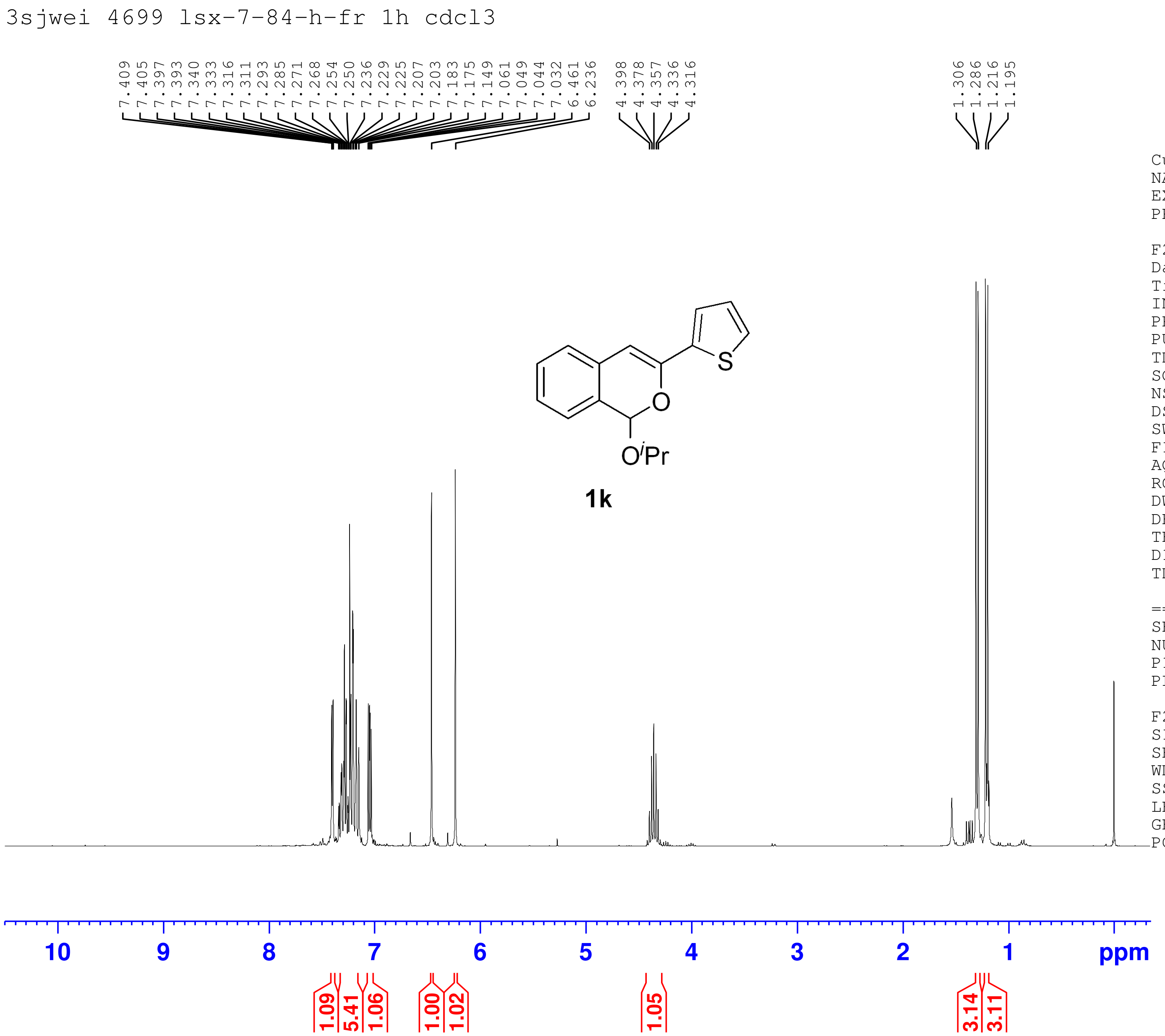


```
Current Data Parameters
NAME                      1g
EXPNO                   4699
PROCNO                     1

F2 - Acquisition Parameters
Date_               20210719
Time                    9.26
INSTRUM                spect
PROBHD     5 mm PABBO BB-
PULPROG                 zg30
TD                     65536
SOLVENT                CDCl3
NS                        16
DS                         2
SWH                 6009.615 Hz
FIDRES              0.091699 Hz
AQ                 5.4525952 sec
RG                       114
DW                    83.200 usec
DE                      6.50 usec
TE                     296.2 K
D1                1.00000000 sec
TD0                        1

======== CHANNEL f1 ========
SFO1             300.1318534 MHz
NUC1                      1H
P1                     10.00 usec
PLW1             14.00000000 W

F2 - Processing parameters
SI                     65536
SF               300.1300145 MHz
WDW                       EM
SSB                        0
LB                      0.30 Hz
GB                         0
PC                      1.00
```

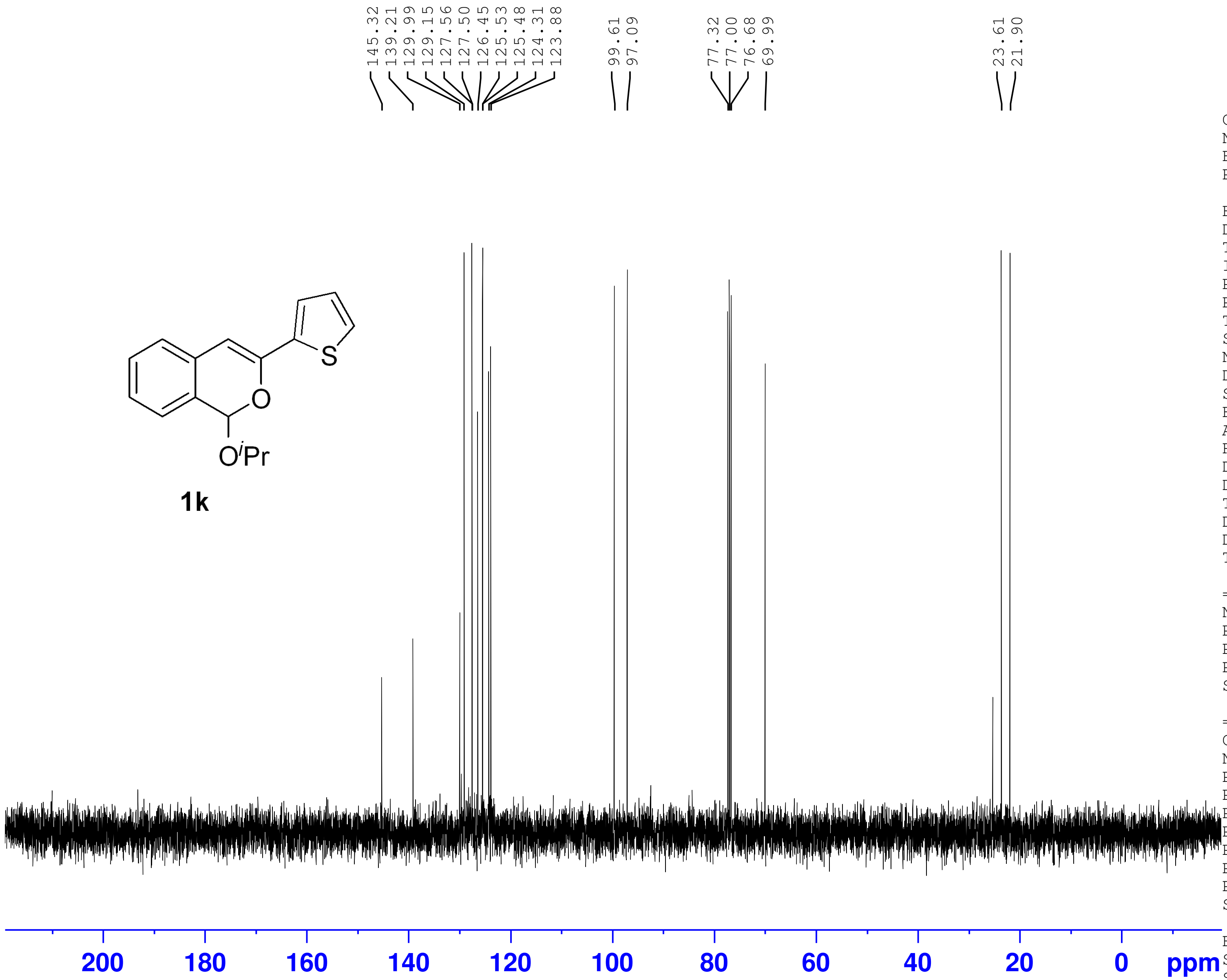

1k

```
Current Data Parameters
NAME     lsx-project3-1g-c
EXPNO                    1
PROCNO                   1

F2 - Acquisition Parameters
Date_             20220722
Time                 20.20
INSTRUM              spect
PROBHD    5 mm DUL 13C-1
PULPROG            zgpg30
TD                   65536
SOLVENT              CDCl3
NS                      26
DS                       1
SWH              24038.461 Hz
FIDRES            0.366798 Hz
AQ               1.3631488 sec
RG                    2050
DW                  20.800 usec
DE                    6.00 usec
TE                   293.1 K
D1              2.00000000 sec
D11             0.03000000 sec
TD0                      1

======== CHANNEL f1 ========
NUC1                   13C
P1                   40.00 usec
PL1                  -3.00 dB
PL1W           60.64365387 W
SFO1           100.6228298 MHz

======== CHANNEL f2 ========
CPDPRG[2           waltz16
NUC2                    1H
PCPD2                80.00 usec
PL2                  -1.00 dB
PL12                 14.39 dB
PL13                 18.00 dB
PL2W           12.17476940 W
PL12W           0.35193357 W
PL13W           0.15327126 W
SFO2           400.1316005 MHz

F2 - Processing parameters
SI                   32768
SF             100.6127824 MHz
WDW                     EM
SSB                      0
LB                    1.00 Hz
GB                       0
PC                    1.40
```

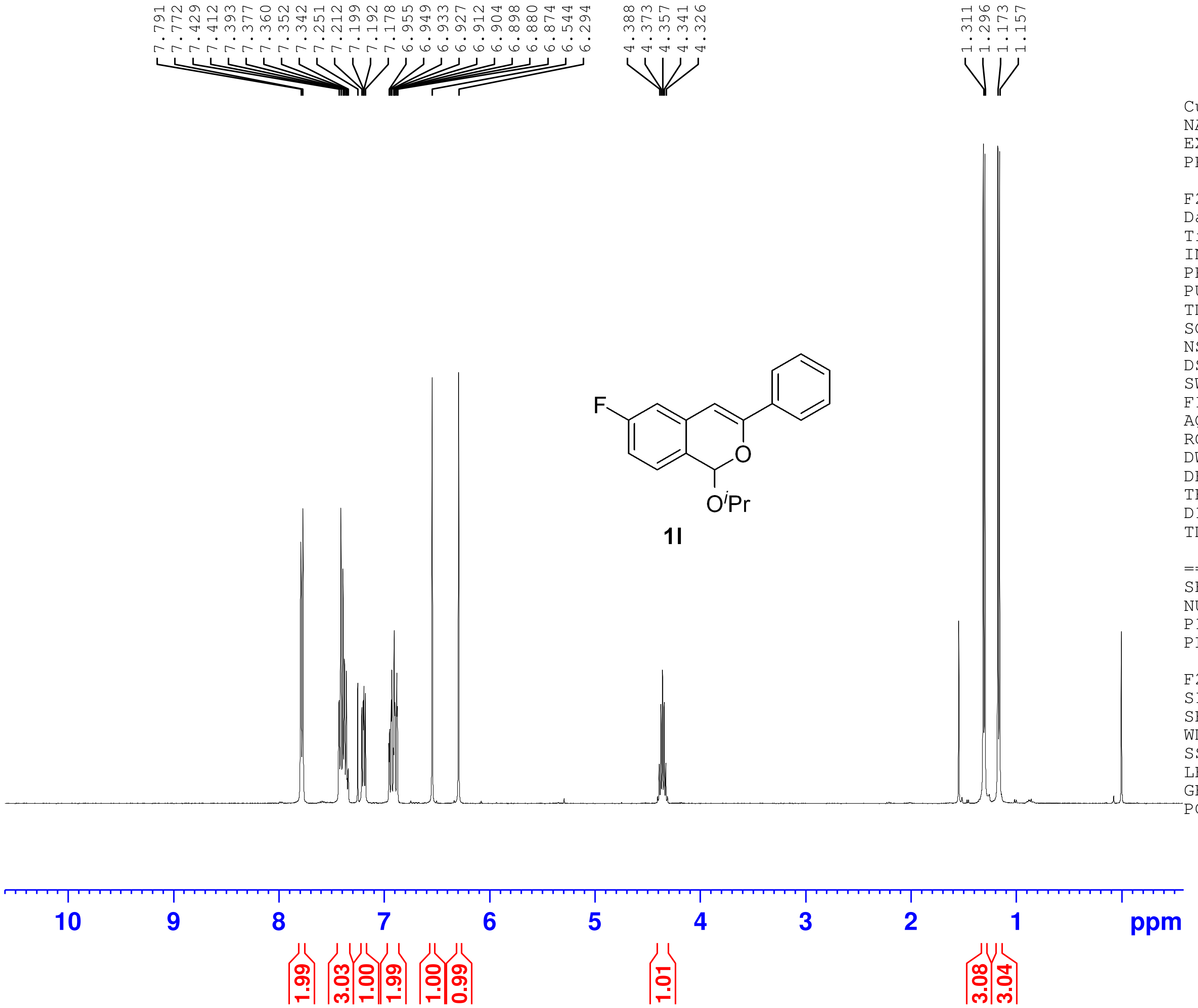


```
Current Data Parameters
NAME          lsx-1-7f-h
EXPNO                  1
PROCNO                 1

F2 - Acquisition Parameters
Date_          20211228
Time              19.37
INSTRUM           spect
PROBHD   5 mm PABBO BB/
PULPROG            zg30
TD                65536
SOLVENT           CDCl3
NS                    7
DS                    2
SWH            8012.820 Hz
FIDRES         0.122266 Hz
AQ            4.0894465 sec
RG               112.31
DW               62.400 usec
DE                 6.50 usec
TE                294.9 K
D1           1.00000000 sec
TD0                   1

======== CHANNEL f1 ========
SFO1        400.1324710 MHz
NUC1                 1H
P1                14.50 usec
PLW1        11.99499989 W

F2 - Processing parameters
SI                65536
SF          400.1300135 MHz
WDW                  EM
SSB                   0
LB                 0.30 Hz
GB                    0
PC                 1.00
```

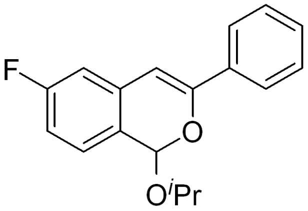

1l

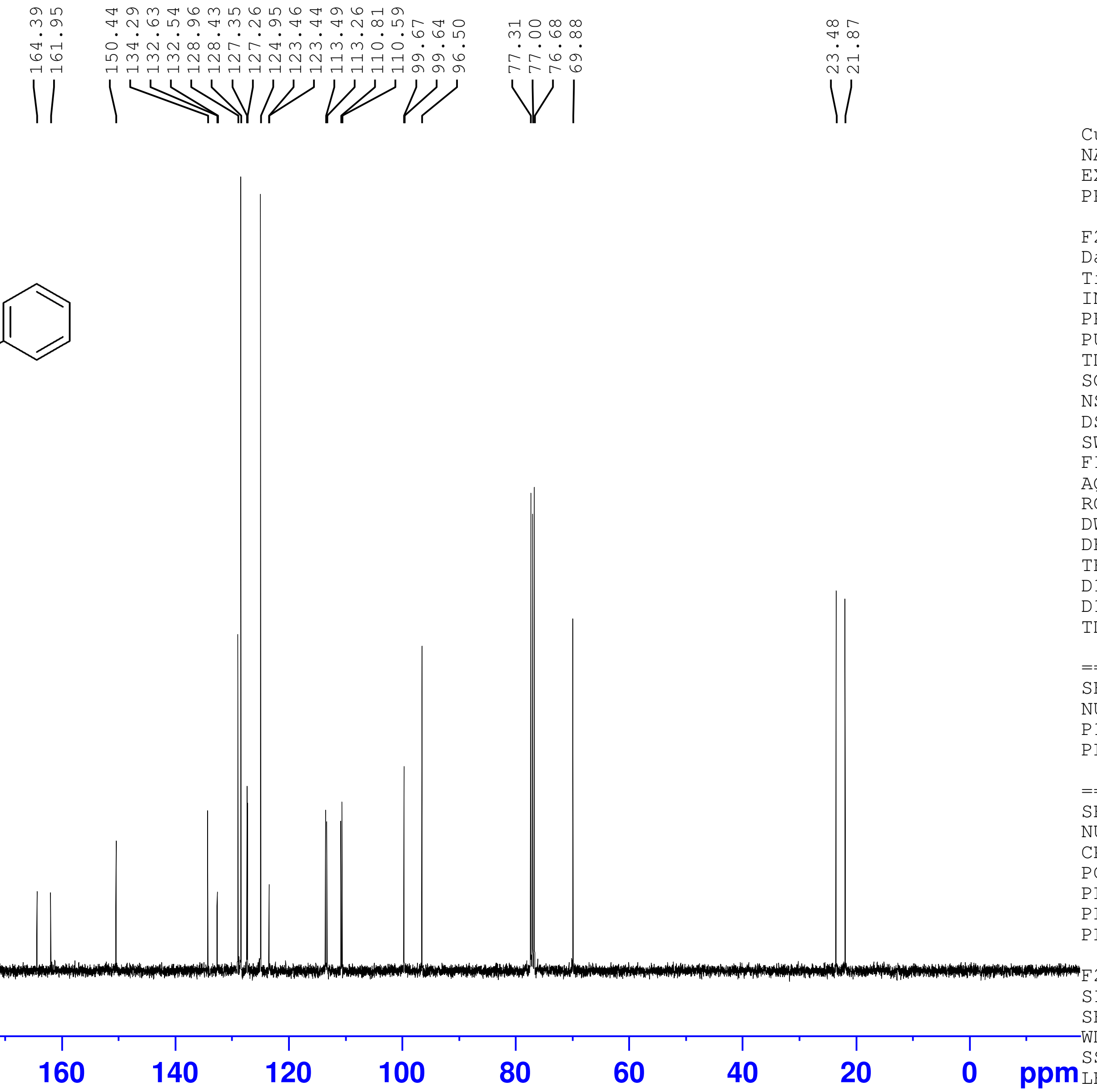

Current Data Parameters
NAME lsx-project3-1h-c-fr
EXPNO 1
PROCNO 1

F2 - Acquisition Parameters
Date_ 20220723
Time 16.36
INSTRUM spect
PROBHD 5 mm PABBO BB/
PULPROG zgpg30
TD 65536
SOLVENT CDCl3
NS 72
DS 2
SWH 24038.461 Hz
FIDRES 0.366798 Hz
AQ 1.3631488 sec
RG 196.92
DW 20.800 usec
DE 6.50 usec
TE 296.3 K
D1 2.00000000 sec
D11 0.03000000 sec
TD0 1

======== CHANNEL f1 ========
SFO1 100.6228298 MHz
NUC1 13C
P1 9.70 usec
PLW1 46.98899841 W

======== CHANNEL f2 ========
SFO2 400.1316005 MHz
NUC2 1H
CPDPRG[2 waltz16
PCPD2 90.00 usec
PLW2 11.99499989 W
PLW12 0.34213999 W
PLW13 0.27713001 W

F2 - Processing parameters
SI 32768
SF 100.6127787 MHz
WDW EM
SSB 0
LB 1.00 Hz
GB 0
PC 1.40

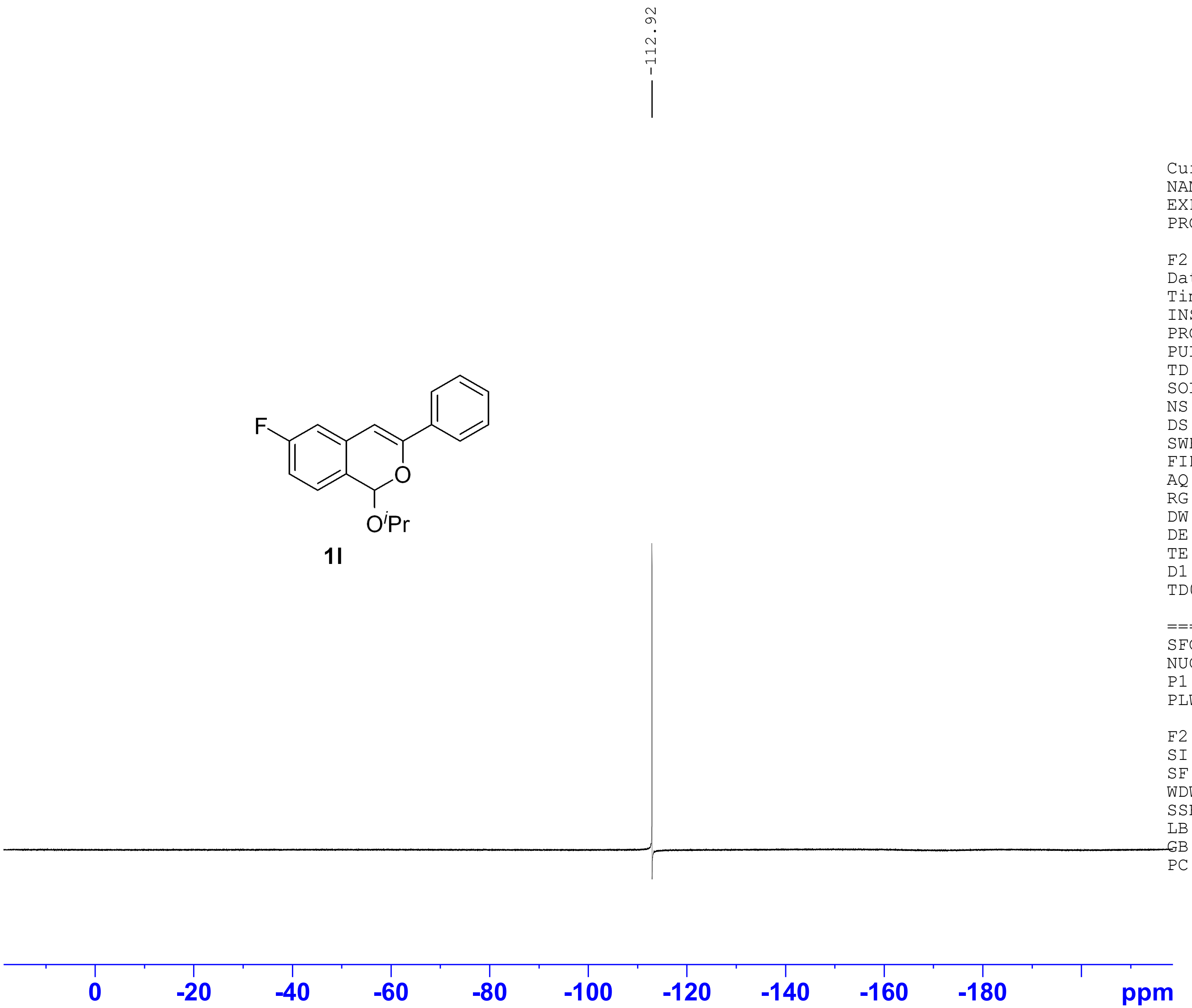


```
Current Data Parameters
NAME           lsx-1-7f-f
EXPNO                   1
PROCNO                  1

F2 - Acquisition Parameters
Date_            20211228
Time                19.39
INSTRUM             spect
PROBHD    5 mm PABBO BB/
PULPROG            zgflqn
TD                 131072
SOLVENT             CDCl3
NS                      8
DS                      4
SWH            89285.711 Hz
FIDRES          0.681196 Hz
AQ             0.7340032 sec
RG                 196.92
DW                  5.600 usec
DE                   6.50 usec
TE                  294.9 K
D1             1.00000000 sec
TD0                     1

======== CHANNEL f1 ========
SFO1         376.4607164 MHz
NUC1                 19F
P1                  14.70 usec
PLW1         15.99600029 W

F2 - Processing parameters
SI                  65536
SF           376.4983660 MHz
WDW                    EM
SSB                     0
LB                   0.30 Hz
GB                      0
PC                   1.00
```

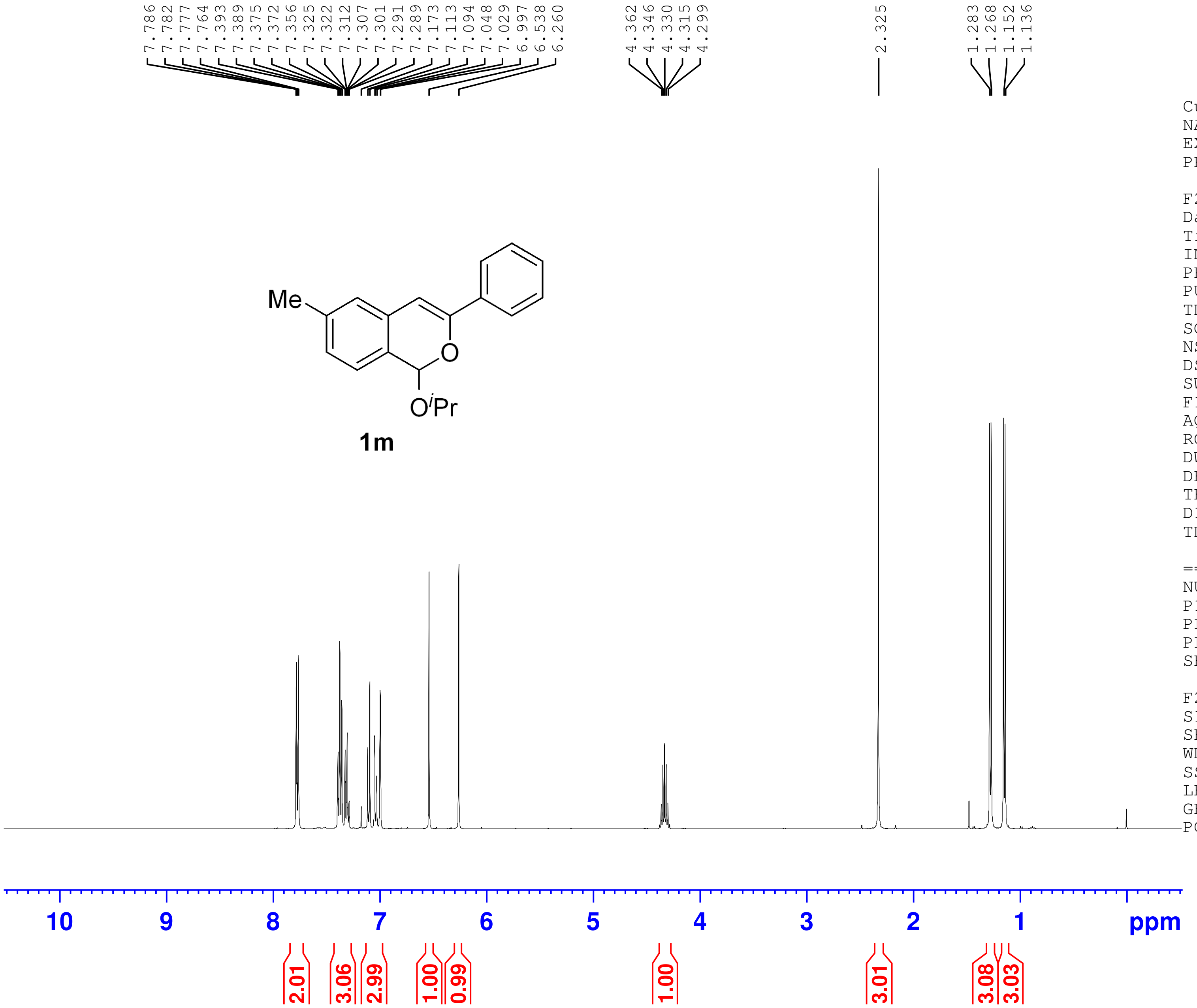

Current Data Parameters
NAME lsx-project3-1i-h
EXPNO 1
PROCNO 1

F2 - Acquisition Parameters
Date_ 20220722
Time 20.23
INSTRUM spect
PROBHD 5 mm DUL 13C-1
PULPROG zg30
TD 65536
SOLVENT CDCl3
NS 7
DS 2
SWH 8223.685 Hz
FIDRES 0.125483 Hz
AQ 3.9845889 sec
RG 64
DW 60.800 usec
DE 6.00 usec
TE 292.8 K
D1 1.00000000 sec
TD0 1

======== CHANNEL f1 ========
NUC1 1H
P1 15.80 usec
PL1 -1.00 dB
PL1W 12.17476940 W
SFO1 400.1324710 MHz

F2 - Processing parameters
SI 32768
SF 400.1300443 MHz
WDW EM
SSB 0
LB 0.30 Hz
GB 0
PC 1.00

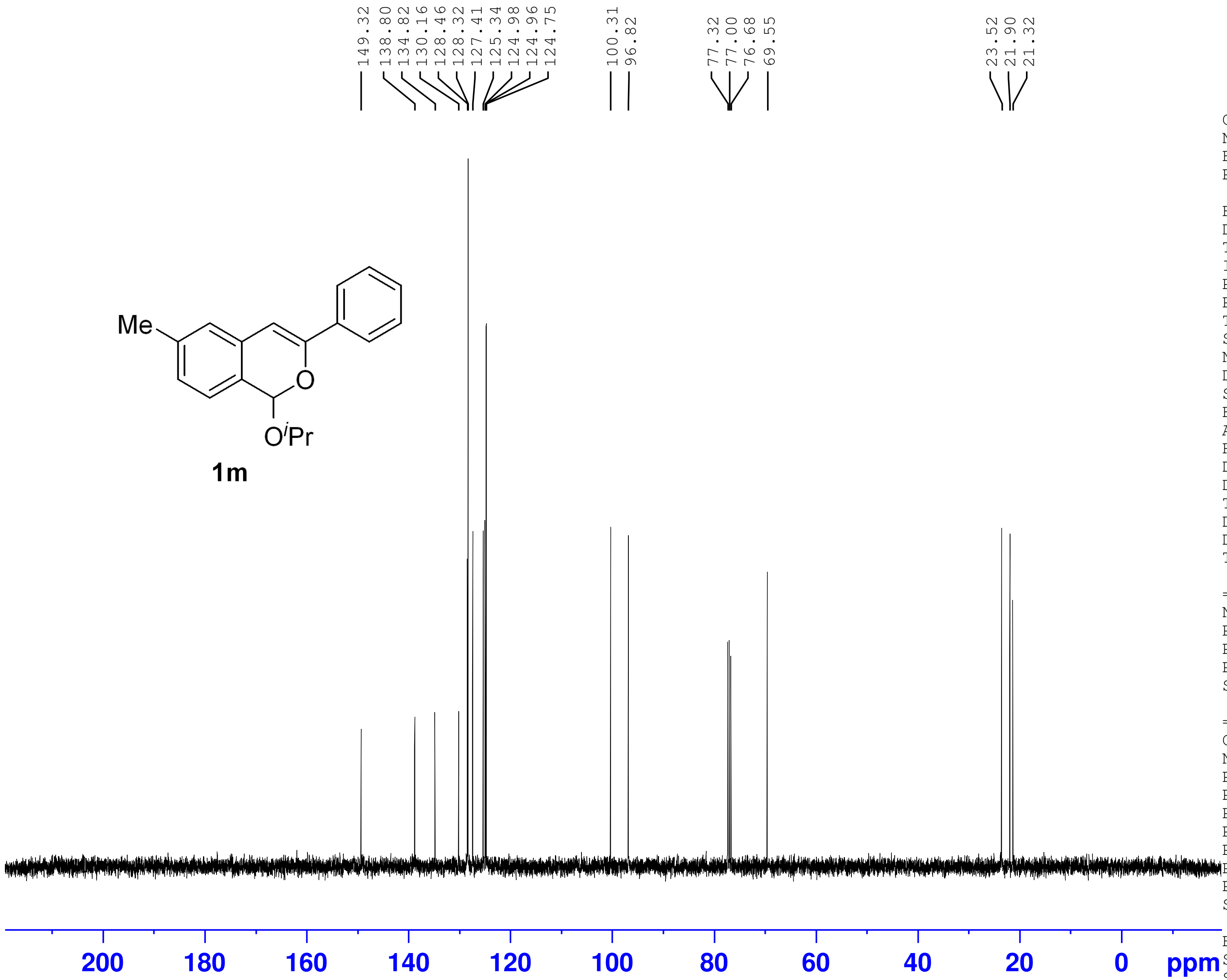


```
Current Data Parameters
NAME      lsx-project3-1i-c
EXPNO                    1
PROCNO                   1

F2 - Acquisition Parameters
Date_             20220722
Time                 20.26
INSTRUM              spect
PROBHD    5 mm DUL 13C-1
PULPROG            zgpg30
TD                  65536
SOLVENT             CDCl3
NS                     33
DS                      1
SWH             24038.461 Hz
FIDRES           0.366798 Hz
AQ              1.3631488 sec
RG                   2050
DW                 20.800 usec
DE                   6.00 usec
TE                  293.1 K
D1             2.00000000 sec
D11            0.03000000 sec
TD0                     1

======== CHANNEL f1 ========
NUC1                  13C
P1                  40.00 usec
PL1                 -3.00 dB
PL1W          60.64365387 W
SFO1          100.6228298 MHz

======== CHANNEL f2 ========
CPDPRG[2          waltz16
NUC2                   1H
PCPD2               80.00 usec
PL2                 -1.00 dB
PL12                14.39 dB
PL13                18.00 dB
PL2W          12.17476940 W
PL12W          0.35193357 W
PL13W          0.15327126 W
SFO2          400.1316005 MHz

F2 - Processing parameters
SI                  32768
SF            100.6127854 MHz
WDW                    EM
SSB                     0
LB                   1.00 Hz
GB                      0
PC                   1.40
```

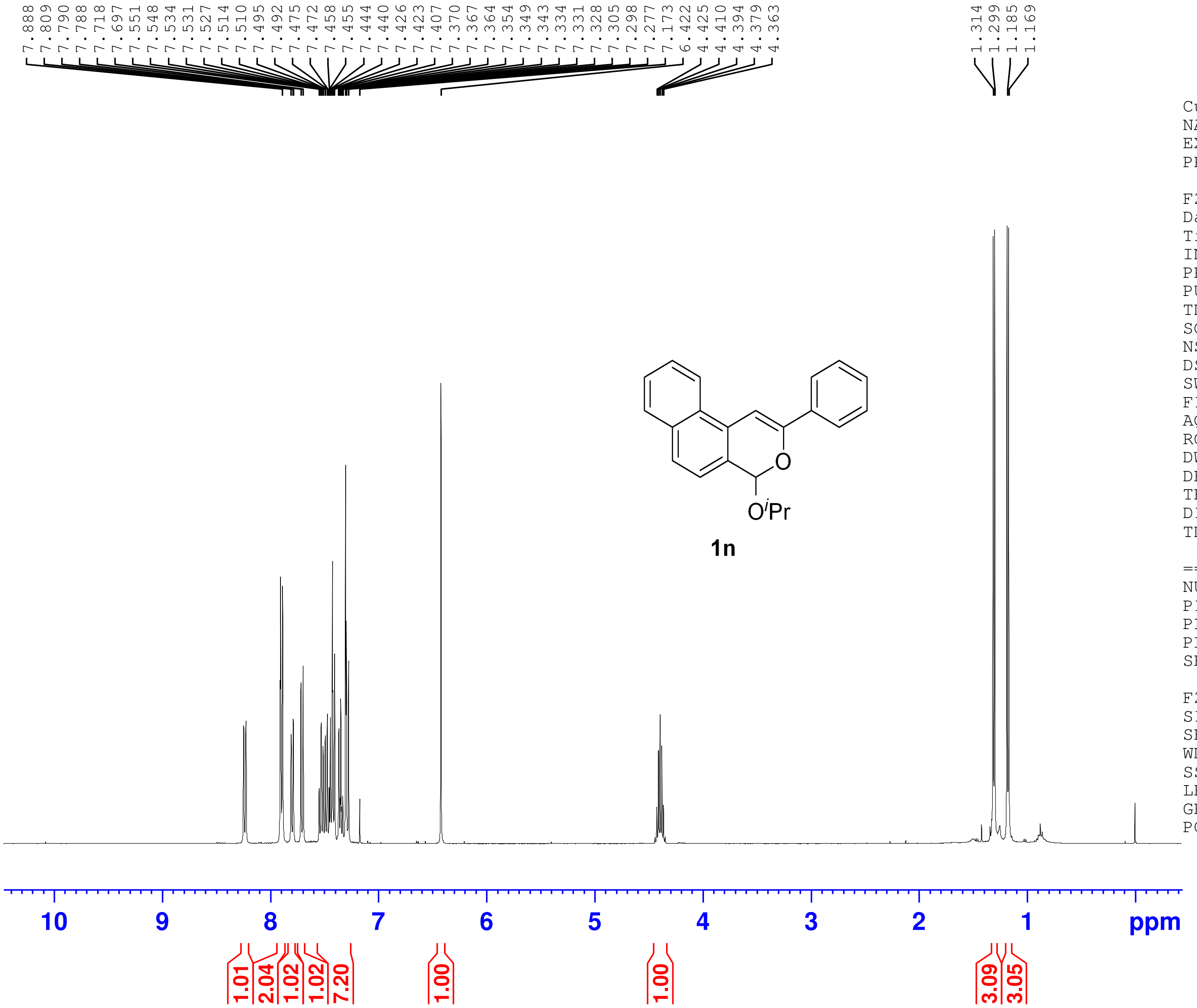


```
Current Data Parameters
NAME      lsx-project3-1j-h
EXPNO                     1
PROCNO                    1

F2 - Acquisition Parameters
Date_              20220722
Time                  20.29
INSTRUM               spect
PROBHD    5 mm DUL 13C-1
PULPROG                zg30
TD                    65536
SOLVENT               CDCl3
NS                        8
DS                        2
SWH                8223.685 Hz
FIDRES             0.125483 Hz
AQ                3.9845889 sec
RG                      101
DW                   60.800 usec
DE                     6.00 usec
TE                    292.8 K
D1               1.00000000 sec
TD0                       1

======== CHANNEL f1 ========
NUC1                     1H
P1                    15.80 usec
PL1                   -1.00 dB
PL1W            12.17476940 W
SFO1            400.1324710 MHz

F2 - Processing parameters
SI                    32768
SF              400.1300444 MHz
WDW                      EM
SSB                       0
LB                     0.30 Hz
GB                        0
PC                     1.00
```

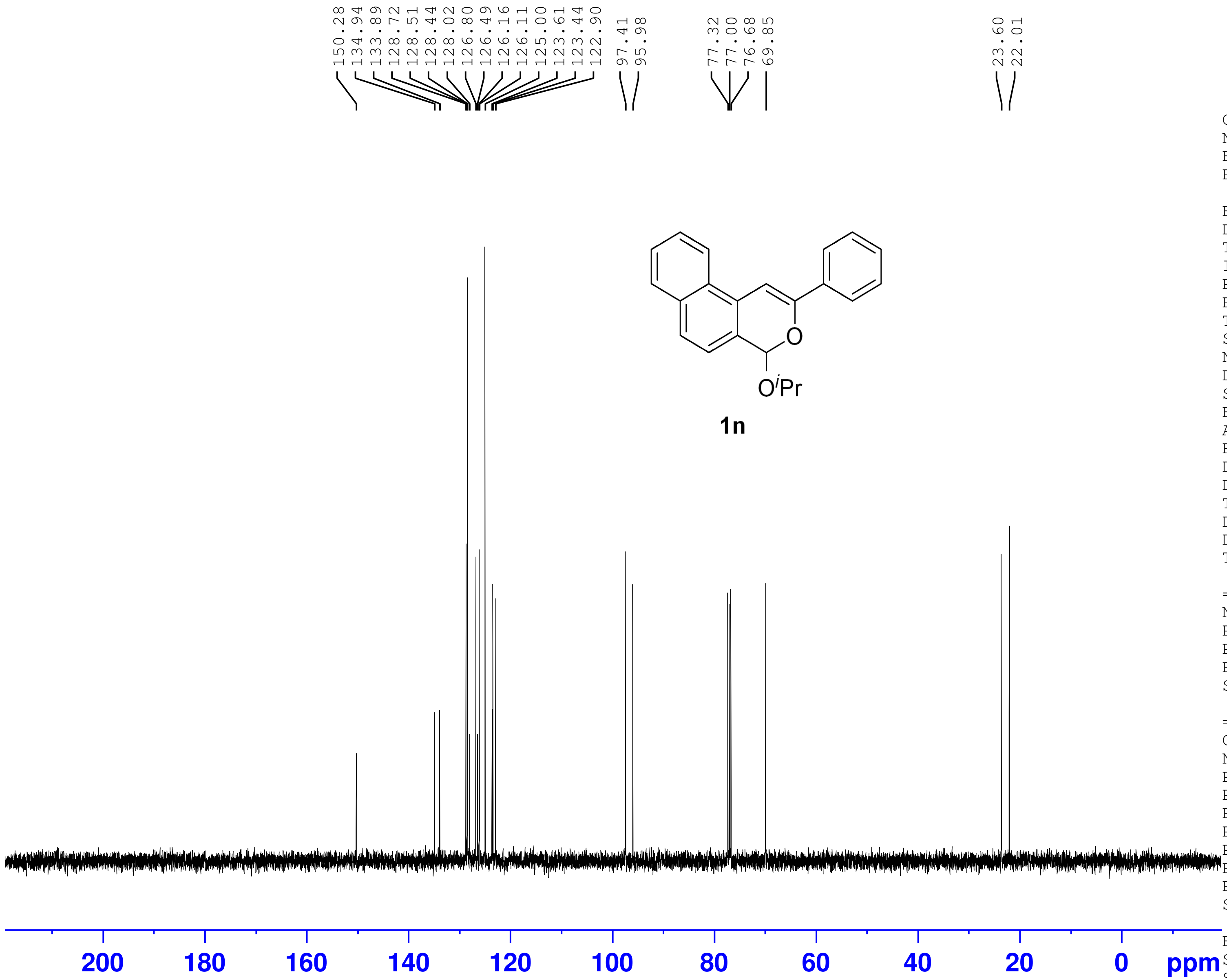

1n

Current Data Parameters
NAME lsx-project3-1j-c
EXPNO 1
PROCNO 1

F2 - Acquisition Parameters
Date_ 20220722
Time 20.32
INSTRUM spect
PROBHD 5 mm DUL 13C-1
PULPROG zgpg30
TD 65536
SOLVENT CDCl3
NS 47
DS 1
SWH 24038.461 Hz
FIDRES 0.366798 Hz
AQ 1.3631488 sec
RG 2050
DW 20.800 usec
DE 6.00 usec
TE 293.1 K
D1 2.00000000 sec
D11 0.03000000 sec
TD0 1

======== CHANNEL f1 ========
NUC1 13C
P1 40.00 usec
PL1 -3.00 dB
PL1W 60.64365387 W
SFO1 100.6228298 MHz

======== CHANNEL f2 ========
CPDPRG[2 waltz16
NUC2 1H
PCPD2 80.00 usec
PL2 -1.00 dB
PL12 14.39 dB
PL13 18.00 dB
PL2W 12.17476940 W
PL12W 0.35193357 W
PL13W 0.15327126 W
SFO2 400.1316005 MHz

F2 - Processing parameters
SI 32768
SF 100.6127847 MHz
WDW EM
SSB 0
LB 1.00 Hz
GB 0
PC 1.40

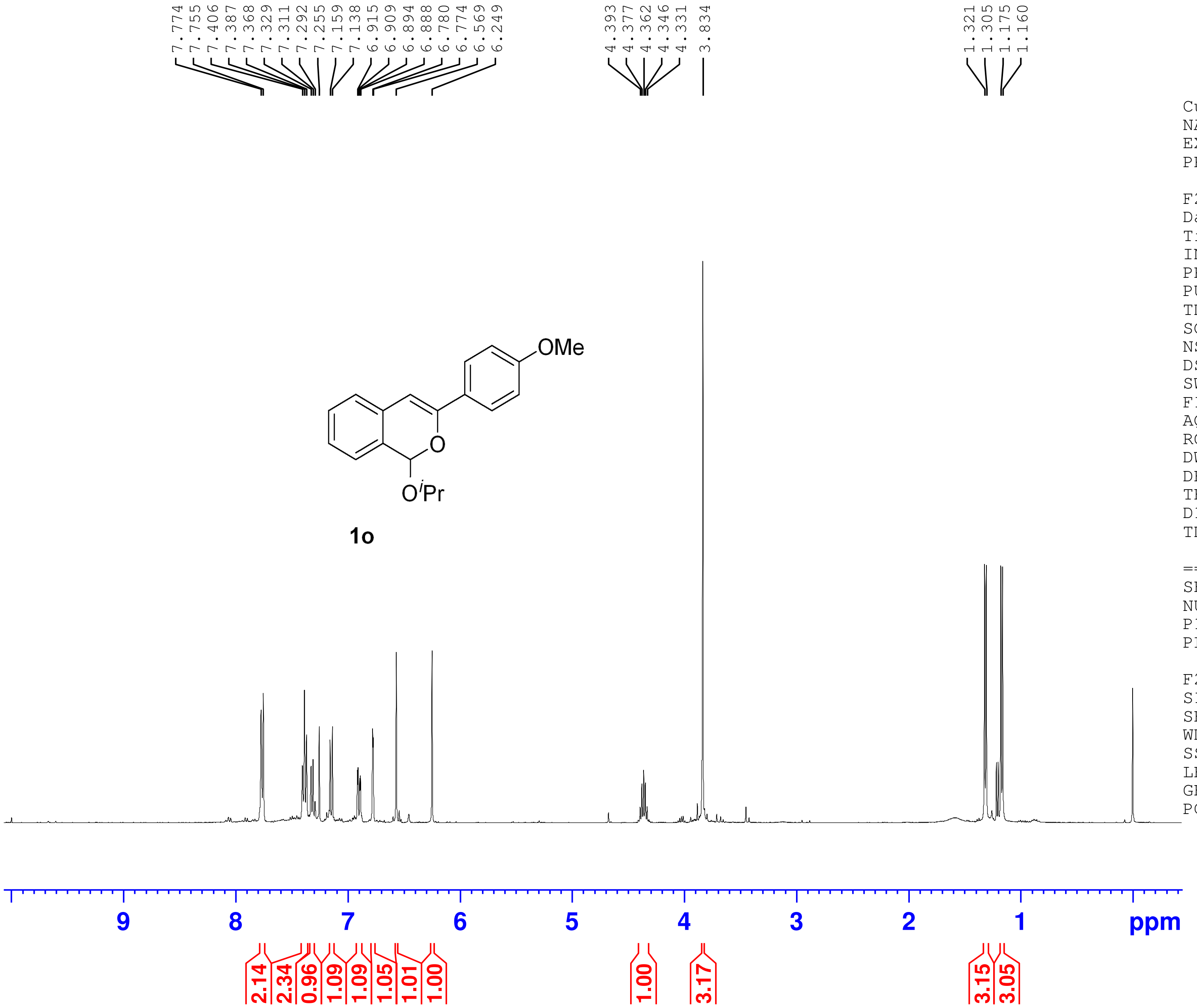


```
Current Data Parameters
NAME        lsx-1-6ome-c
EXPNO                  1
PROCNO                 1

F2 - Acquisition Parameters
Date_           20211228
Time               19.50
INSTRUM            spect
PROBHD   5 mm PABBO BB/
PULPROG             zg30
TD                 65536
SOLVENT            CDCl3
NS                     9
DS                     2
SWH             8012.820 Hz
FIDRES          0.122266 Hz
AQ             4.0894465 sec
RG                126.97
DW                62.400 usec
DE                  6.50 usec
TE                 295.4 K
D1            1.00000000 sec
TD0                    1

======== CHANNEL f1 ========
SFO1         400.1324710 MHz
NUC1                  1H
P1                 14.50 usec
PLW1         11.99499989 W

F2 - Processing parameters
SI                 65536
SF           400.1300119 MHz
WDW                   EM
SSB                    0
LB                  0.30 Hz
GB                     0
PC                  1.00
```

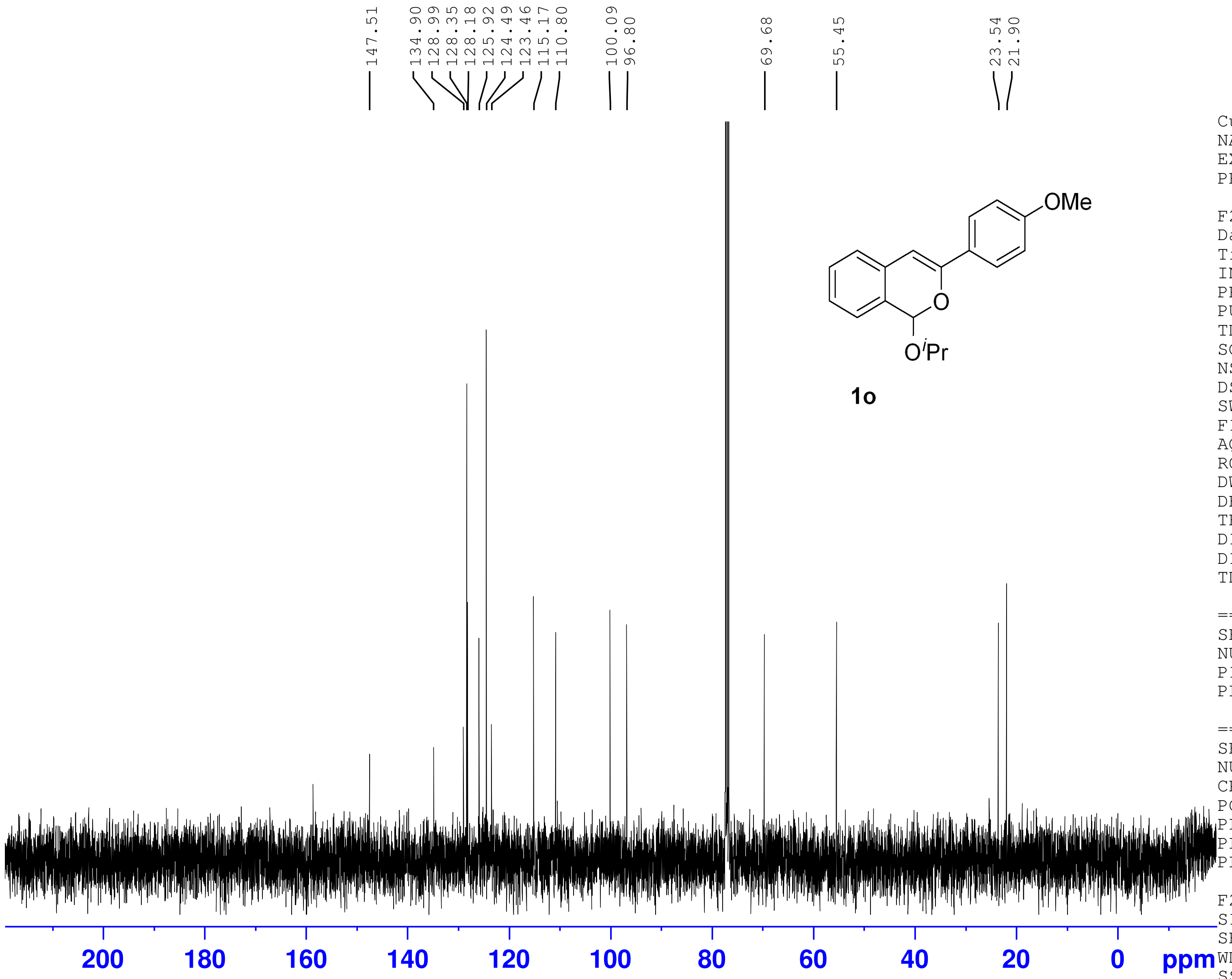

Current Data Parameters
NAME lsx-1-6ome-h
EXPNO 1
PROCNO 1

F2 - Acquisition Parameters
Date_ 20211228
Time 19.48
INSTRUM spect
PROBHD 5 mm PABBO BB/
PULPROG zgpg30
TD 65536
SOLVENT CDCl3
NS 59
DS 2
SWH 24038.461 Hz
FIDRES 0.366798 Hz
AQ 1.3631488 sec
RG 196.92
DW 20.800 usec
DE 6.50 usec
TE 296.0 K
D1 2.00000000 sec
D11 0.03000000 sec
TD0 1

======== CHANNEL f1 ========
SFO1 100.6228298 MHz
NUC1 13C
P1 9.70 usec
PLW1 46.98899841 W

======== CHANNEL f2 ========
SFO2 400.1316005 MHz
NUC2 1H
CPDPRG[2 waltz16
PCPD2 90.00 usec
PLW2 11.99499989 W
PLW12 0.34213999 W
PLW13 0.27713001 W

F2 - Processing parameters
SI 32768
SF 100.6127725 MHz
WDW EM
SSB 0
LB 1.00 Hz
GB 0
PC 1.40

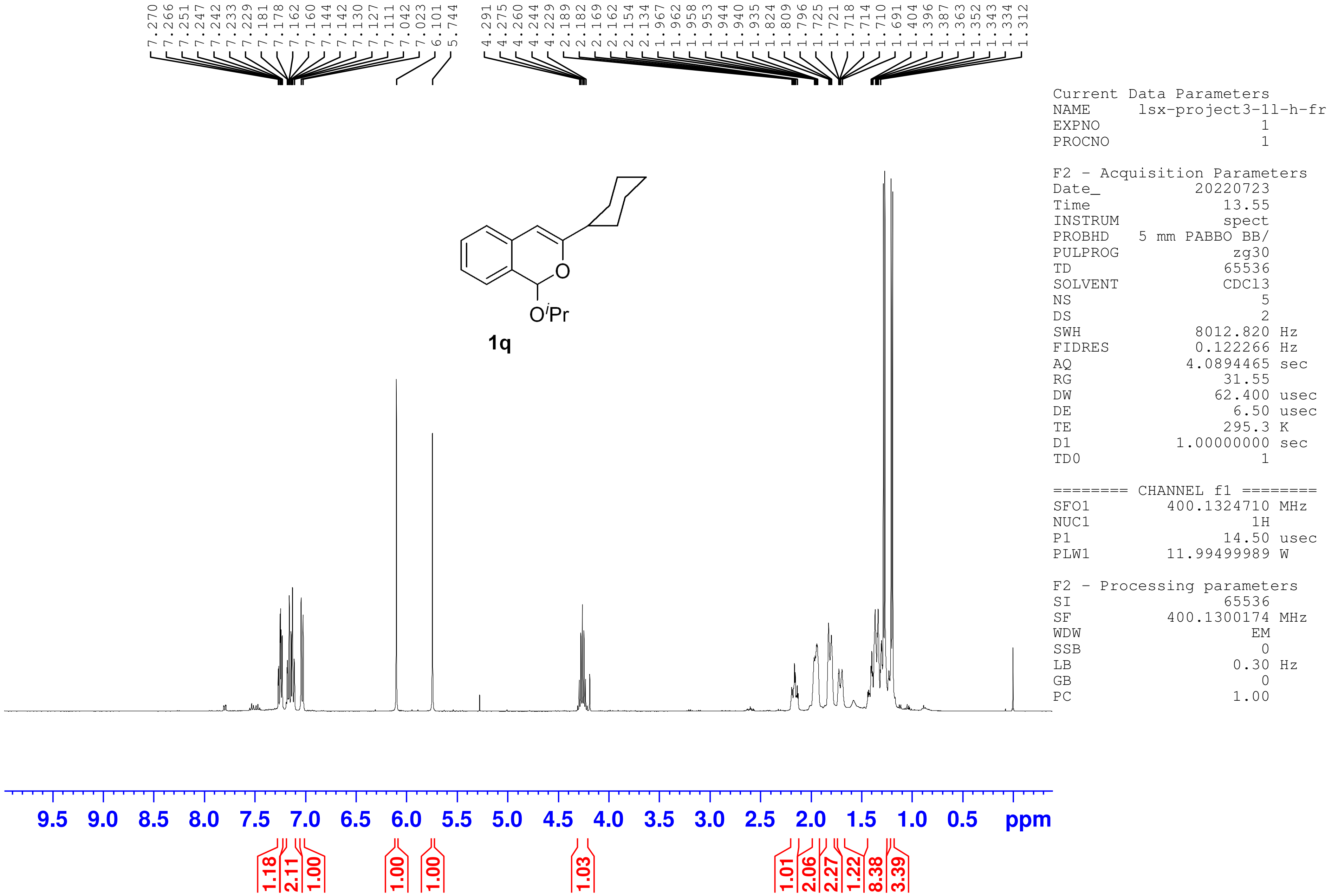
O^{i}Pr
1q
Current Data Parameters
NAME lsx-project3-1l-h-fr
EXPNO 1
PROCNO 1
F2 - Acquisition Parameters
Date_ 20220723
Time 13.55
INSTRUM spect
PROBHD 5 mm PABBO BB/
PULPROG zg30
TD 65536
SOLVENT CDCl3
NS 5
DS 2
SWH 8012.820 Hz
FIDRES 0.122266 Hz
AQ 4.0894465 sec
RG 31.55
DW 62.400 usec
DE 6.50 usec
TE 295.3 K
D1 1.00000000 sec
TD0 1
======== CHANNEL f1 ========
SFO1 400.1324710 MHz
NUC1 1H
P1 14.50 usec
PLW1 11.99499989 W
F2 - Processing parameters
SI 65536
SF 400.1300174 MHz
WDW EM
SSB 0
LB 0.30 Hz
GB 0
PC 1.00
ppm

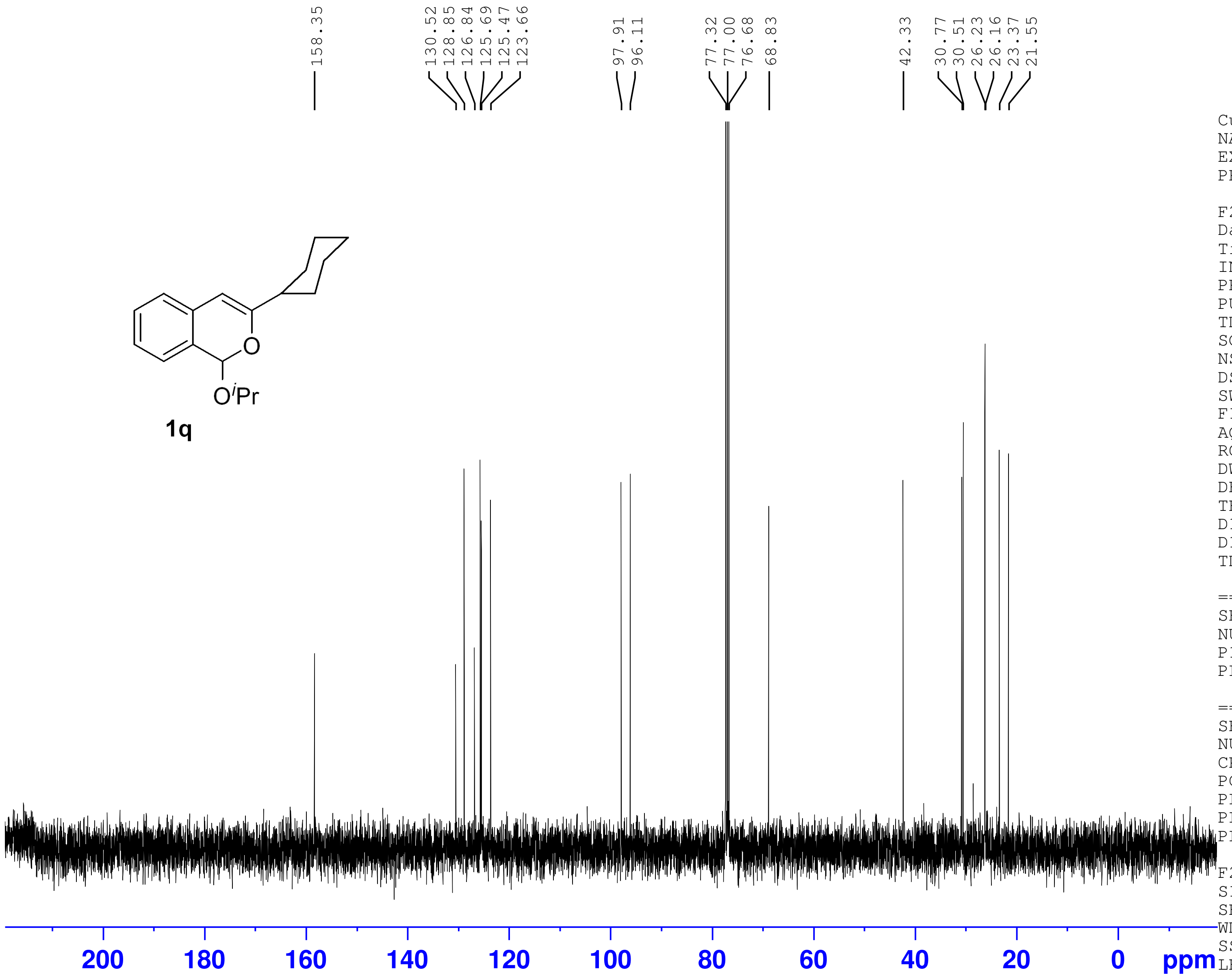


```
Current Data Parameters
NAME     lsx-project3-1l-c-fr
EXPNO                       1
PROCNO                      1

F2 - Acquisition Parameters
Date_            20220723
Time                13.56
INSTRUM             spect
PROBHD    5 mm PABBO BB/
PULPROG           zgpg30
TD                  65536
SOLVENT             CDCl3
NS                     12
DS                      2
SWH             24038.461 Hz
FIDRES           0.366798 Hz
AQ              1.3631488 sec
RG                 196.92
DW                 20.800 usec
DE                   6.50 usec
TE                  295.7 K
D1             2.00000000 sec
D11            0.03000000 sec
TD0                     1

======== CHANNEL f1 ========
SFO1       100.6228298 MHz
NUC1                   13C
P1                    9.70 usec
PLW1           46.98899841 W

======== CHANNEL f2 ========
SFO2       400.1316005 MHz
NUC2                    1H
CPDPRG[2           waltz16
PCPD2                90.00 usec
PLW2           11.99499989 W
PLW12           0.34213999 W
PLW13           0.27713001 W

F2 - Processing parameters
SI                   32768
SF           100.6127753 MHz
WDW                     EM
SSB                      0
LB                    1.00 Hz
GB                       0
PC                    1.40
```

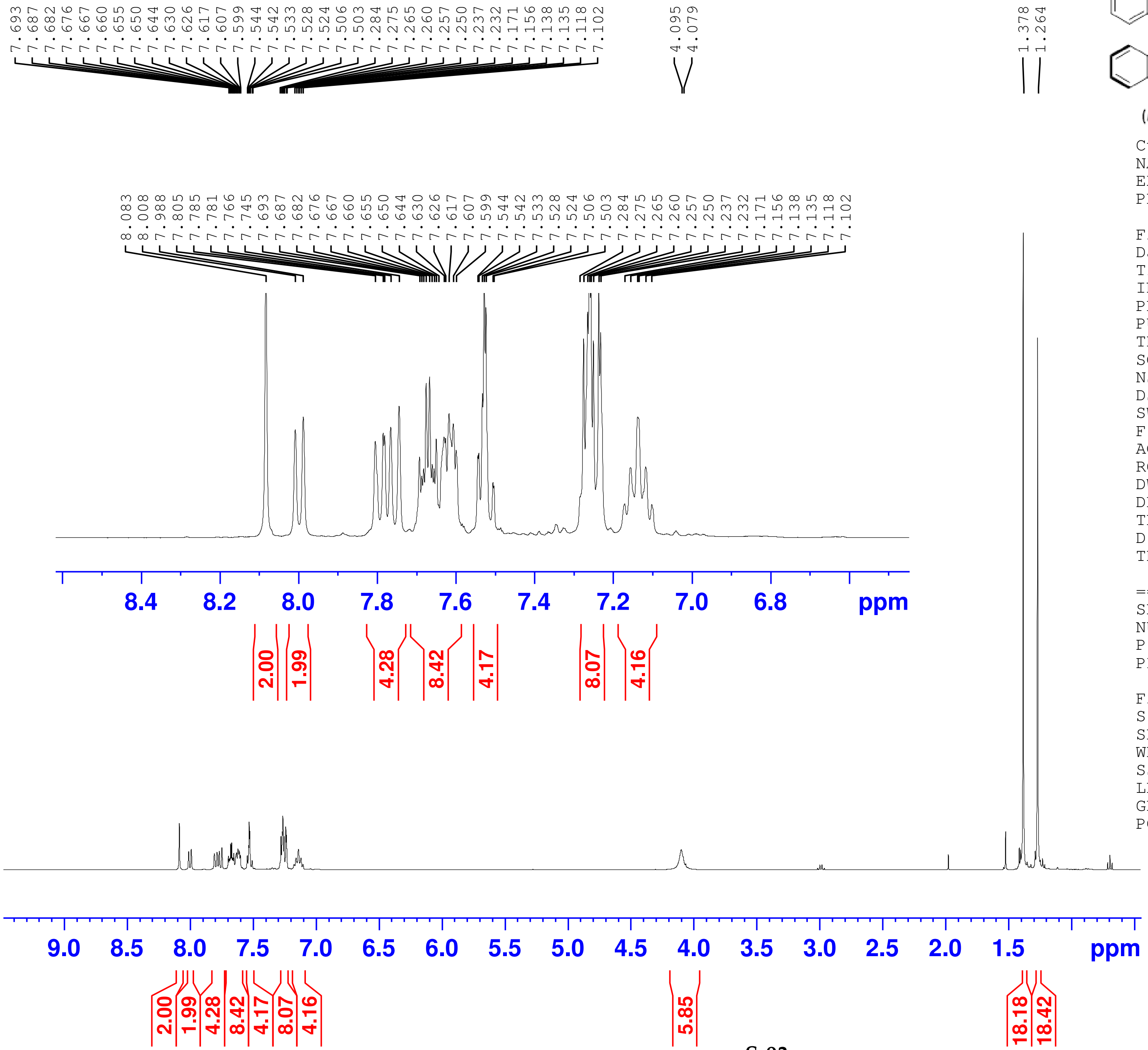

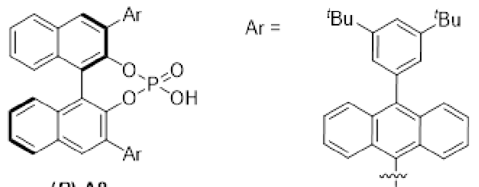

(*R*)-A8

Current Data Parameters
NAME lsx-p3-cat-h-fr
EXPNO 1
PROCNO 1

F2 - Acquisition Parameters
Date_ 20240515
Time 10.05
INSTRUM spect
PROBHD 5 mm PABBO BB/
PULPROG zg30
TD 65536
SOLVENT CDCl3
NS 16
DS 2
SWH 8012.820 Hz
FIDRES 0.122266 Hz
AQ 4.0894465 sec
RG 62.93
DW 62.400 usec
DE 6.50 usec
TE 295.8 K
D1 1.00000000 sec
TD0 1

======== CHANNEL f1 ========
SFO1 400.1324710 MHz
NUC1 1H
P1 14.50 usec
PLW1 11.99499989 W

F2 - Processing parameters
SI 65536
SF 400.1300189 MHz
WDW EM
SSB 0
LB 0.30 Hz
GB 0
PC 1.00

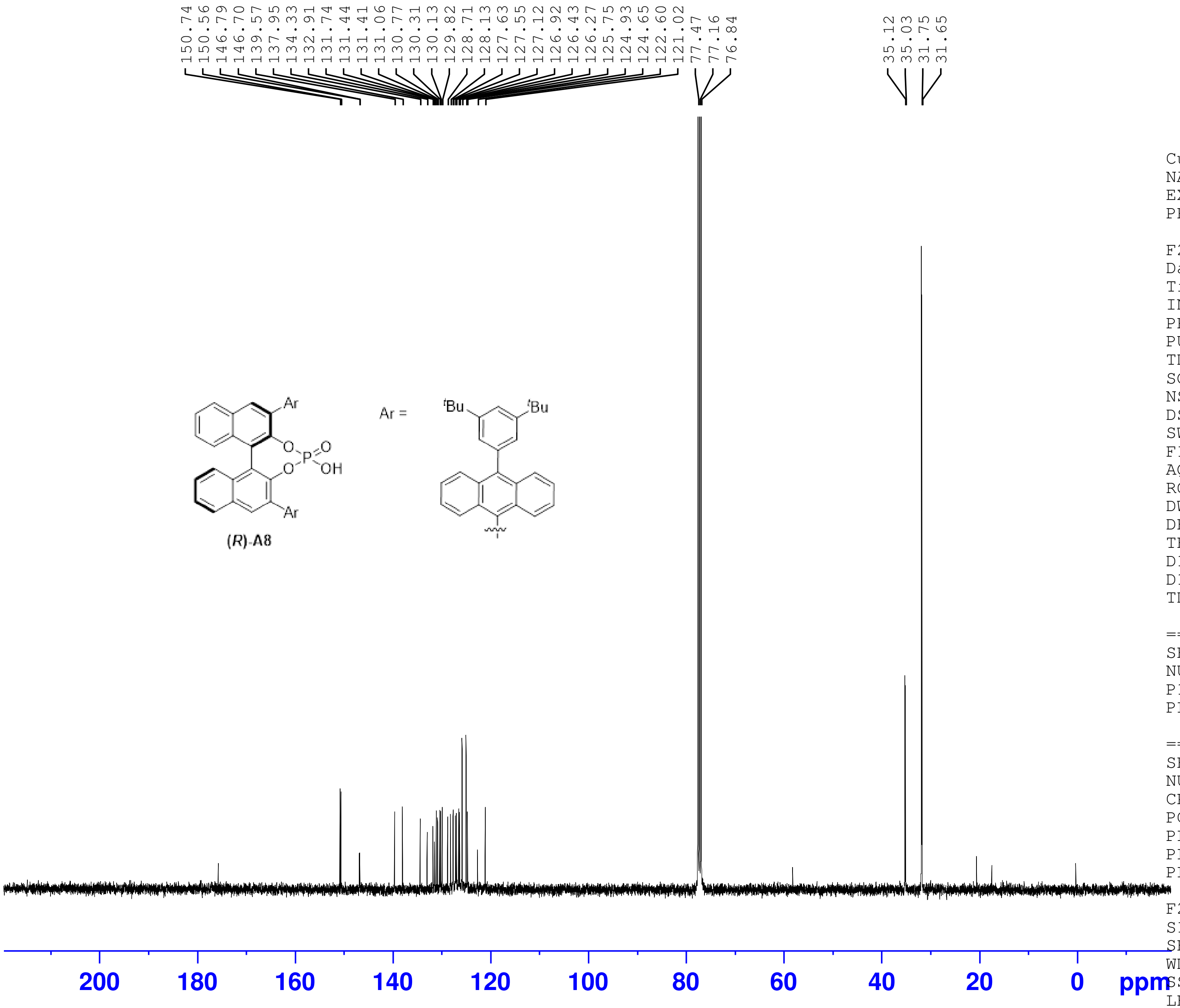


```
Current Data Parameters
NAME      lsx-p3-cat-c-frrrr
EXPNO                    1
PROCNO                   1

F2 - Acquisition Parameters
Date_             20240515
Time                 11.09
INSTRUM              spect
PROBHD     5 mm PABBO BB/
PULPROG            zgpg30
TD                   65536
SOLVENT              CDCl3
NS                    1000
DS                       2
SWH          24038.461 Hz
FIDRES        0.366798 Hz
AQ           1.3631488 sec
RG                  196.92
DW              20.800 usec
DE               6.50 usec
TE               297.0 K
D1          2.00000000 sec
D11         0.03000000 sec
TD0                      1

======== CHANNEL f1 ========
SFO1       100.6228298 MHz
NUC1                   13C
P1               9.70 usec
PLW1        46.98899841 W

======== CHANNEL f2 ========
SFO2       400.1316005 MHz
NUC2                    1H
CPDPRG[2          waltz16
PCPD2           90.00 usec
PLW2        11.99499989 W
PLW12        0.34213999 W
PLW13        0.27713001 W

F2 - Processing parameters
SI                   32768
SF         100.6127574 MHz
WDW                     EM
SSB                      0
LB                 1.00 Hz
GB                       0
PC                    1.40
```

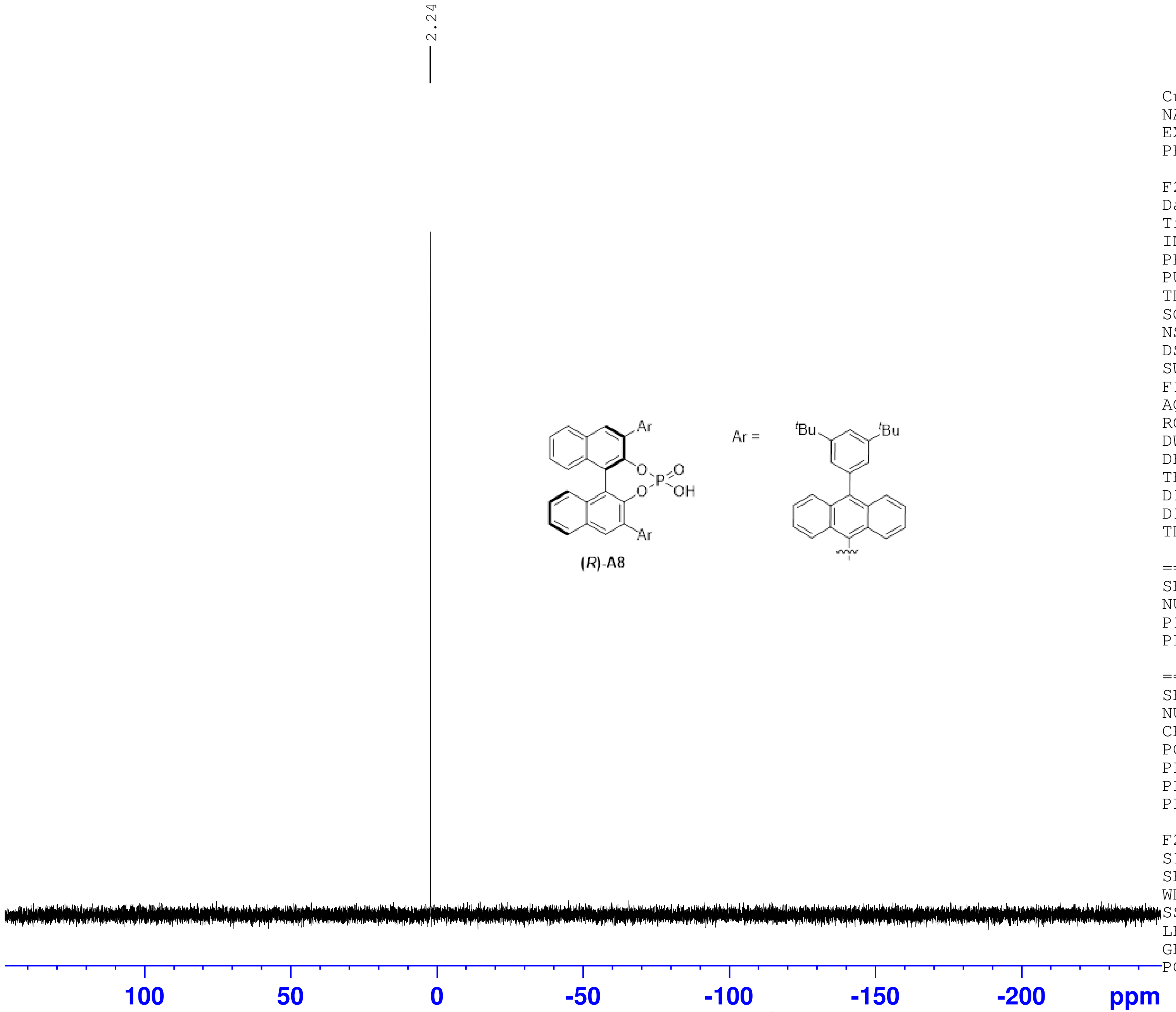

(R)-A8

```
Current Data Parameters
NAME       lsx-p3-cat-p-fr
EXPNO                    1
PROCNO                   1

F2 - Acquisition Parameters
Date_             20240515
Time                 10.07
INSTRUM              spect
PROBHD    5 mm PABBO BB/
PULPROG            zgpg30
TD                   65536
SOLVENT              CDCl3
NS                      17
DS                       4
SWH              64102.562 Hz
FIDRES            0.978127 Hz
AQ               0.5111808 sec
RG                  196.92
DW                   7.800 usec
DE                    6.50 usec
TE                   296.2 K
D1              2.00000000 sec
D11             0.03000000 sec
TD0                      1

======== CHANNEL f1 ========
SFO1           161.9674942 MHz
NUC1                   31P
P1                   14.70 usec
PLW1           11.99499989 W

======== CHANNEL f2 ========
SFO2           400.1316005 MHz
NUC2                    1H
CPDPRG[2          waltz16
PCPD2                90.00 usec
PLW2           11.99499989 W
PLW12           0.34213999 W
PLW13           0.27713001 W

F2 - Processing parameters
SI                   32768
SF             161.9755930 MHz
WDW                     EM
SSB                      0
LB                    1.00 Hz
GB                       0
PC                    1.40
```

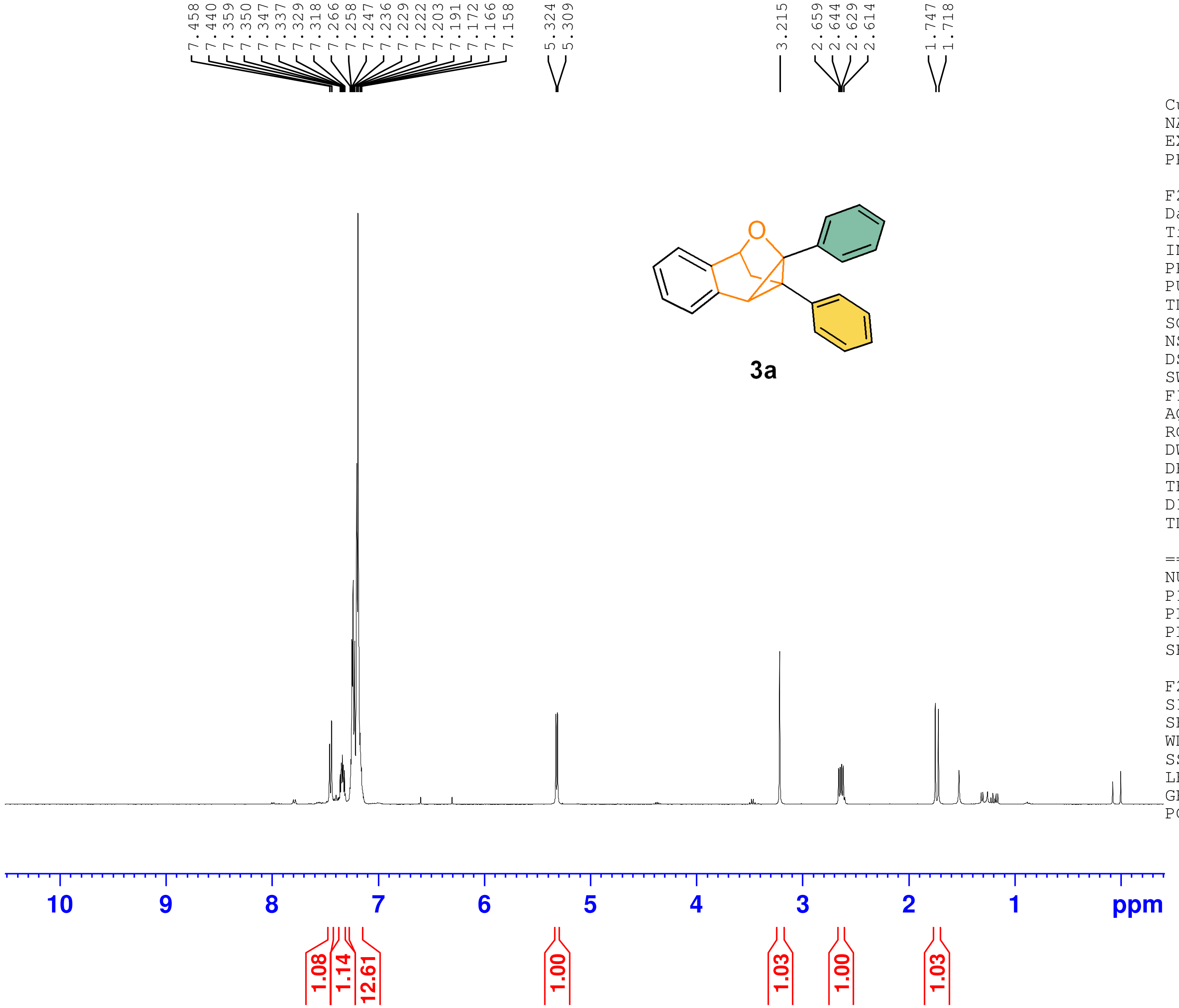

3a

```
Current Data Parameters
NAME        lsx-p3-3a-h-fr
EXPNO                    1
PROCNO                   1

F2 - Acquisition Parameters
Date_             20220726
Time                 17.19
INSTRUM              spect
PROBHD    5 mm DUL 13C-1
PULPROG               zg30
TD                   65536
SOLVENT              CDCl3
NS                       8
DS                       2
SWH               8223.685 Hz
FIDRES            0.125483 Hz
AQ               3.9845889 sec
RG                     228
DW                  60.800 usec
DE                    6.00 usec
TE                   292.8 K
D1              1.00000000 sec
TD0                      1

======== CHANNEL f1 ========
NUC1                    1H
P1                   15.80 usec
PL1                  -1.00 dB
PL1W           12.17476940 W
SFO1           400.1324710 MHz

F2 - Processing parameters
SI                   32768
SF             400.1300235 MHz
WDW                     EM
SSB                      0
LB                    0.30 Hz
GB                       0
PC                    1.00
```

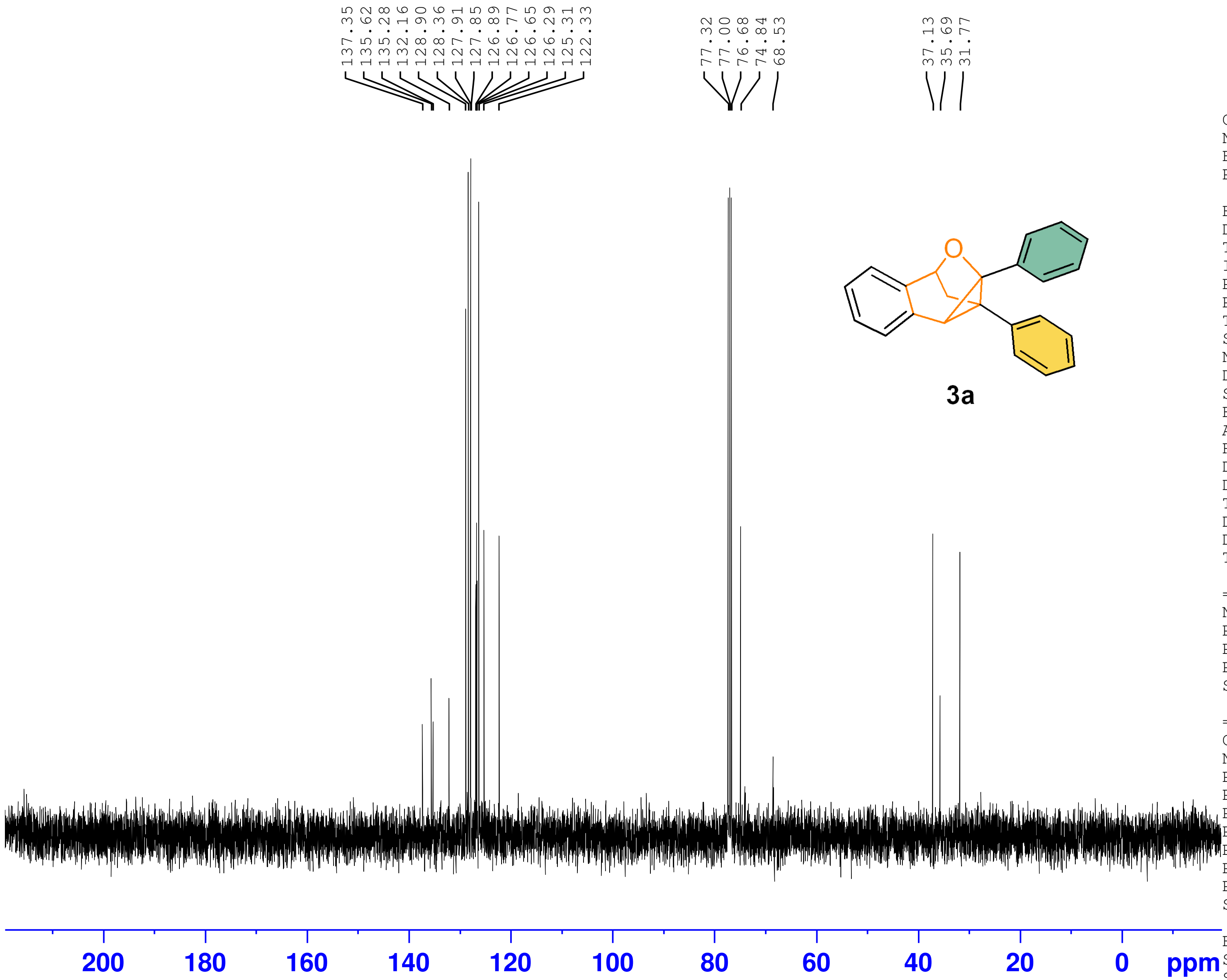

3a

Current Data Parameters
NAME lsx-p3-3a-c-fr
EXPNO 1
PROCNO 1

F2 - Acquisition Parameters
Date_ 20220726
Time 17.21
INSTRUM spect
PROBHD 5 mm DUL 13C-1
PULPROG zgpg30
TD 65536
SOLVENT CDCl3
NS 46
DS 1
SWH 24038.461 Hz
FIDRES 0.366798 Hz
AQ 1.3631488 sec
RG 2050
DW 20.800 usec
DE 6.00 usec
TE 293.1 K
D1 2.00000000 sec
D11 0.03000000 sec
TD0 1

======== CHANNEL f1 ========
NUC1 13C
P1 40.00 usec
PL1 -3.00 dB
PL1W 60.64365387 W
SFO1 100.6228298 MHz

======== CHANNEL f2 ========
CPDPRG[2 waltz16
NUC2 1H
PCPD2 80.00 usec
PL2 -1.00 dB
PL12 14.39 dB
PL13 18.00 dB
PL2W 12.17476940 W
PL12W 0.35193357 W
PL13W 0.15327126 W
SFO2 400.1316005 MHz

F2 - Processing parameters
SI 32768
SF 100.6127769 MHz
WDW EM
SSB 0
LB 1.00 Hz
GB 0
PC 1.40

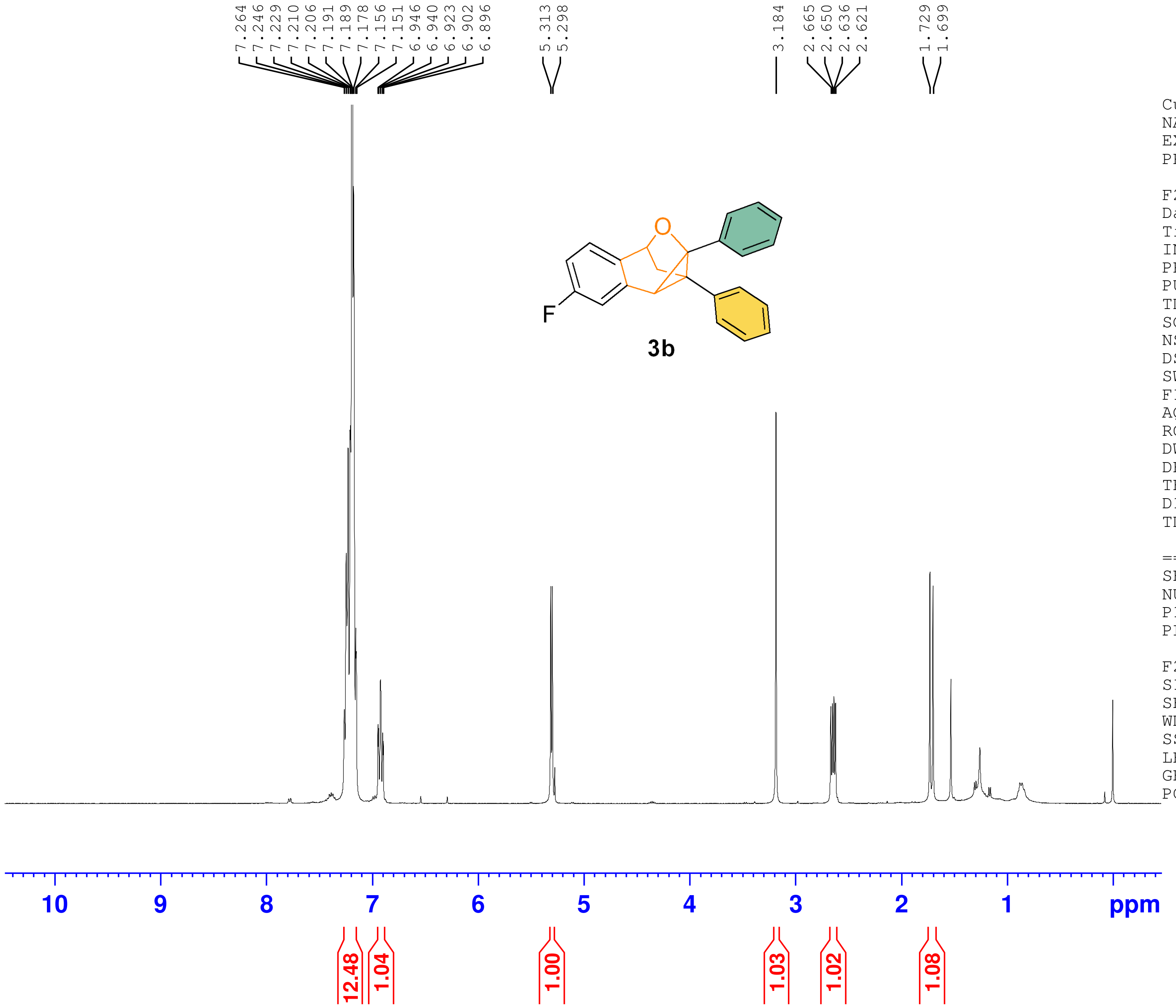


```
Current Data Parameters
NAME      lsx-1-174-h-fr
EXPNO                  1
PROCNO                 1

F2 - Acquisition Parameters
Date_           20220104
Time               15.40
INSTRUM            spect
PROBHD   5 mm PABBO BB/
PULPROG             zg30
TD                 65536
SOLVENT            CDCl3
NS                    16
DS                     2
SWH             8012.820 Hz
FIDRES          0.122266 Hz
AQ             4.0894465 sec
RG                164.33
DW                62.400 usec
DE                  6.50 usec
TE                 295.3 K
D1            1.00000000 sec
TD0                    1

======== CHANNEL f1 ========
SFO1         400.1324710 MHz
NUC1                  1H
P1                 14.50 usec
PLW1         11.99499989 W

F2 - Processing parameters
SI                 65536
SF           400.1300192 MHz
WDW                   EM
SSB                    0
LB                  0.30 Hz
GB                     0
PC                  1.00
```

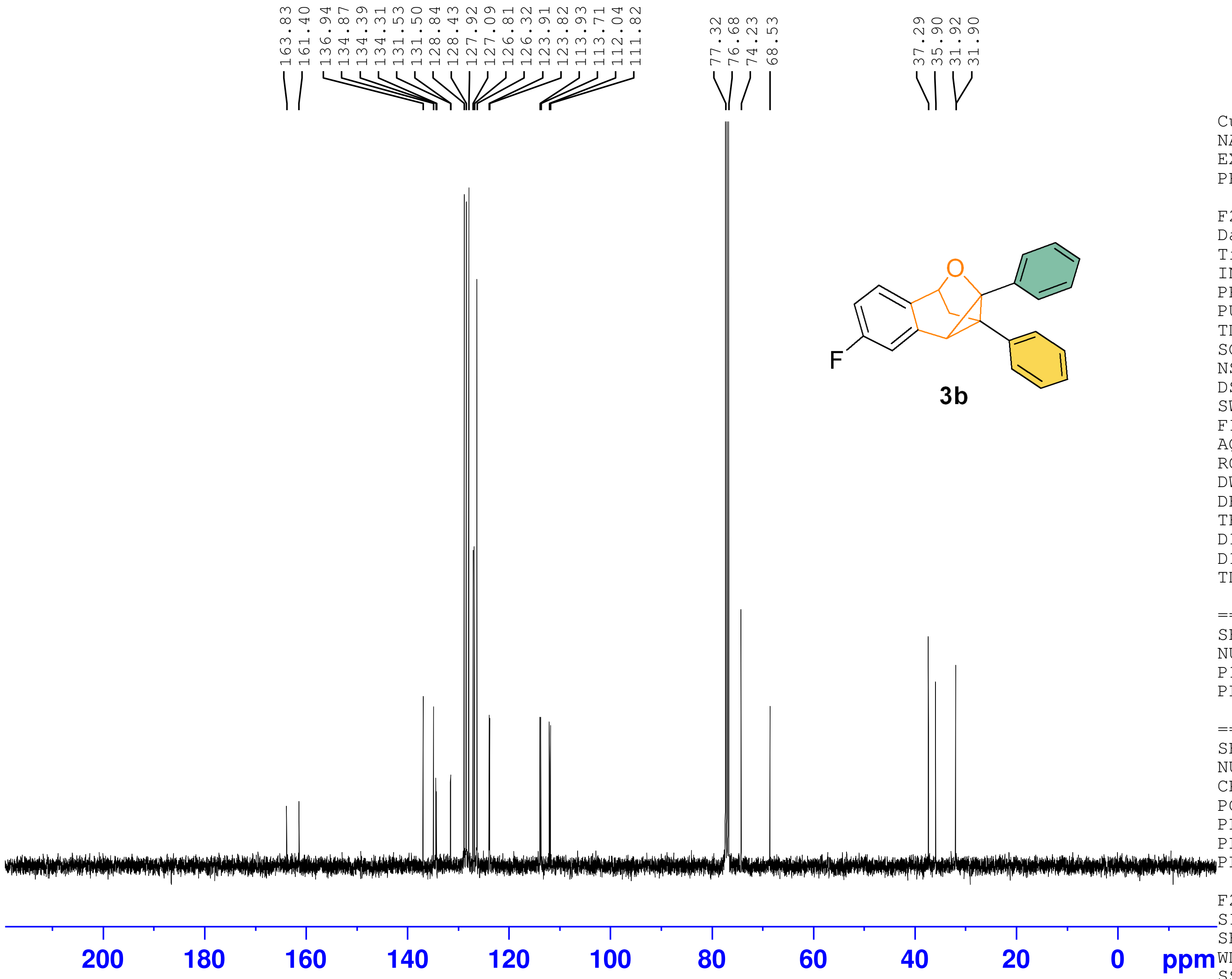


```
Current Data Parameters
NAME       lsx-1-174-c-fr
EXPNO                   1
PROCNO                  1

F2 - Acquisition Parameters
Date_            20220104
Time                15.53
INSTRUM             spect
PROBHD   5 mm PABBO BB/
PULPROG           zgpg30
TD                  65536
SOLVENT             CDCl3
NS                    209
DS                      2
SWH             24038.461 Hz
FIDRES           0.366798 Hz
AQ              1.3631488 sec
RG                 196.92
DW                 20.800 usec
DE                   6.50 usec
TE                  296.0 K
D1             2.00000000 sec
D11            0.03000000 sec
TD0                     1

======== CHANNEL f1 ========
SFO1          100.6228298 MHz
NUC1                  13C
P1                   9.70 usec
PLW1          46.98899841 W

======== CHANNEL f2 ========
SFO2          400.1316005 MHz
NUC2                   1H
CPDPRG[2          waltz16
PCPD2               90.00 usec
PLW2          11.99499989 W
PLW12          0.34213999 W
PLW13          0.27713001 W

F2 - Processing parameters
SI                  32768
SF            100.6127748 MHz
WDW                    EM
SSB                     0
LB                   1.00 Hz
GB                      0
PC                   1.40
```

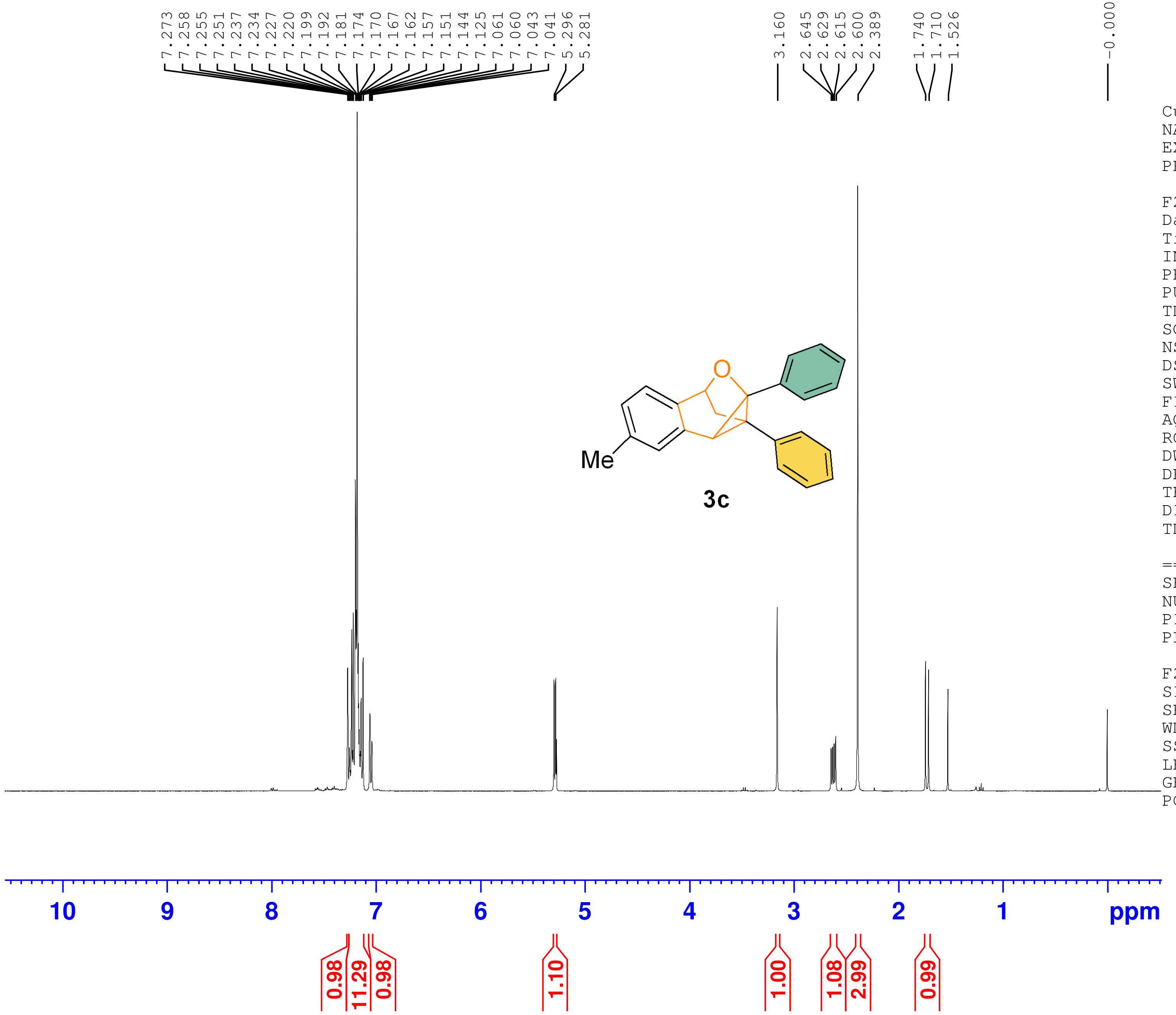


```
Current Data Parameters
NAME              lsx-2-9c-h
EXPNO                      1
PROCNO                     1

F2 - Acquisition Parameters
Date_               20220122
Time                   12.25
INSTRUM                spect
PROBHD    5 mm PABBO BB/
PULPROG                 zg30
TD                     65536
SOLVENT                CDCl3
NS                         7
DS                         2
SWH                 8012.820 Hz
FIDRES              0.122266 Hz
AQ                 4.0894465 sec
RG                     82.92
DW                    62.400 usec
DE                      6.50 usec
TE                     295.1 K
D1                1.00000000 sec
TD0                        1

======== CHANNEL f1 ========
SFO1             400.1324710 MHz
NUC1                      1H
P1                     14.50 usec
PLW1             11.99499989 W

F2 - Processing parameters
SI                     65536
SF               400.1300205 MHz
WDW                       EM
SSB                        0
LB                      0.30 Hz
GB                         0
PC                      1.00
```

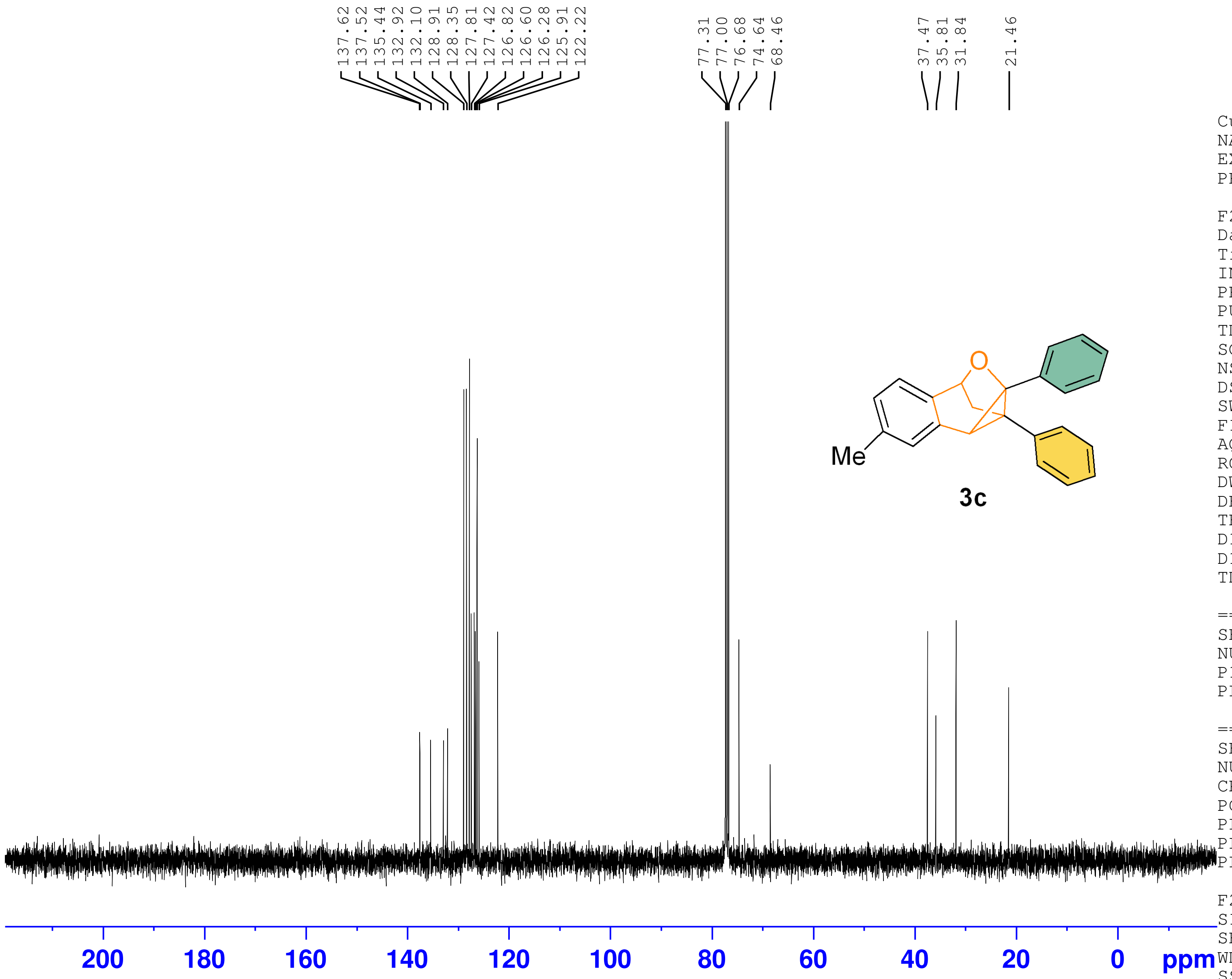


```
Current Data Parameters
NAME              lsx-2-9c-c
EXPNO                      1
PROCNO                     1

F2 - Acquisition Parameters
Date_               20220122
Time                   12.28
INSTRUM                spect
PROBHD   5 mm PABBO BB/
PULPROG              zgpg30
TD                     65536
SOLVENT                CDCl3
NS                        40
DS                         2
SWH                24038.461 Hz
FIDRES              0.366798 Hz
AQ                 1.3631488 sec
RG                    196.92
DW                    20.800 usec
DE                      6.50 usec
TE                     295.9 K
D1                2.00000000 sec
D11               0.03000000 sec
TD0                        1

======== CHANNEL f1 ========
SFO1             100.6228298 MHz
NUC1                     13C
P1                      9.70 usec
PLW1             46.98899841 W

======== CHANNEL f2 ========
SFO2             400.1316005 MHz
NUC2                      1H
CPDPRG[2             waltz16
PCPD2                  90.00 usec
PLW2             11.99499989 W
PLW12             0.34213999 W
PLW13             0.27713001 W

F2 - Processing parameters
SI                     32768
SF               100.6127754 MHz
WDW                       EM
SSB                        0
LB                      1.00 Hz
GB                         0
PC                      1.40
```

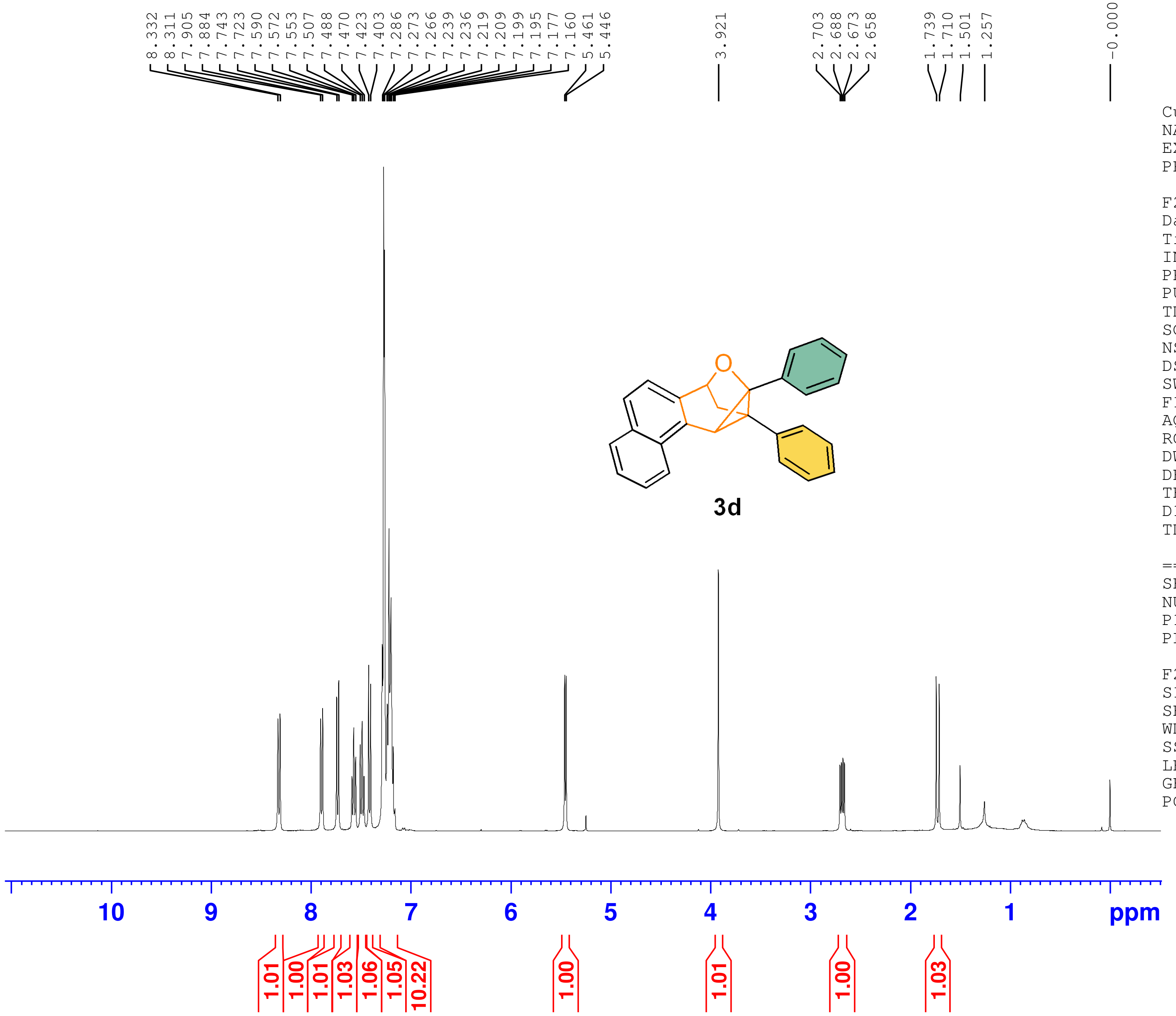

3d

```
Current Data Parameters
NAME        lsx-1-176-h-fr
EXPNO                    1
PROCNO                   1

F2 - Acquisition Parameters
Date_             20220104
Time                 15.59
INSTRUM              spect
PROBHD    5 mm PABBO BB/
PULPROG               zg30
TD                   65536
SOLVENT              CDCl3
NS                      16
DS                       2
SWH               8012.820 Hz
FIDRES            0.122266 Hz
AQ               4.0894465 sec
RG                  112.31
DW                  62.400 usec
DE                    6.50 usec
TE                   295.3 K
D1              1.00000000 sec
TD0                      1

======== CHANNEL f1 ========
SFO1           400.1324710 MHz
NUC1                    1H
P1                   14.50 usec
PLW1           11.99499989 W

F2 - Processing parameters
SI                   65536
SF             400.1300303 MHz
WDW                     EM
SSB                      0
LB                    0.30 Hz
GB                       0
PC                    1.00
```

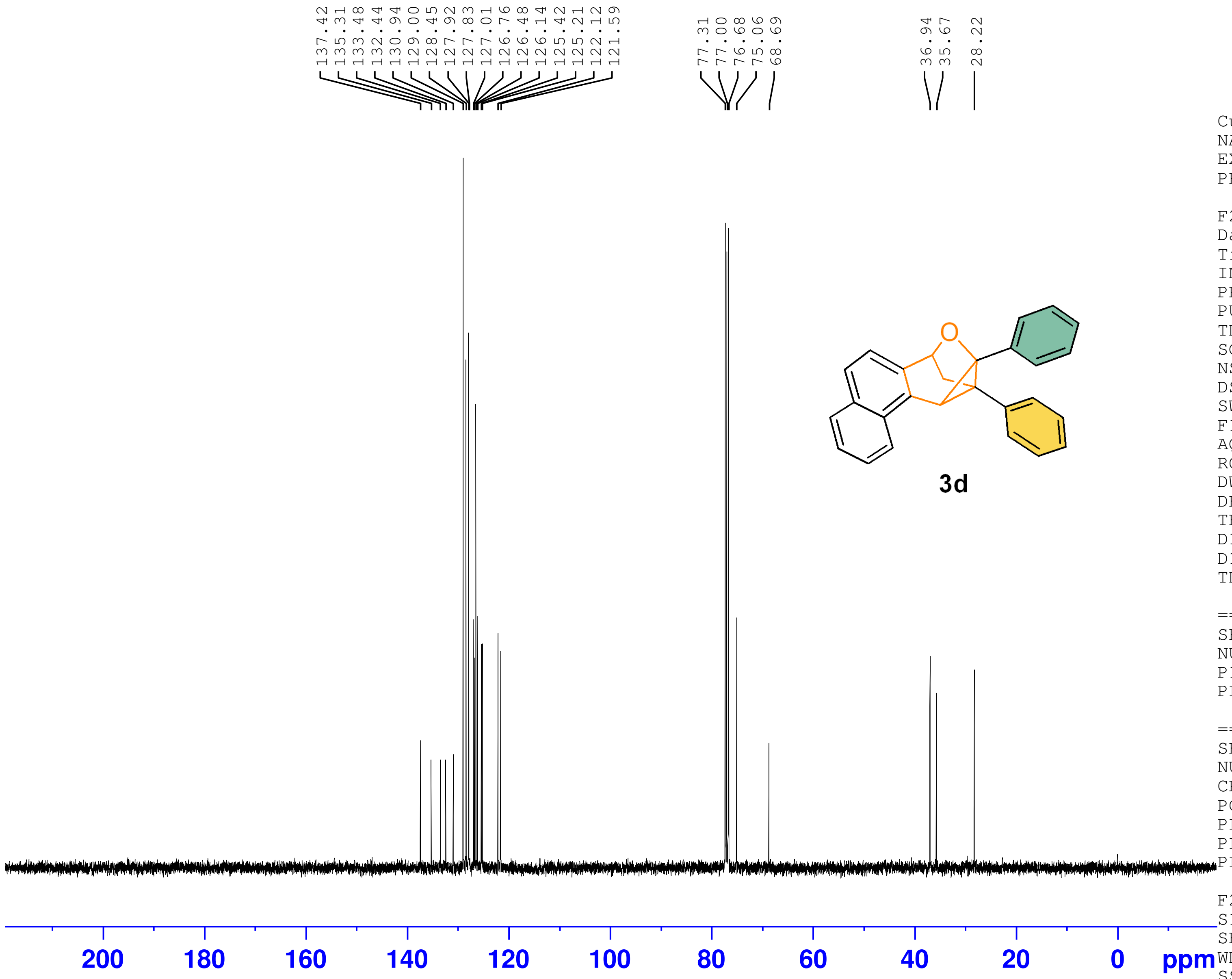


```
Current Data Parameters
NAME      lsx-1-176-c-fr
EXPNO                  1
PROCNO                 1

F2 - Acquisition Parameters
Date_           20220104
Time               16.07
INSTRUM            spect
PROBHD    5 mm PABBO BB/
PULPROG          zgpg30
TD                 65536
SOLVENT            CDCl3
NS                   118
DS                     2
SWH            24038.461 Hz
FIDRES          0.366798 Hz
AQ             1.3631488 sec
RG                196.92
DW                20.800 usec
DE                  6.50 usec
TE                 296.4 K
D1            2.00000000 sec
D11           0.03000000 sec
TD0                    1

======== CHANNEL f1 ========
SFO1         100.6228298 MHz
NUC1                 13C
P1                  9.70 usec
PLW1         46.98899841 W

======== CHANNEL f2 ========
SFO2         400.1316005 MHz
NUC2                  1H
CPDPRG[2         waltz16
PCPD2              90.00 usec
PLW2         11.99499989 W
PLW12         0.34213999 W
PLW13         0.27713001 W

F2 - Processing parameters
SI                 32768
SF           100.6127780 MHz
WDW                   EM
SSB                    0
LB                  1.00 Hz
GB                     0
PC                  1.40
```

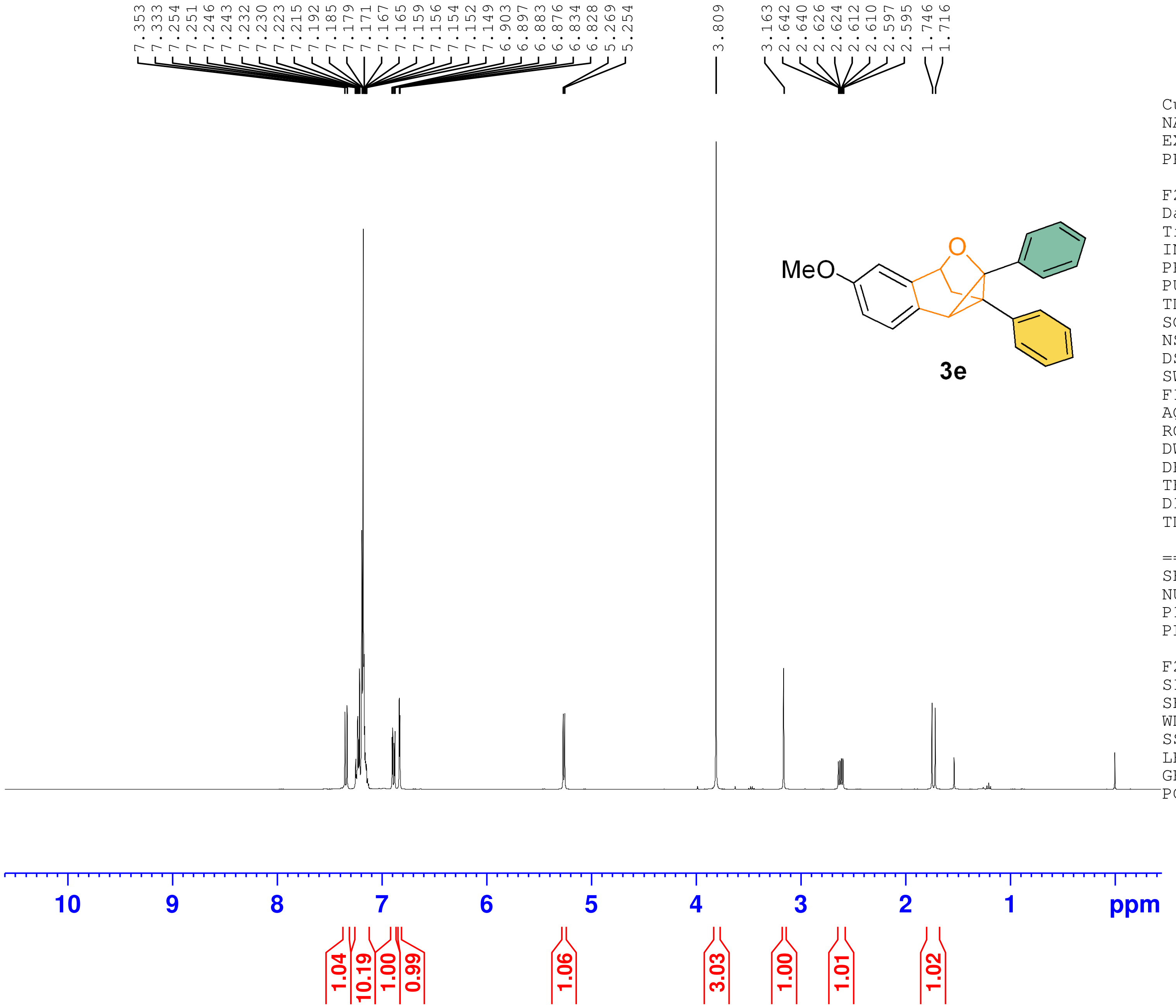

3e

```
Current Data Parameters
NAME            lsx-2-9d-h
EXPNO                    1
PROCNO                   1

F2 - Acquisition Parameters
Date_             20220122
Time                 12.31
INSTRUM              spect
PROBHD    5 mm PABBO BB/
PULPROG               zg30
TD                   65536
SOLVENT              CDCl3
NS                       7
DS                       2
SWH               8012.820 Hz
FIDRES            0.122266 Hz
AQ               4.0894465 sec
RG                   54.81
DW                  62.400 usec
DE                    6.50 usec
TE                   295.1 K
D1              1.00000000 sec
TD0                      1

======== CHANNEL f1 ========
SFO1           400.1324710 MHz
NUC1                    1H
P1                   14.50 usec
PLW1           11.99499989 W

F2 - Processing parameters
SI                   65536
SF             400.1300246 MHz
WDW                     EM
SSB                      0
LB                    0.30 Hz
GB                       0
PC                    1.00
```

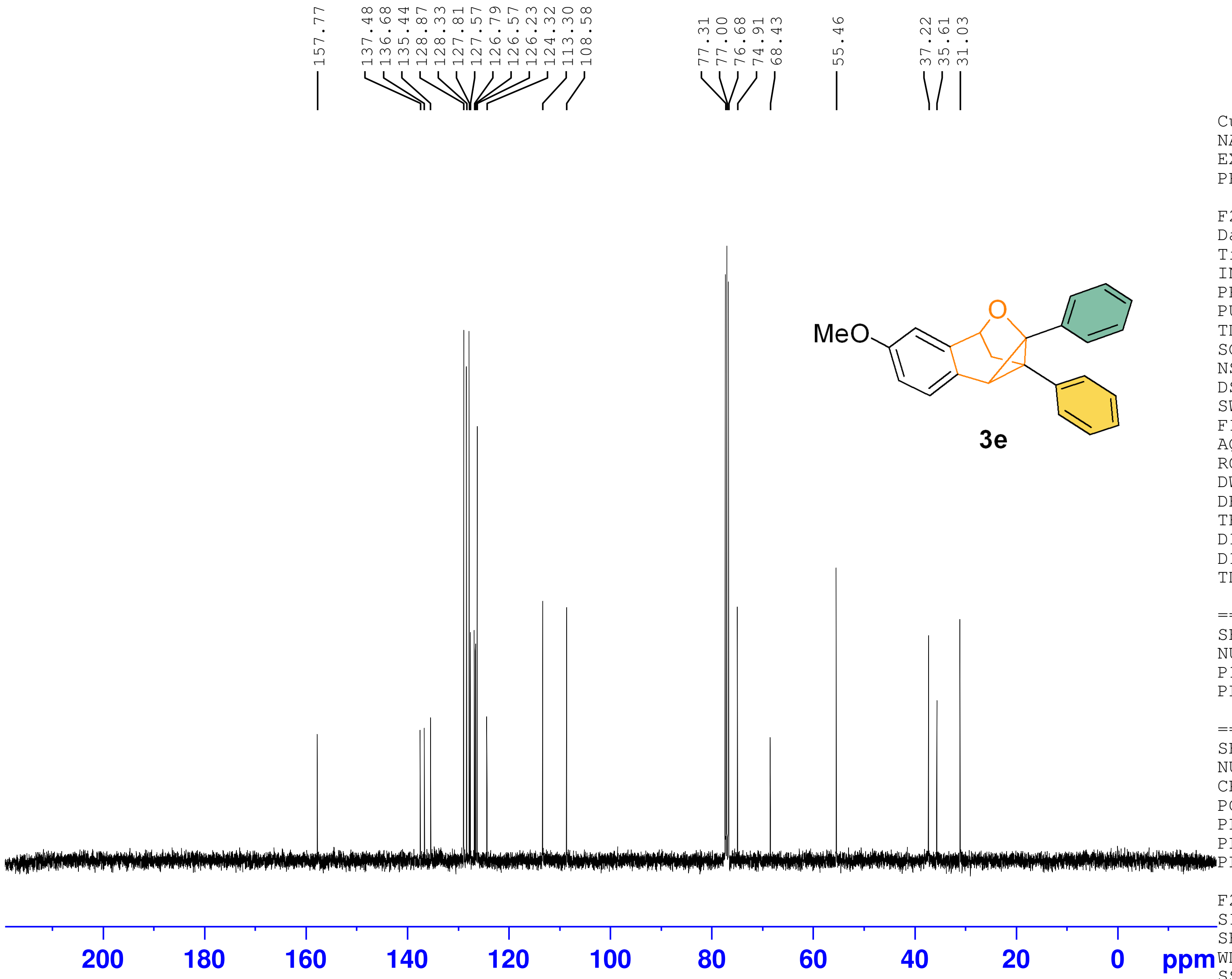


```
Current Data Parameters
NAME          lsx-2-9d-c
EXPNO                  1
PROCNO                 1

F2 - Acquisition Parameters
Date_           20220122
Time               12.35
INSTRUM            spect
PROBHD   5 mm PABBO BB/
PULPROG          zgpg30
TD                65536
SOLVENT            CDCl3
NS                    59
DS                     2
SWH            24038.461 Hz
FIDRES          0.366798 Hz
AQ             1.3631488 sec
RG                196.92
DW                20.800 usec
DE                  6.50 usec
TE                 296.0 K
D1            2.00000000 sec
D11           0.03000000 sec
TD0                    1

======== CHANNEL f1 ========
SFO1         100.6228298 MHz
NUC1                 13C
P1                  9.70 usec
PLW1         46.98899841 W

======== CHANNEL f2 ========
SFO2         400.1316005 MHz
NUC2                  1H
CPDPRG[2         waltz16
PCPD2              90.00 usec
PLW2         11.99499989 W
PLW12         0.34213999 W
PLW13         0.27713001 W

F2 - Processing parameters
SI                 32768
SF           100.6127777 MHz
WDW                   EM
SSB                    0
LB                  1.00 Hz
GB                     0
PC                  1.40
```

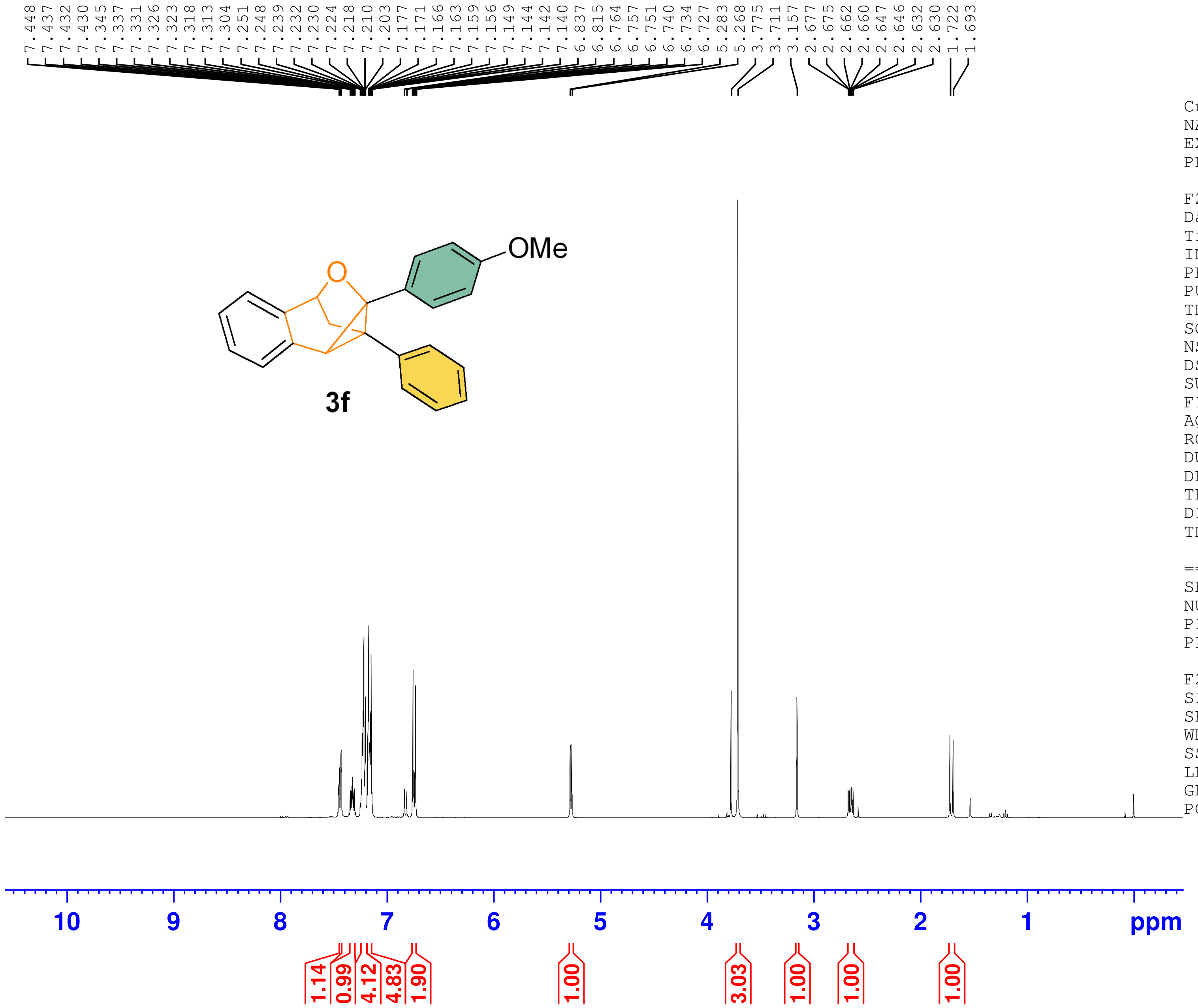


```
Current Data Parameters
NAME              lsx-2-9b-h
EXPNO                      1
PROCNO                     1

F2 - Acquisition Parameters
Date_               20220122
Time                   12.21
INSTRUM                spect
PROBHD     5 mm PABBO BB/
PULPROG                 zg30
TD                     65536
SOLVENT                CDCl3
NS                         9
DS                         2
SWH                 8012.820 Hz
FIDRES              0.122266 Hz
AQ                 4.0894465 sec
RG                     27.78
DW                    62.400 usec
DE                      6.50 usec
TE                     295.2 K
D1                1.00000000 sec
TD0                        1

======== CHANNEL f1 ========
SFO1             400.1324710 MHz
NUC1                      1H
P1                     14.50 usec
PLW1             11.99499989 W

F2 - Processing parameters
SI                     65536
SF               400.1300322 MHz
WDW                       EM
SSB                        0
LB                      0.30 Hz
GB                         0
PC                      1.00
```

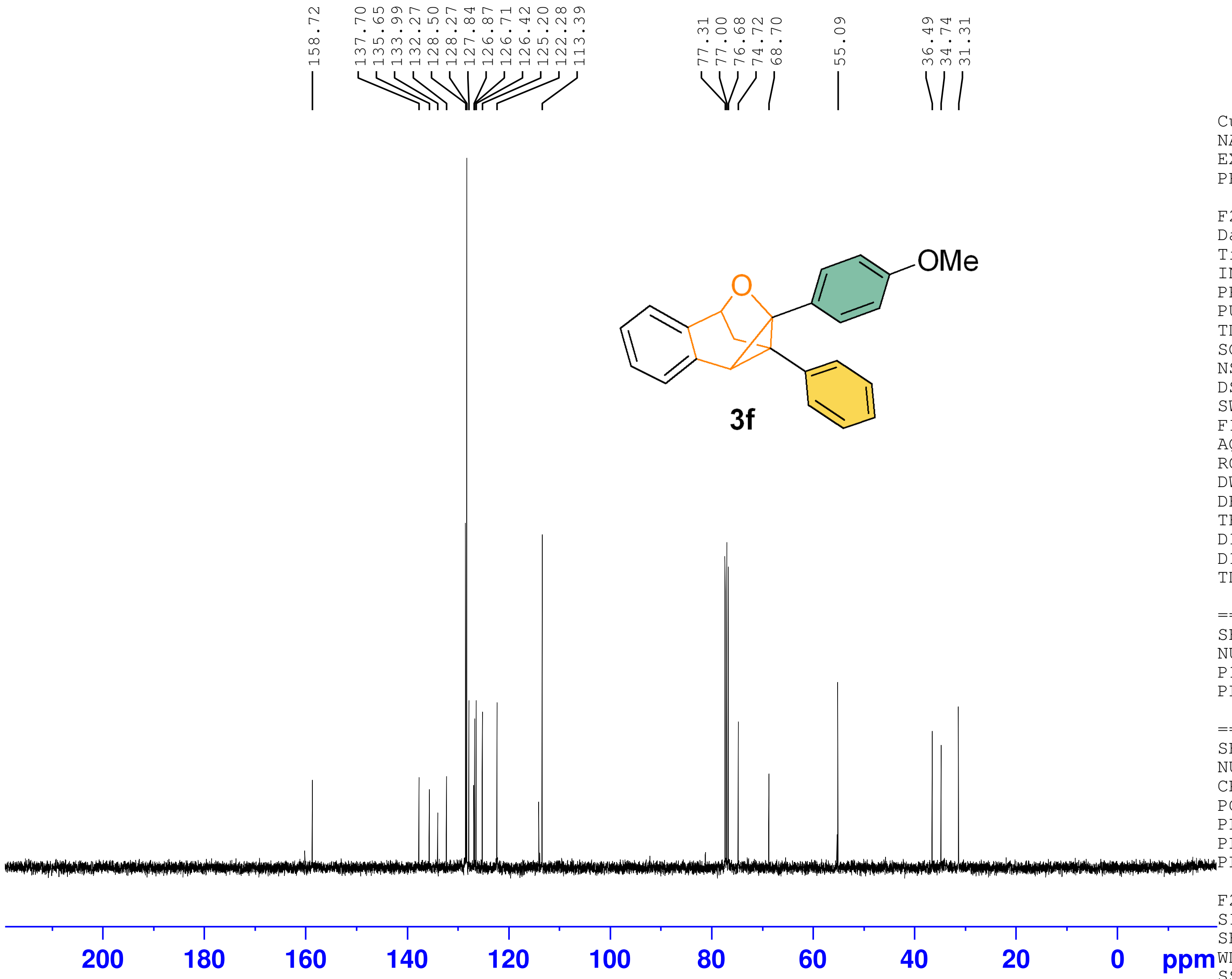


```
Current Data Parameters
NAME            lsx-2-9b-c
EXPNO                    1
PROCNO                   1

F2 - Acquisition Parameters
Date_             20220122
Time                 12.22
INSTRUM              spect
PROBHD    5 mm PABBO BB/
PULPROG            zgpg30
TD                   65536
SOLVENT              CDCl3
NS                      21
DS                       2
SWH              24038.461 Hz
FIDRES            0.366798 Hz
AQ               1.3631488 sec
RG                  196.92
DW                  20.800 usec
DE                    6.50 usec
TE                   295.7 K
D1              2.00000000 sec
D11             0.03000000 sec
TD0                      1

======== CHANNEL f1 ========
SFO1           100.6228298 MHz
NUC1                   13C
P1                    9.70 usec
PLW1           46.98899841 W

======== CHANNEL f2 ========
SFO2           400.1316005 MHz
NUC2                    1H
CPDPRG[2           waltz16
PCPD2                90.00 usec
PLW2           11.99499989 W
PLW12           0.34213999 W
PLW13           0.27713001 W

F2 - Processing parameters
SI                   32768
SF             100.6127814 MHz
WDW                     EM
SSB                      0
LB                    1.00 Hz
GB                       0
PC                    1.40
```

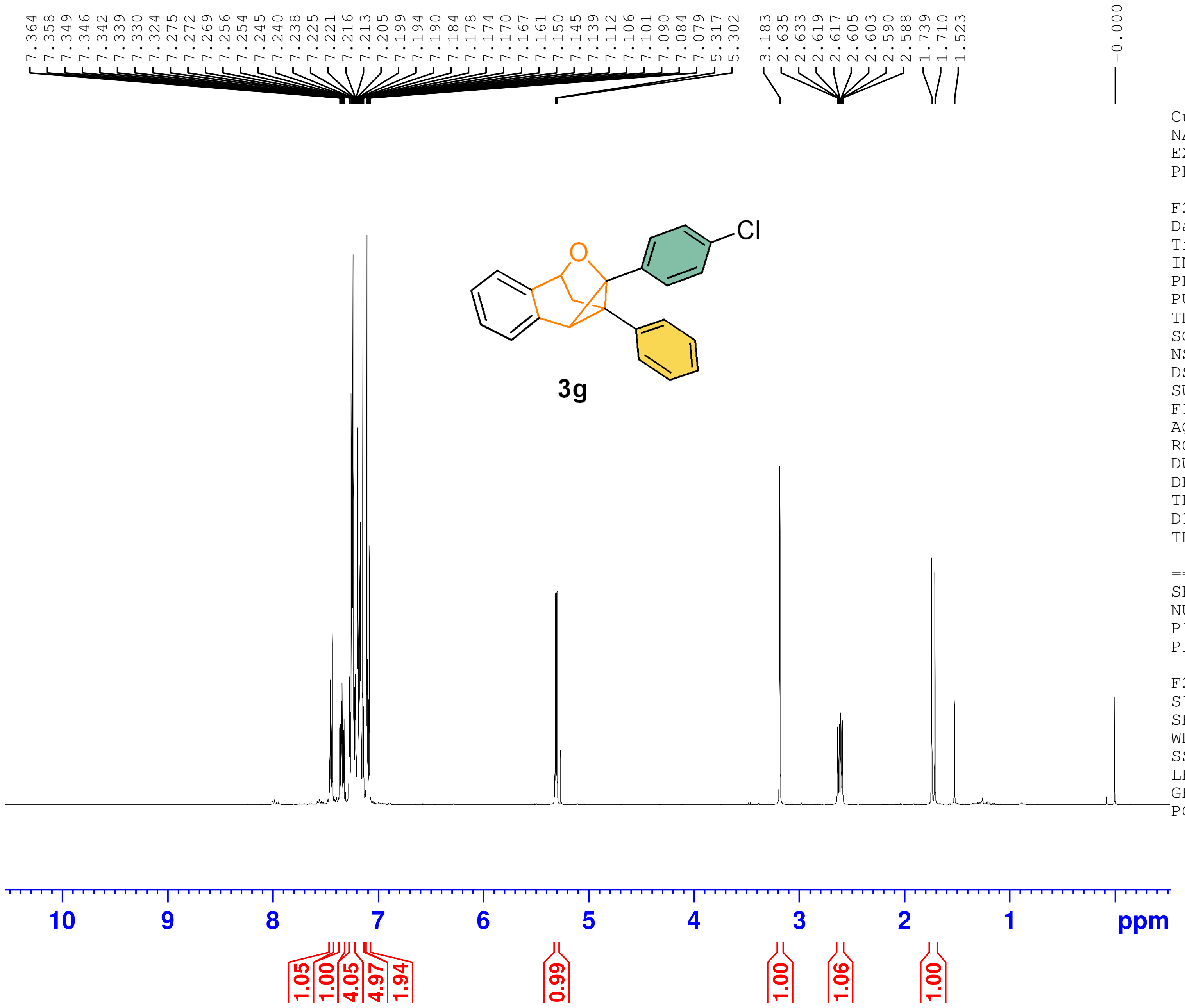


```
Current Data Parameters
NAME              lsx-2-9a-h
EXPNO                      1
PROCNO                     1

F2 - Acquisition Parameters
Date_               20220122
Time                   12.16
INSTRUM                spect
PROBHD     5 mm PABBO BB/
PULPROG                 zg30
TD                     65536
SOLVENT                CDCl3
NS                         6
DS                         2
SWH                 8012.820 Hz
FIDRES              0.122266 Hz
AQ                 4.0894465 sec
RG                     62.93
DW                    62.400 usec
DE                      6.50 usec
TE                     295.2 K
D1                1.00000000 sec
TD0                        1

======== CHANNEL f1 ========
SFO1             400.1324710 MHz
NUC1                      1H
P1                     14.50 usec
PLW1             11.99499989 W

F2 - Processing parameters
SI                     65536
SF               400.1300239 MHz
WDW                       EM
SSB                        0
LB                      0.30 Hz
GB                         0
PC                      1.00
```

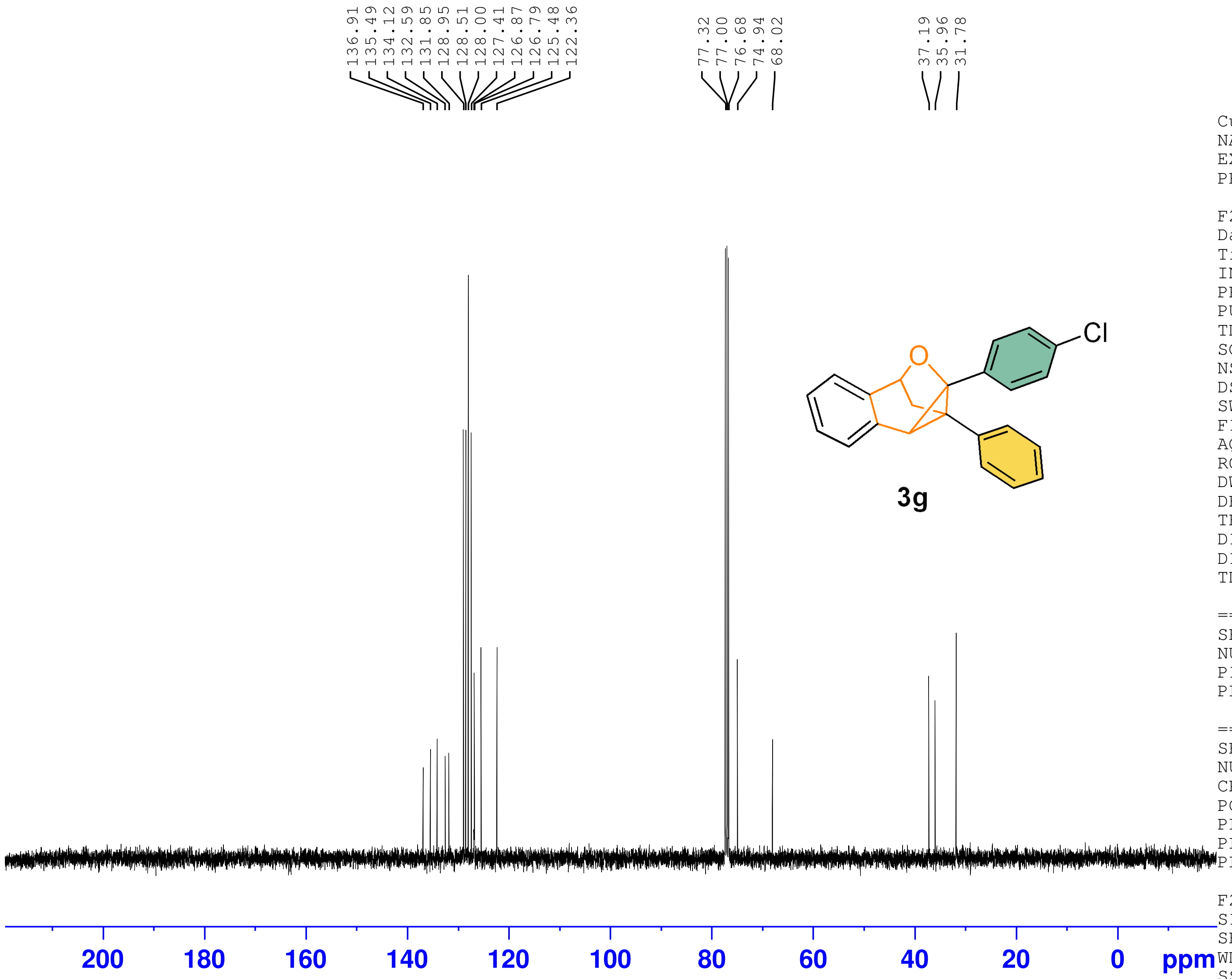


```
Current Data Parameters
NAME             lsx-2-9a-c
EXPNO                     1
PROCNO                    1

F2 - Acquisition Parameters
Date_              20220122
Time                  12.18
INSTRUM               spect
PROBHD   5 mm PABBO BB/
PULPROG             zgpg30
TD                    65536
SOLVENT               CDCl3
NS                       30
DS                        2
SWH            24038.461 Hz
FIDRES          0.366798 Hz
AQ             1.3631488 sec
RG                   196.92
DW                20.800 usec
DE                  6.50 usec
TE                  295.9 K
D1            2.00000000 sec
D11           0.03000000 sec
TD0                       1

======== CHANNEL f1 ========
SFO1        100.6228298 MHz
NUC1                    13C
P1                 9.70 usec
PLW1         46.98899841 W

======== CHANNEL f2 ========
SFO2        400.1316005 MHz
NUC2                     1H
CPDPRG[2            waltz16
PCPD2             90.00 usec
PLW2         11.99499989 W
PLW12         0.34213999 W
PLW13         0.27713001 W

F2 - Processing parameters
SI                    32768
SF          100.6127770 MHz
WDW                      EM
SSB                       0
LB                  1.00 Hz
GB                        0
PC                     1.40
```

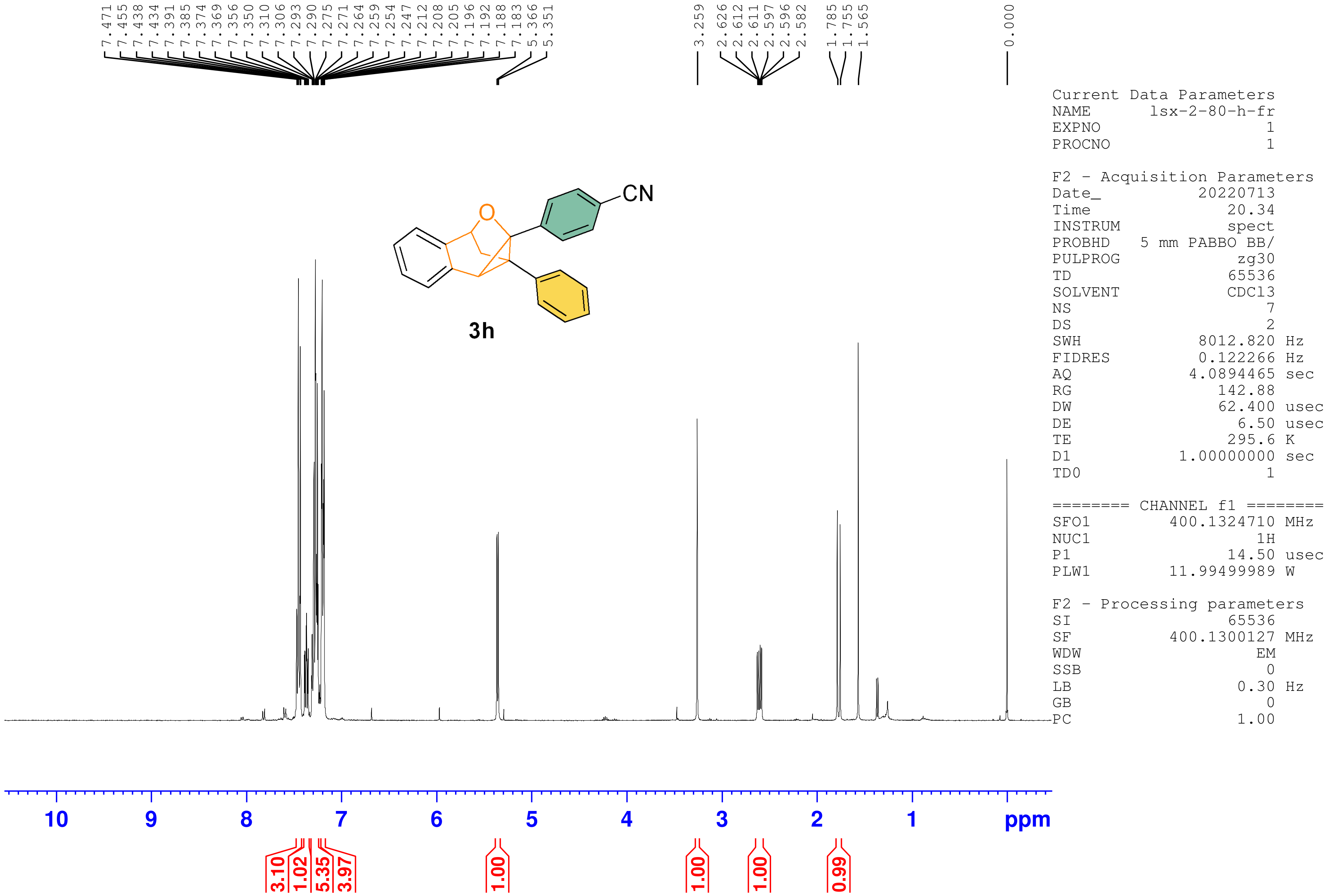
7.471
7.455
7.438
7.434
7.391
7.385
7.374
7.369
7.356
7.350
7.310
7.306
7.293
7.290
7.275
7.271
7.264
7.259
7.254
7.247
7.212
7.208
7.205
7.196
7.192
7.188
7.183
5.366
5.351
3.259
2.626
2.612
2.611
2.597
2.596
2.582
1.785
1.755
1.565
0.000
CN
O
3h
Current Data Parameters
NAME lsx-2-80-h-fr
EXPNO 1
PROCNO 1
F2 - Acquisition Parameters
Date_ 20220713
Time 20.34
INSTRUM spect
PROBHD 5 mm PABBO BB/
PULPROG zg30
TD 65536
SOLVENT CDCl3
NS 7
DS 2
SWH 8012.820 Hz
FIDRES 0.122266 Hz
AQ 4.0894465 sec
RG 142.88
DW 62.400 usec
DE 6.50 usec
TE 295.6 K
D1 1.00000000 sec
TD0 1
======== CHANNEL f1 ========
SFO1 400.1324710 MHz
NUC1 1H
P1 14.50 usec
PLW1 11.99499989 W
F2 - Processing parameters
SI 65536
SF 400.1300127 MHz
WDW EM
SSB 0
LB 0.30 Hz
GB 0
PC 1.00
10
9
8
7
6
5
4
3
2
1
ppm
3.10
1.02
5.35
3.97
1.00
1.00
1.00
0.99

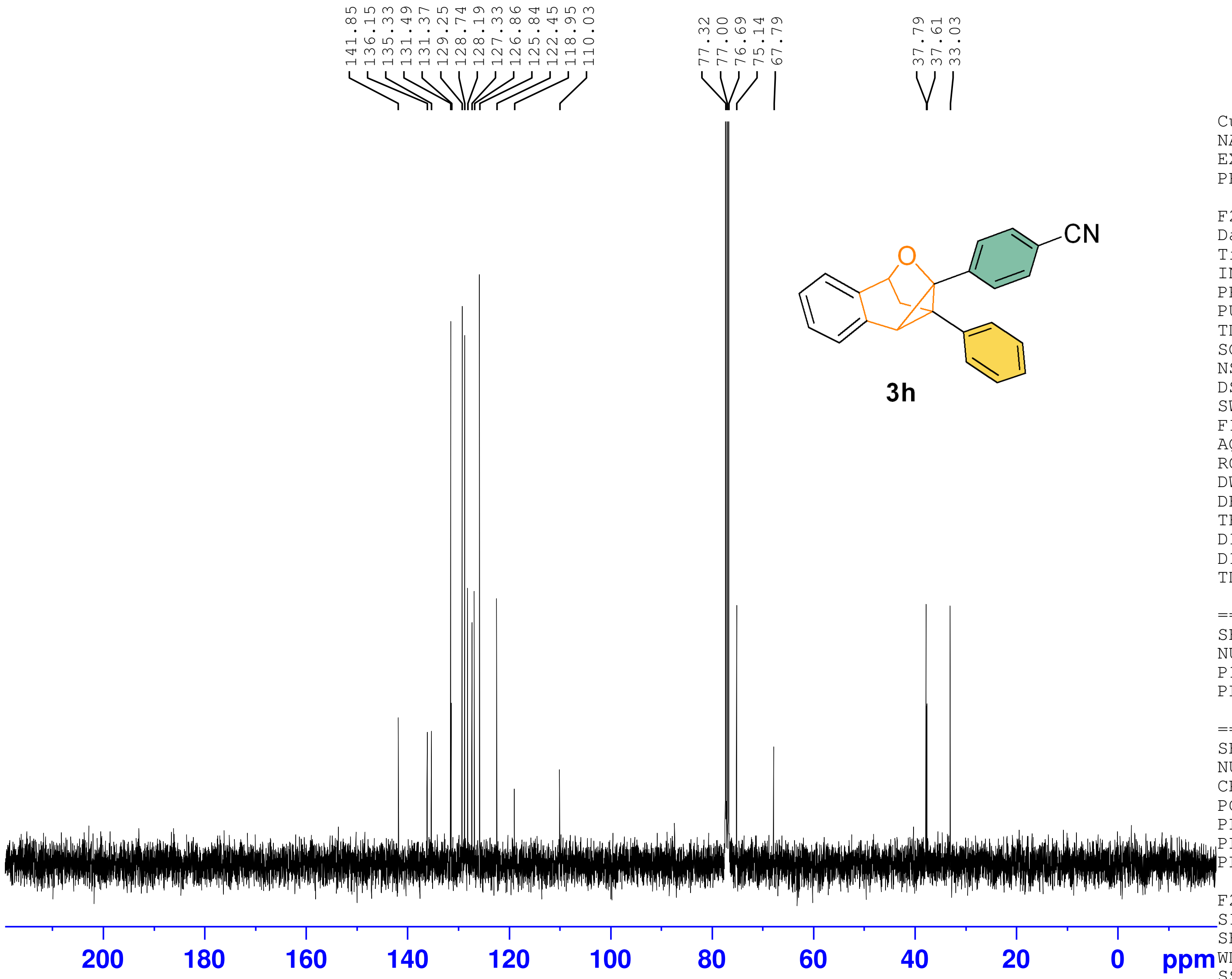


```
Current Data Parameters
NAME         lsx-2-80-c-fr
EXPNO                    1
PROCNO                   1

F2 - Acquisition Parameters
Date_             20220713
Time                 20.38
INSTRUM              spect
PROBHD     5 mm PABBO BB/
PULPROG            zgpg30
TD                   65536
SOLVENT              CDCl3
NS                      88
DS                       2
SWH              24038.461 Hz
FIDRES            0.366798 Hz
AQ               1.3631488 sec
RG                  196.92
DW                  20.800 usec
DE                    6.50 usec
TE                   296.4 K
D1              2.00000000 sec
D11             0.03000000 sec
TD0                      1

======== CHANNEL f1 ========
SFO1           100.6228298 MHz
NUC1                   13C
P1                    9.70 usec
PLW1           46.98899841 W

======== CHANNEL f2 ========
SFO2           400.1316005 MHz
NUC2                    1H
CPDPRG[2           waltz16
PCPD2                90.00 usec
PLW2           11.99499989 W
PLW12           0.34213999 W
PLW13           0.27713001 W

F2 - Processing parameters
SI                   32768
SF             100.6127729 MHz
WDW                     EM
SSB                      0
LB                    1.00 Hz
GB                       0
PC                    1.40
```

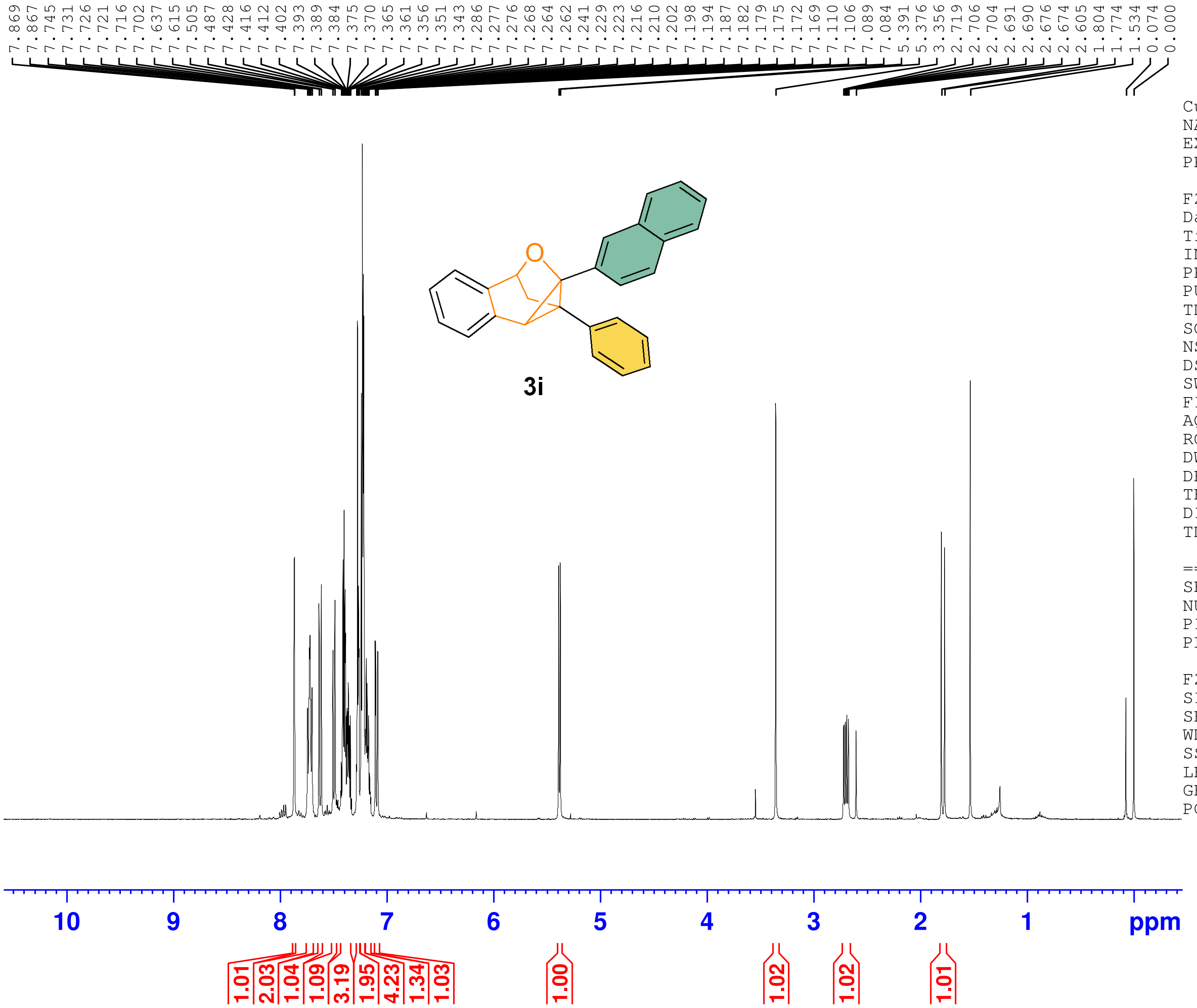

Current Data Parameters
NAME lsx-2-79-h-fr
EXPNO 1
PROCNO 1

F2 - Acquisition Parameters
Date_ 20220713
Time 20.24
INSTRUM spect
PROBHD 5 mm PABBO BB/
PULPROG zg30
TD 65536
SOLVENT CDCl3
NS 6
DS 2
SWH 8012.820 Hz
FIDRES 0.122266 Hz
AQ 4.0894465 sec
RG 112.31
DW 62.400 usec
DE 6.50 usec
TE 295.6 K
D1 1.00000000 sec
TD0 1

======== CHANNEL f1 ========
SFO1 400.1324710 MHz
NUC1 1H
P1 14.50 usec
PLW1 11.99499989 W

F2 - Processing parameters
SI 65536
SF 400.1300179 MHz
WDW EM
SSB 0
LB 0.30 Hz
GB 0
PC 1.00

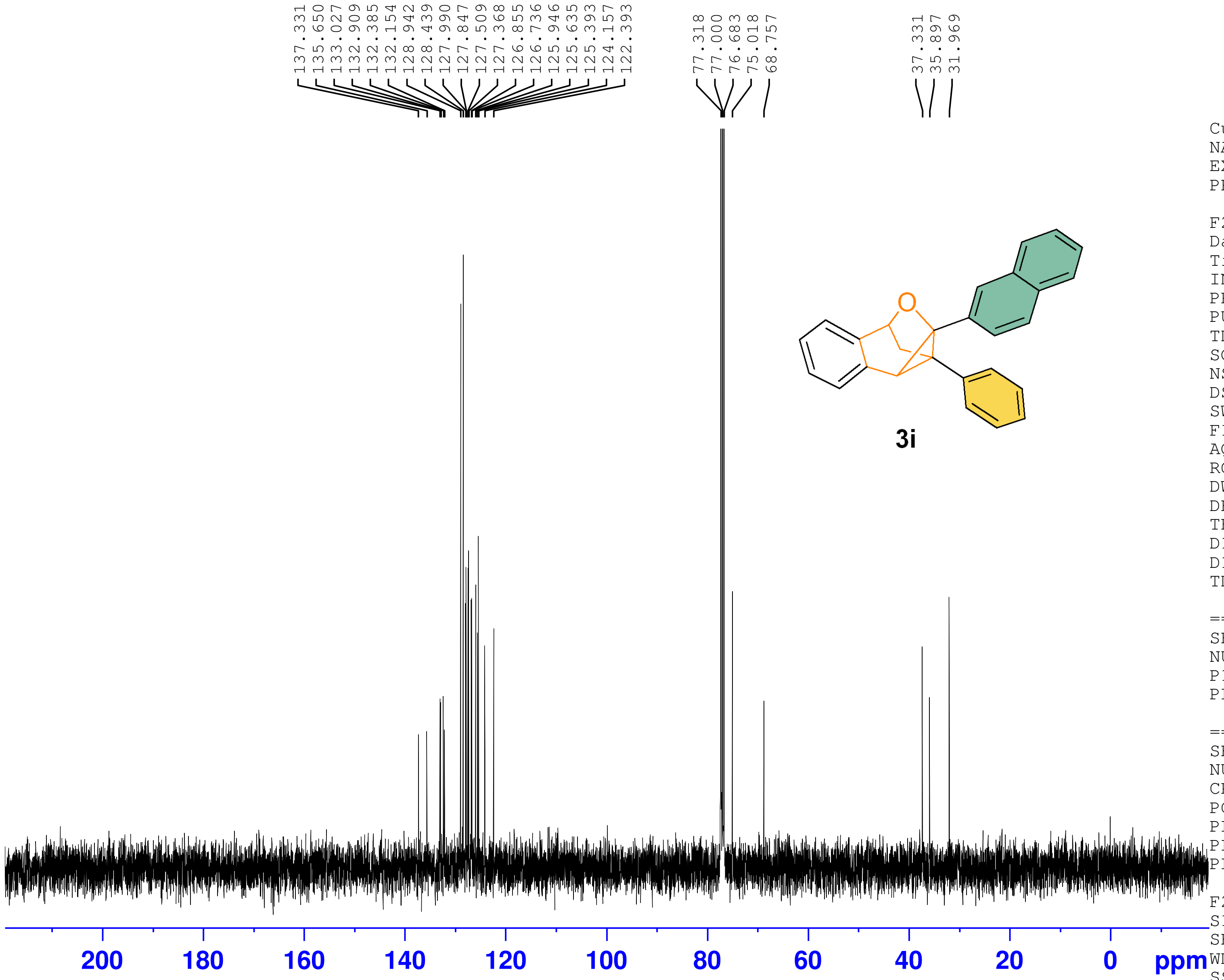

3i

```
Current Data Parameters
NAME          lsx-2-79-c-fr
EXPNO                     1
PROCNO                    1

F2 - Acquisition Parameters
Date_              20220713
Time                  20.31
INSTRUM               spect
PROBHD     5 mm PABBO BB/
PULPROG             zgpg30
TD                   65536
SOLVENT              CDCl3
NS                     105
DS                       2
SWH              24038.461 Hz
FIDRES            0.366798 Hz
AQ               1.3631488 sec
RG                  196.92
DW                  20.800 usec
DE                    6.50 usec
TE                   296.4 K
D1              2.00000000 sec
D11             0.03000000 sec
TD0                      1

======== CHANNEL f1 ========
SFO1         100.6228298 MHz
NUC1                 13C
P1                  9.70 usec
PLW1         46.98899841 W

======== CHANNEL f2 ========
SFO2         400.1316005 MHz
NUC2                  1H
CPDPRG[2          waltz16
PCPD2              90.00 usec
PLW2         11.99499989 W
PLW12         0.34213999 W
PLW13         0.27713001 W

F2 - Processing parameters
SI                 32768
SF          100.6127736 MHz
WDW                   EM
SSB                    0
LB                  1.00 Hz
GB                     0
PC                  1.40
```

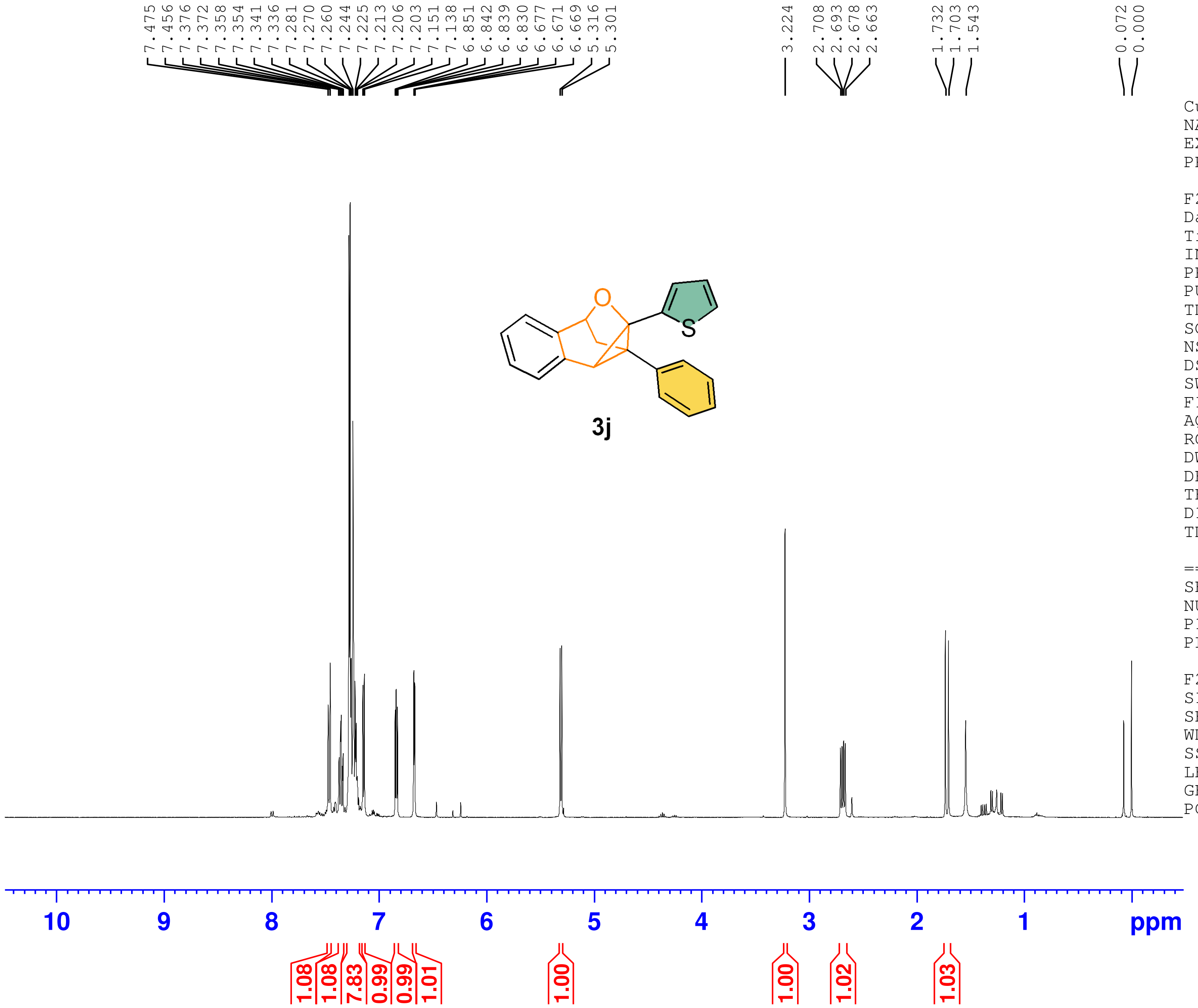


```
Current Data Parameters
NAME          lsx-2-78-h-fr
EXPNO                     1
PROCNO                    1

F2 - Acquisition Parameters
Date_              20220713
Time                  20.15
INSTRUM               spect
PROBHD     5 mm PABBO BB/
PULPROG                zg30
TD                    65536
SOLVENT               CDCl3
NS                       12
DS                        2
SWH                8012.820 Hz
FIDRES             0.122266 Hz
AQ                4.0894465 sec
RG                   112.31
DW                   62.400 usec
DE                     6.50 usec
TE                    295.4 K
D1               1.00000000 sec
TD0                       1

======== CHANNEL f1 ========
SFO1            400.1324710 MHz
NUC1                     1H
P1                    14.50 usec
PLW1            11.99499989 W

F2 - Processing parameters
SI                    65536
SF              400.1300162 MHz
WDW                      EM
SSB                       0
LB                     0.30 Hz
GB                        0
PC                     1.00
```

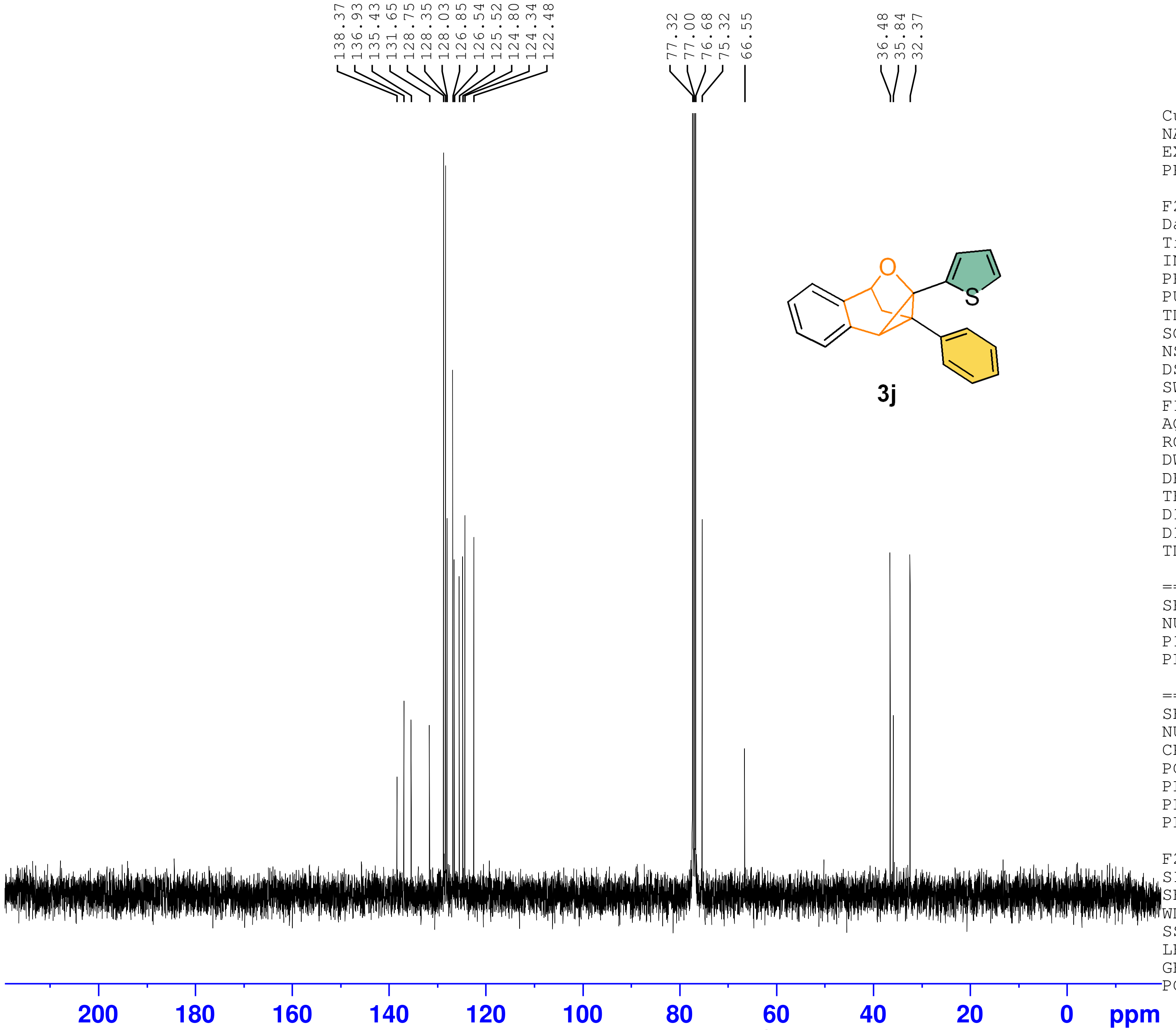


```
Current Data Parameters
NAME      lsx-2-78-c-fr
EXPNO                 1
PROCNO                1

F2 - Acquisition Parameters
Date_          20220713
Time              20.21
INSTRUM           spect
PROBHD   5 mm PABBO BB/
PULPROG         zgpg30
TD                65536
SOLVENT           CDCl3
NS                  103
DS                    2
SWH           24038.461 Hz
FIDRES         0.366798 Hz
AQ            1.3631488 sec
RG               196.92
DW               20.800 usec
DE                 6.50 usec
TE                296.4 K
D1           2.00000000 sec
D11          0.03000000 sec
TD0                   1

======== CHANNEL f1 ========
SFO1        100.6228298 MHz
NUC1                13C
P1                 9.70 usec
PLW1        46.98899841 W

======== CHANNEL f2 ========
SFO2        400.1316005 MHz
NUC2                 1H
CPDPRG[2        waltz16
PCPD2             90.00 usec
PLW2        11.99499989 W
PLW12        0.34213999 W
PLW13        0.27713001 W

F2 - Processing parameters
SI                32768
SF          100.6127736 MHz
WDW                  EM
SSB                   0
LB                 1.00 Hz
GB                    0
PC                 1.40
```

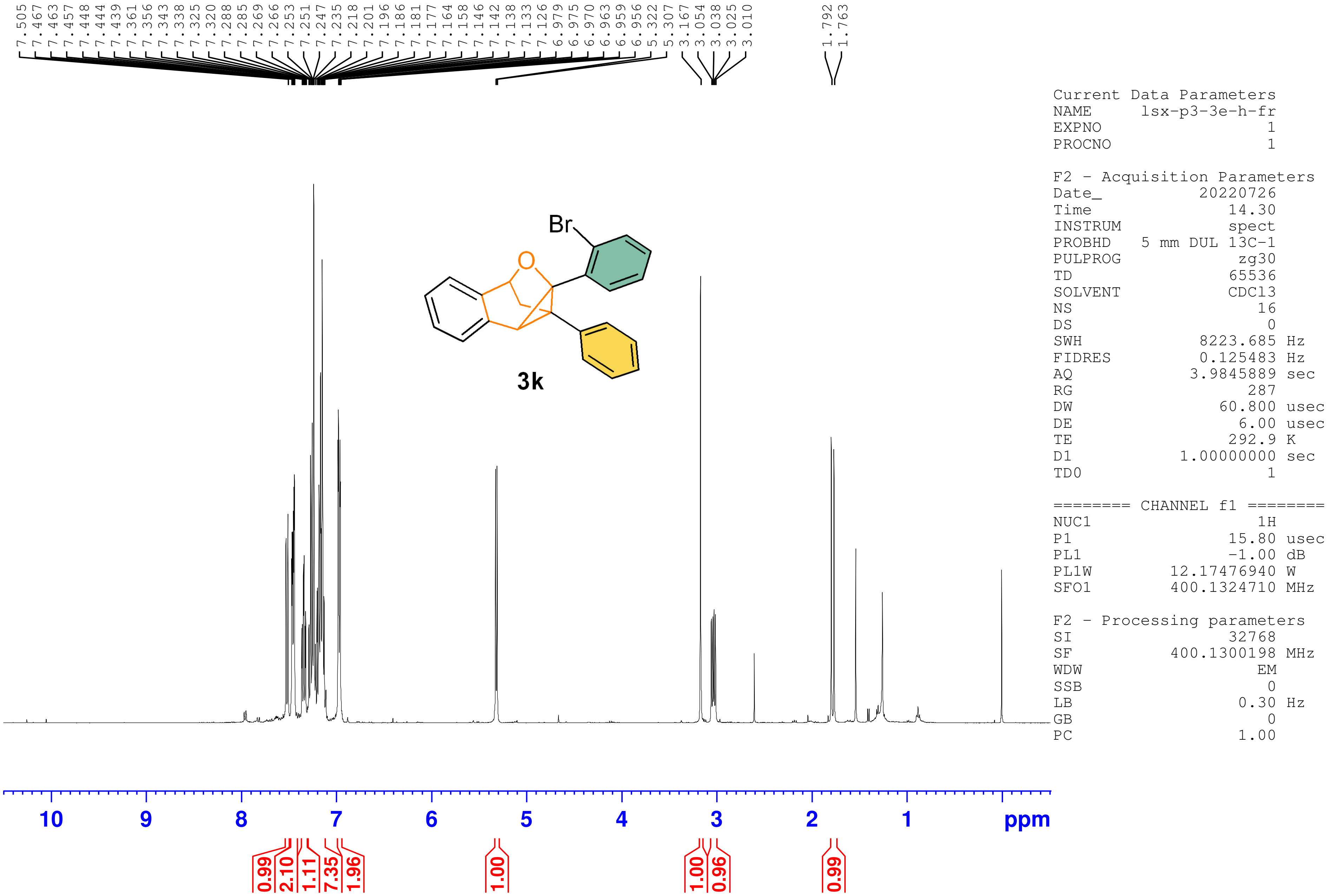
7.505 7.467 7.463 7.457 7.448 7.444 7.439 7.361 7.356 7.343 7.338 7.325 7.320 7.288 7.285 7.269 7.266 7.253 7.251 7.247 7.235 7.218 7.201 7.196 7.186 7.181 7.177 7.164 7.158 7.146 7.142 7.138 7.133 7.126 6.979 6.975 6.970 6.963 6.959 6.956 5.322 5.307 3.167 3.054 3.038 3.025 3.010 1.792 1.763
Br
O
3k
Current Data Parameters
NAME lsx-p3-3e-h-fr
EXPNO 1
PROCNO 1
F2 - Acquisition Parameters
Date_ 20220726
Time 14.30
INSTRUM spect
PROBHD 5 mm DUL 13C-1
PULPROG zg30
TD 65536
SOLVENT CDCl3
NS 16
DS 0
SWH 8223.685 Hz
FIDRES 0.125483 Hz
AQ 3.9845889 sec
RG 287
DW 60.800 usec
DE 6.00 usec
TE 292.9 K
D1 1.00000000 sec
TD0 1
======== CHANNEL f1 ========
NUC1 1H
P1 15.80 usec
PL1 -1.00 dB
PL1W 12.17476940 W
SFO1 400.1324710 MHz
F2 - Processing parameters
SI 32768
SF 400.1300198 MHz
WDW EM
SSB 0
LB 0.30 Hz
GB 0
PC 1.00
10 9 8 7 6 5 4 3 2 1 ppm
0.99 2.10 1.11 7.35 1.96 1.00 1.00 0.96 0.99

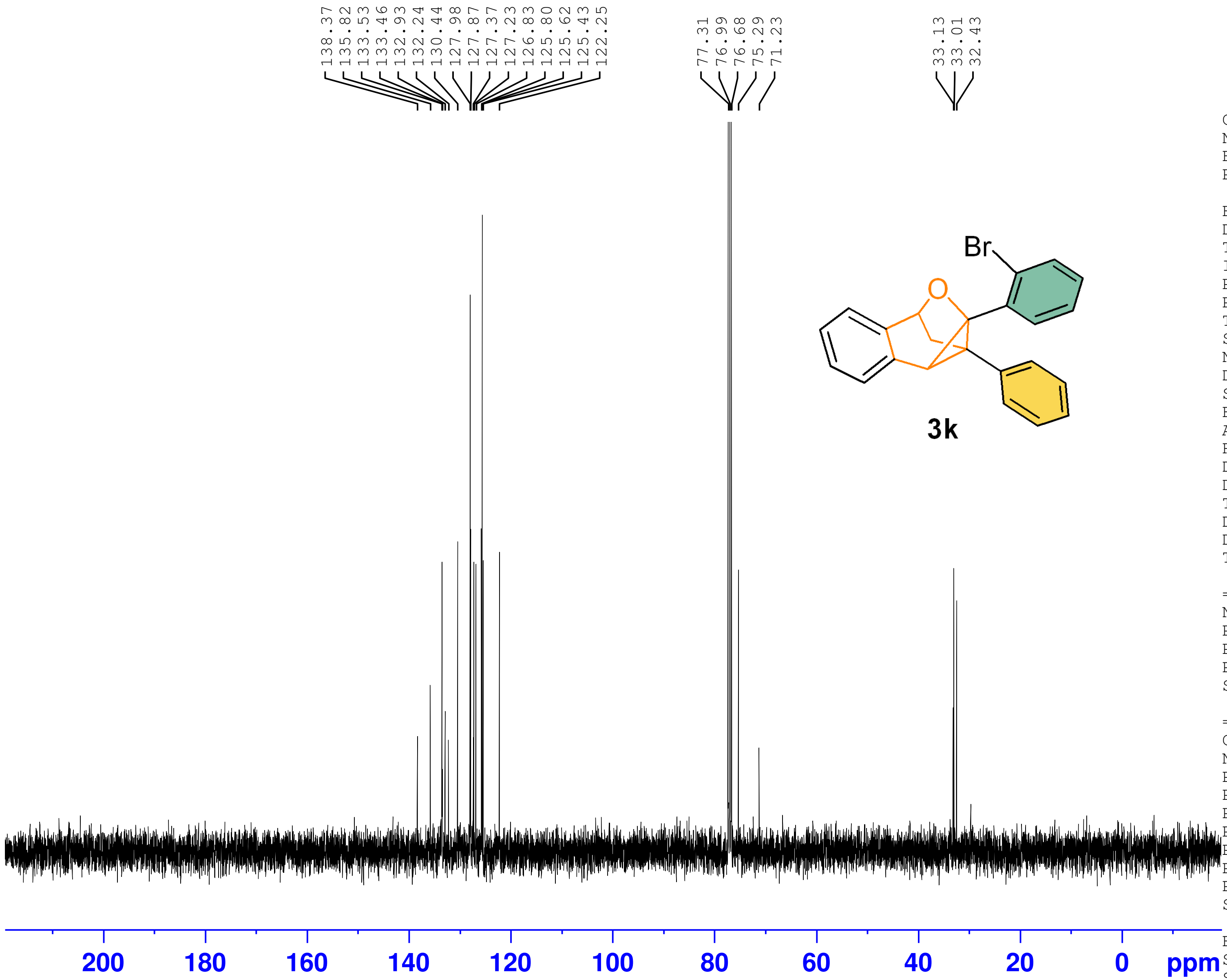

3k

```
Current Data Parameters
NAME      lsx-p3-3e-h-fr-C
EXPNO                    1
PROCNO                   1

F2 - Acquisition Parameters
Date_             20220726
Time                 14.31
INSTRUM              spect
PROBHD    5 mm DUL 13C-1
PULPROG            zgpg30
TD                   65536
SOLVENT              CDCl3
NS                      81
DS                       0
SWH            24038.461 Hz
FIDRES          0.366798 Hz
AQ             1.3631488 sec
RG                    2050
DW                  20.800 usec
DE                    6.00 usec
TE                   293.0 K
D1              2.00000000 sec
D11             0.03000000 sec
TD0                      1

======== CHANNEL f1 ========
NUC1                   13C
P1                   40.00 usec
PL1                  -3.00 dB
PL1W           60.64365387 W
SFO1          100.6228298 MHz

======== CHANNEL f2 ========
CPDPRG[2           waltz16
NUC2                    1H
PCPD2                80.00 usec
PL2                  -1.00 dB
PL12                 14.39 dB
PL13                 18.00 dB
PL2W           12.17476940 W
PL12W           0.35193357 W
PL13W           0.15327126 W
SFO2          400.1316005 MHz

F2 - Processing parameters
SI                   32768
SF            100.6127769 MHz
WDW                     EM
SSB                      0
LB                    1.00 Hz
GB                       0
PC                    1.40
```

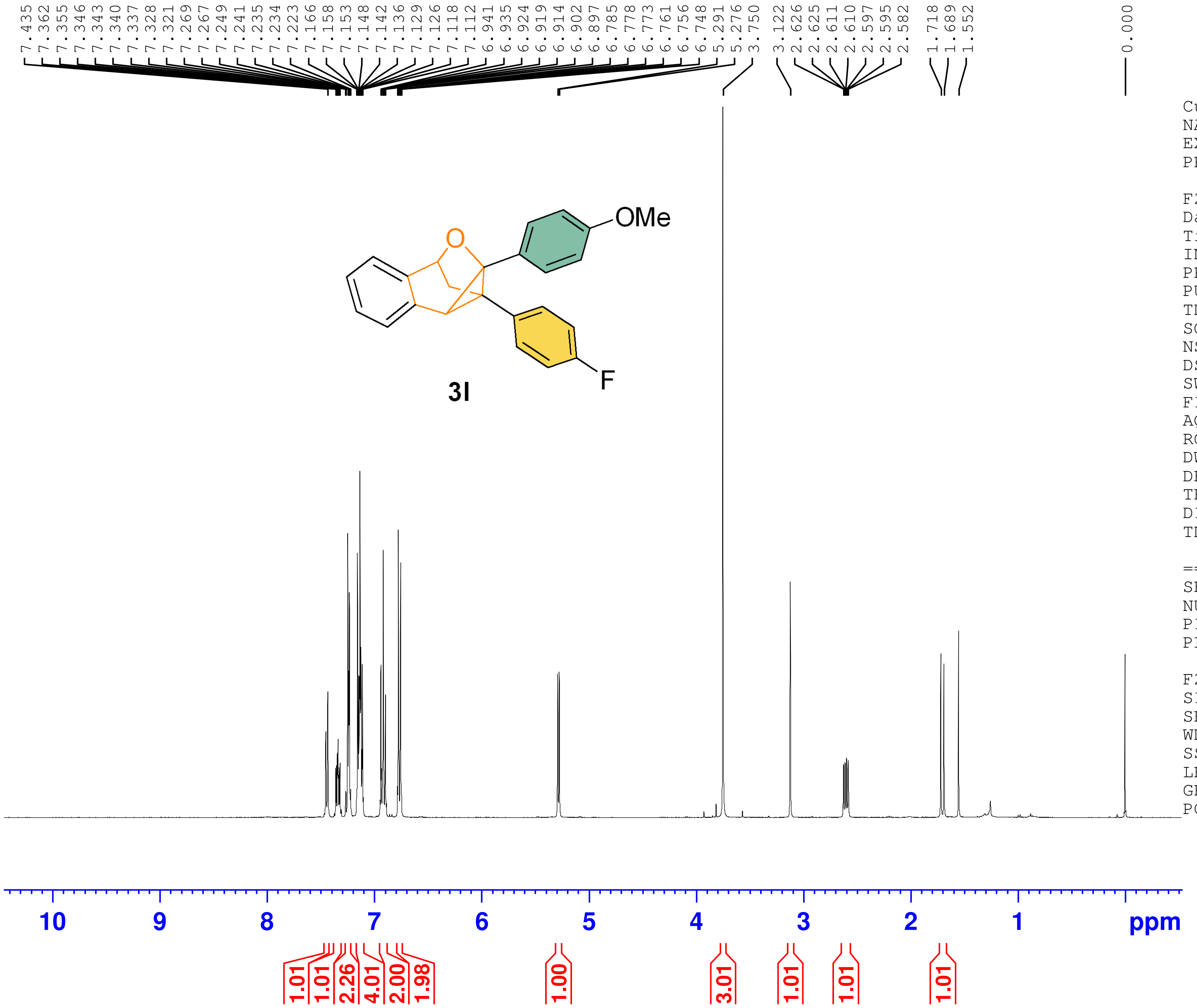


```
Current Data Parameters
NAME       lsx-2-32-h-fr
EXPNO                  1
PROCNO                 1

F2 - Acquisition Parameters
Date_           20220712
Time               16.43
INSTRUM            spect
PROBHD   5 mm PABBO BB/
PULPROG             zg30
TD                 65536
SOLVENT            CDCl3
NS                    16
DS                     2
SWH             8012.820 Hz
FIDRES          0.122266 Hz
AQ             4.0894465 sec
RG                103.52
DW                62.400 usec
DE                  6.50 usec
TE                 295.4 K
D1            1.00000000 sec
TD0                    1

======== CHANNEL f1 ========
SFO1         400.1324710 MHz
NUC1                  1H
P1                 14.50 usec
PLW1         11.99499989 W

F2 - Processing parameters
SI                 65536
SF           400.1300146 MHz
WDW                   EM
SSB                    0
LB                  0.30 Hz
GB                     0
PC                  1.00
```

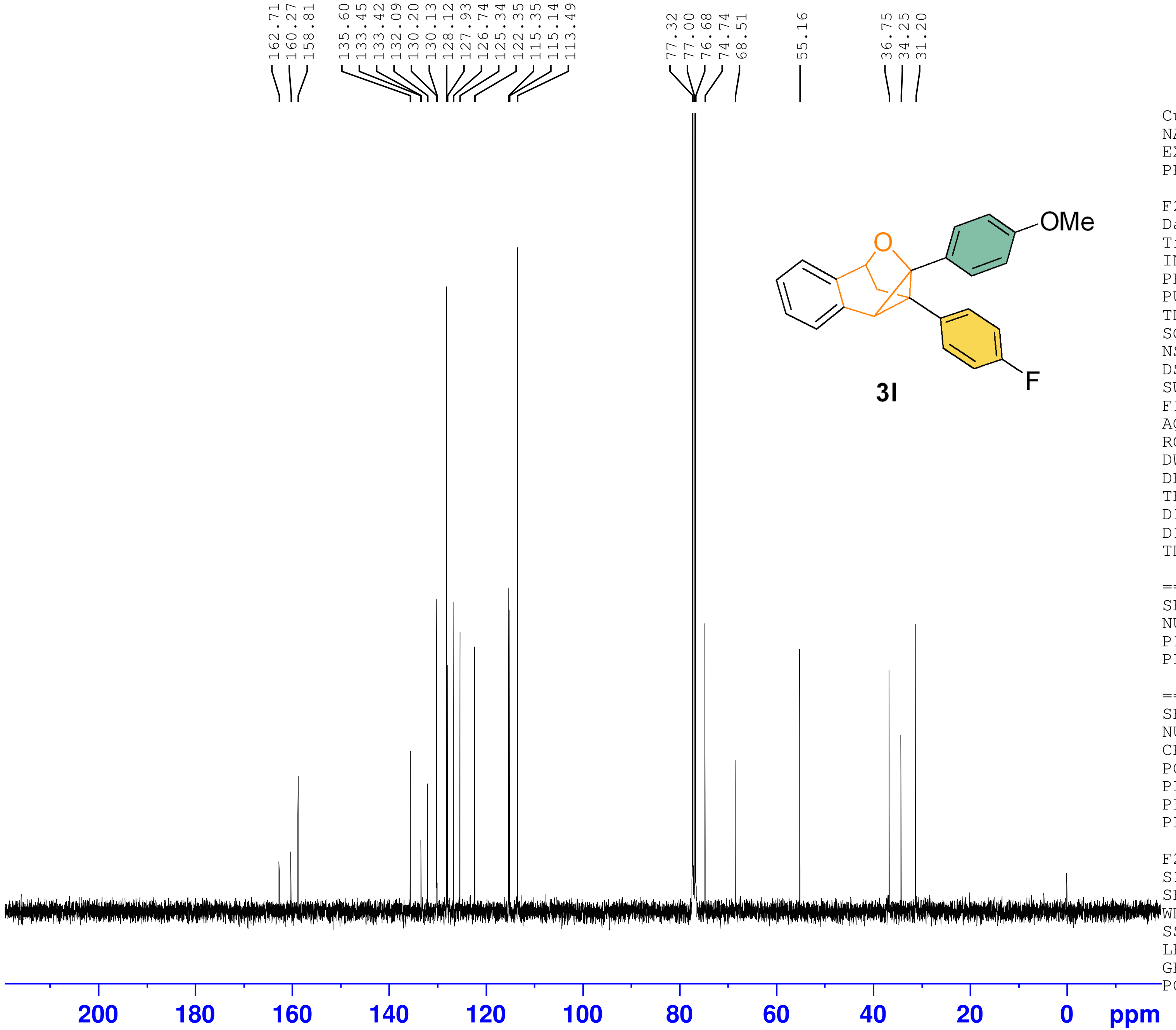

Current Data Parameters
NAME lsx-2-32-c-fr
EXPNO 1
PROCNO 1

F2 - Acquisition Parameters
Date_ 20220712
Time 16.59
INSTRUM spect
PROBHD 5 mm PABBO BB/
PULPROG zgpg30
TD 65536
SOLVENT CDCl3
NS 268
DS 2
SWH 24038.461 Hz
FIDRES 0.366798 Hz
AQ 1.3631488 sec
RG 196.92
DW 20.800 usec
DE 6.50 usec
TE 296.5 K
D1 2.00000000 sec
D11 0.03000000 sec
TD0 1

======== CHANNEL f1 ========
SFO1 100.6228298 MHz
NUC1 13C
P1 9.70 usec
PLW1 46.98899841 W

======== CHANNEL f2 ========
SFO2 400.1316005 MHz
NUC2 1H
CPDPRG[2 waltz16
PCPD2 90.00 usec
PLW2 11.99499989 W
PLW12 0.34213999 W
PLW13 0.27713001 W

F2 - Processing parameters
SI 32768
SF 100.6127729 MHz
WDW EM
SSB 0
LB 1.00 Hz
GB 0
PC 1.40

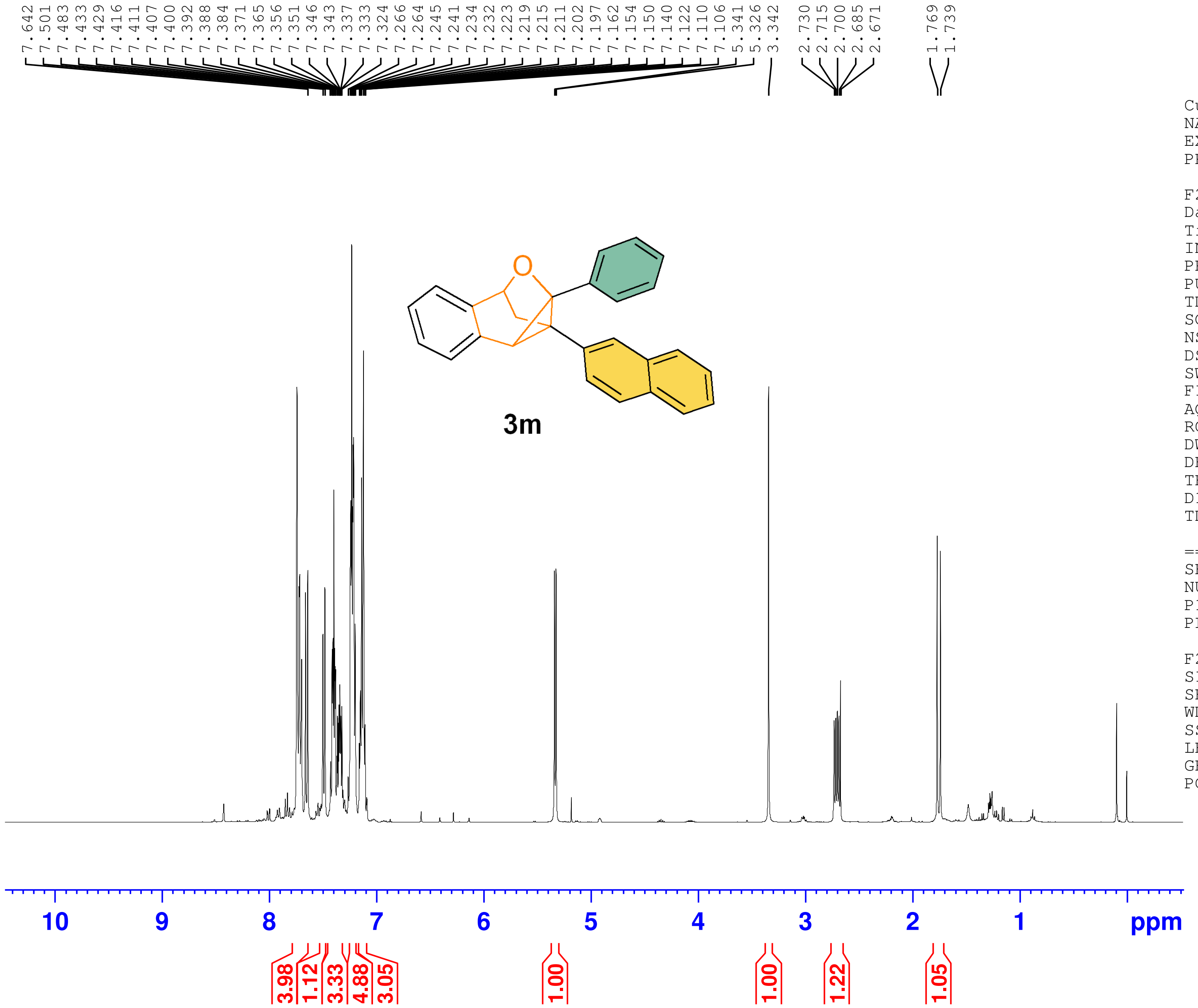


```
Current Data Parameters
NAME       lsx-project3-3m-h-fr
EXPNO                     1
PROCNO                    1

F2 - Acquisition Parameters
Date_              20220723
Time                  14.19
INSTRUM               spect
PROBHD    5 mm PABBO BB/
PULPROG                zg30
TD                    65536
SOLVENT               CDCl3
NS                       16
DS                        2
SWH                8012.820 Hz
FIDRES             0.122266 Hz
AQ                4.0894465 sec
RG                    39.46
DW                   62.400 usec
DE                     6.50 usec
TE                    295.4 K
D1               1.00000000 sec
TD0                       1

======== CHANNEL f1 ========
SFO1            400.1324710 MHz
NUC1                     1H
P1                    14.50 usec
PLW1            11.99499989 W

F2 - Processing parameters
SI                    65536
SF              400.1300537 MHz
WDW                      EM
SSB                       0
LB                     0.30 Hz
GB                        0
PC                     1.00
```

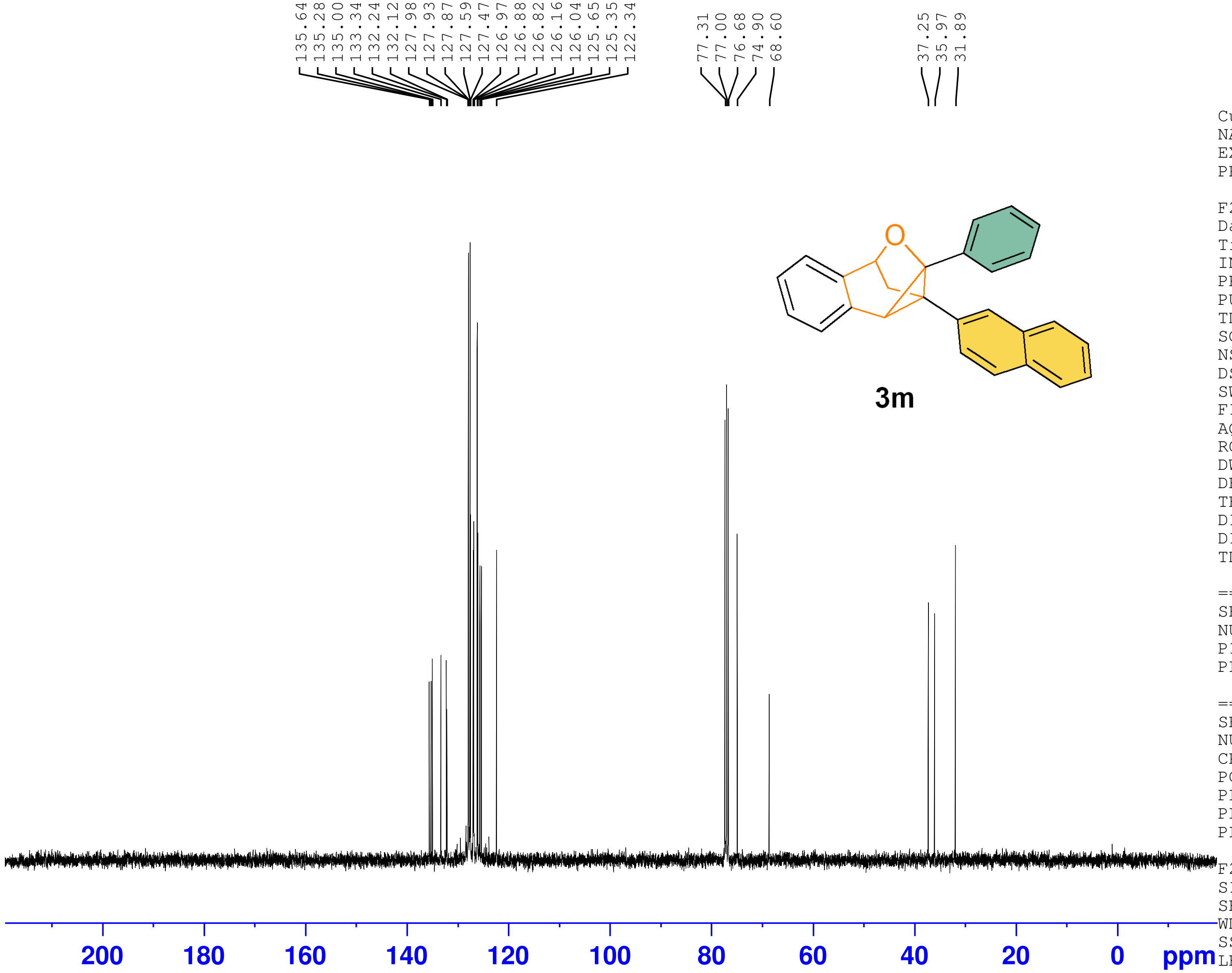

Current Data Parameters

NAME	lsx-project3-3m-c-fr
EXPNO	1
PROCNO	1

F2 - Acquisition Parameters

Date_	20220723
Time	14.22
INSTRUM	spect
PROBHD	5 mm PABBO BB/
PULPROG	zgpg30
TD	65536
SOLVENT	CDCl3
NS	46
DS	2
SWH	24038.461 Hz
FIDRES	0.366798 Hz
AQ	1.3631488 sec
RG	196.92
DW	20.800 usec
DE	6.50 usec
TE	296.2 K
D1	2.00000000 sec
D11	0.03000000 sec
TD0	1

======== CHANNEL f1 ========

SFO1	100.6228298 MHz
NUC1	13C
P1	9.70 usec
PLW1	46.98899841 W

======== CHANNEL f2 ========

SFO2	400.1316005 MHz
NUC2	1H
CPDPRG[2	waltz16
PCPD2	90.00 usec
PLW2	11.99499989 W
PLW12	0.34213999 W
PLW13	0.27713001 W

F2 - Processing parameters

SI	32768
SF	100.6127872 MHz
WDW	EM
SSB	0
LB	1.00 Hz
GB	0
PC	1.40

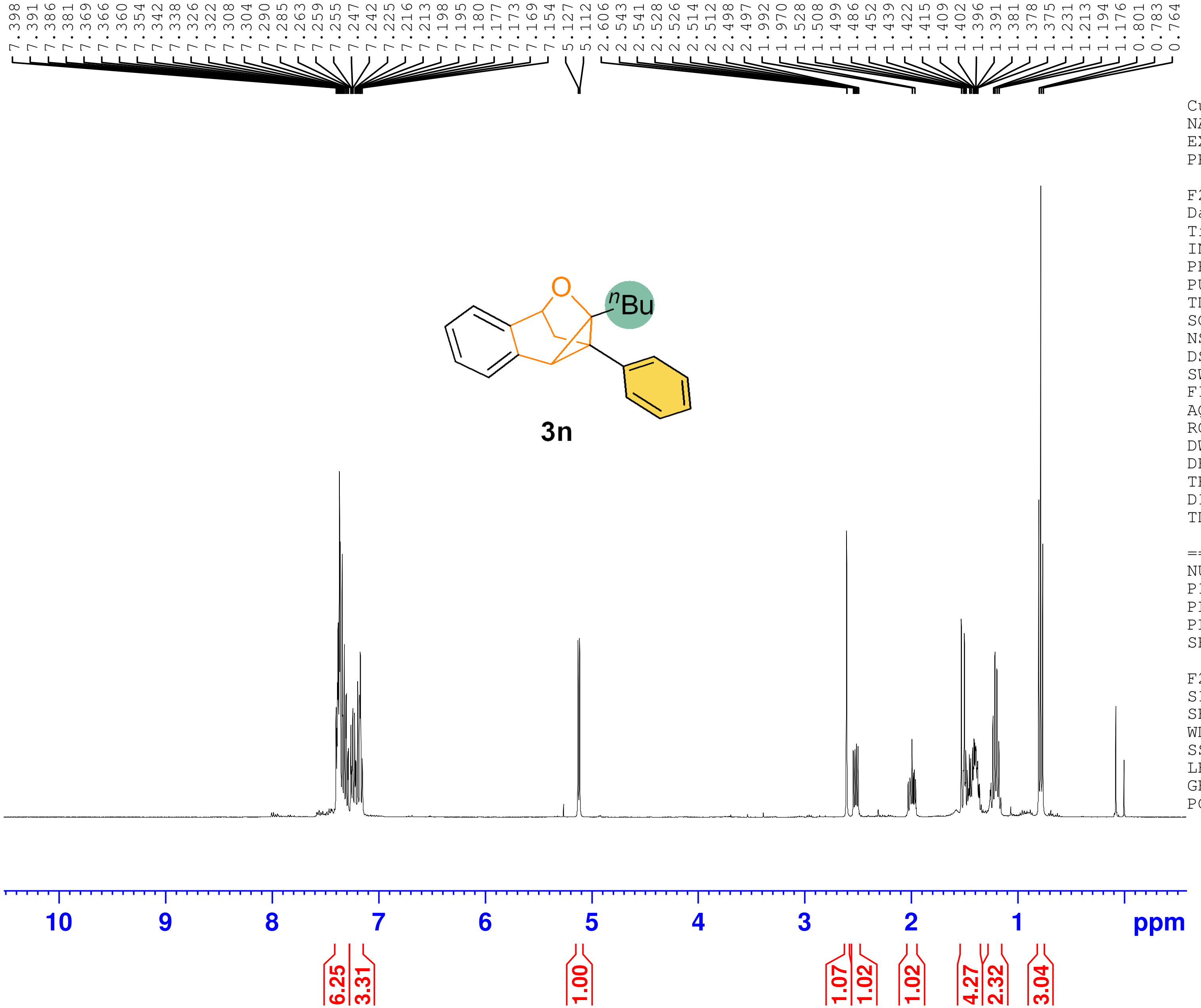


```
Current Data Parameters
NAME      lsx-project3-3k-h-fr
EXPNO                        1
PROCNO                       1

F2 - Acquisition Parameters
Date_             20220725
Time                 20.53
INSTRUM              spect
PROBHD    5 mm DUL 13C-1
PULPROG               zg30
TD                   65536
SOLVENT              CDCl3
NS                      16
DS                       2
SWH               8223.685 Hz
FIDRES            0.125483 Hz
AQ               3.9845889 sec
RG                     144
DW                  60.800 usec
DE                    6.00 usec
TE                   292.7 K
D1              1.00000000 sec
TD0                      1

======== CHANNEL f1 ========
NUC1                    1H
P1                   15.80 usec
PL1                  -1.00 dB
PL1W           12.17476940 W
SFO1           400.1324710 MHz

F2 - Processing parameters
SI                   32768
SF             400.1300236 MHz
WDW                     EM
SSB                      0
LB                    0.30 Hz
GB                       0
PC                    1.00
```

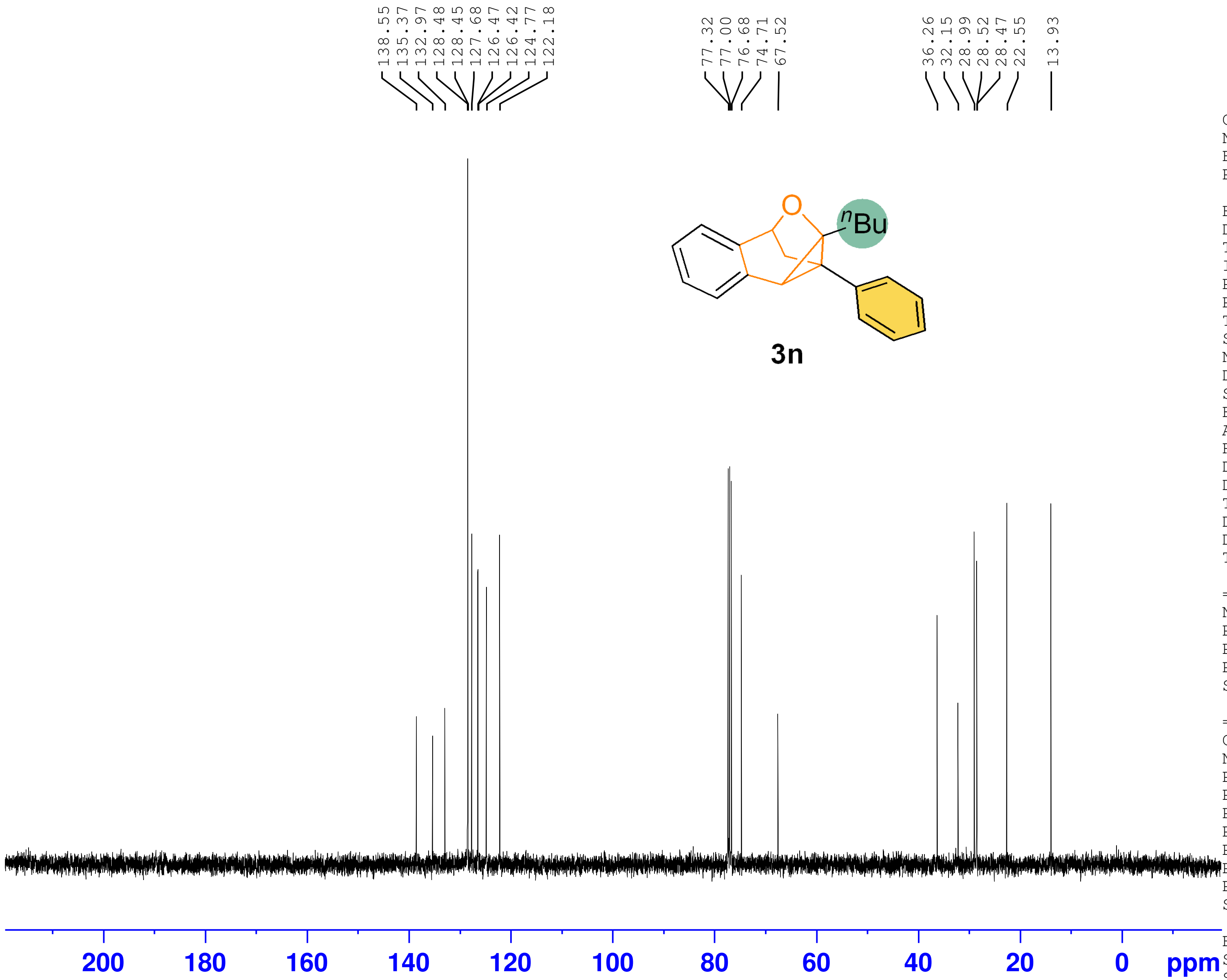


```
Current Data Parameters
NAME     lsx-project3-3k-c-fr
EXPNO                       1
PROCNO                      1

F2 - Acquisition Parameters
Date_              20220725
Time                  21.01
INSTRUM               spect
PROBHD    5 mm DUL 13C-1
PULPROG              zgpg30
TD                    65536
SOLVENT               CDCl3
NS                      126
DS                        1
SWH            24038.461 Hz
FIDRES          0.366798 Hz
AQ             1.3631488 sec
RG                     2050
DW                20.800 usec
DE                  6.00 usec
TE                 293.1 K
D1            2.00000000 sec
D11           0.03000000 sec
TD0                       1

======== CHANNEL f1 ========
NUC1                    13C
P1                 40.00 usec
PL1                -3.00 dB
PL1W         60.64365387 W
SFO1         100.6228298 MHz

======== CHANNEL f2 ========
CPDPRG[2           waltz16
NUC2                     1H
PCPD2              80.00 usec
PL2                -1.00 dB
PL12               14.39 dB
PL13               18.00 dB
PL2W         12.17476940 W
PL12W         0.35193357 W
PL13W         0.15327126 W
SFO2         400.1316005 MHz

F2 - Processing parameters
SI                    32768
SF           100.6127774 MHz
WDW                      EM
SSB                       0
LB                  1.00 Hz
GB                        0
PC                     1.40
```

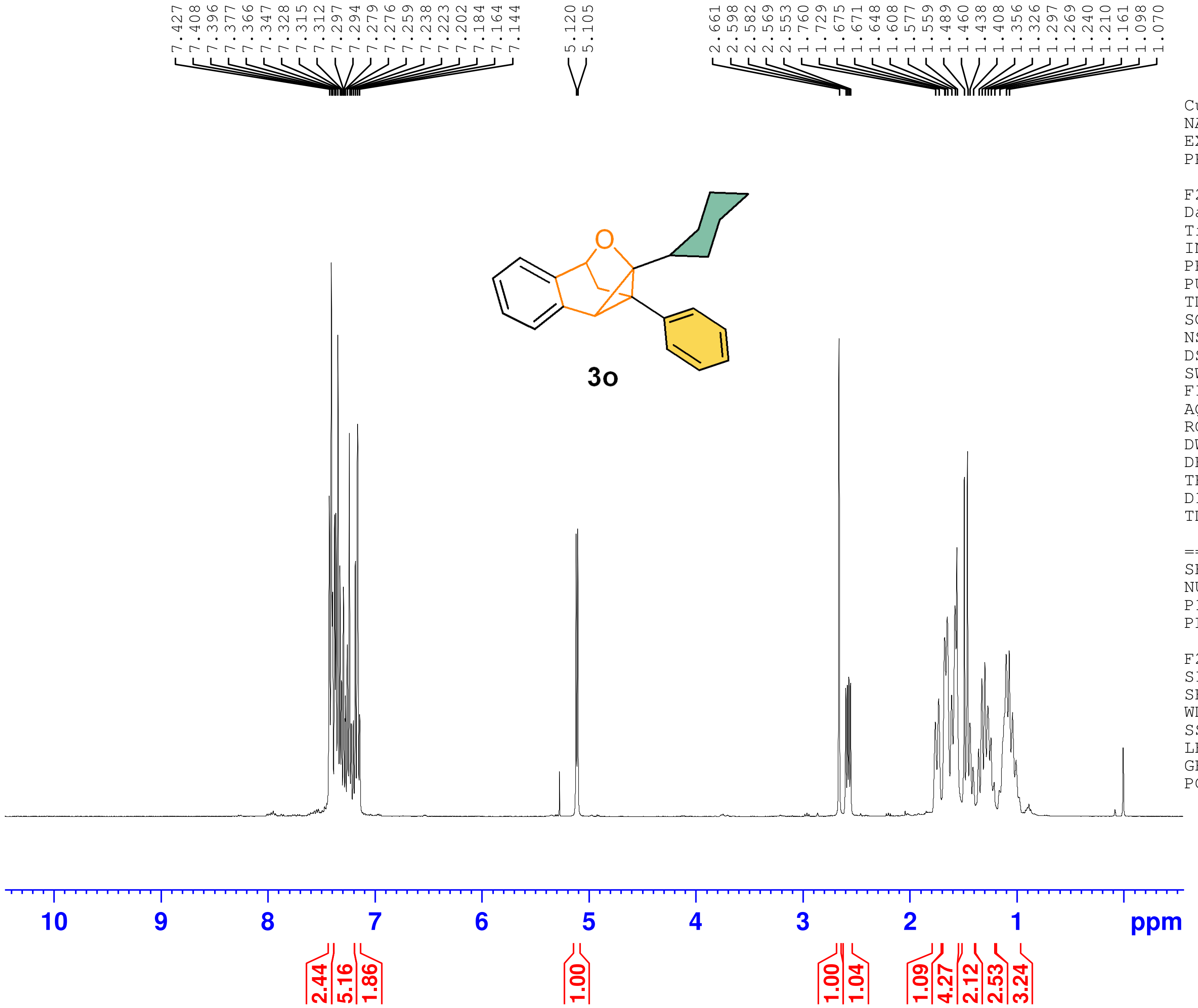

Current Data Parameters
NAME lsx-project3-3l-h-fr
EXPNO 1
PROCNO 1

F2 - Acquisition Parameters
Date_ 20220723
Time 14.12
INSTRUM spect
PROBHD 5 mm PABBO BB/
PULPROG zg30
TD 65536
SOLVENT CDCl3
NS 16
DS 2
SWH 8012.820 Hz
FIDRES 0.122266 Hz
AQ 4.0894465 sec
RG 31.55
DW 62.400 usec
DE 6.50 usec
TE 295.4 K
D1 1.00000000 sec
TD0 1

======== CHANNEL f1 ========
SFO1 400.1324710 MHz
NUC1 1H
P1 14.50 usec
PLW1 11.99499989 W

F2 - Processing parameters
SI 65536
SF 400.1300190 MHz
WDW EM
SSB 0
LB 0.30 Hz
GB 0
PC 1.00

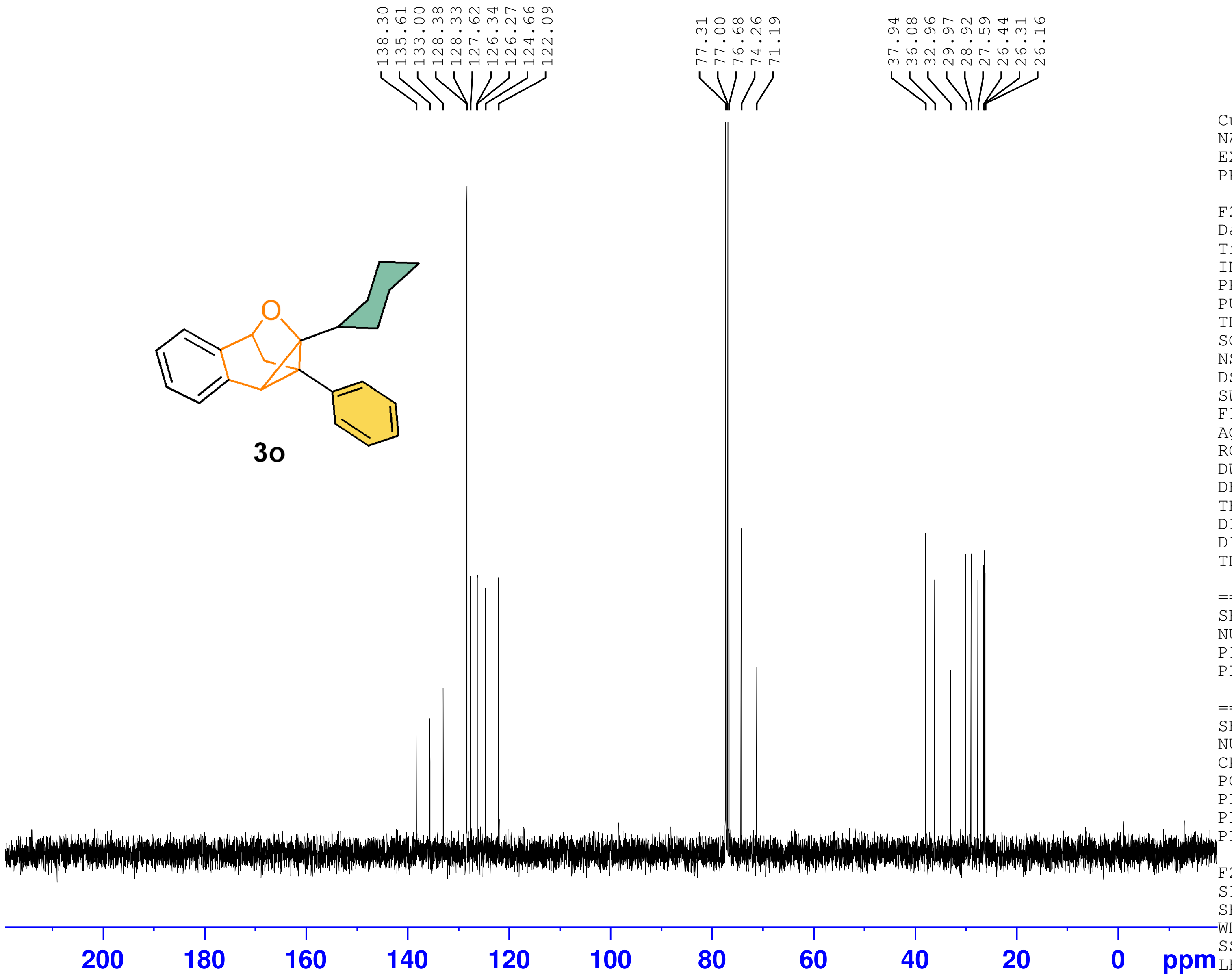

3o

```
Current Data Parameters
NAME       lsx-project3-3l-c-fr
EXPNO                         1
PROCNO                        1

F2 - Acquisition Parameters
Date_              20220723
Time                  14.15
INSTRUM               spect
PROBHD    5 mm PABBO BB/
PULPROG              zgpg30
TD                    65536
SOLVENT               CDCl3
NS                       30
DS                        2
SWH               24038.461 Hz
FIDRES             0.366798 Hz
AQ                1.3631488 sec
RG                   196.92
DW                   20.800 usec
DE                     6.50 usec
TE                    296.1 K
D1               2.00000000 sec
D11              0.03000000 sec
TD0                       1

======== CHANNEL f1 ========
SFO1           100.6228298 MHz
NUC1                   13C
P1                    9.70 usec
PLW1           46.98899841 W

======== CHANNEL f2 ========
SFO2           400.1316005 MHz
NUC2                    1H
CPDPRG[2           waltz16
PCPD2                90.00 usec
PLW2           11.99499989 W
PLW12           0.34213999 W
PLW13           0.27713001 W

F2 - Processing parameters
SI                   32768
SF             100.6127761 MHz
WDW                     EM
SSB                      0
LB                    1.00 Hz
GB                       0
PC                    1.40
```

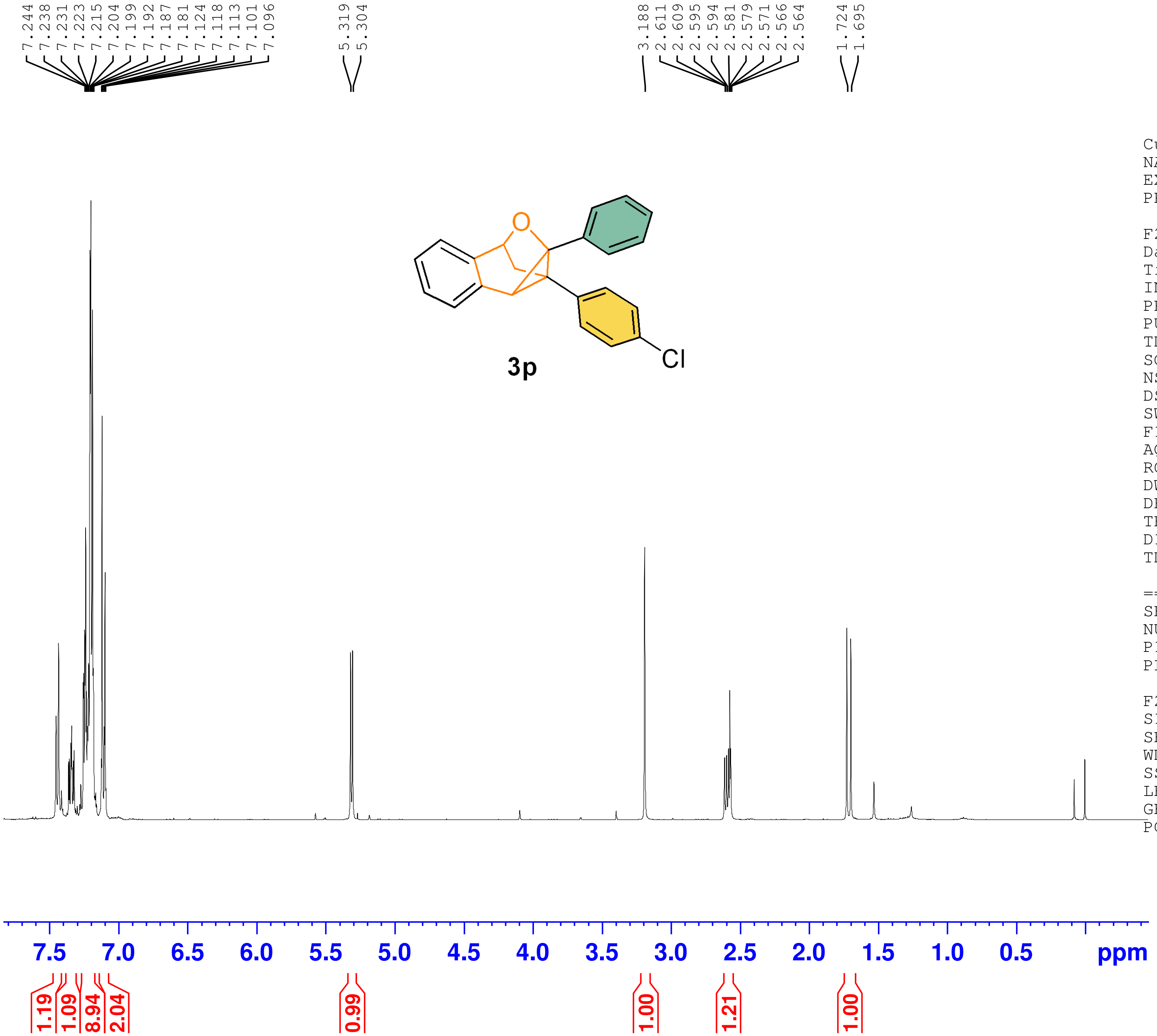


```
Current Data Parameters
NAME      lsx-8-143cc-h-fr
EXPNO                    1
PROCNO                   1

F2 - Acquisition Parameters
Date_             20240722
Time                 19.31
INSTRUM              spect
PROBHD   5 mm PABBO BB/
PULPROG               zg30
TD                   65536
SOLVENT              CDCl3
NS                      16
DS                       2
SWH               8012.820 Hz
FIDRES            0.122266 Hz
AQ               4.0894465 sec
RG                   62.93
DW                  62.400 usec
DE                    6.50 usec
TE                   296.1 K
D1              1.00000000 sec
TD0                      1

======== CHANNEL f1 ========
SFO1           400.1324710 MHz
NUC1                    1H
P1                   14.50 usec
PLW1           11.99499989 W

F2 - Processing parameters
SI                   65536
SF             400.1300214 MHz
WDW                     EM
SSB                      0
LB                    0.30 Hz
GB                       0
PC                    1.00
```

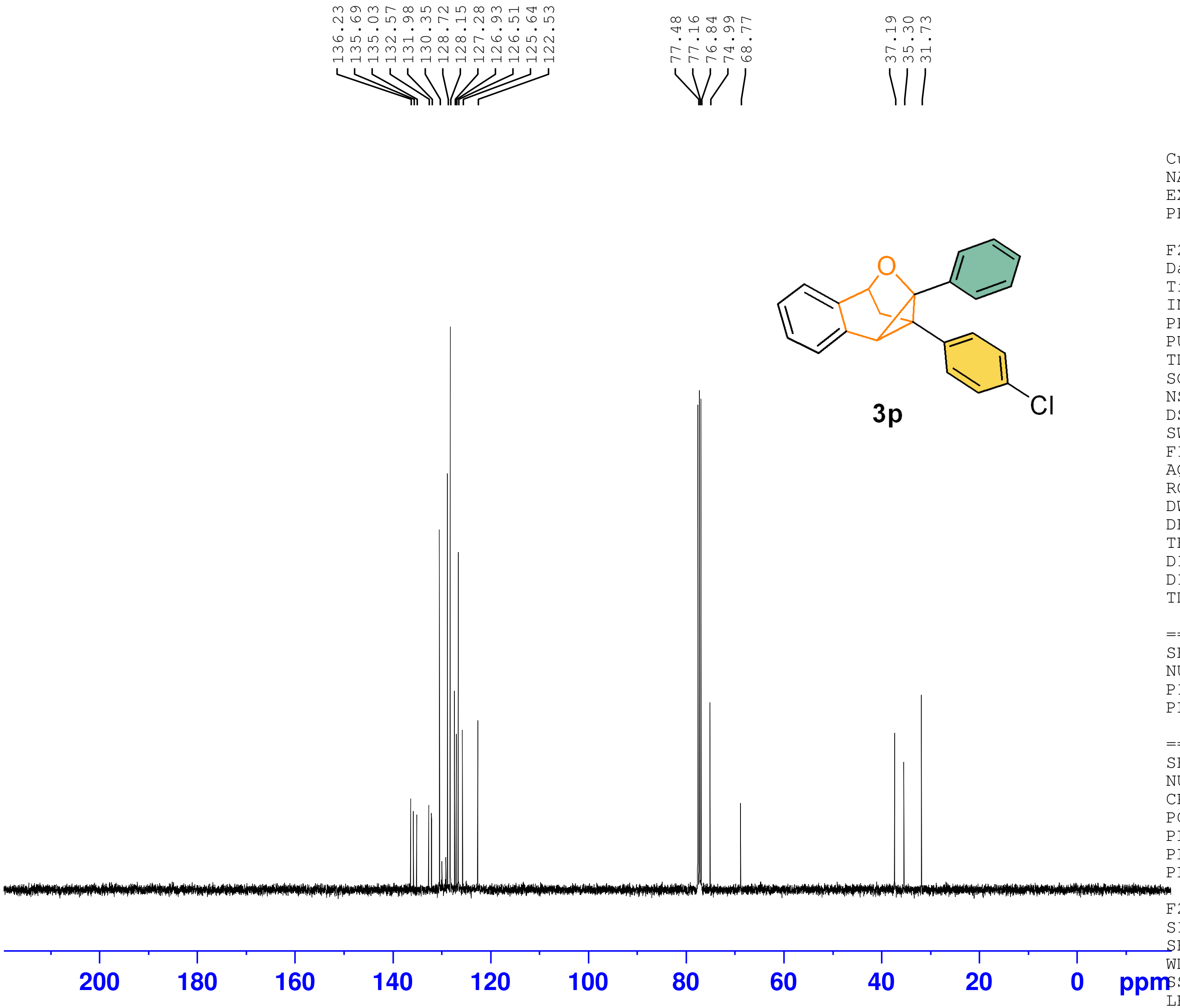


```
Current Data Parameters
NAME      lsx-8-143cc-c-fr
EXPNO                   1
PROCNO                  1

F2 - Acquisition Parameters
Date_            20240722
Time                19.38
INSTRUM             spect
PROBHD   5 mm PABBO BB/
PULPROG           zgpg30
TD                  65536
SOLVENT             CDCl3
NS                    116
DS                      2
SWH            24038.461 Hz
FIDRES          0.366798 Hz
AQ             1.3631488 sec
RG                196.92
DW                20.800 usec
DE                  6.50 usec
TE                 297.0 K
D1            2.00000000 sec
D11           0.03000000 sec
TD0                     1

======== CHANNEL f1 ========
SFO1         100.6228298 MHz
NUC1                 13C
P1                  9.70 usec
PLW1         46.98899841 W

======== CHANNEL f2 ========
SFO2         400.1316005 MHz
NUC2                  1H
CPDPRG[2          waltz16
PCPD2              90.00 usec
PLW2         11.99499989 W
PLW12         0.34213999 W
PLW13         0.27713001 W

F2 - Processing parameters
SI                  32768
SF           100.6127597 MHz
WDW                    EM
SSB                     0
LB                   1.00 Hz
GB                      0
PC                   1.40
```

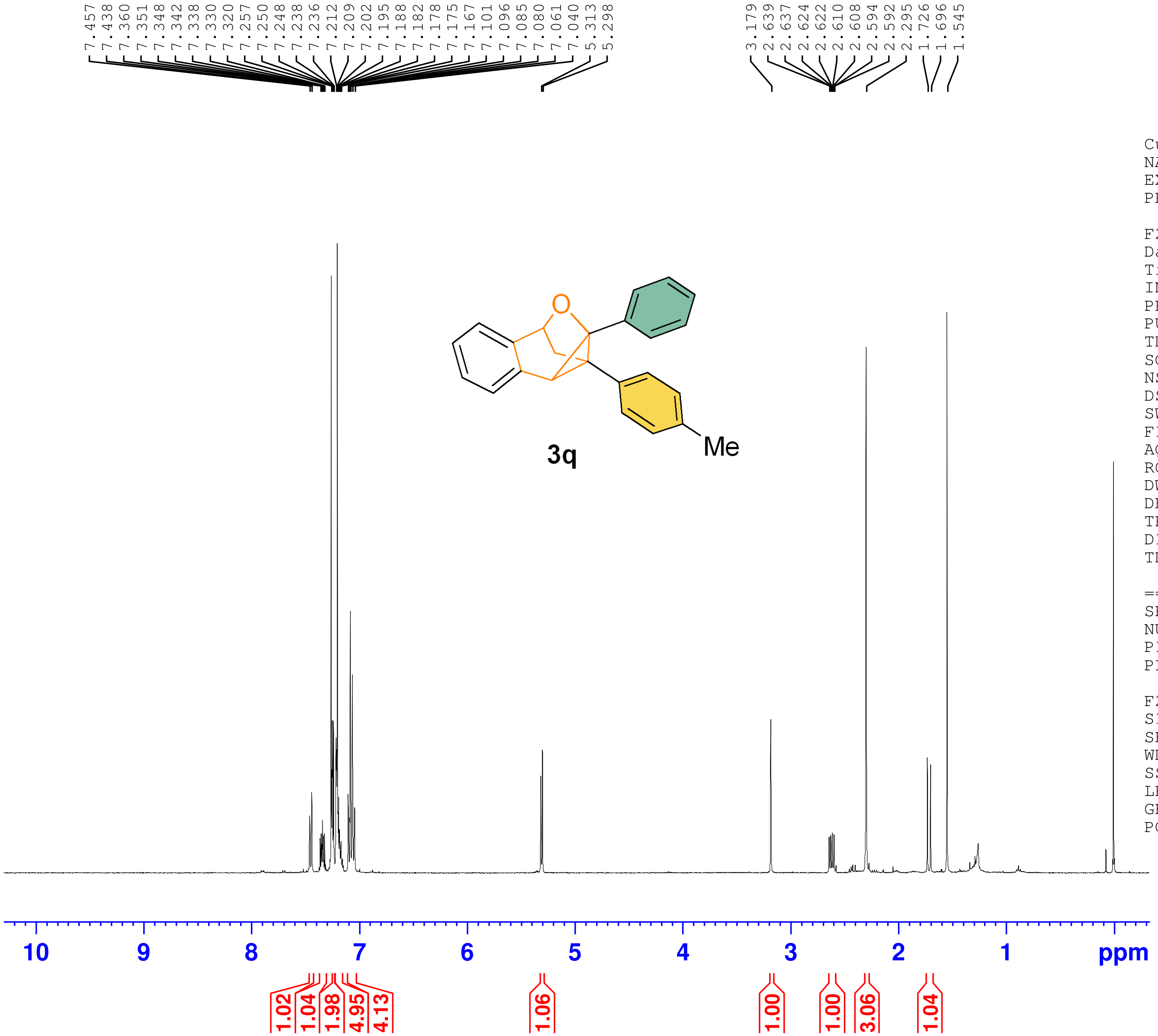


```
Current Data Parameters
NAME       lsx-8-143b-h-fr
EXPNO                    1
PROCNO                   1

F2 - Acquisition Parameters
Date_             20240721
Time                 18.22
INSTRUM              spect
PROBHD   5 mm PABBO BB/
PULPROG               zg30
TD                   65536
SOLVENT              CDCl3
NS                      16
DS                       2
SWH              8012.820 Hz
FIDRES           0.122266 Hz
AQ              4.0894465 sec
RG                  164.33
DW                  62.400 usec
DE                    6.50 usec
TE                   296.2 K
D1             1.00000000 sec
TD0                      1

======== CHANNEL f1 ========
SFO1       400.1324710 MHz
NUC1                    1H
P1                   14.50 usec
PLW1          11.99499989 W

F2 - Processing parameters
SI                   65536
SF         400.1300107 MHz
WDW                     EM
SSB                      0
LB                    0.30 Hz
GB                       0
PC                    1.00
```

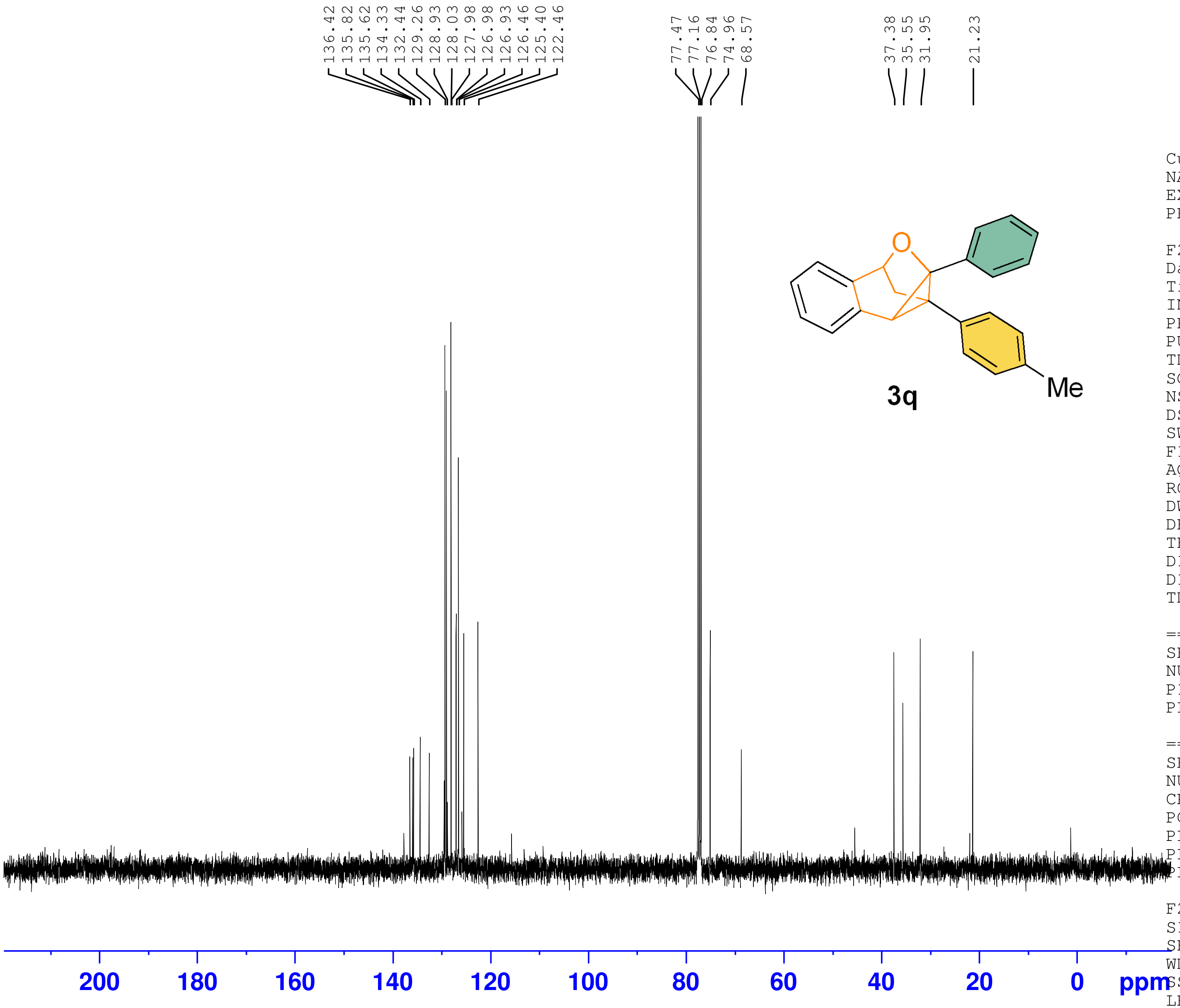

Current Data Parameters
NAME lsx-8-143bb-c-fr
EXPNO 1
PROCNO 1

F2 - Acquisition Parameters
Date_ 20240722
Time 19.26
INSTRUM spect
PROBHD 5 mm PABBO BB/
PULPROG zgpg30
TD 65536
SOLVENT CDCl3
NS 71
DS 2
SWH 24038.461 Hz
FIDRES 0.366798 Hz
AQ 1.3631488 sec
RG 196.92
DW 20.800 usec
DE 6.50 usec
TE 296.9 K
D1 2.00000000 sec
D11 0.03000000 sec
TD0 1

======== CHANNEL f1 ========
SFO1 100.6228298 MHz
NUC1 13C
P1 9.70 usec
PLW1 46.98899841 W

======== CHANNEL f2 ========
SFO2 400.1316005 MHz
NUC2 1H
CPDPRG[2 waltz16
PCPD2 90.00 usec
PLW2 11.99499989 W
PLW12 0.34213999 W
PLW13 0.27713001 W

F2 - Processing parameters
SI 32768
SF 100.6127593 MHz
WDW EM
SSB 0
LB 1.00 Hz
GB 0
PC 1.40

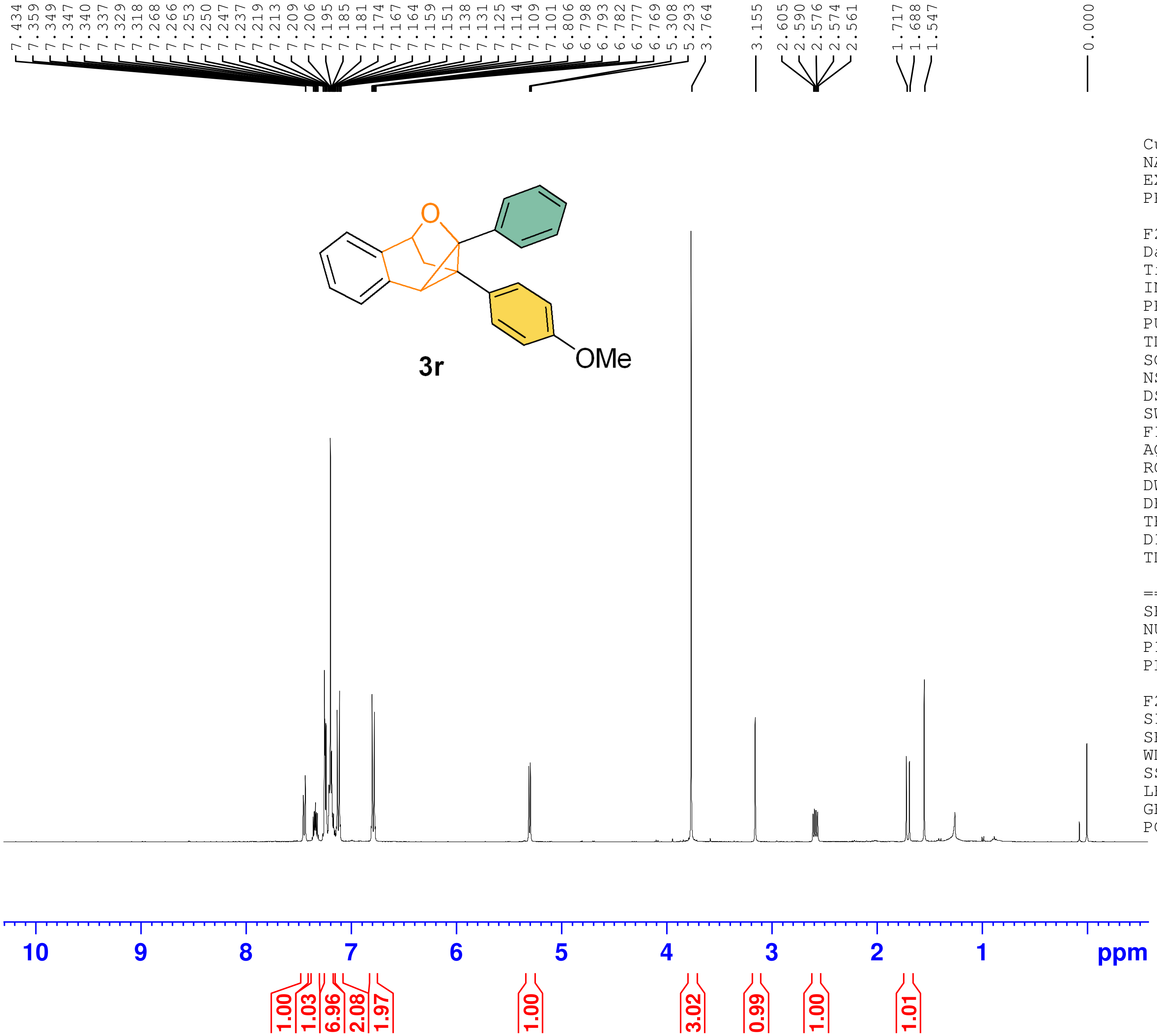

Current Data Parameters
NAME lsx-8-143aa-h-fr
EXPNO 1
PROCNO 1

F2 - Acquisition Parameters
Date_ 20240722
Time 19.00
INSTRUM spect
PROBHD 5 mm PABBO BB/
PULPROG zg30
TD 65536
SOLVENT CDCl3
NS 16
DS 2
SWH 8012.820 Hz
FIDRES 0.122266 Hz
AQ 4.0894465 sec
RG 164.33
DW 62.400 usec
DE 6.50 usec
TE 295.9 K
D1 1.00000000 sec
TD0 1

======== CHANNEL f1 ========
SFO1 400.1324710 MHz
NUC1 1H
P1 14.50 usec
PLW1 11.99499989 W

F2 - Processing parameters
SI 65536
SF 400.1300124 MHz
WDW EM
SSB 0
LB 0.30 Hz
GB 0
PC 1.00

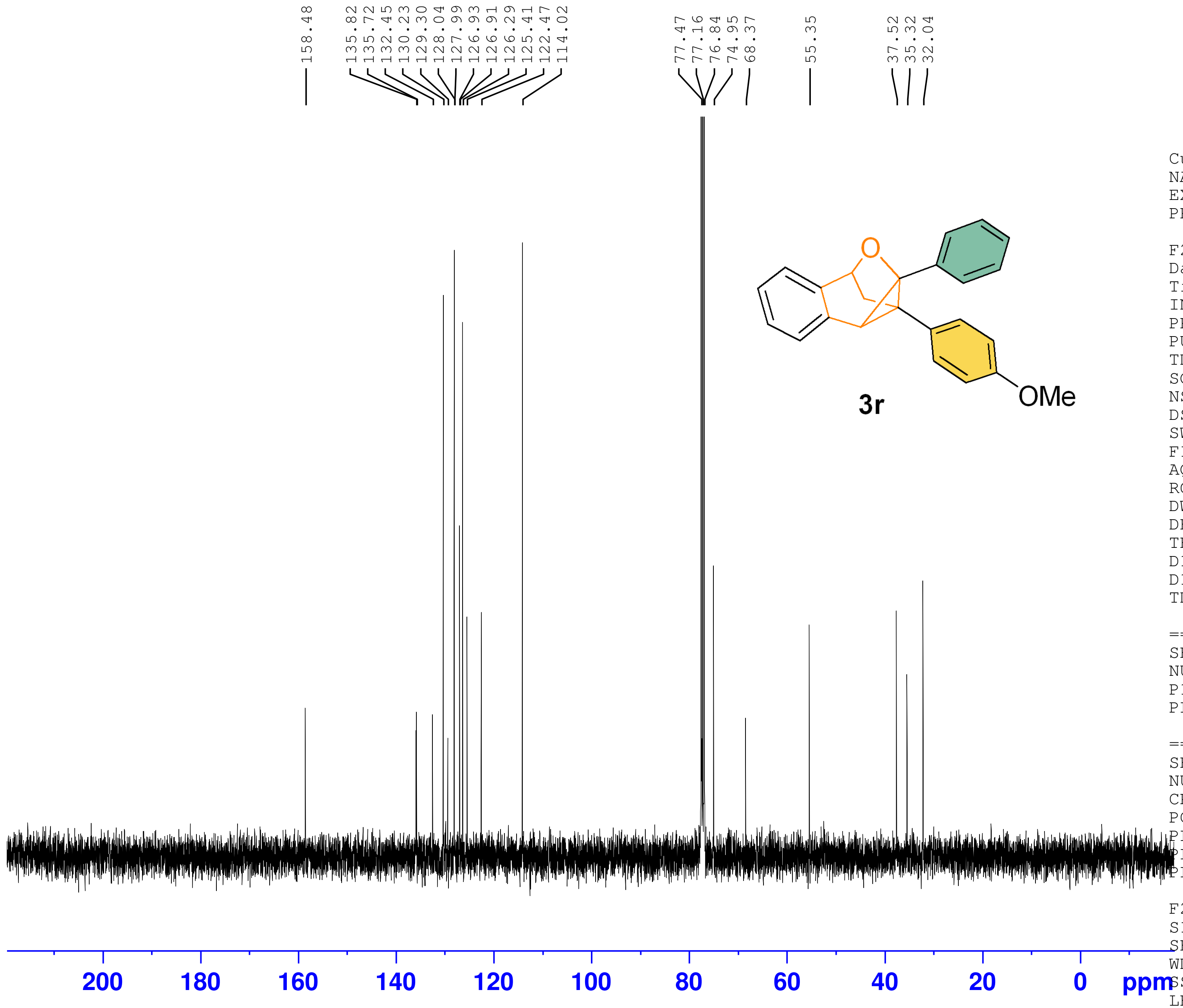


```
Current Data Parameters
NAME        lsx-8-143aa-c-fr
EXPNO                      1
PROCNO                     1

F2 - Acquisition Parameters
Date_               20240722
Time                   19.17
INSTRUM                spect
PROBHD    5 mm PABBO BB/
PULPROG              zgpg30
TD                     65536
SOLVENT                CDCl3
NS                       268
DS                         2
SWH             24038.461 Hz
FIDRES           0.366798 Hz
AQ              1.3631488 sec
RG                    196.92
DW                    20.800 usec
DE                      6.50 usec
TE                     297.0 K
D1            2.00000000 sec
D11           0.03000000 sec
TD0                        1

======== CHANNEL f1 ========
SFO1         100.6228298 MHz
NUC1                     13C
P1                      9.70 usec
PLW1         46.98899841 W

======== CHANNEL f2 ========
SFO2         400.1316005 MHz
NUC2                      1H
CPDPRG[2             waltz16
PCPD2                  90.00 usec
PLW2         11.99499989 W
PLW12         0.34213999 W
PLW13         0.27713001 W

F2 - Processing parameters
SI                     32768
SF           100.6127560 MHz
WDW                       EM
SSB                        0
LB                      1.00 Hz
GB                         0
PC                      1.40
```

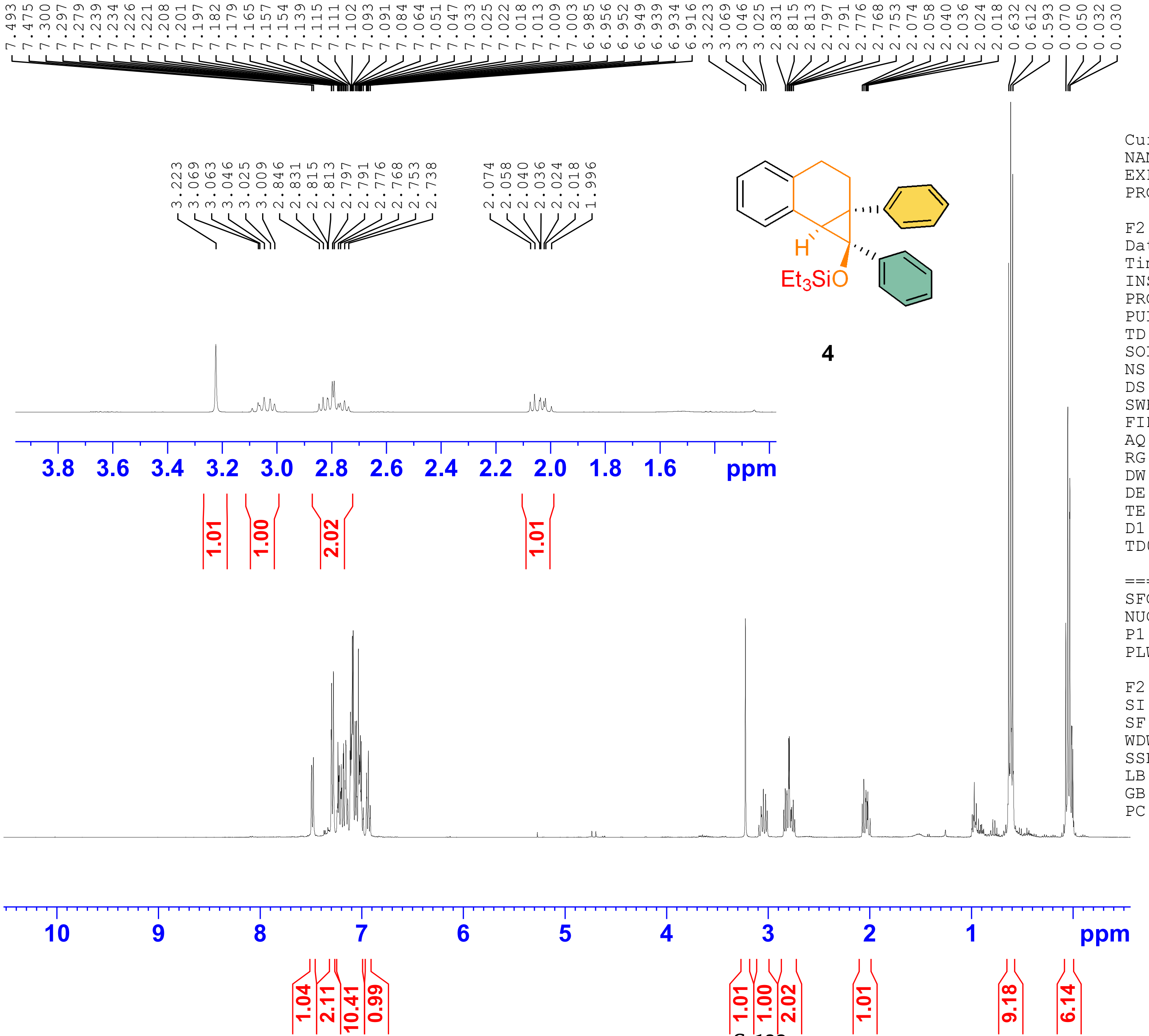


```
Current Data Parameters
NAME       lsx-2-144-h-fr
EXPNO                   1
PROCNO                  1

F2 - Acquisition Parameters
Date_            20220805
Time                19.18
INSTRUM             spect
PROBHD    5 mm PABBO BB/
PULPROG              zg30
TD                  65536
SOLVENT             CDCl3
NS                     16
DS                      2
SWH            8012.820 Hz
FIDRES         0.122266 Hz
AQ            4.0894465 sec
RG                  31.55
DW               62.400 usec
DE                 6.50 usec
TE                296.1 K
D1           1.00000000 sec
TD0                     1

======== CHANNEL f1 ========
SFO1         400.1324710 MHz
NUC1                   1H
P1                  14.50 usec
PLW1          11.99499989 W

F2 - Processing parameters
SI                  65536
SF           400.1300206 MHz
WDW                    EM
SSB                     0
LB                   0.30 Hz
GB                      0
PC                   1.00
```

144.01
141.45
137.88
133.19
130.74
128.68
128.46
128.37
127.67
127.38
126.72
125.90
125.54
125.44
77.32
77.00
76.68
73.02
37.95
28.66
28.62
27.64
6.77
5.25

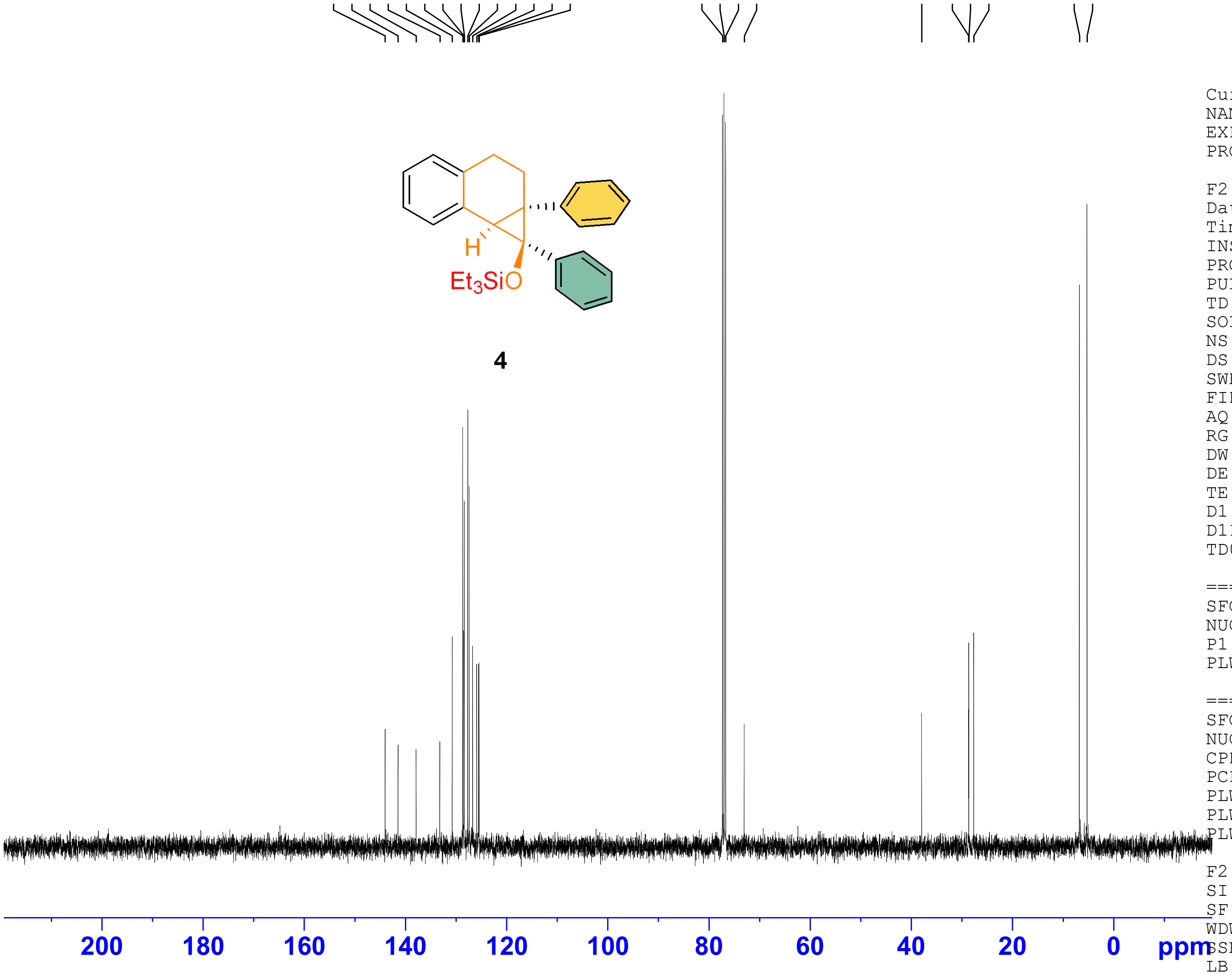

4

```
Current Data Parameters
NAME      lsx-2-144-cc-fr
EXPNO                   1
PROCNO                  1

F2 - Acquisition Parameters
Date_            20220805
Time                19.25
INSTRUM             spect
PROBHD    5 mm PABBO BB/
PULPROG           zgpg30
TD                 65536
SOLVENT            CDCl3
NS                    52
DS                     2
SWH            24038.461 Hz
FIDRES          0.366798 Hz
AQ             1.3631488 sec
RG                196.92
DW                20.800 usec
DE                  6.50 usec
TE                 296.7 K
D1            2.00000000 sec
D11           0.03000000 sec
TD0                    1

======== CHANNEL f1 ========
SFO1         100.6228298 MHz
NUC1                 13C
P1                  9.70 usec
PLW1         46.98899841 W

======== CHANNEL f2 ========
SFO2         400.1316005 MHz
NUC2                  1H
CPDPRG[2         waltz16
PCPD2              90.00 usec
PLW2         11.99499989 W
PLW12         0.34213999 W
PLW13         0.27713001 W

F2 - Processing parameters
SI                 32768
SF           100.6127737 MHz
WDW                   EM
SSB                    0
LB                  1.00 Hz
GB                     0
PC                  1.40
```

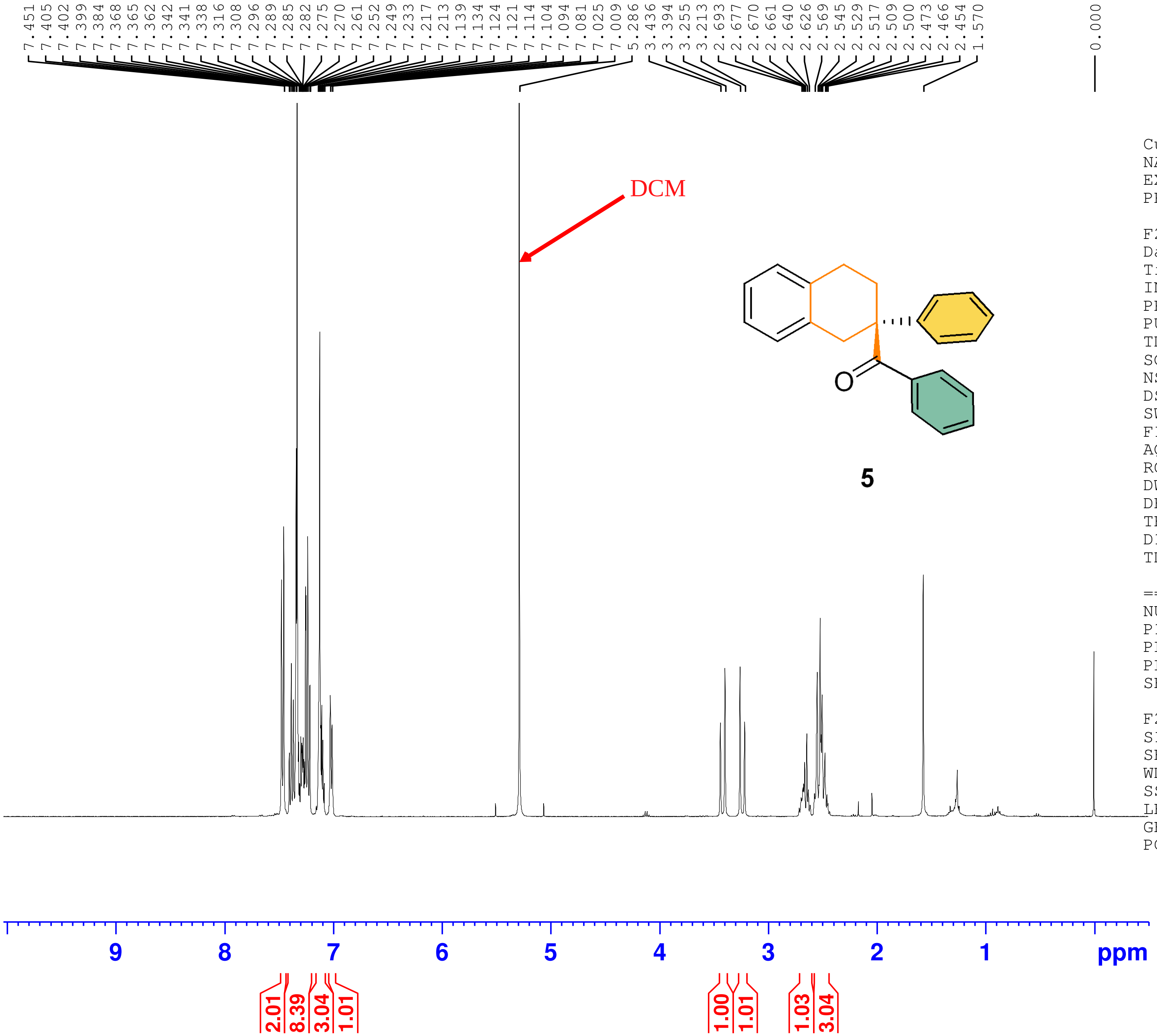


```
Current Data Parameters
NAME        lsx-8-tbaf-h-fr
EXPNO                     1
PROCNO                    1

F2 - Acquisition Parameters
Date_              20240713
Time                  19.24
INSTRUM               spect
PROBHD    5 mm DUL 13C-1
PULPROG                zg30
TD                    65536
SOLVENT               CDCl3
NS                       16
DS                        2
SWH              8223.685 Hz
FIDRES           0.125483 Hz
AQ              3.9845889 sec
RG                      362
DW                 60.800 usec
DE                   6.00 usec
TE                  296.7 K
D1             1.00000000 sec
TD0                       1

======== CHANNEL f1 ========
NUC1                     1H
P1                  15.80 usec
PL1                 -1.00 dB
PL1W          12.17476940 W
SFO1          400.1324710 MHz

F2 - Processing parameters
SI                    32768
SF            400.1300141 MHz
WDW                      EM
SSB                       0
LB                     0.30 Hz
GB                        0
PC                     1.00
```

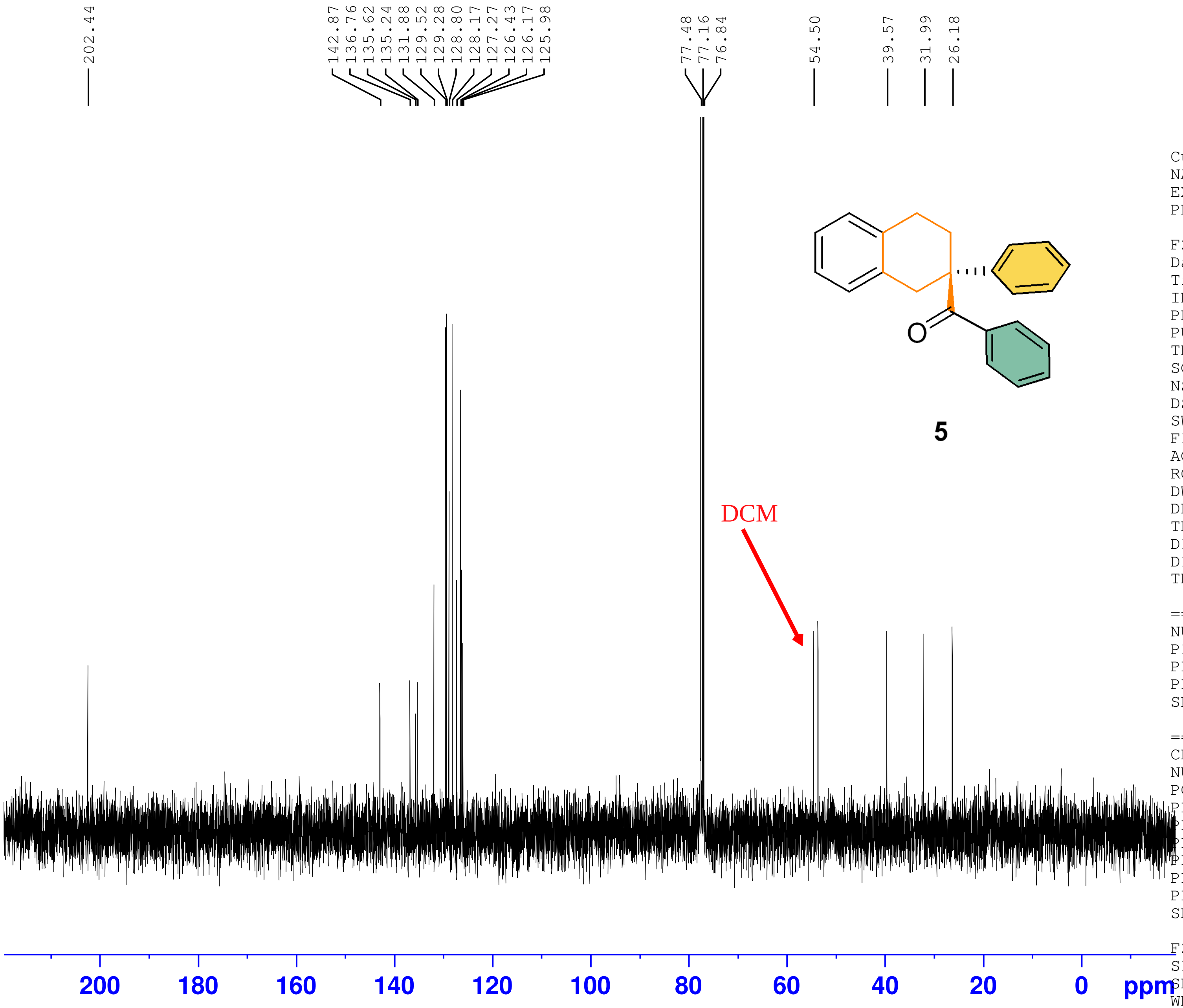

5

```
Current Data Parameters
NAME       lsx-8-tbaf-c-fr
EXPNO                    1
PROCNO                   1

F2 - Acquisition Parameters
Date_             20240713
Time                 19.36
INSTRUM              spect
PROBHD    5 mm DUL 13C-1
PULPROG            zgpg30
TD                  65536
SOLVENT             CDCl3
NS                    181
DS                      1
SWH             24038.461 Hz
FIDRES           0.366798 Hz
AQ              1.3631488 sec
RG                   80.6
DW                 20.800 usec
DE                   6.00 usec
TE                  297.0 K
D1             2.00000000 sec
D11            0.03000000 sec
TD0                     1

======== CHANNEL f1 ========
NUC1                  13C
P1                  40.00 usec
PL1                 -3.00 dB
PL1W          60.64365387 W
SFO1          100.6228298 MHz

======== CHANNEL f2 ========
CPDPRG[2          waltz16
NUC2                   1H
PCPD2               80.00 usec
PL2                 -1.00 dB
PL12                14.39 dB
PL13                18.00 dB
PL2W          12.17476940 W
PL12W          0.35193357 W
PL13W          0.15327126 W
SFO2          400.1316005 MHz

F2 - Processing parameters
SI                  32768
SF            100.6127572 MHz
WDW                    EM
SSB                     0
LB                   1.00 Hz
GB                      0
PC                   1.40
```

Data File C:\DATA\202207-12\SUN_12 2022-08-01 21-33-13\LSX-P3-3A-RAC.D
Sample Name:

```
=====================================================================
Acq. Operator   :                               Seq. Line :    5
Acq. Instrument : Instrument 1                   Location :   41
Injection Date  : 1/8/2022 7:59:55 am                 Inj :    1
                                               Inj Volume : 5.000 µl
Acq. Method     : C:\Chem32\1\DATA\SUN_12 2022-08-01 21-33-13\IC-02-30.M
Last changed    :
                  (modified after loading)
Analysis Method : C:\Users\Public\Documents\ChemStation\1\Data\SUN\SUN 2024-03-13 00-13-49\AD-01
                  -30-0.5.M (Sequence Method)
Last changed    : 13/3/2024 6:46:32 pm by SYSTEM
                  (modified after loading)
Additional Info : Peak(s) manually integrated
```

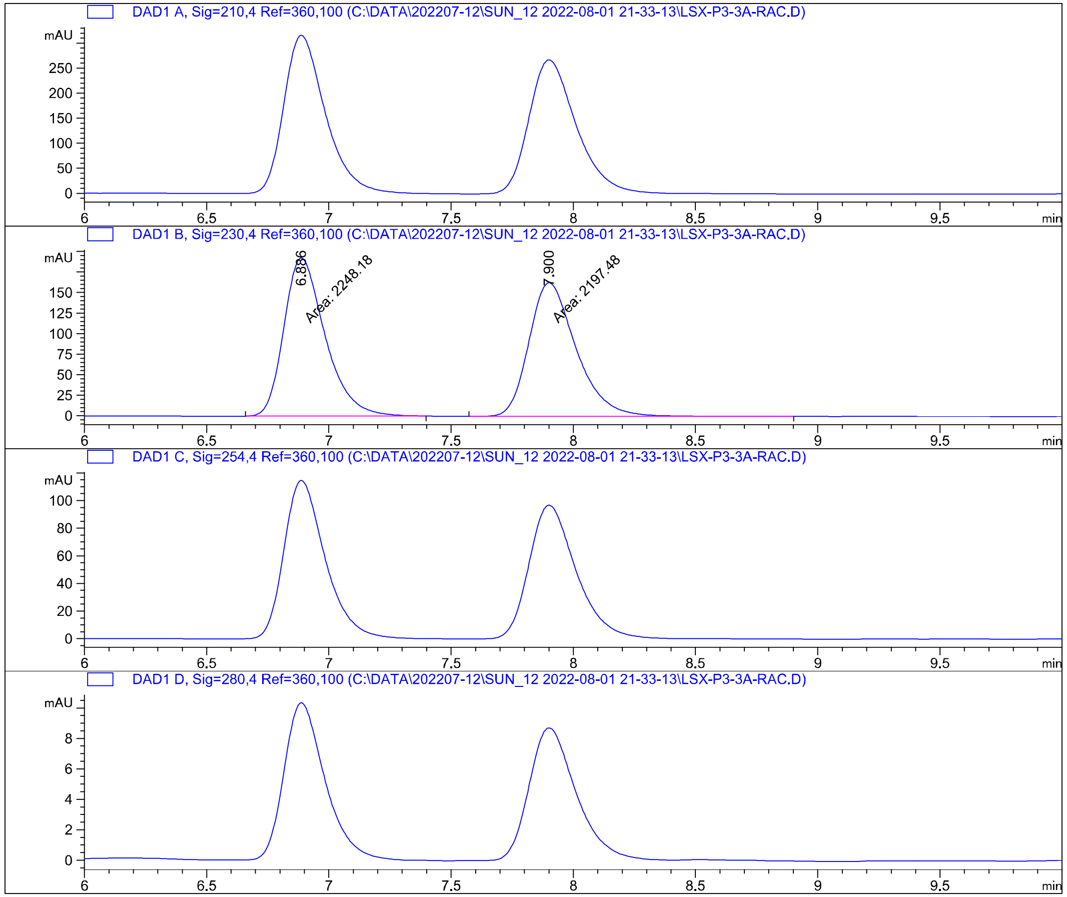

Data File C:\DATA\202207-12\SUN_12 2022-08-01 21-33-13\LSX-P3-3A-RAC.D
Sample Name:

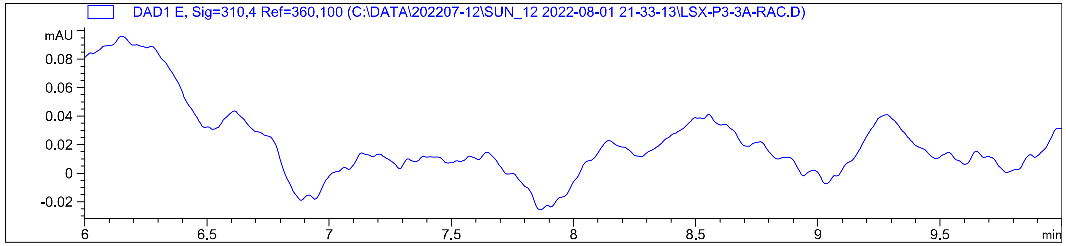


```
=====================================================================
                         Area Percent Report
=====================================================================

Sorted By             :      Signal
Multiplier            :      1.0000
Dilution              :      1.0000
Use Multiplier & Dilution Factor with ISTDs


Signal 1: DAD1 A, Sig=210,4 Ref=360,100


Signal 2: DAD1 B, Sig=230,4 Ref=360,100
```

Peak #	RetTime [min]	Type	Width [min]	Area [mAU*s]	Height [mAU]	Area %
1	6.886	MM	0.1945	2248.17627	192.65378	50.5702
2	7.900	MM	0.2246	2197.47681	163.04517	49.4298

Totals : 4445.65308 355.69894

```
Signal 3: DAD1 C, Sig=254,4 Ref=360,100


Signal 4: DAD1 D, Sig=280,4 Ref=360,100


Signal 5: DAD1 E, Sig=310,4 Ref=360,100

=====================================================================
                        *** End of Report ***
```

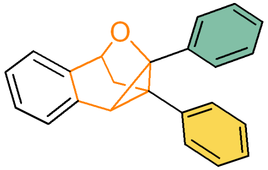

rac-3a

Data File C:\DATA\202109-1231\SUN_12 2021-12-12 20-18-38\LSX-1-146.D
Sample Name:

===

Acq. Operator	:	Seq. Line :	2
Acq. Instrument	: Instrument 1	Location :	41
Injection Date	: 12/12/2021 4:31:25 am	Inj :	1
		Inj Volume :	5.000 µl

Acq. Method : C:\Chem32\1\DATA\SUN_12 2021-12-12 20-18-38\IC-02-30.M
Last changed :
(modified after loading)
Analysis Method : C:\Users\Public\Documents\ChemStation\1\Data\SUN\SUN 2024-03-13 00-13-49\AD-01-30-0.5.M (Sequence Method)
Last changed : 13/3/2024 6:43:39 pm by SYSTEM
(modified after loading)
Additional Info : Peak(s) manually integrated

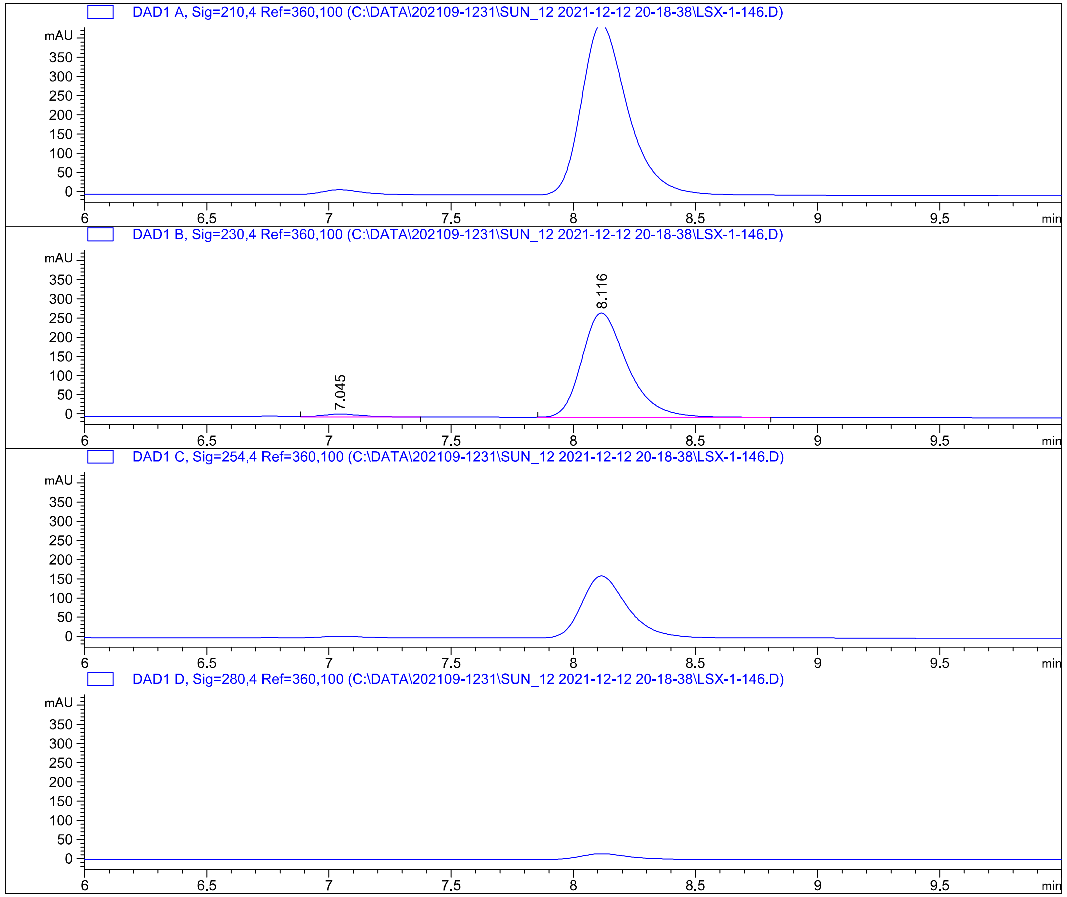

Data File C:\DATA\202109-1231\SUN_12 2021-12-12 20-18-38\LSX-1-146.D
Sample Name:

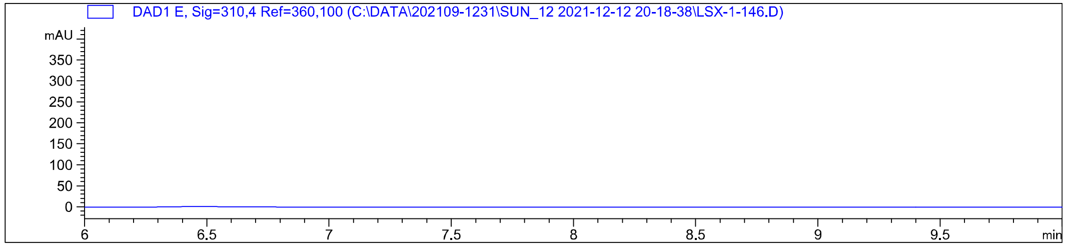

===
Area Percent Report
===

Sorted By : Signal
Multiplier : 1.0000
Dilution : 1.0000
Use Multiplier & Dilution Factor with ISTDs

Signal 1: DAD1 A, Sig=210,4 Ref=360,100

Signal 2: DAD1 B, Sig=230,4 Ref=360,100

Peak #	RetTime [min]	Type	Width [min]	Area [mAU*s]	Height [mAU]	Area %
1	7.045	VB	0.1691	79.63106	7.19634	2.2089
2	8.116	BB	0.1972	3525.37769	272.00723	97.7911

Totals : 3605.00874 279.20358

Signal 3: DAD1 C, Sig=254,4 Ref=360,100

Signal 4: DAD1 D, Sig=280,4 Ref=360,100

Signal 5: DAD1 E, Sig=310,4 Ref=360,100

===
*** End of Report ***

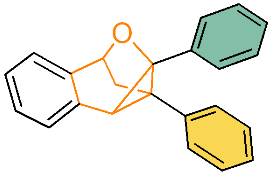

3a

Data File C:\DATA\202207-12\SUN_12 2022-07-24 16-49-13\LSX-PROJECT3-3H-RAC.D
Sample Name:

===

```
Acq. Operator    :                                  Seq. Line :    6
Acq. Instrument  : Instrument 1                      Location :   45
Injection Date   : 24/7/2022 4:06:57 am                   Inj :    1
                                                   Inj Volume : 5.000 µl
Acq. Method      : C:\Chem32\1\DATA\SUN_12 2022-07-24 16-49-13\IC-02-30.M
Last changed     :
                   (modified after loading)
Analysis Method  : C:\Users\Public\Documents\ChemStation\1\Data\SUN\SUN 2024-03-13 17-26-47\OD-00
                   -20.M (Sequence Method)
Last changed     : 13/3/2024 10:28:03 pm by SYSTEM
                   (modified after loading)
Additional Info  : Peak(s) manually integrated
```

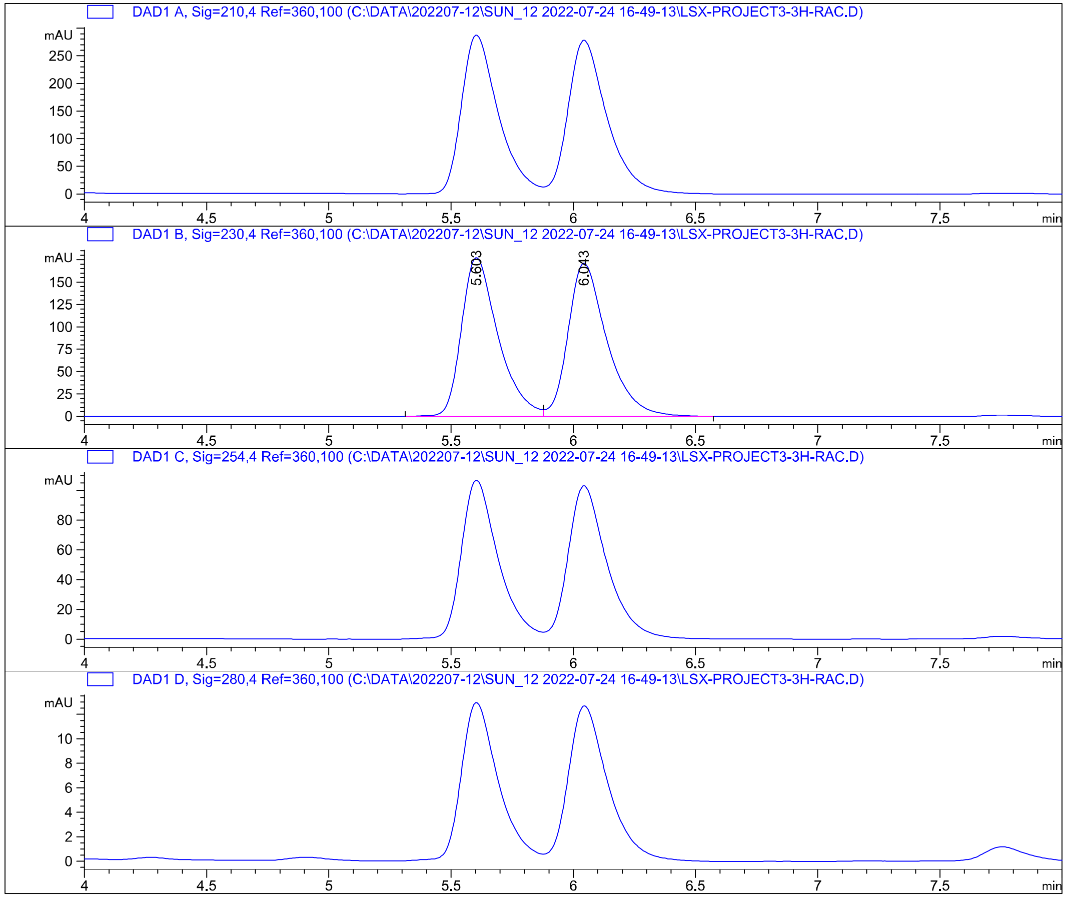

Data File C:\DATA\202207-12\SUN_12 2022-07-24 16-49-13\LSX-PROJECT3-3H-RAC.D
Sample Name:

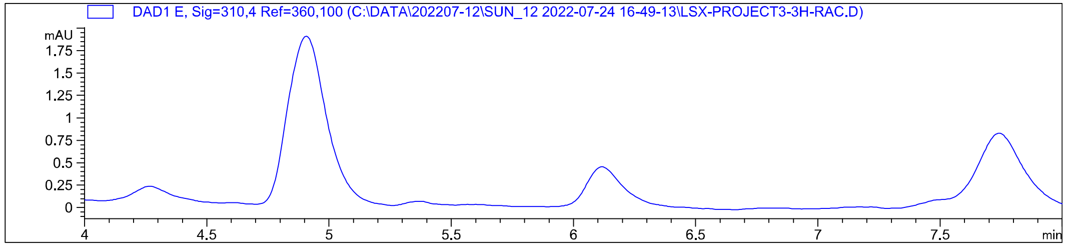

===

Area Percent Report

===

```
Sorted By             :      Signal
Multiplier            :      1.0000
Dilution              :      1.0000
Use Multiplier & Dilution Factor with ISTDs
```

Signal 1: DAD1 A, Sig=210,4 Ref=360,100

Signal 2: DAD1 B, Sig=230,4 Ref=360,100

Peak #	RetTime [min]	Type	Width [min]	Area [mAU*s]	Height [mAU]	Area %
1	5.603	BV	0.1560	1857.32861	177.67133	49.4033
2	6.043	VB	0.1653	1902.19141	171.62758	50.5967

Totals : 3759.52002 349.29890

Signal 3: DAD1 C, Sig=254,4 Ref=360,100

Signal 4: DAD1 D, Sig=280,4 Ref=360,100

Signal 5: DAD1 E, Sig=310,4 Ref=360,100

===

*** End of Report ***

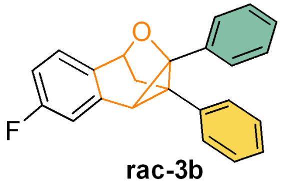

rac-3b

===

Acq. Operator : Seq. Line : 18
Acq. Instrument : Instrument 1 Location : 41
Injection Date : 2/1/2022 6:09:32 am Inj : 1
Inj Volume : 5.000 µl
Acq. Method : C:\Chem32\1\DATA\SUN_12 2022-01-02 17-11-59\IC-02-30.M
Last changed :
(modified after loading)
Analysis Method : C:\Users\Public\Documents\ChemStation\1\Data\SUN\SUN 2024-03-13 17-26-47\OD-00-20.M (Sequence Method)
Last changed : 13/3/2024 10:29:02 pm by SYSTEM
(modified after loading)
Additional Info : Peak(s) manually integrated

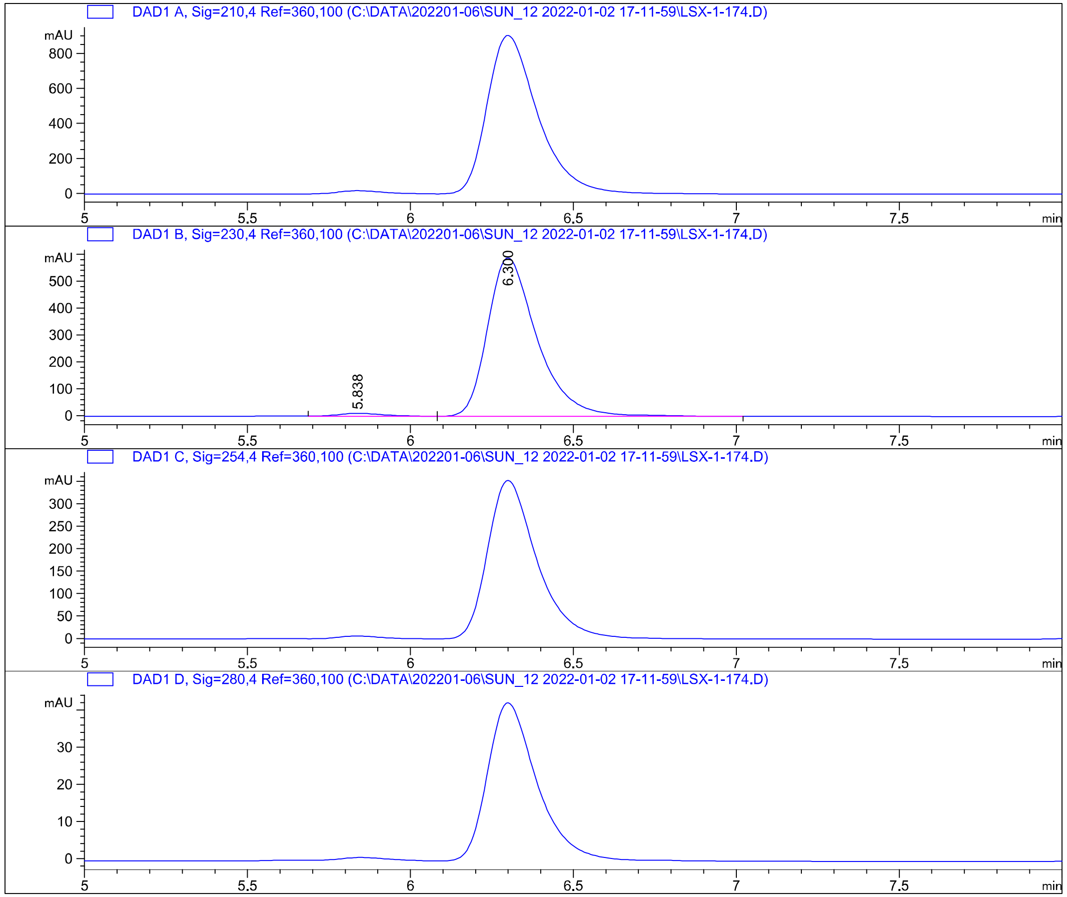

Data File C:\DATA\202201-06\SUN_12 2022-01-02 17-11-59\LSX-1-174.D
Sample Name:

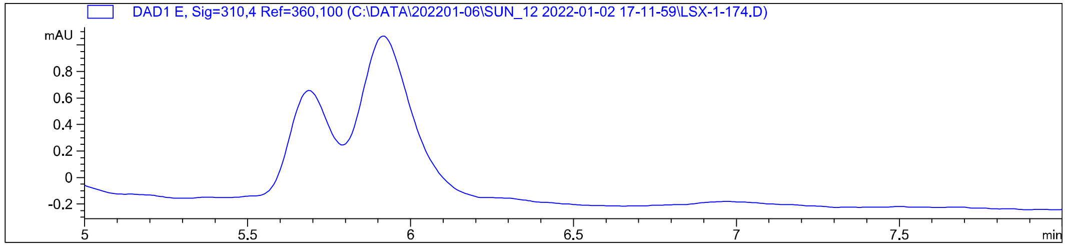

===

Area Percent Report

===

Sorted By : Signal
Multiplier : 1.0000
Dilution : 1.0000
Use Multiplier & Dilution Factor with ISTDs

Signal 1: DAD1 A, Sig=210,4 Ref=360,100

Signal 2: DAD1 B, Sig=230,4 Ref=360,100

Peak #	RetTime [min]	Type	Width [min]	Area [mAU*s]	Height [mAU]	Area %
1	5.838	VV	0.1522	111.83696	11.04016	1.7620
2	6.300	VB	0.1594	6235.43604	589.52393	98.2380
Totals :				6347.27299	600.56408	

Signal 3: DAD1 C, Sig=254,4 Ref=360,100

Signal 4: DAD1 D, Sig=280,4 Ref=360,100

Signal 5: DAD1 E, Sig=310,4 Ref=360,100

===

*** End of Report ***

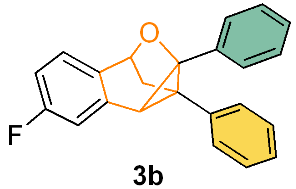

3b

Data File C:\DATA\202207-12\SUN_12 2022-07-24 16-49-13\LSX-PROJECT3-3I-RAC.D
Sample Name:

Acq. Operator	:	Seq. Line :	7
Acq. Instrument	: Instrument 1	Location :	46
Injection Date	: 24/7/2022 4:37:58 am	Inj :	1
		Inj Volume :	5.000 µl

Acq. Method : C:\Chem32\1\DATA\SUN_12 2022-07-24 16-49-13\IC-02-30.M
Last changed :
(modified after loading)
Analysis Method : C:\Users\Public\Documents\ChemStation\1\Data\SUN\SUN 2024-03-13 17-26-47\OD-00-20.M (Sequence Method)
Last changed : 13/3/2024 10:32:00 pm by SYSTEM
(modified after loading)
Additional Info : Peak(s) manually integrated

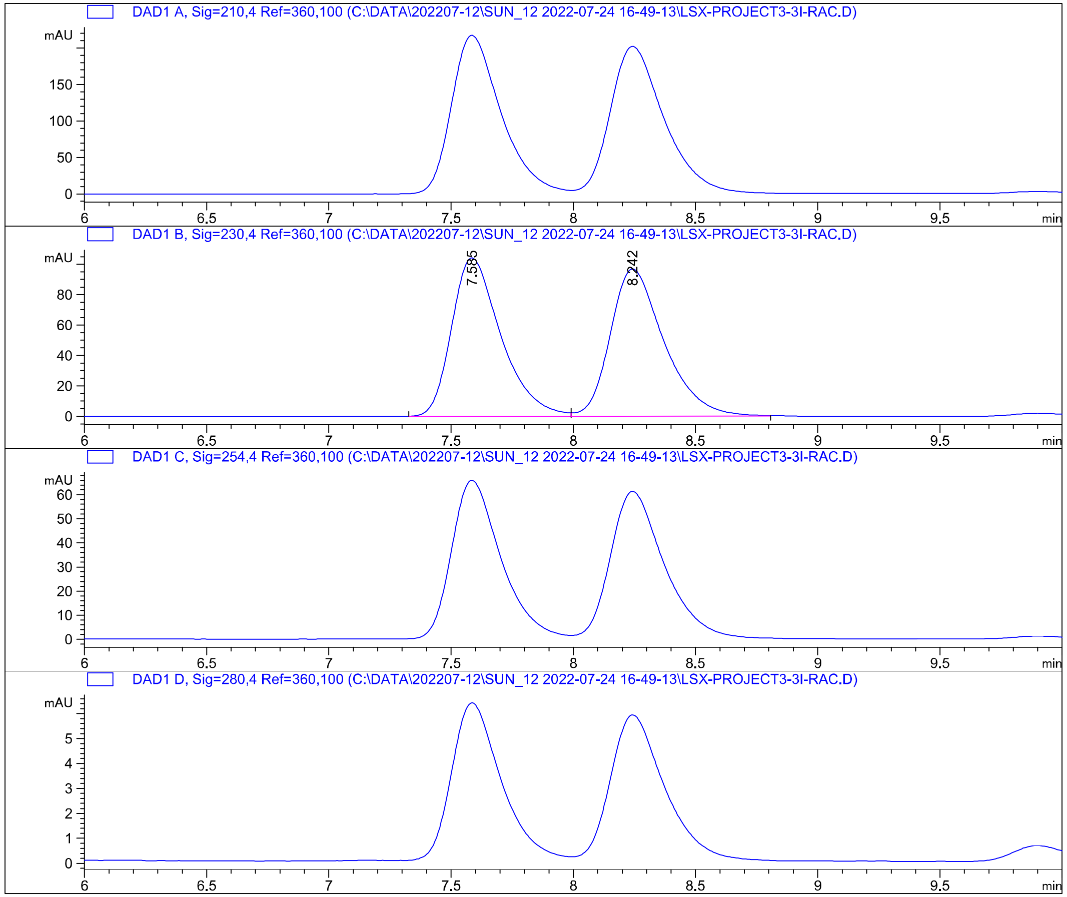

Data File C:\DATA\202207-12\SUN_12 2022-07-24 16-49-13\LSX-PROJECT3-3I-RAC.D
Sample Name:

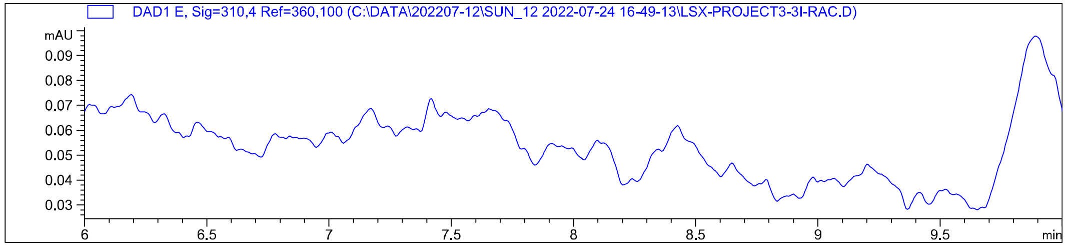

Area Percent Report

Sorted By : Signal
Multiplier : 1.0000
Dilution : 1.0000
Use Multiplier & Dilution Factor with ISTDs

Signal 1: DAD1 A, Sig=210,4 Ref=360,100

Signal 2: DAD1 B, Sig=230,4 Ref=360,100

Peak #	RetTime [min]	Type	Width [min]	Area [mAU*s]	Height [mAU]	Area %
1	7.585	BV	0.2117	1462.05786	104.22356	49.6991
2	8.242	VB	0.2283	1479.75928	96.82571	50.3009
Totals :				2941.81714	201.04926	

Signal 3: DAD1 C, Sig=254,4 Ref=360,100

Signal 4: DAD1 D, Sig=280,4 Ref=360,100

Signal 5: DAD1 E, Sig=310,4 Ref=360,100

*** End of Report ***

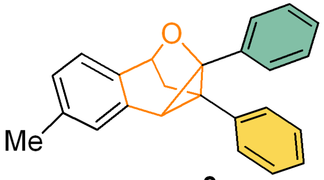

rac-3c

Data File C:\DATA\202201-06\SUN_12 2022-01-22 14-41-42\LSX-2-9C.D
Sample Name:

===

```
Acq. Operator   :                              Seq. Line :    4
Acq. Instrument : Instrument 1                  Location :   43
Injection Date  : 21/1/2022 11:56:27 pm              Inj :    1
                                              Inj Volume : 5.000 µl
Acq. Method     : C:\Chem32\1\DATA\SUN_12 2022-01-22 14-41-42\IC-02-30.M
Last changed    :
                  (modified after loading)
Analysis Method : C:\Users\Public\Documents\ChemStation\1\Data\SUN\SUN 2024-03-13 17-26-47\OD-00
                  -20.M (Sequence Method)
Last changed    : 13/3/2024 10:32:00 pm by SYSTEM
                  (modified after loading)
Additional Info : Peak(s) manually integrated
```

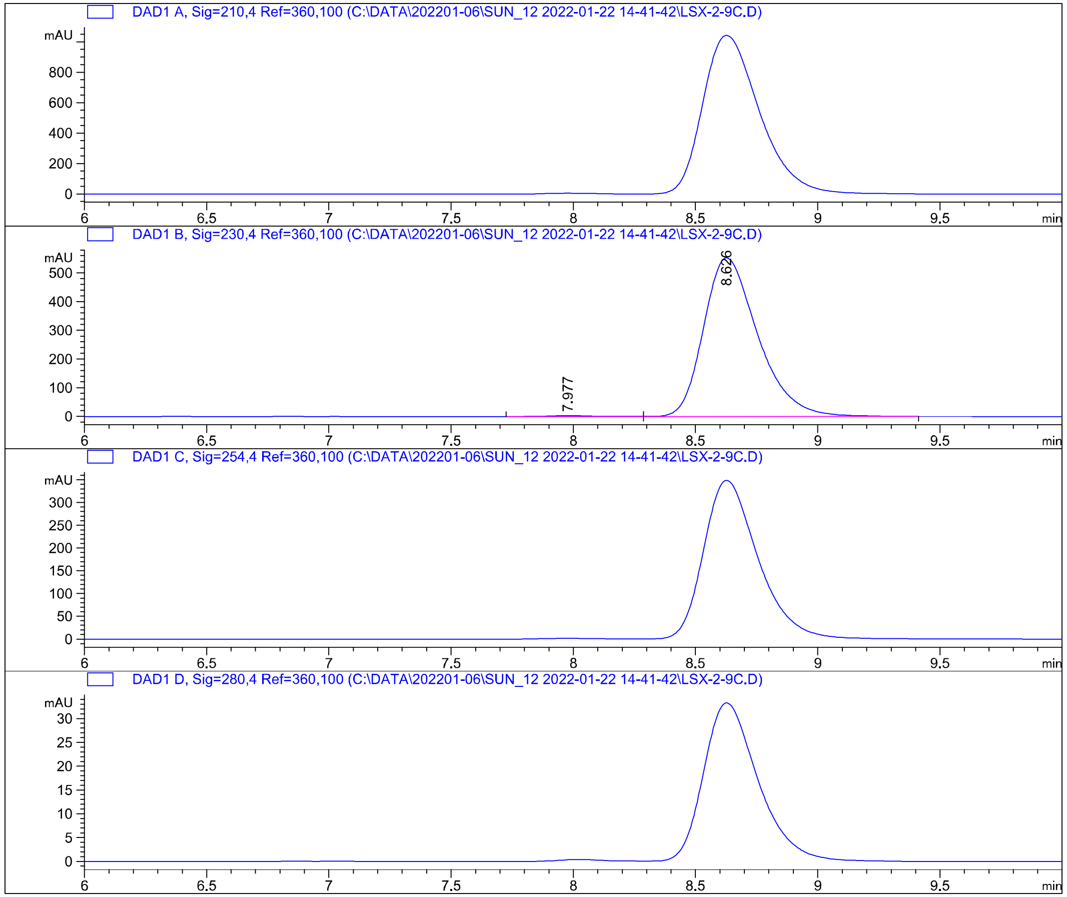

Data File C:\DATA\202201-06\SUN_12 2022-01-22 14-41-42\LSX-2-9C.D
Sample Name:

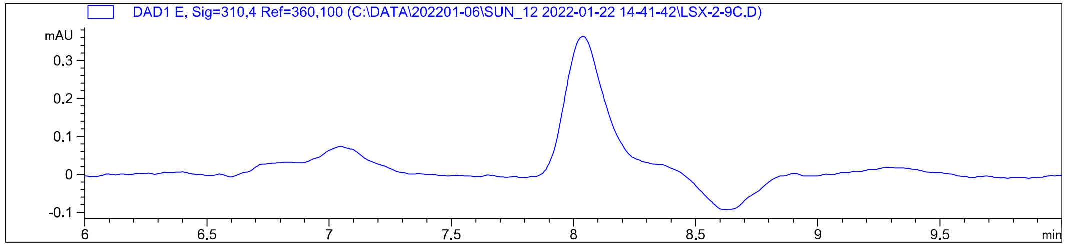

===
Area Percent Report
===

```
Sorted By             :      Signal
Multiplier            :      1.0000
Dilution              :      1.0000
Use Multiplier & Dilution Factor with ISTDs
```

Signal 1: DAD1 A, Sig=210,4 Ref=360,100

Signal 2: DAD1 B, Sig=230,4 Ref=360,100

Peak #	RetTime [min]	Type	Width [min]	Area [mAU*s]	Height [mAU]	Area %
1	7.977	BV	0.2237	41.06731	2.79093	0.4792
2	8.626	VB	0.2339	8529.00488	553.08282	99.5208

Totals : 8570.07219 555.87375

Signal 3: DAD1 C, Sig=254,4 Ref=360,100

Signal 4: DAD1 D, Sig=280,4 Ref=360,100

Signal 5: DAD1 E, Sig=310,4 Ref=360,100

===
*** End of Report ***

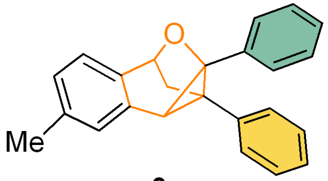

3c

Data File C:\DATA\202207-12\SUN_12 2022-07-24 16-49-13\LSX-PROJECT3-3J-RAC.D
Sample Name:

===

Acq. Operator	:	Seq. Line :	8
Acq. Instrument	: Instrument 1	Location :	47
Injection Date	: 24/7/2022 5:08:58 am	Inj :	1
		Inj Volume :	5.000 µl

Acq. Method : C:\Chem32\1\DATA\SUN_12 2022-07-24 16-49-13\IC-02-30.M
Last changed :
(modified after loading)
Analysis Method : C:\Users\Public\Documents\ChemStation\1\Data\SUN\SUN 2024-03-13 17-26-47\OD-00-20.M (Sequence Method)
Last changed : 13/3/2024 10:34:10 pm by SYSTEM
(modified after loading)
Additional Info : Peak(s) manually integrated

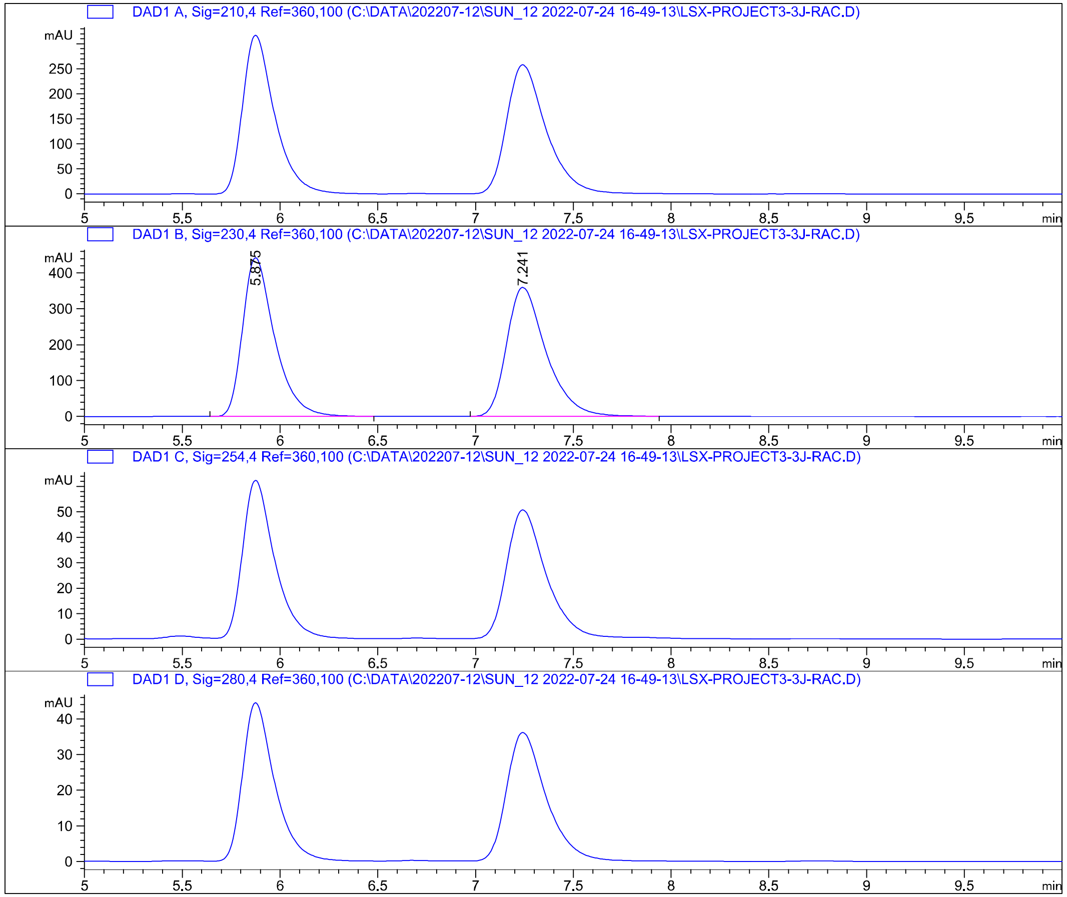

Data File C:\DATA\202207-12\SUN_12 2022-07-24 16-49-13\LSX-PROJECT3-3J-RAC.D
Sample Name:

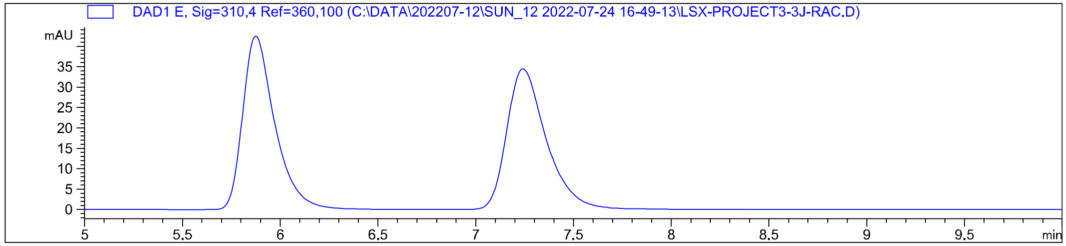

===
Area Percent Report
===

Sorted By : Signal
Multiplier : 1.0000
Dilution : 1.0000
Use Multiplier & Dilution Factor with ISTDs

Signal 1: DAD1 A, Sig=210,4 Ref=360,100

Signal 2: DAD1 B, Sig=230,4 Ref=360,100

Peak #	RetTime [min]	Type	Width [min]	Area [mAU*s]	Height [mAU]	Area %
1	5.875	VB	0.1710	5037.94092	441.99258	49.9651
2	7.241	BB	0.2118	5044.97656	359.28168	50.0349
Totals :				1.00829e4	801.27426	

Signal 3: DAD1 C, Sig=254,4 Ref=360,100

Signal 4: DAD1 D, Sig=280,4 Ref=360,100

Signal 5: DAD1 E, Sig=310,4 Ref=360,100

===
*** End of Report ***

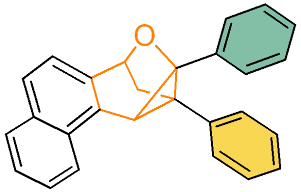

rac-3d

Data File C:\DATA\202201-06\SUN_12 2022-01-02 17-11-59\LSX-1-176.D
Sample Name:

===

Acq. Operator : Seq. Line : 19
Acq. Instrument : Instrument 1 Location : 42
Injection Date : 2/1/2022 6:40:32 am Inj : 1
Inj Volume : 5.000 µl
Acq. Method : C:\Chem32\1\DATA\SUN_12 2022-01-02 17-11-59\IC-02-30.M
Last changed :
(modified after loading)
Analysis Method : C:\Users\Public\Documents\ChemStation\1\Data\SUN\SUN 2024-03-13 17-26-47\OD-00-20.M (Sequence Method)
Last changed : 13/3/2024 10:37:58 pm by SYSTEM
(modified after loading)
Additional Info : Peak(s) manually integrated

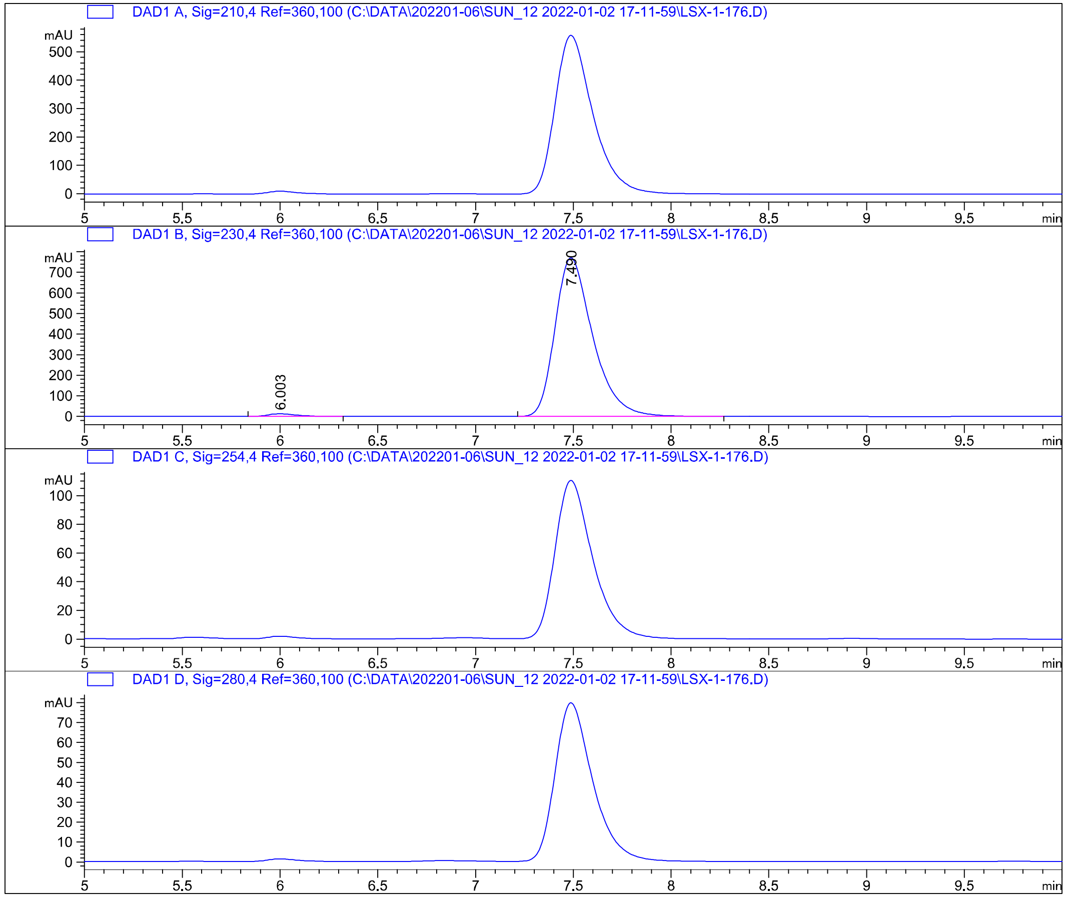

Data File C:\DATA\202201-06\SUN_12 2022-01-02 17-11-59\LSX-1-176.D
Sample Name:

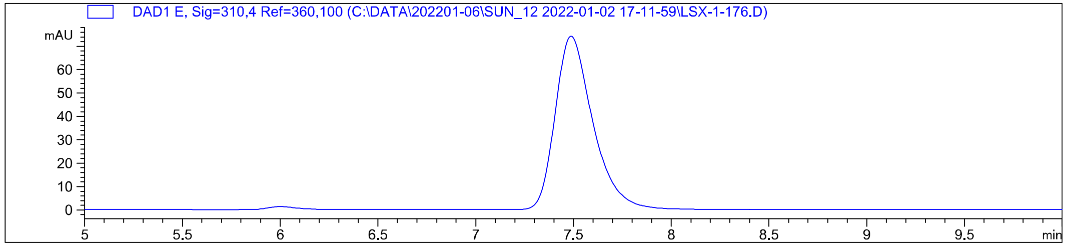

===

Area Percent Report

===

Sorted By : Signal
Multiplier : 1.0000
Dilution : 1.0000
Use Multiplier & Dilution Factor with ISTDs

Signal 1: DAD1 A, Sig=210,4 Ref=360,100

Signal 2: DAD1 B, Sig=230,4 Ref=360,100

Peak #	RetTime [min]	Type	Width [min]	Area [mAU*s]	Height [mAU]	Area %
1	6.003	BB	0.1590	135.40482	12.83902	1.2870
2	7.490	BB	0.2032	1.03853e4	770.76685	98.7130

Totals : 1.05207e4 783.60586

Signal 3: DAD1 C, Sig=254,4 Ref=360,100

Signal 4: DAD1 D, Sig=280,4 Ref=360,100

Signal 5: DAD1 E, Sig=310,4 Ref=360,100

===

*** End of Report ***

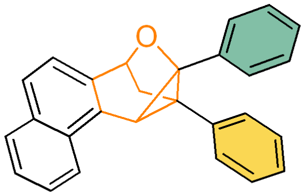

3d

```
Data File C:\DATA\202207-12\SUN_12 2022-08-01 21-33-13\LSX-P3-3K-RAC.D
Sample Name:

=====================================================================
Acq. Operator   :                               Seq. Line :    7
Acq. Instrument : Instrument 1                   Location :   44
Injection Date  : 1/8/2022 9:01:54 am                 Inj :    1
                                               Inj Volume : 5.000 µl
Acq. Method     : C:\Chem32\1\DATA\SUN_12 2022-08-01 21-33-13\IC-02-30.M
Last changed    :
                  (modified after loading)
Analysis Method : C:\Users\Public\Documents\ChemStation\1\Data\SUN\SUN 2024-03-13 00-13-49\AD-01
                  -30-0.5.M (Sequence Method)
Last changed    : 13/3/2024 6:50:29 pm by SYSTEM
                  (modified after loading)
Additional Info : Peak(s) manually integrated
```

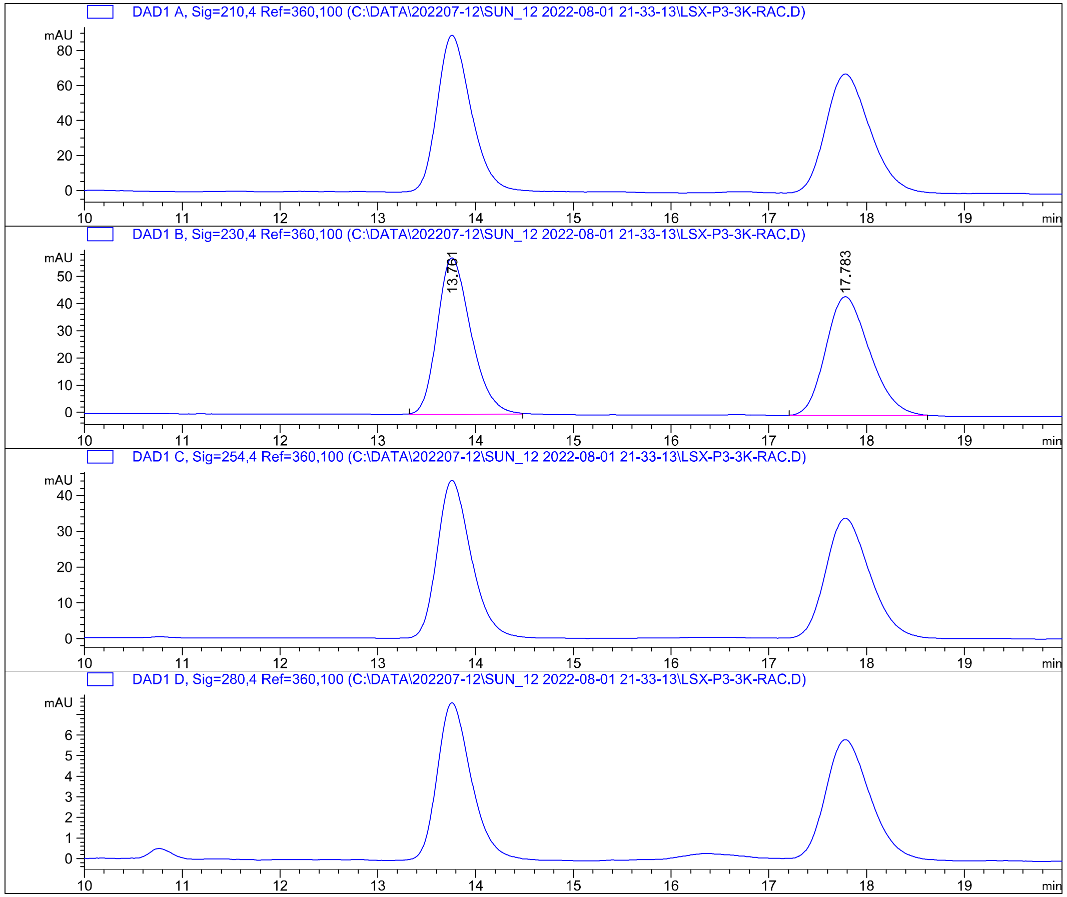


```
Data File C:\DATA\202207-12\SUN_12 2022-08-01 21-33-13\LSX-P3-3K-RAC.D
Sample Name:
```

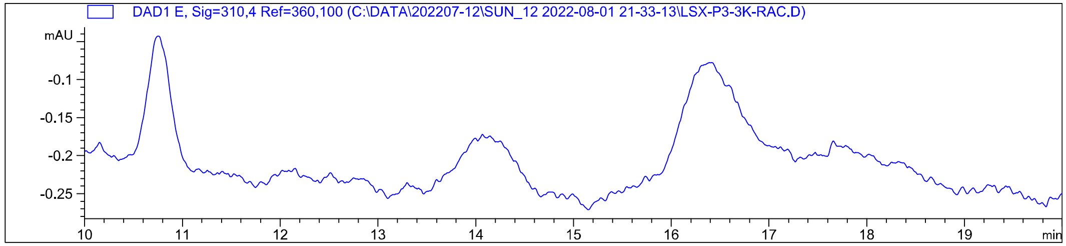


```
=====================================================================
                         Area Percent Report
=====================================================================

Sorted By             :      Signal
Multiplier            :      1.0000
Dilution              :      1.0000
Use Multiplier & Dilution Factor with ISTDs


Signal 1: DAD1 A, Sig=210,4 Ref=360,100


Signal 2: DAD1 B, Sig=230,4 Ref=360,100

Peak RetTime Type  Width     Area      Height     Area
  #   [min]        [min]   [mAU*s]     [mAU]        %
----|-------|----|-------|----------|----------|--------|
   1  13.761 BB    0.3678 1380.30212   57.54430  50.2088
   2  17.783 BB    0.4792 1368.82336   43.72640  49.7912

Totals :                  2749.12549  101.27070


Signal 3: DAD1 C, Sig=254,4 Ref=360,100


Signal 4: DAD1 D, Sig=280,4 Ref=360,100


Signal 5: DAD1 E, Sig=310,4 Ref=360,100

=====================================================================
                         *** End of Report ***
```

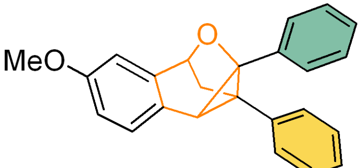

rac-3e

Data File C:\DATA\202201-06\SUN_12 2022-01-22 14-41-42\LSX-2-9D.D
Sample Name:

===

Acq. Operator : Seq. Line : 5
Acq. Instrument : Instrument 1 Location : 44
Injection Date : 22/1/2022 12:27:26 am Inj : 1
Inj Volume : 5.000 µl
Acq. Method : C:\Chem32\1\DATA\SUN_12 2022-01-22 14-41-42\IC-02-30.M
Last changed :
(modified after loading)
Analysis Method : C:\Users\Public\Documents\ChemStation\1\Data\SUN\SUN 2024-03-13 17-26-47\OD-00-20.M (Sequence Method)
Last changed : 13/3/2024 10:39:15 pm by SYSTEM
(modified after loading)
Additional Info : Peak(s) manually integrated

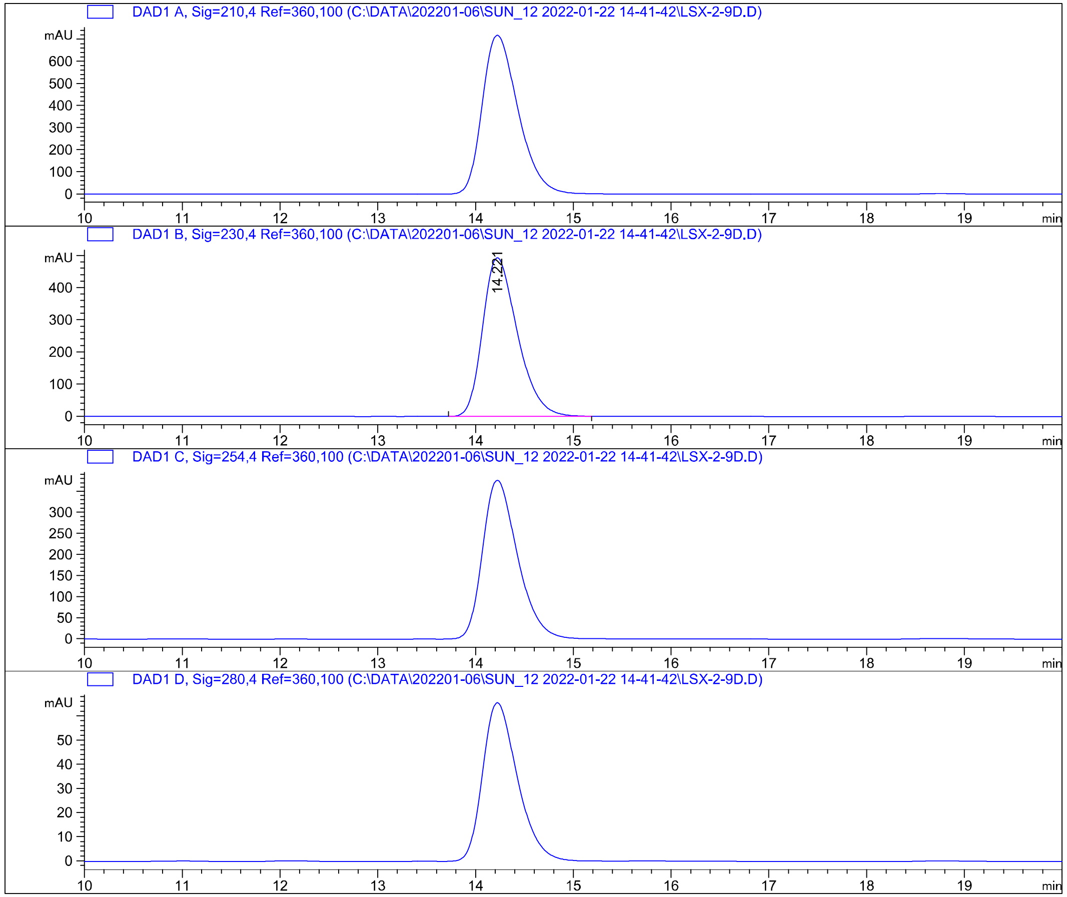

Data File C:\DATA\202201-06\SUN_12 2022-01-22 14-41-42\LSX-2-9D.D
Sample Name:

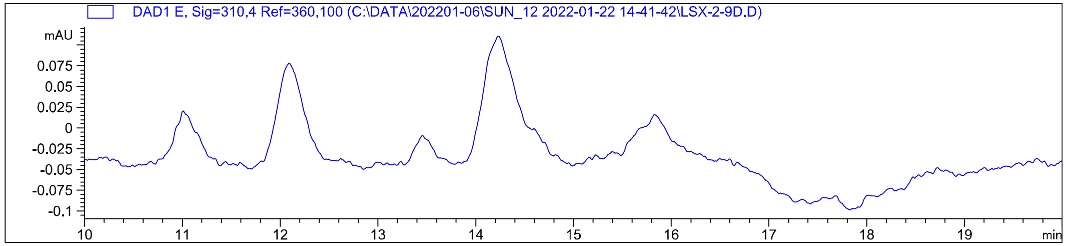

===

Area Percent Report

===

Sorted By : Signal
Multiplier : 1.0000
Dilution : 1.0000
Use Multiplier & Dilution Factor with ISTDs

Signal 1: DAD1 A, Sig=210,4 Ref=360,100

Signal 2: DAD1 B, Sig=230,4 Ref=360,100

Peak #	RetTime [min]	Type	Width [min]	Area [mAU*s]	Height [mAU]	Area %
1	14.221	BB	0.3809	1.22079e4	493.01736	100.0000
Totals :				1.22079e4	493.01736	

Signal 3: DAD1 C, Sig=254,4 Ref=360,100

Signal 4: DAD1 D, Sig=280,4 Ref=360,100

Signal 5: DAD1 E, Sig=310,4 Ref=360,100

===

*** End of Report ***

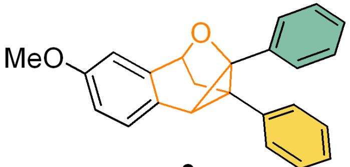

3e

Data File C:\DATA\202207-12\SUN_12 2022-07-24 16-49-13\LSX-PROJECT3-3B-RAC.D
Sample Name:

===
Acq. Operator   :   Seq. Line :   2
Acq. Instrument : Instrument 1   Location :   41
Injection Date : 24/7/2022 2:03:03 am   Inj :   1
                                          Inj Volume : 5.000 µl
Acq. Method   : C:\Chem32\1\DATA\SUN_12 2022-07-24 16-49-13\IC-02-30.M
Last changed   :
                  (modified after loading)
Analysis Method : C:\Users\Public\Documents\ChemStation\1\Data\SUN\SUN 2024-03-13 00-13-49\AD-01-30-0.5.M (Sequence Method)
Last changed   : 13/3/2024 6:54:01 pm by SYSTEM
                  (modified after loading)
Additional Info : Peak(s) manually integrated

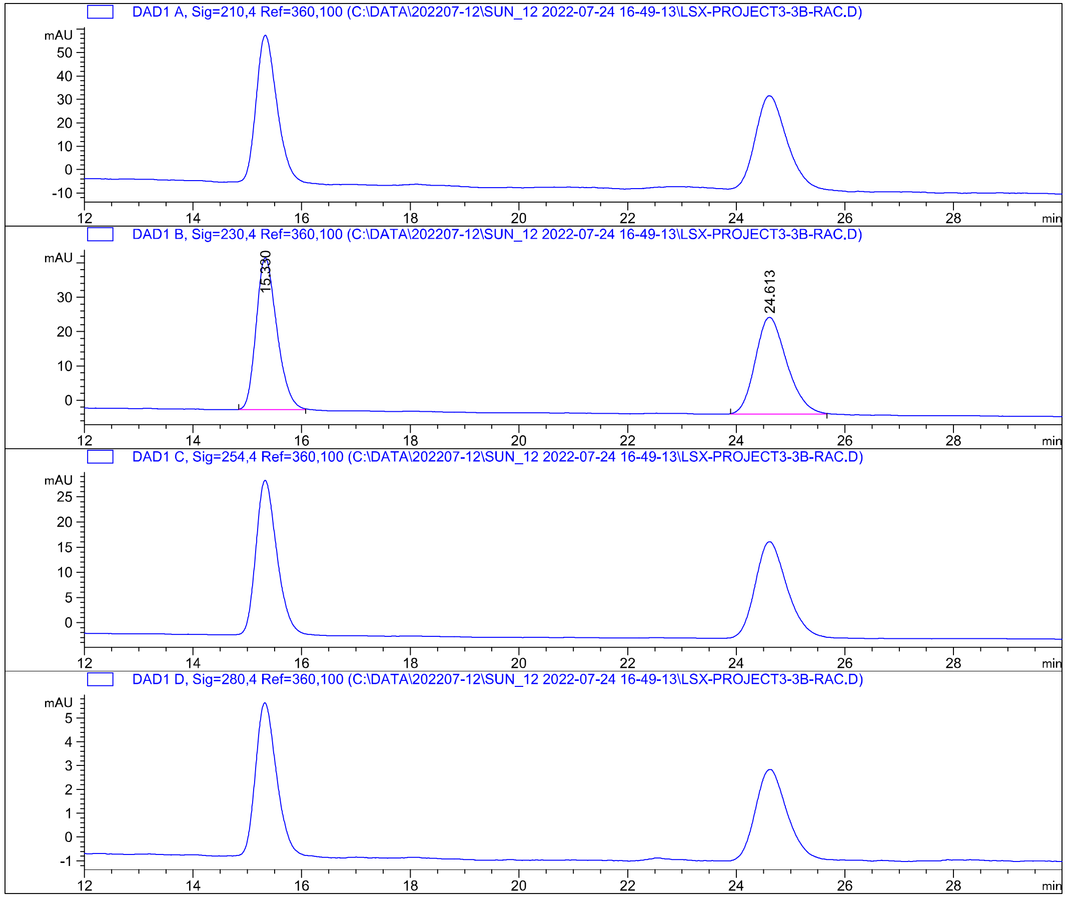

Data File C:\DATA\202207-12\SUN_12 2022-07-24 16-49-13\LSX-PROJECT3-3B-RAC.D
Sample Name:

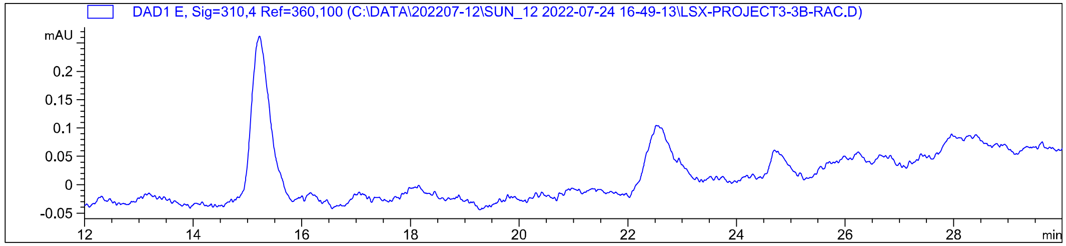

===
Area Percent Report
===

Sorted By   :   Signal
Multiplier   :   1.0000
Dilution   :   1.0000
Use Multiplier & Dilution Factor with ISTDs

Signal 1: DAD1 A, Sig=210,4 Ref=360,100

Signal 2: DAD1 B, Sig=230,4 Ref=360,100

Peak #	RetTime [min]	Type	Width [min]	Area [mAU*s]	Height [mAU]	Area %
1	15.330	BB	0.4085	1177.19836	44.22970	50.8037
2	24.613	BB	0.6081	1139.95337	28.17158	49.1963

Totals :   2317.15173   72.40128

Signal 3: DAD1 C, Sig=254,4 Ref=360,100

Signal 4: DAD1 D, Sig=280,4 Ref=360,100

Signal 5: DAD1 E, Sig=310,4 Ref=360,100

===
*** End of Report ***

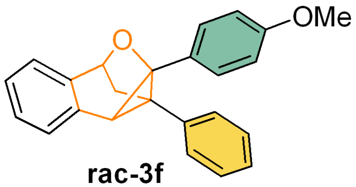

rac-3f

Data File C:\DATA\202201-06\SUN_12 2022-01-22 14-41-42\LSX-2-9B.D
Sample Name:

===

Acq. Operator : Seq. Line : 3
Acq. Instrument : Instrument 1 Location : 42
Injection Date : 21/1/2022 11:25:27 pm Inj : 1
Inj Volume : 5.000 µl
Acq. Method : C:\Chem32\1\DATA\SUN_12 2022-01-22 14-41-42\IC-02-30.M
Last changed :
(modified after loading)
Analysis Method : C:\Users\Public\Documents\ChemStation\1\Data\SUN\SUN 2024-03-13 00-13-49\AD-01-30-0.5.M (Sequence Method)
Last changed : 13/3/2024 6:54:01 pm by SYSTEM
(modified after loading)
Additional Info : Peak(s) manually integrated

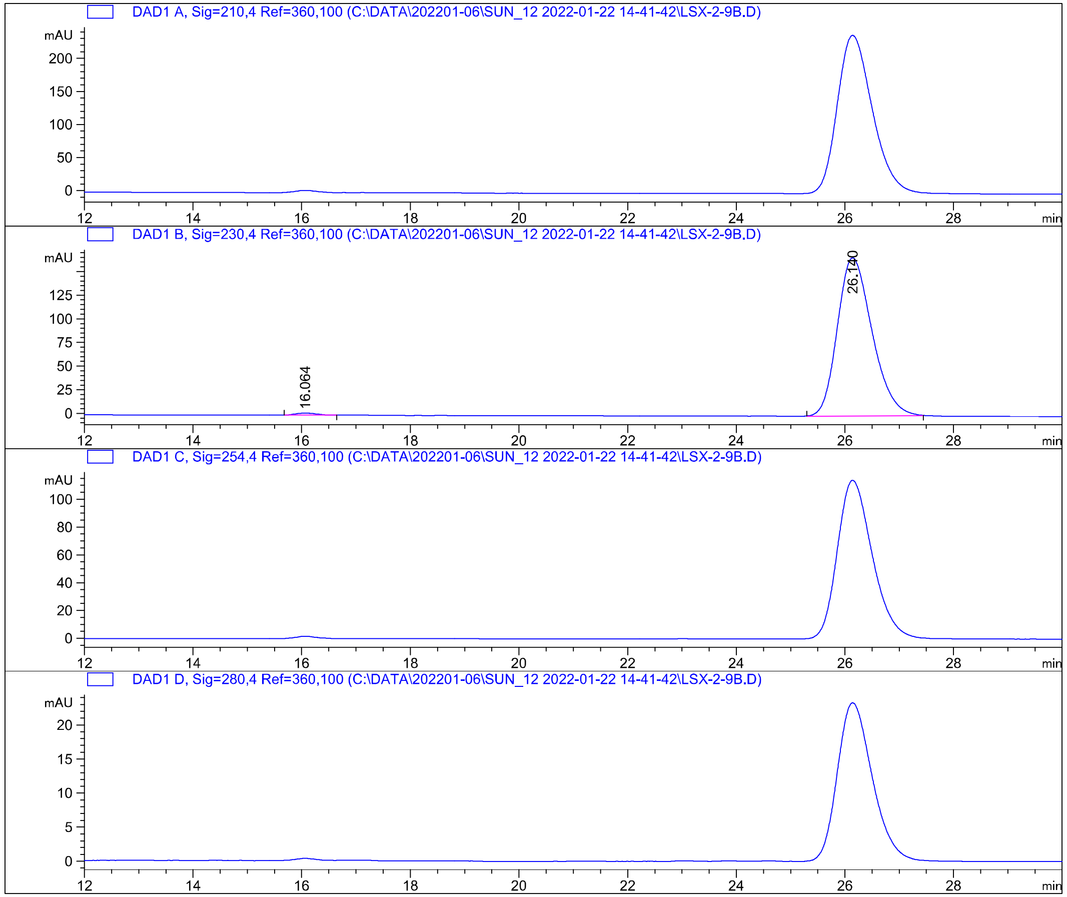

Data File C:\DATA\202201-06\SUN_12 2022-01-22 14-41-42\LSX-2-9B.D
Sample Name:

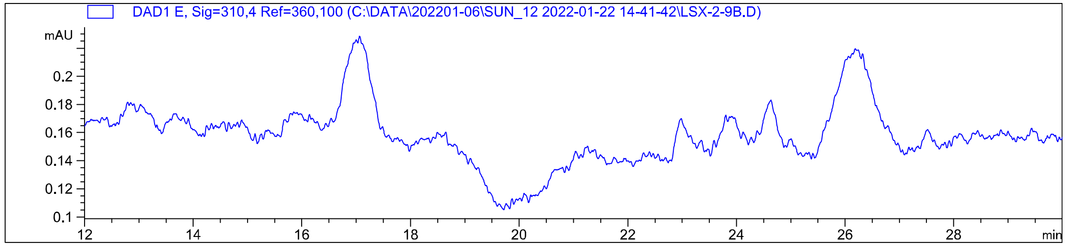

===

Area Percent Report

===

Sorted By : Signal
Multiplier : 1.0000
Dilution : 1.0000
Use Multiplier & Dilution Factor with ISTDs

Signal 1: DAD1 A, Sig=210,4 Ref=360,100

Signal 2: DAD1 B, Sig=230,4 Ref=360,100

Peak #	RetTime [min]	Type	Width [min]	Area [mAU*s]	Height [mAU]	Area %
1	16.064	BB	0.3842	54.60185	2.13649	0.7303
2	26.140	BB	0.6878	7422.14063	167.23262	99.2697

Totals : 7476.74247 169.36911

Signal 3: DAD1 C, Sig=254,4 Ref=360,100

Signal 4: DAD1 D, Sig=280,4 Ref=360,100

Signal 5: DAD1 E, Sig=310,4 Ref=360,100

===

*** End of Report ***

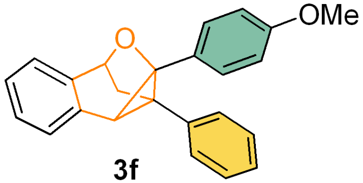

3f

Data File C:\DATA\202207-12\SUN_12 2022-08-01 21-33-13\LSX-P3-3C-RAC.D
Sample Name:

===

Acq. Operator : Seq. Line : 6
Acq. Instrument : Instrument 1 Location : 43
Injection Date : 1/8/2022 8:30:54 am Inj : 1
Inj Volume : 5.000 µl
Different Inj Volume from Sample Entry! Actual Inj Volume : 3.000 µl
Acq. Method : C:\Chem32\1\DATA\SUN_12 2022-08-01 21-33-13\IC-02-30.M
Last changed :
(modified after loading)
Analysis Method : C:\Users\Public\Documents\ChemStation\1\Data\SUN\SUN 2024-03-13 00-13-49\AD-01-30-0.5.M (Sequence Method)
Last changed : 13/3/2024 6:49:02 pm by SYSTEM
(modified after loading)
Additional Info : Peak(s) manually integrated

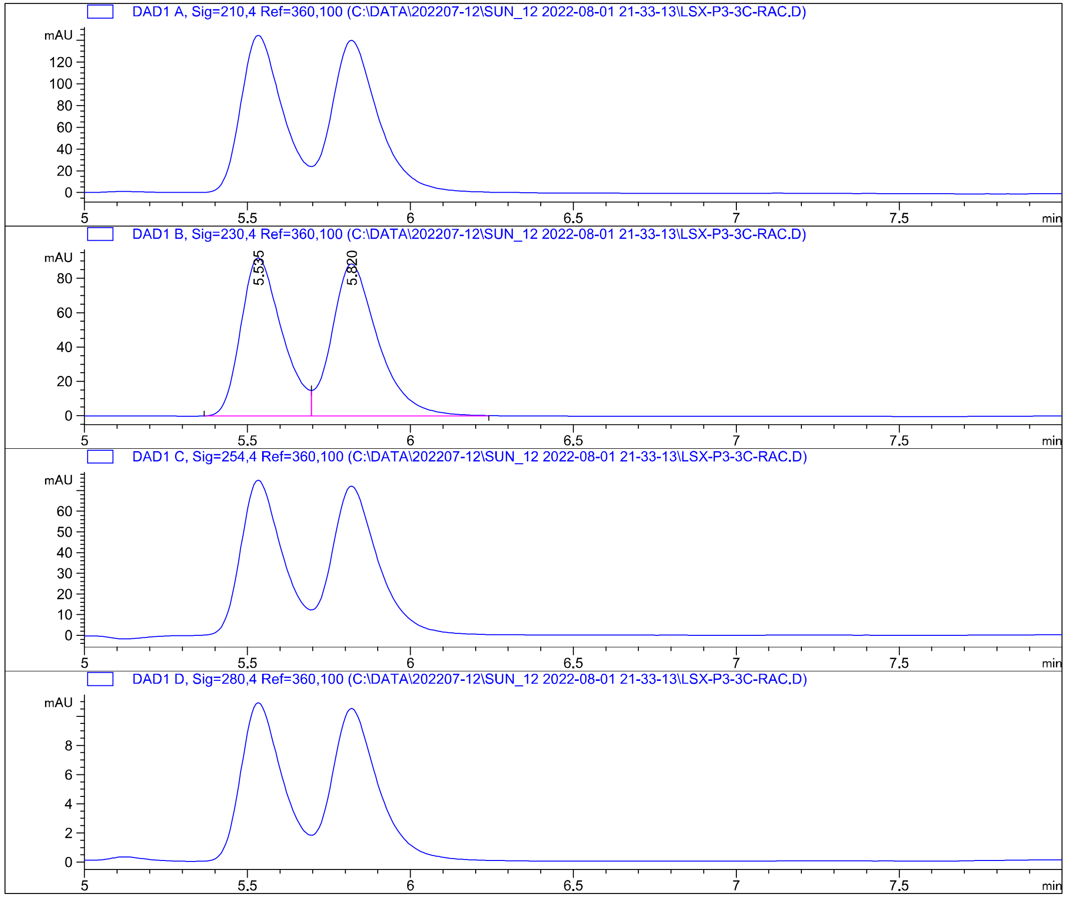

Data File C:\DATA\202207-12\SUN_12 2022-08-01 21-33-13\LSX-P3-3C-RAC.D
Sample Name:

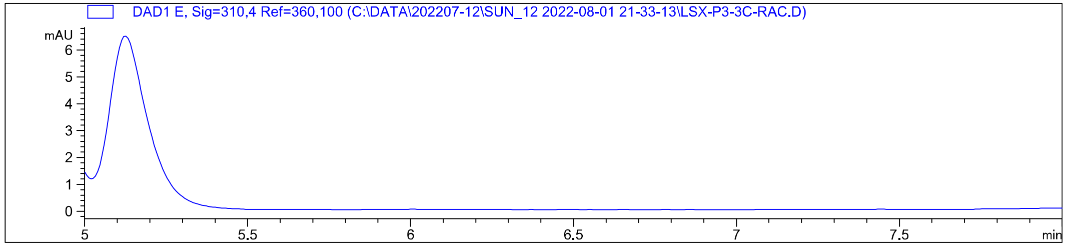

===

Area Percent Report

===

Sorted By : Signal
Multiplier : 1.0000
Dilution : 1.0000
Use Multiplier & Dilution Factor with ISTDs

Signal 1: DAD1 A, Sig=210,4 Ref=360,100

Signal 2: DAD1 B, Sig=230,4 Ref=360,100

Peak #	RetTime [min]	Type	Width [min]	Area [mAU*s]	Height [mAU]	Area %
1	5.535	BV	0.1323	808.85852	92.28972	47.9678
2	5.820	VB	0.1474	877.39423	88.69310	52.0322

Totals : 1686.25275 180.98282

Signal 3: DAD1 C, Sig=254,4 Ref=360,100

Signal 4: DAD1 D, Sig=280,4 Ref=360,100

Signal 5: DAD1 E, Sig=310,4 Ref=360,100

===

*** End of Report ***

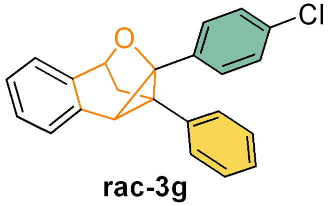

rac-3g

```
=====================================================================
Acq. Operator   :                                  Seq. Line :    2
Acq. Instrument : Instrument 1                      Location :   41
Injection Date  : 21/1/2022 10:54:29 pm                  Inj :    1
                                                  Inj Volume : 5.000 µl
Acq. Method     : C:\Chem32\1\DATA\SUN_12 2022-01-22 14-41-42\IC-02-30.M
Last changed    :
                  (modified after loading)
Analysis Method : C:\Users\Public\Documents\ChemStation\1\Data\SUN\SUN 2024-03-13 00-13-49\AD-01
                  -30-0.5.M (Sequence Method)
Last changed    : 13/3/2024 6:51:40 pm by SYSTEM
                  (modified after loading)
Additional Info : Peak(s) manually integrated
```

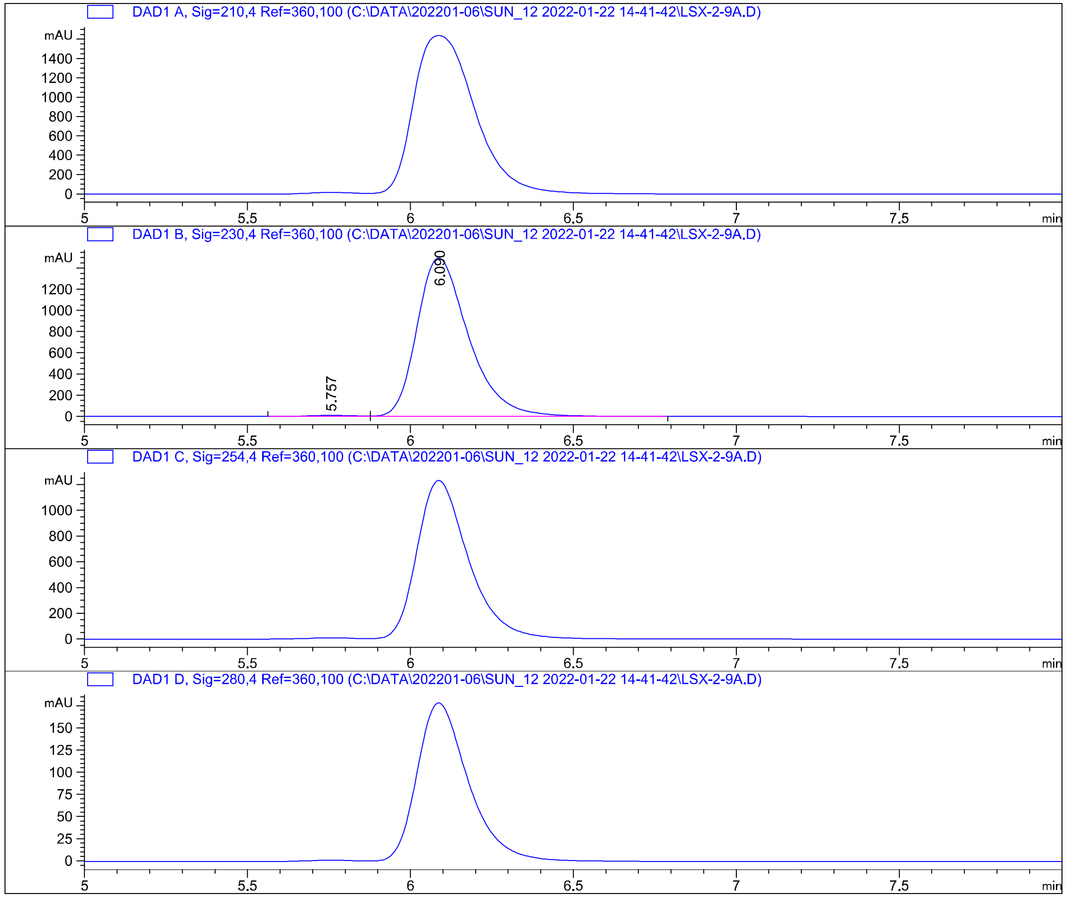

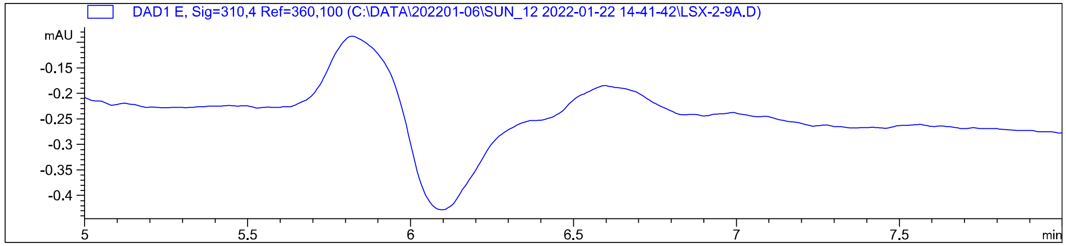


```
=====================================================================
                         Area Percent Report
=====================================================================

Sorted By             :      Signal
Multiplier            :      1.0000
Dilution              :      1.0000
Use Multiplier & Dilution Factor with ISTDs


Signal 1: DAD1 A, Sig=210,4 Ref=360,100


Signal 2: DAD1 B, Sig=230,4 Ref=360,100

Peak RetTime Type  Width     Area      Height     Area
  #   [min]        [min]   [mAU*s]     [mAU]        %
----|-------|----|-------|----------|----------|--------|
   1   5.757 BV    0.1488  108.80631   11.45479   0.6537
   2   6.090 VB    0.1666 1.65353e4  1500.02625  99.3463

Totals :                  1.66441e4  1511.48103


Signal 3: DAD1 C, Sig=254,4 Ref=360,100


Signal 4: DAD1 D, Sig=280,4 Ref=360,100


Signal 5: DAD1 E, Sig=310,4 Ref=360,100

Peak RetTime Type  Width     Area      Height     Area
  #   [min]        [min]   [mAU*s]     [mAU]        %
----|-------|----|-------|----------|----------|--------|
   1   4.167 BB    0.1859   18.95916    1.51598 100.0000

Totals :                    18.95916    1.51598

=====================================================================
                         *** End of Report ***
```

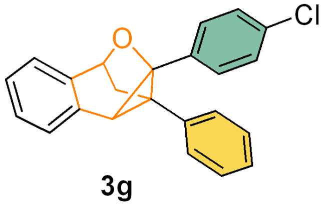

3g

Data File C:\DATA\202207-12\SUN_12 2022-08-02 10-25-51\LSX-P3-3D-RAC.D
Sample Name:

===

```
Acq. Operator   :                        Seq. Line :    3
Acq. Instrument : Instrument 1            Location :   41
Injection Date  : 1/8/2022 8:09:45 pm          Inj :    1
                                        Inj Volume : 5.000 µl
Different Inj Volume from Sample Entry!  Actual Inj Volume : 10.000 µl
Acq. Method     : C:\Chem32\1\DATA\SUN_12 2022-08-02 10-25-51\IC-05-60.M
Last changed    :
                  (modified after loading)
Analysis Method : C:\Users\Public\Documents\ChemStation\1\Data\SUN\SUN 2024-03-13 00-13-49\AD-01
                  -30-0.5.M (Sequence Method)
Last changed    : 13/3/2024 6:59:52 pm by SYSTEM
                  (modified after loading)
Additional Info : Peak(s) manually integrated
```

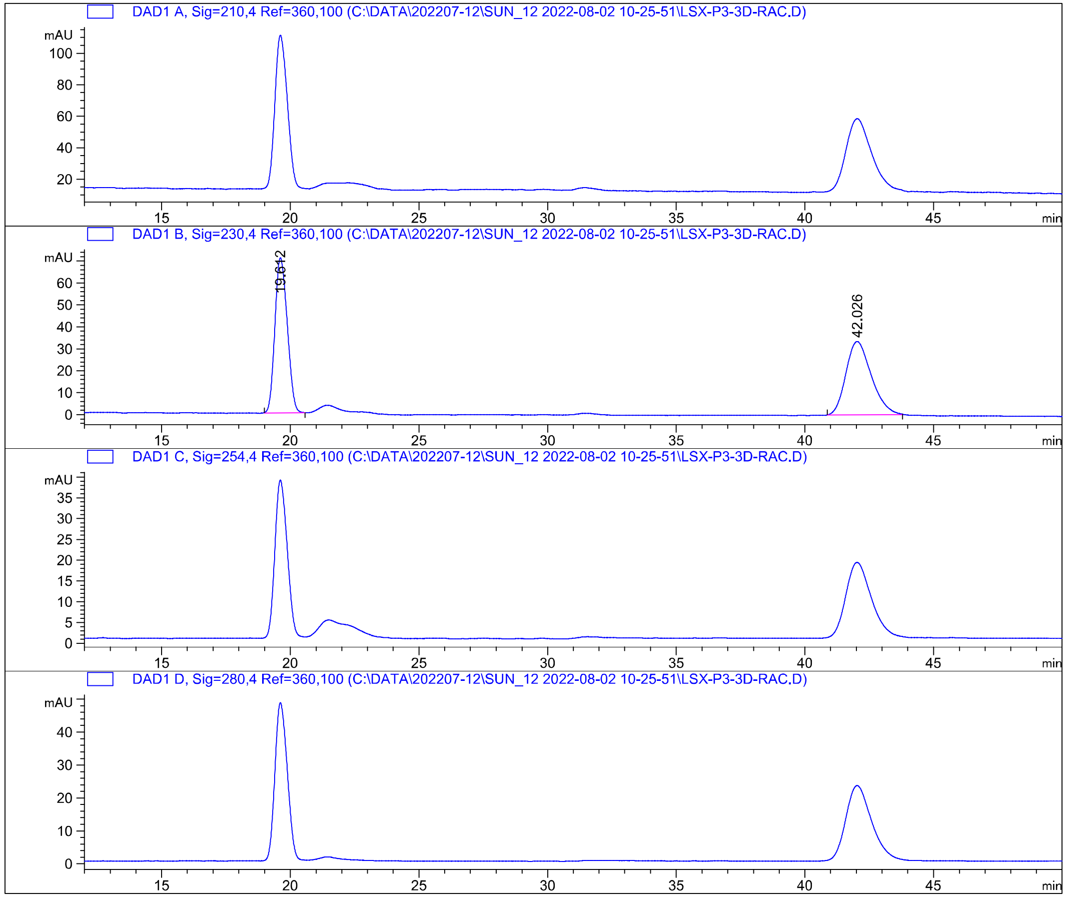

Data File C:\DATA\202207-12\SUN_12 2022-08-02 10-25-51\LSX-P3-3D-RAC.D
Sample Name:

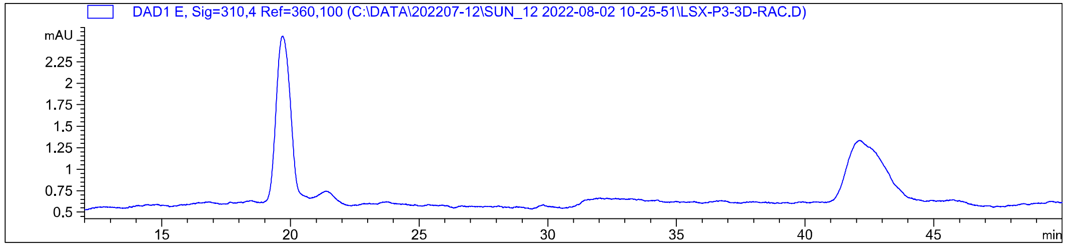

===

Area Percent Report

===

```
Sorted By              :      Signal
Multiplier             :      1.0000
Dilution               :      1.0000
Use Multiplier & Dilution Factor with ISTDs
```

Signal 1: DAD1 A, Sig=210,4 Ref=360,100

Signal 2: DAD1 B, Sig=230,4 Ref=360,100

Peak #	RetTime [min]	Type	Width [min]	Area [mAU*s]	Height [mAU]	Area %
1	19.612	BB	0.5332	2361.09155	70.76770	50.6720
2	42.026	BB	1.0080	2298.46875	33.43648	49.3280

Totals : 4659.56030 104.20418

Signal 3: DAD1 C, Sig=254,4 Ref=360,100

Signal 4: DAD1 D, Sig=280,4 Ref=360,100

Signal 5: DAD1 E, Sig=310,4 Ref=360,100

===

*** End of Report ***

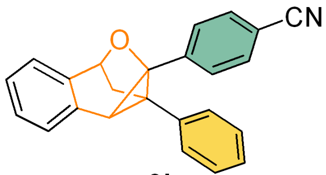

rac-3h

Data File C:\DATA\202201-06\SUN_12 2022-03-26 14-17-08\LSX-2-80.D
Sample Name:

===

Acq. Operator   :   Seq. Line : 17
Acq. Instrument : Instrument 1   Location :   43
Injection Date : 26/3/2022 3:46:18 am   Inj :   1
                                                 Inj Volume : 5.000 µl
Different Inj Volume from Sample Entry! Actual Inj Volume : 8.000 µl
Acq. Method   : C:\Chem32\1\DATA\SUN_12 2022-03-26 14-17-08\IC-05-60.M
Last changed   :
                  (modified after loading)
Analysis Method : C:\Users\Public\Documents\ChemStation\1\Data\SUN\SUN 2024-03-13 17-26-47\OD-00
                  -20.M (Sequence Method)
Last changed   : 13/3/2024 10:13:55 pm by SYSTEM
                  (modified after loading)
Additional Info : Peak(s) manually integrated

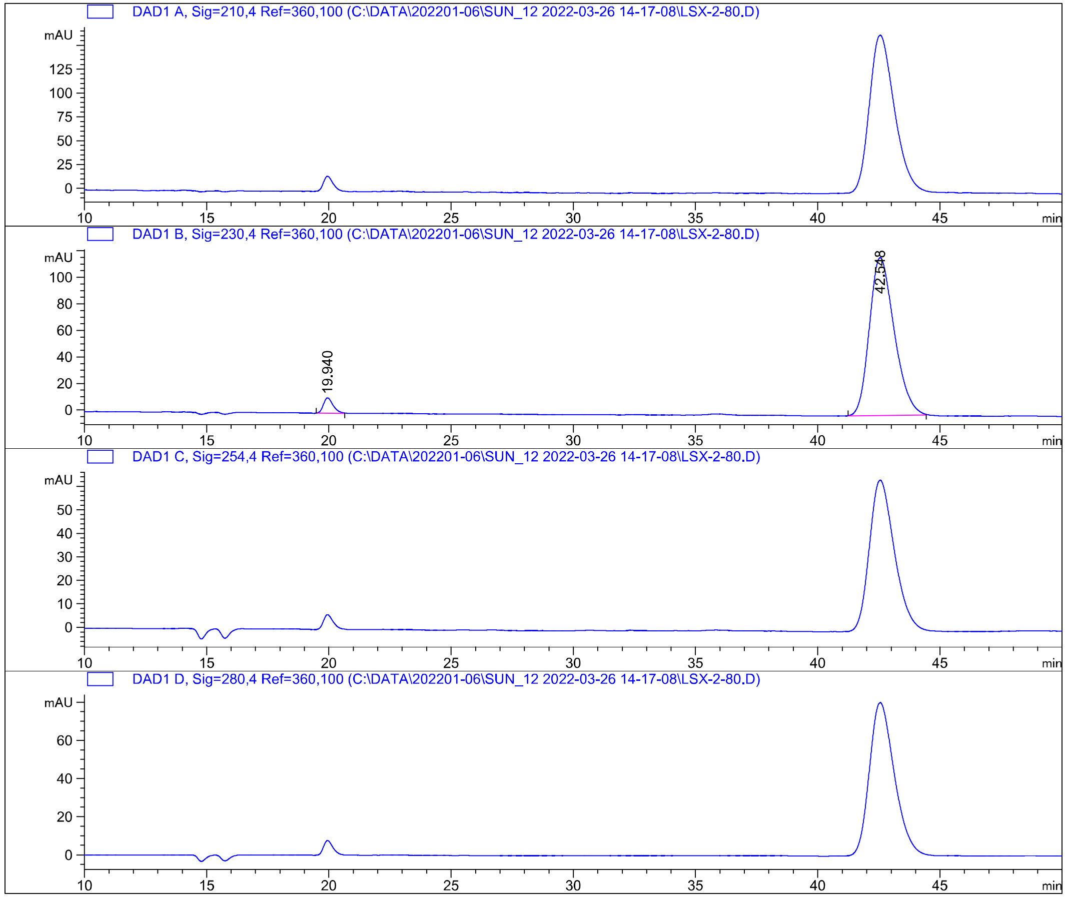

Data File C:\DATA\202201-06\SUN_12 2022-03-26 14-17-08\LSX-2-80.D
Sample Name:

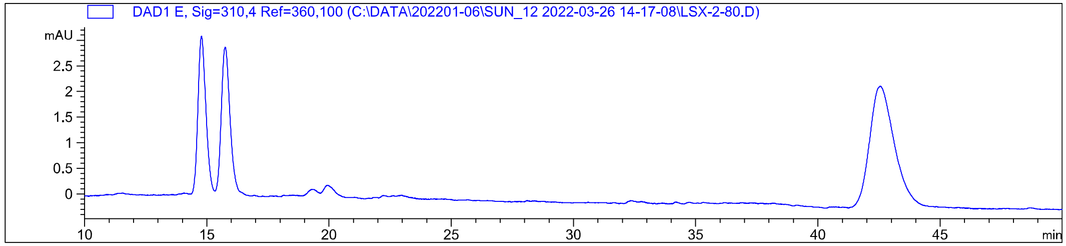

===

Area Percent Report

===

Sorted By : Signal
Multiplier : 1.0000
Dilution : 1.0000
Use Multiplier & Dilution Factor with ISTDs

Signal 1: DAD1 A, Sig=210,4 Ref=360,100

Signal 2: DAD1 B, Sig=230,4 Ref=360,100

Peak #	RetTime [min]	Type	Width [min]	Area [mAU*s]	Height [mAU]	Area %
1	19.940	BB	0.4227	313.61639	11.48019	3.5907
2	42.548	BB	1.0803	8420.54102	118.88423	96.4093

Totals : 8734.15741 130.36442

Signal 3: DAD1 C, Sig=254,4 Ref=360,100

Signal 4: DAD1 D, Sig=280,4 Ref=360,100

Signal 5: DAD1 E, Sig=310,4 Ref=360,100

===

*** End of Report ***

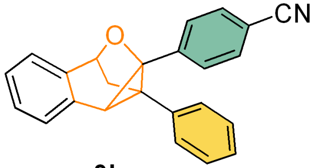

3h

Data File C:\DATA\202207-12\SUN_12 2022-07-24 16-49-13\LSX-PROJECT3-3F-RAC.D
Sample Name:

===

```
Acq. Operator   :                              Seq. Line :    4
Acq. Instrument : Instrument 1                  Location :   43
Injection Date  : 24/7/2022 3:05:01 am               Inj :    1
                                              Inj Volume : 5.000 µl
Acq. Method     : C:\Chem32\1\DATA\SUN_12 2022-07-24 16-49-13\IC-02-30.M
Last changed    :
                  (modified after loading)
Analysis Method : C:\Users\Public\Documents\ChemStation\1\Data\SUN\SUN 2024-03-13 17-26-47\OD-00
                  -20.M (Sequence Method)
Last changed    : 13/3/2024 10:20:12 pm by SYSTEM
                  (modified after loading)
Additional Info : Peak(s) manually integrated
```

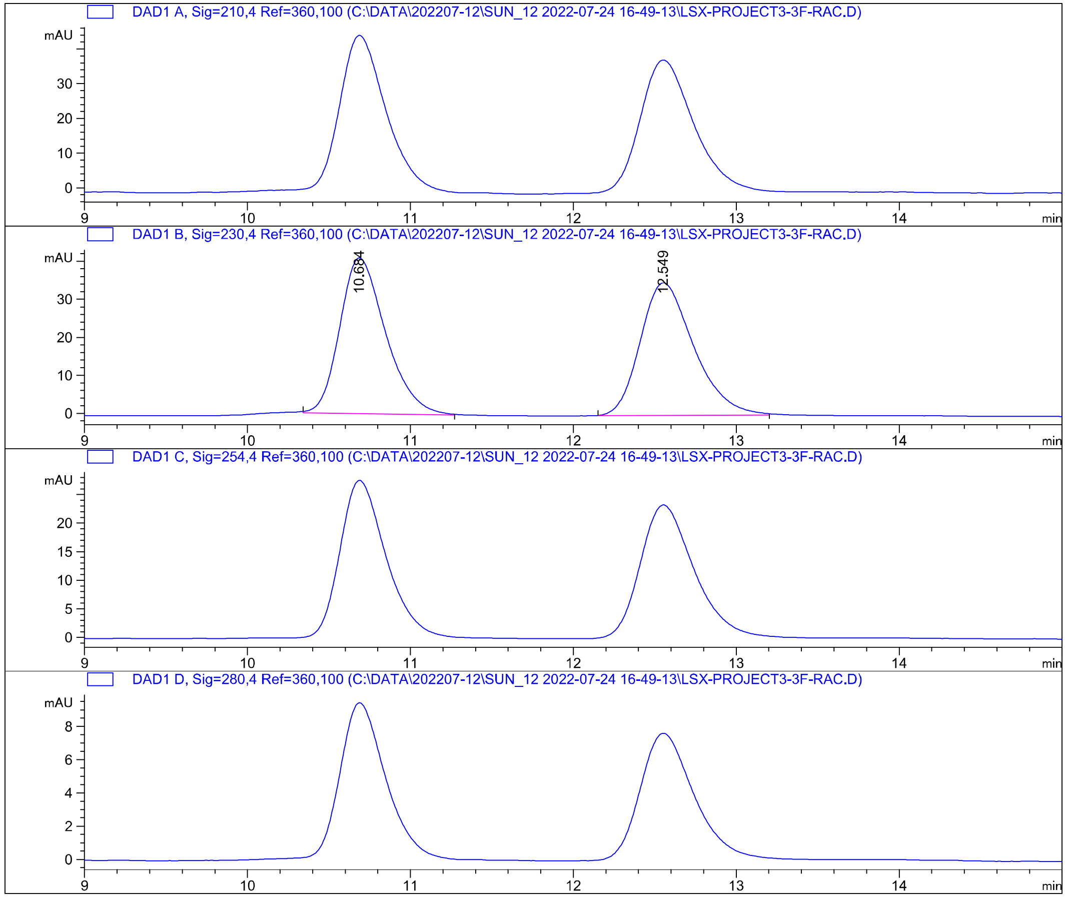

Data File C:\DATA\202207-12\SUN_12 2022-07-24 16-49-13\LSX-PROJECT3-3F-RAC.D
Sample Name:

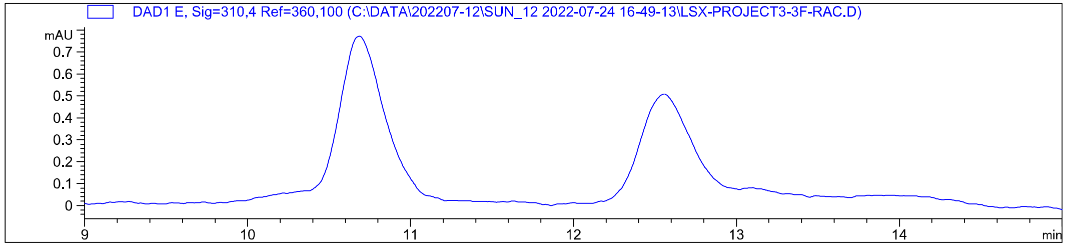

===
Area Percent Report
===

Sorted By : Signal
Multiplier : 1.0000
Dilution : 1.0000
Use Multiplier & Dilution Factor with ISTDs

Signal 1: DAD1 A, Sig=210,4 Ref=360,100

Signal 2: DAD1 B, Sig=230,4 Ref=360,100

Peak #	RetTime [min]	Type	Width [min]	Area [mAU*s]	Height [mAU]	Area %
1	10.684	BB	0.2979	800.22064	40.97282	50.1543
2	12.549	BB	0.3459	795.29755	34.89804	49.8457

Totals : 1595.51819 75.87086

Signal 3: DAD1 C, Sig=254,4 Ref=360,100

Signal 4: DAD1 D, Sig=280,4 Ref=360,100

Signal 5: DAD1 E, Sig=310,4 Ref=360,100

===
*** End of Report ***

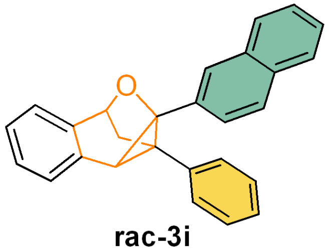

rac-3i

Data File C:\DATA\202201-06\SUN_12 2022-03-26 14-17-08\LSX-2-79.D
Sample Name:

===

Acq. Operator : Seq. Line : 11
Acq. Instrument : Instrument 1 Location : 42
Injection Date : 26/3/2022 2:09:47 am Inj : 1
Inj Volume : 5.000 µl
Different Inj Volume from Sample Entry! Actual Inj Volume : 8.000 µl
Acq. Method : C:\Chem32\1\DATA\SUN_12 2022-03-26 14-17-08\IC-02-30.M
Last changed :
(modified after loading)
Analysis Method : C:\Users\Public\Documents\ChemStation\1\Data\SUN\SUN 2024-03-13 17-26-47\OD-00-20.M (Sequence Method)
Last changed : 13/3/2024 10:20:12 pm by SYSTEM
(modified after loading)
Additional Info : Peak(s) manually integrated

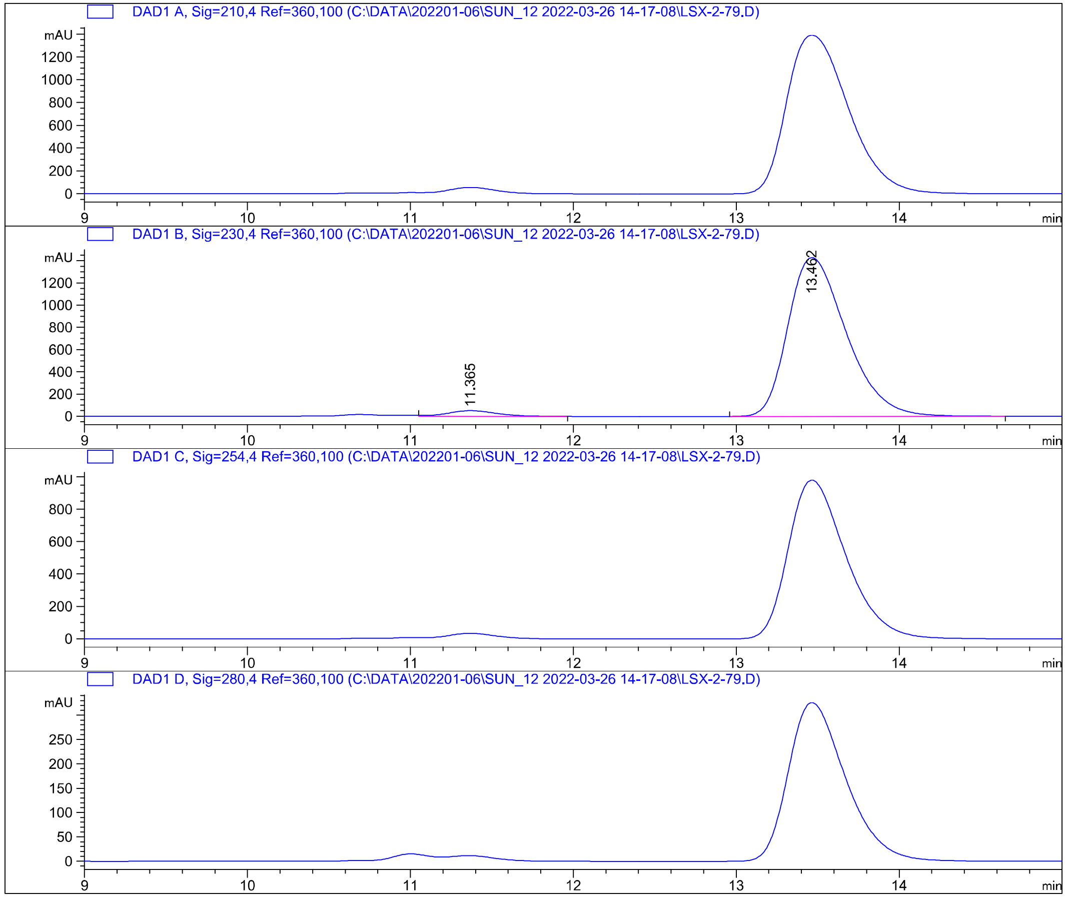

Data File C:\DATA\202201-06\SUN_12 2022-03-26 14-17-08\LSX-2-79.D
Sample Name:

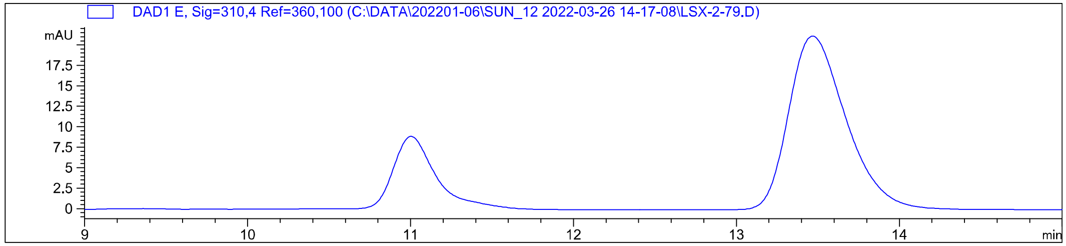

===

Area Percent Report

===

Sorted By : Signal
Multiplier : 1.0000
Dilution : 1.0000
Use Multiplier & Dilution Factor with ISTDs

Signal 1: DAD1 A, Sig=210,4 Ref=360,100

Signal 2: DAD1 B, Sig=230,4 Ref=360,100

Peak #	RetTime [min]	Type	Width [min]	Area [mAU*s]	Height [mAU]	Area %
1	11.365	VB	0.3222	1072.72888	50.84315	2.9248
2	13.462	BB	0.3867	3.56041e4	1428.71472	97.0752

Totals : 3.66769e4 1479.55787

Signal 3: DAD1 C, Sig=254,4 Ref=360,100

Signal 4: DAD1 D, Sig=280,4 Ref=360,100

Signal 5: DAD1 E, Sig=310,4 Ref=360,100

===

*** End of Report ***

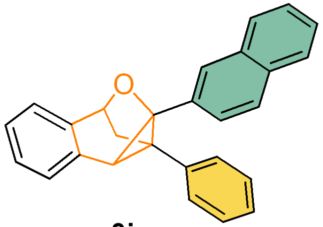

3i

Data File C:\DATA\202207-12\SUN_12 2022-07-24 16-49-13\LSX-PROJECT3-3G-RAC.D
Sample Name:

===
Acq. Operator   :   Seq. Line :   5
Acq. Instrument : Instrument 1   Location :   44
Injection Date : 24/7/2022 3:35:58 am   Inj :   1
                                               Inj Volume : 5.000 µl
Acq. Method   : C:\Chem32\1\DATA\SUN_12 2022-07-24 16-49-13\IC-02-30.M
Last changed   :
                  (modified after loading)
Analysis Method : C:\Users\Public\Documents\ChemStation\1\Data\SUN\SUN 2024-03-13 17-26-47\OD-00
                  -20.M (Sequence Method)
Last changed   : 13/3/2024 10:25:44 pm by SYSTEM
                  (modified after loading)
Additional Info : Peak(s) manually integrated

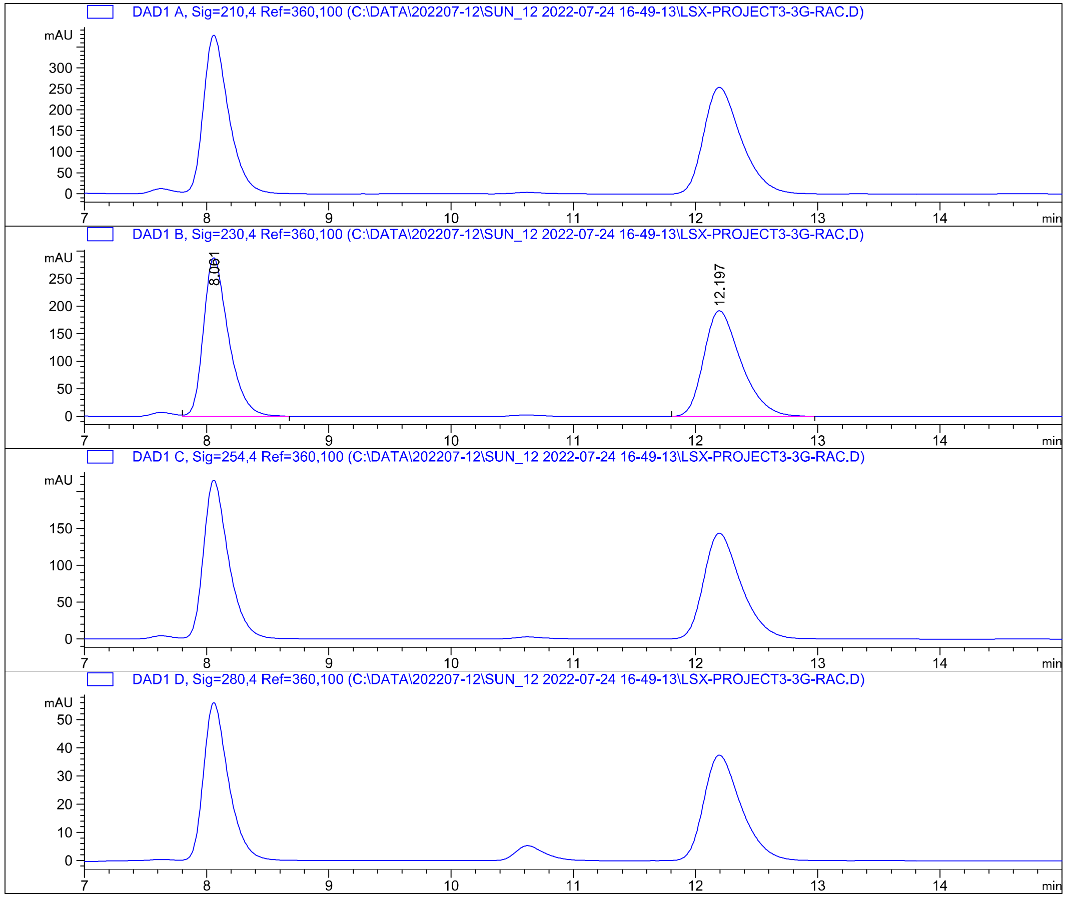

Data File C:\DATA\202207-12\SUN_12 2022-07-24 16-49-13\LSX-PROJECT3-3G-RAC.D
Sample Name:

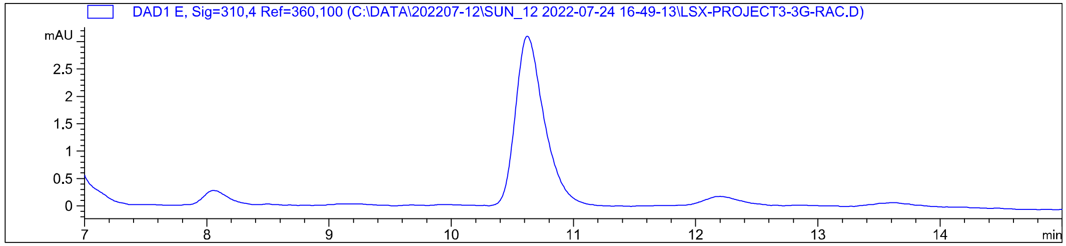

===
Area Percent Report
===

Sorted By   :   Signal
Multiplier   :   1.0000
Dilution   :   1.0000
Use Multiplier & Dilution Factor with ISTDs

Signal 1: DAD1 A, Sig=210,4 Ref=360,100

Signal 2: DAD1 B, Sig=230,4 Ref=360,100

Peak #	RetTime [min]	Type	Width [min]	Area [mAU*s]	Height [mAU]	Area %
1	8.061	VB	0.2140	4101.22998	288.21036	50.1711
2	12.197	BB	0.3213	4073.25879	192.12576	49.8289

Totals : 8174.48877 480.33612

Signal 3: DAD1 C, Sig=254,4 Ref=360,100

Signal 4: DAD1 D, Sig=280,4 Ref=360,100

Signal 5: DAD1 E, Sig=310,4 Ref=360,100

===
*** End of Report ***

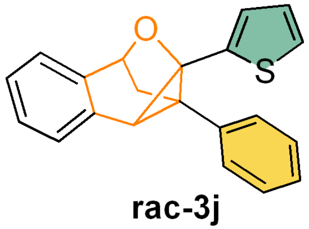

rac-3j

Data File C:\DATA\202201-06\SUN_12 2022-03-26 10-50-48\LSX-2-78.D
Sample Name:

===

```
Acq. Operator   :                               Seq. Line :    2
Acq. Instrument : Instrument 1                   Location :   41
Injection Date  : 25/3/2022 8:03:36 pm                Inj :    1
                                               Inj Volume : 5.000 µl
Different Inj Volume from Sample Entry!  Actual Inj Volume : 8.000 µl
Acq. Method     : C:\Chem32\1\DATA\SUN_12 2022-03-26 10-50-48\IC-02-30.M
Last changed    :
                  (modified after loading)
Analysis Method : C:\Users\Public\Documents\ChemStation\1\Data\SUN\SUN 2024-03-13 17-26-47\OD-00
                  -20.M (Sequence Method)
Last changed    : 13/3/2024 10:25:44 pm by SYSTEM
                  (modified after loading)
Additional Info : Peak(s) manually integrated
```

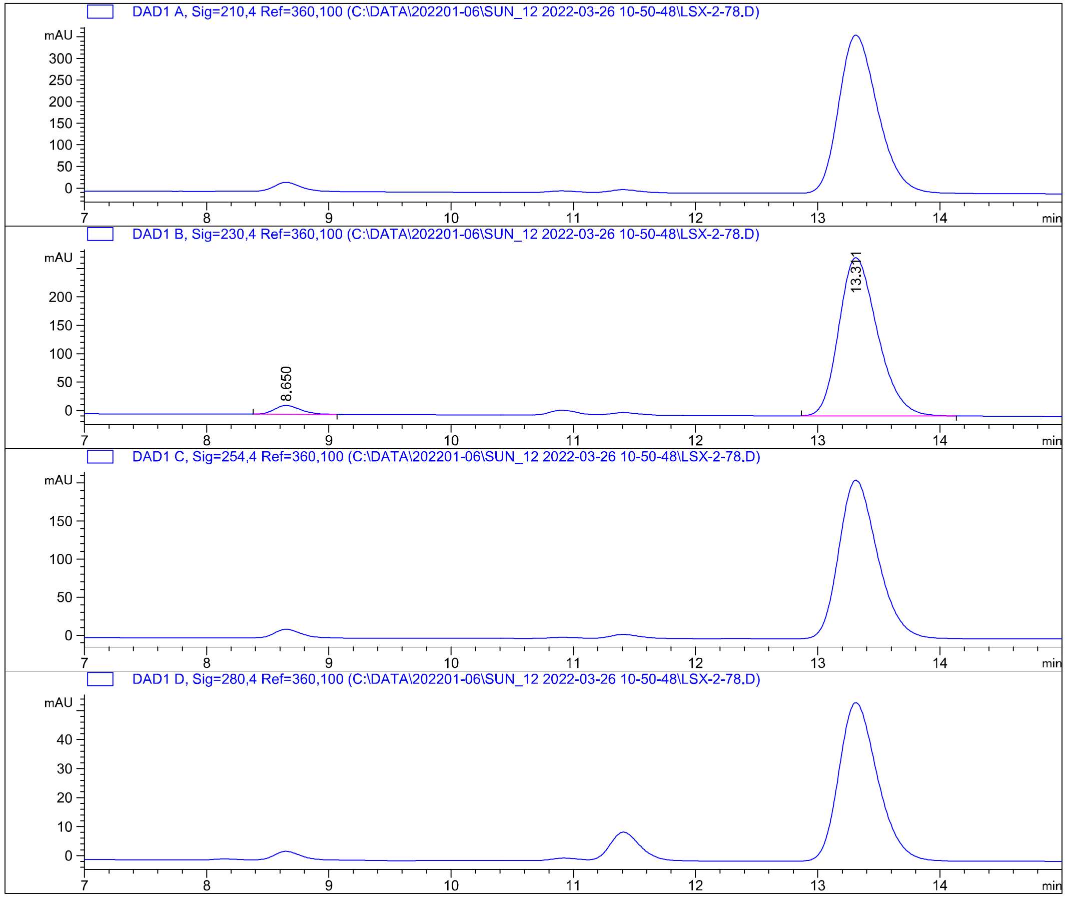

Data File C:\DATA\202201-06\SUN_12 2022-03-26 10-50-48\LSX-2-78.D
Sample Name:

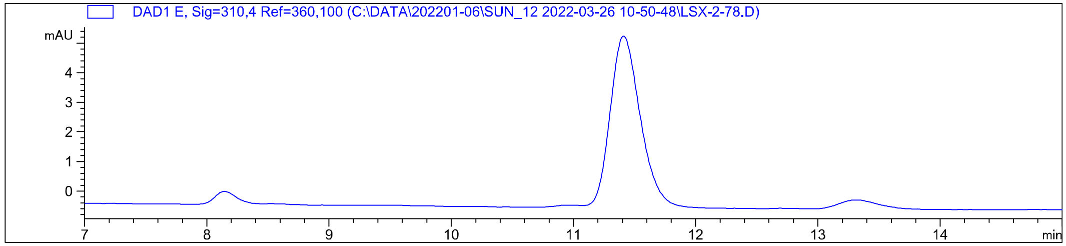

===

Area Percent Report

===

```
Sorted By             :      Signal
Multiplier            :      1.0000
Dilution              :      1.0000
Use Multiplier & Dilution Factor with ISTDs
```

Signal 1: DAD1 A, Sig=210,4 Ref=360,100

Signal 2: DAD1 B, Sig=230,4 Ref=360,100

Peak #	RetTime [min]	Type	Width [min]	Area [mAU*s]	Height [mAU]	Area %
1	8.650	BB	0.2285	231.77061	15.49835	3.6366
2	13.311	BB	0.3387	6141.45313	279.10840	96.3634

Totals : 6373.22374 294.60674

Signal 3: DAD1 C, Sig=254,4 Ref=360,100

Signal 4: DAD1 D, Sig=280,4 Ref=360,100

Signal 5: DAD1 E, Sig=310,4 Ref=360,100

===

*** End of Report ***

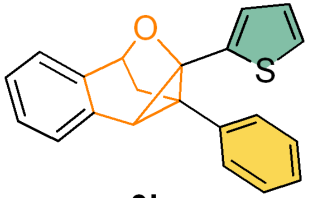

3j

Data File C:\DATA\202207-12\SUN_12 2022-08-05 08-58-56\LSX-P3-3E-RAC.D
Sample Name:

===

Acq. Operator : Seq. Line : 8
Acq. Instrument : Instrument 1 Location : 41
Injection Date : 4/8/2022 9:02:18 pm Inj : 1
Inj Volume : 5.000 µl
Different Inj Volume from Sample Entry! Actual Inj Volume : 8.000 µl
Acq. Method : C:\Chem32\1\DATA\SUN_12 2022-08-05 08-58-56\IC-02-30.M
Last changed :
(modified after loading)
Analysis Method : C:\Users\Public\Documents\ChemStation\1\Data\SUN\SUN 2024-03-13 17-26-47\OD-00-20.M (Sequence Method)
Last changed : 13/3/2024 10:16:44 pm by SYSTEM
(modified after loading)
Additional Info : Peak(s) manually integrated

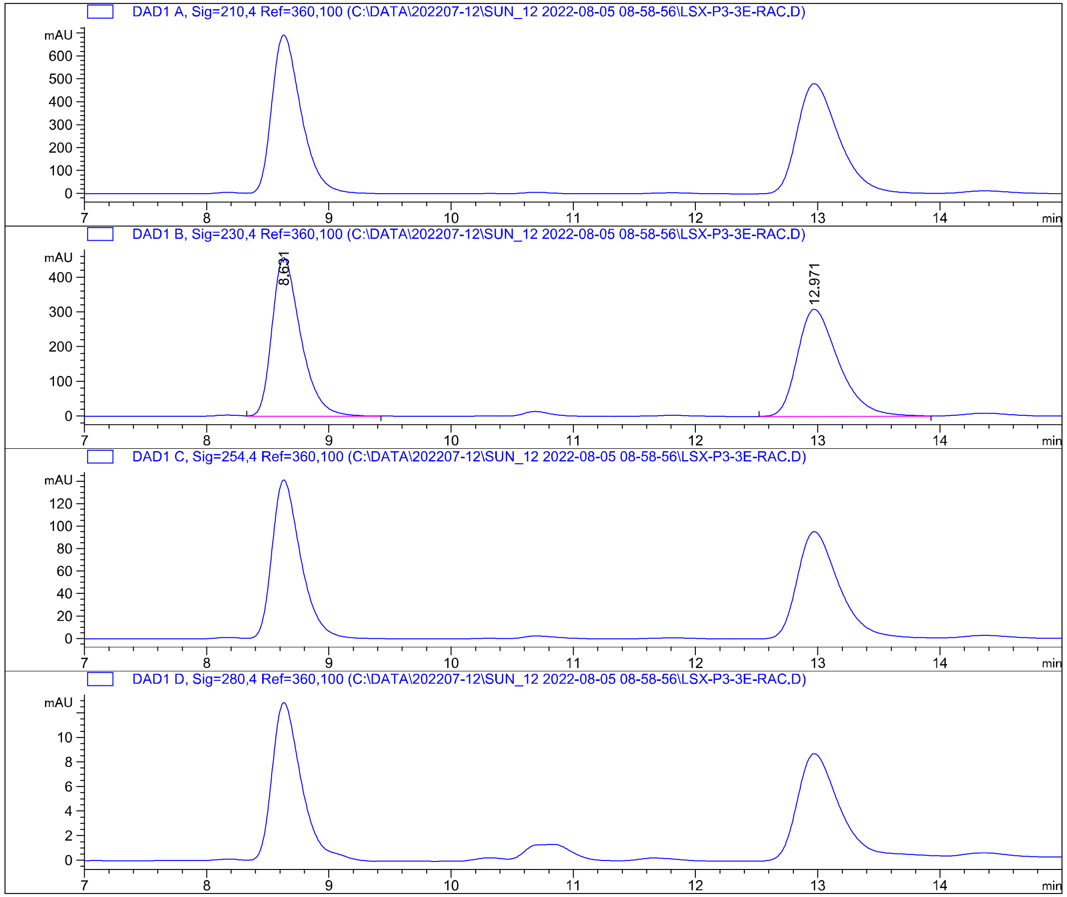

Data File C:\DATA\202207-12\SUN_12 2022-08-05 08-58-56\LSX-P3-3E-RAC.D
Sample Name:

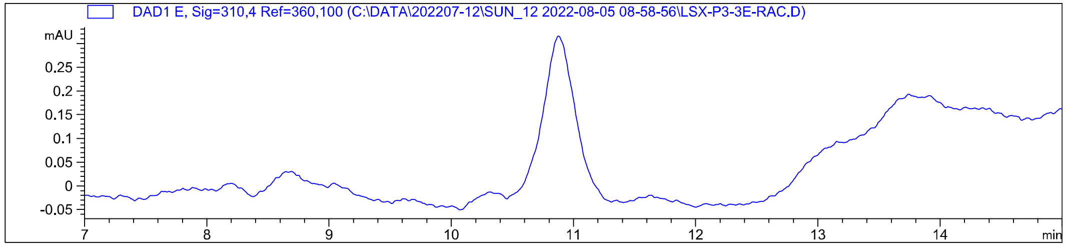

===

Area Percent Report

===

Sorted By : Signal
Multiplier : 1.0000
Dilution : 1.0000
Use Multiplier & Dilution Factor with ISTDs

Signal 1: DAD1 A, Sig=210,4 Ref=360,100

Signal 2: DAD1 B, Sig=230,4 Ref=360,100

Peak #	RetTime [min]	Type	Width [min]	Area [mAU*s]	Height [mAU]	Area %
1	8.631	VB	0.2464	7458.77051	457.08408	50.7257
2	12.971	BB	0.3602	7245.36279	308.31094	49.2743

Totals : 1.47041e4 765.39502

Signal 3: DAD1 C, Sig=254,4 Ref=360,100

Signal 4: DAD1 D, Sig=280,4 Ref=360,100

Signal 5: DAD1 E, Sig=310,4 Ref=360,100

===

*** End of Report ***

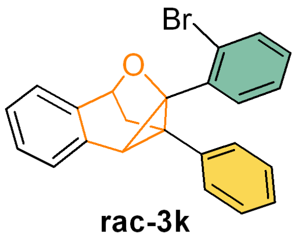

rac-3k

Data File C:\DATA\202201-06\SUN_12 2022-05-06 19-51-00\LSX-2-141-FR.D
Sample Name:

===

Acq. Operator : Seq. Line : 2
Acq. Instrument : Instrument 1 Location : 41
Injection Date : 6/5/2022 5:04:00 am Inj : 1
Inj Volume : 5.000 µl
Different Inj Volume from Sample Entry! Actual Inj Volume : 10.000 µl
Acq. Method : C:\Chem32\1\DATA\SUN_12 2022-05-06 19-51-00\IC-02-30.M
Last changed :
(modified after loading)
Analysis Method : C:\Users\Public\Documents\ChemStation\1\Data\SUN\SUN 2024-03-13 17-26-47\OD-00-20.M (Sequence Method)
Last changed : 13/3/2024 10:18:07 pm by SYSTEM
(modified after loading)
Additional Info : Peak(s) manually integrated

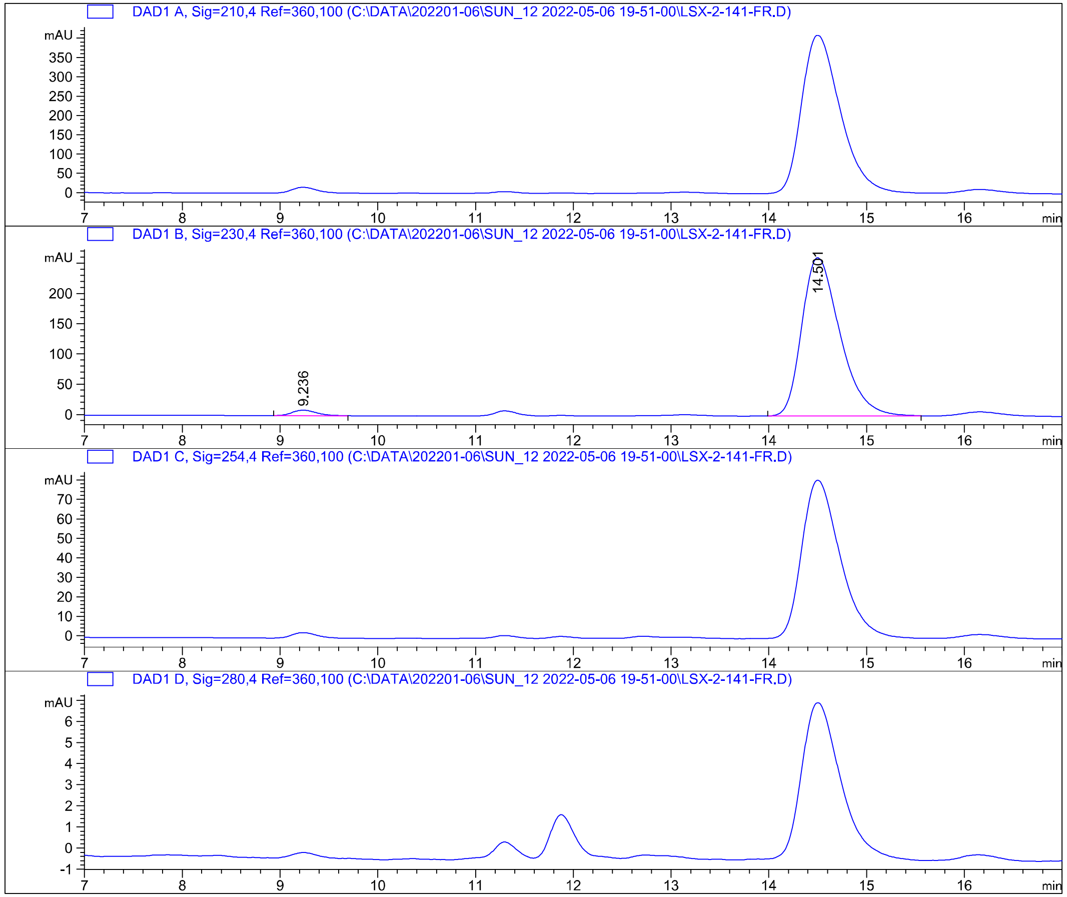

Data File C:\DATA\202201-06\SUN_12 2022-05-06 19-51-00\LSX-2-141-FR.D
Sample Name:

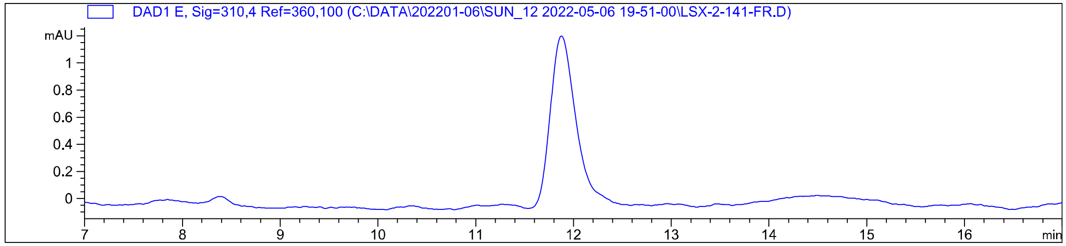

===

Area Percent Report

===

Sorted By : Signal
Multiplier : 1.0000
Dilution : 1.0000
Use Multiplier & Dilution Factor with ISTDs

Signal 1: DAD1 A, Sig=210,4 Ref=360,100

Signal 2: DAD1 B, Sig=230,4 Ref=360,100

Peak #	RetTime [min]	Type	Width [min]	Area [mAU*s]	Height [mAU]	Area %
1	9.236	BB	0.2621	157.32568	9.08126	2.1678
2	14.501	BB	0.4166	7100.08203	261.63687	97.8322
Totals :				7257.40771	270.71813	

Signal 3: DAD1 C, Sig=254,4 Ref=360,100

Signal 4: DAD1 D, Sig=280,4 Ref=360,100

Signal 5: DAD1 E, Sig=310,4 Ref=360,100

===

*** End of Report ***

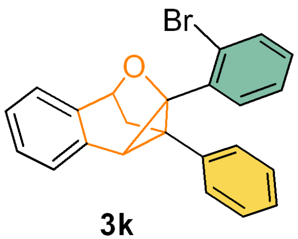

3k

Data File C:\DATA\202207-12\SUN_12 2022-08-03 10-53-42\LSX-P3-3O-RAC.D
Sample Name:

===

Acq. Operator : Seq. Line : 2
Acq. Instrument : Instrument 1 Location : 41
Injection Date : 2/8/2022 8:06:45 pm Inj : 1
Inj Volume : 5.000 µl
Different Inj Volume from Sample Entry! Actual Inj Volume : 8.000 µl
Acq. Method : C:\Chem32\1\DATA\SUN_12 2022-08-03 10-53-42\IC-02-30.M
Last changed :
(modified after loading)
Analysis Method : C:\Users\Public\Documents\ChemStation\1\Data\SUN\SUN 2024-03-13 17-26-47\OD-00-20.M (Sequence Method)
Last changed : 13/3/2024 10:50:10 pm by SYSTEM
(modified after loading)
Additional Info : Peak(s) manually integrated

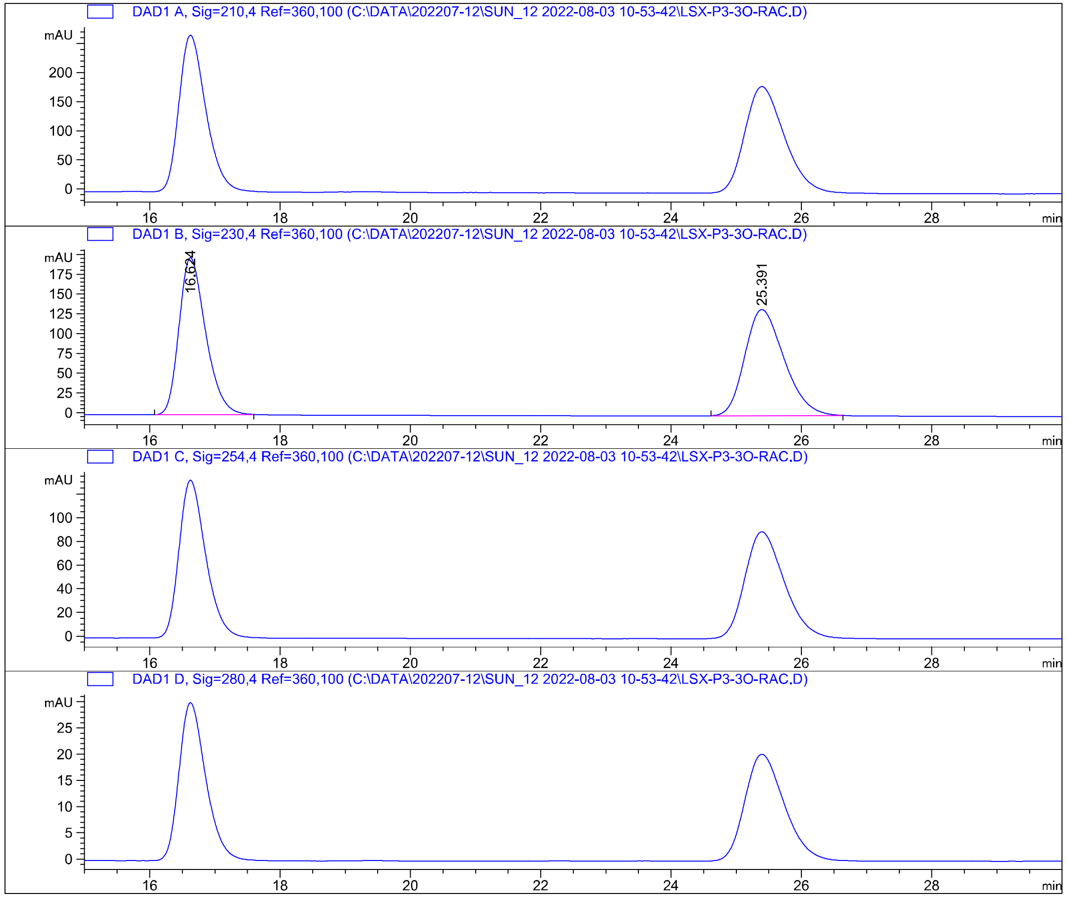

Data File C:\DATA\202207-12\SUN_12 2022-08-03 10-53-42\LSX-P3-3O-RAC.D
Sample Name:

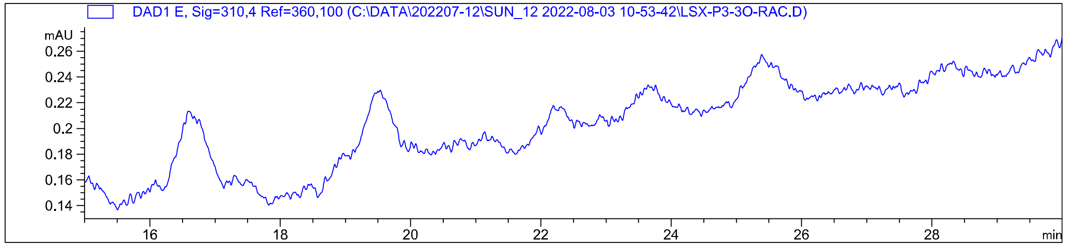

===
Area Percent Report
===

Sorted By : Signal
Multiplier : 1.0000
Dilution : 1.0000
Use Multiplier & Dilution Factor with ISTDs

Signal 1: DAD1 A, Sig=210,4 Ref=360,100

Signal 2: DAD1 B, Sig=230,4 Ref=360,100

Peak #	RetTime [min]	Type	Width [min]	Area [mAU*s]	Height [mAU]	Area %
1	16.624	BB	0.4381	5646.89551	198.38628	50.0481
2	25.391	BB	0.6482	5636.03174	134.14885	49.9519

Totals : 1.12829e4 332.53513

Signal 3: DAD1 C, Sig=254,4 Ref=360,100

Signal 4: DAD1 D, Sig=280,4 Ref=360,100

Signal 5: DAD1 E, Sig=310,4 Ref=360,100

===
*** End of Report ***

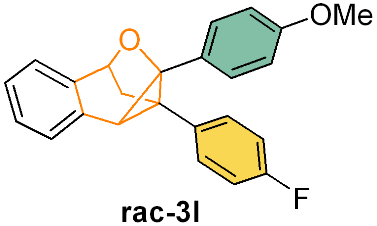

rac-3l

```
=====================================================================
Acq. Operator   :                                Seq. Line :  20
Acq. Instrument : Instrument 1                    Location :   44
Injection Date  : 14/2/2022 1:22:01 pm                 Inj :   1
                                                Inj Volume : 5.000 µl
Different Inj Volume from Sample Entry!  Actual Inj Volume : 10.000 µl
Acq. Method     : C:\Chem32\1\DATA\SUN_12 2022-02-14 21-09-52\IC-02-30.M
Last changed    :
                  (modified after loading)
Analysis Method : C:\Users\Public\Documents\ChemStation\1\Data\SUN\SUN 2024-03-13 17-26-47\OD-00
                  -20.M (Sequence Method)
Last changed    : 13/3/2024 10:50:10 pm by SYSTEM
                  (modified after loading)
Additional Info : Peak(s) manually integrated
```

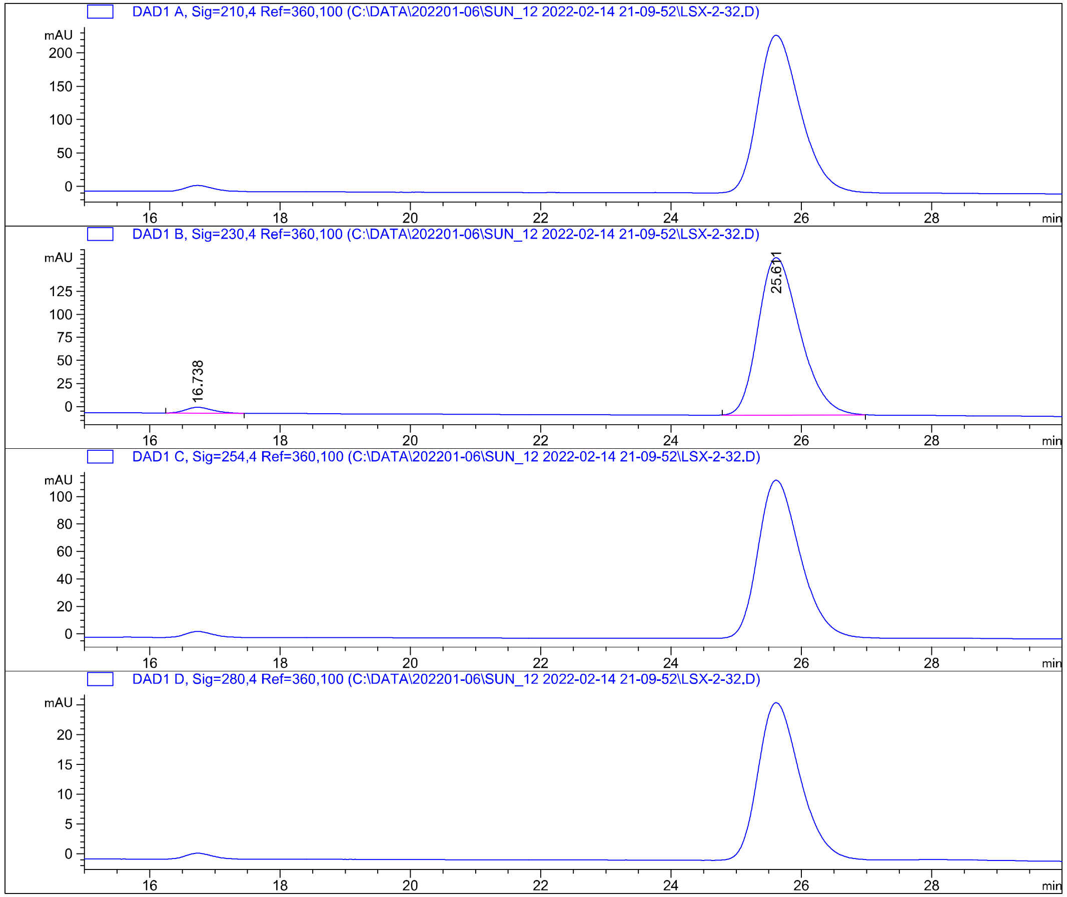

Data File C:\DATA\202201-06\SUN_12 2022-02-14 21-09-52\LSX-2-32.D
Sample Name:

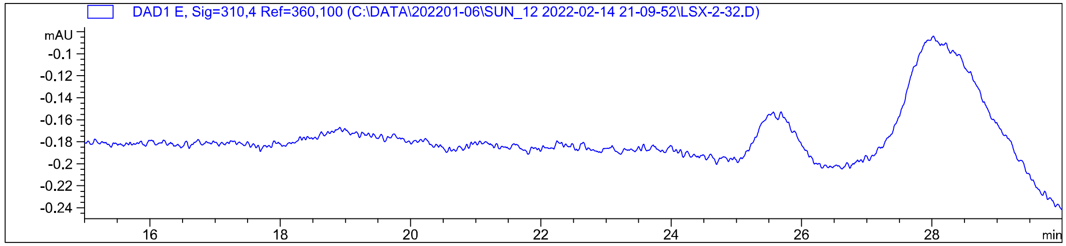


```
=====================================================================
                         Area Percent Report
=====================================================================

Sorted By             :      Signal
Multiplier            :      1.0000
Dilution              :      1.0000
Use Multiplier & Dilution Factor with ISTDs


Signal 1: DAD1 A, Sig=210,4 Ref=360,100


Signal 2: DAD1 B, Sig=230,4 Ref=360,100

Peak RetTime Type  Width     Area      Height     Area
  #   [min]        [min]   [mAU*s]     [mAU]       %
----|-------|----|-------|----------|----------|--------|
   1  16.738 BB    0.4070  178.94763    6.30701   2.2903
   2  25.611 BB    0.6872 7634.36328  170.87814  97.7097

Totals :                  7813.31091  177.18515


Signal 3: DAD1 C, Sig=254,4 Ref=360,100


Signal 4: DAD1 D, Sig=280,4 Ref=360,100


Signal 5: DAD1 E, Sig=310,4 Ref=360,100

=====================================================================
                         *** End of Report ***
```

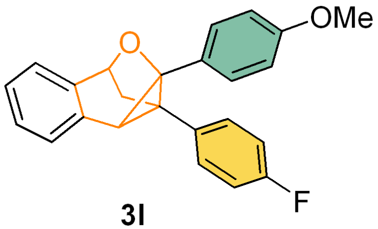

3l

Data File C:\DATA\202207-12\SUN_12 2022-07-27 23-10-28\LSX-P3-NAPTH-RAC.D
Sample Name:

===
Acq. Operator : Seq. Line : 5
Acq. Instrument : Instrument 1 Location : 74
Injection Date : 27/7/2022 9:56:54 am Inj : 1
Inj Volume : 5.000 µl
Different Inj Volume from Sample Entry! Actual Inj Volume : 10.000 µl
Acq. Method : C:\Chem32\1\DATA\SUN_12 2022-07-27 23-10-28\IC-02-30.M
Last changed :
(modified after loading)
Analysis Method : C:\Users\Public\Documents\ChemStation\1\Data\SUN\SUN 2024-03-13 17-26-47\OD-00
-20.M (Sequence Method)
Last changed : 13/3/2024 10:47:51 pm by SYSTEM
(modified after loading)
Additional Info : Peak(s) manually integrated

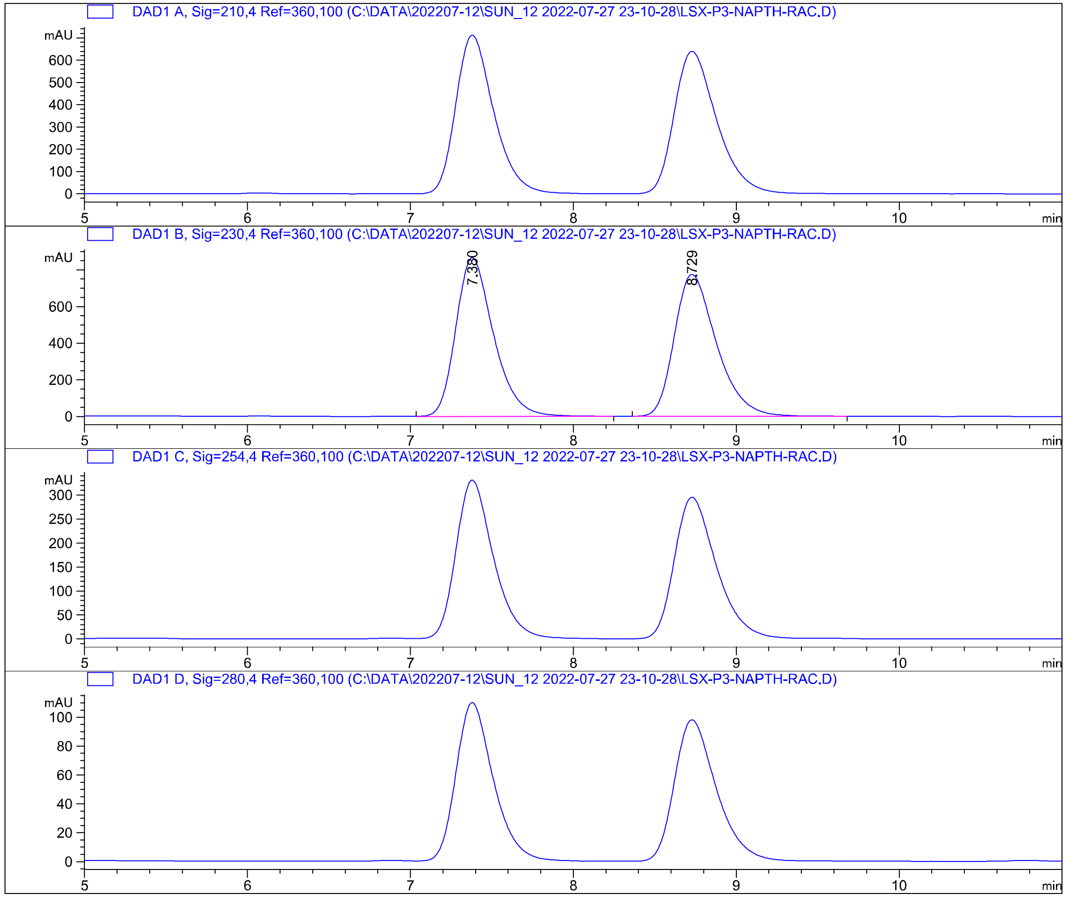

Data File C:\DATA\202207-12\SUN_12 2022-07-27 23-10-28\LSX-P3-NAPTH-RAC.D
Sample Name:

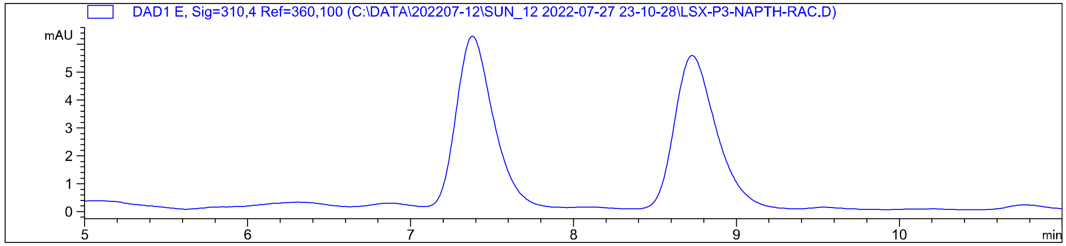

===
Area Percent Report
===

Sorted By : Signal
Multiplier : 1.0000
Dilution : 1.0000
Use Multiplier & Dilution Factor with ISTDs

Signal 1: DAD1 A, Sig=210,4 Ref=360,100

Signal 2: DAD1 B, Sig=230,4 Ref=360,100

Peak #	RetTime [min]	Type	Width [min]	Area [mAU*s]	Height [mAU]	Area %
1	7.380	BB	0.2440	1.39588e4	866.09265	50.0258
2	8.729	BB	0.2741	1.39444e4	774.32813	49.9742

Totals : 2.79032e4 1640.42078

Signal 3: DAD1 C, Sig=254,4 Ref=360,100

Signal 4: DAD1 D, Sig=280,4 Ref=360,100

Signal 5: DAD1 E, Sig=310,4 Ref=360,100

===
*** End of Report ***

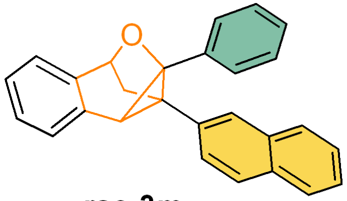

rac-3m

Data File C:\DATA\202201-06\SUN_12 2022-05-06 19-51-00\LSX-2-142-FR.D
Sample Name:

```
=====================================================================
Acq. Operator   :                               Seq. Line :    3
Acq. Instrument : Instrument 1                   Location :   42
Injection Date  : 6/5/2022 5:35:06 am                 Inj :    1
                                               Inj Volume : 5.000 µl
Different Inj Volume from Sample Entry!  Actual Inj Volume : 10.000 µl
Acq. Method     : C:\Chem32\1\DATA\SUN_12 2022-05-06 19-51-00\IC-02-30.M
Last changed    :
                  (modified after loading)
Analysis Method : C:\Users\Public\Documents\ChemStation\1\Data\SUN\SUN 2024-03-13 17-26-47\OD-00
                  -20.M (Sequence Method)
Last changed    : 13/3/2024 10:47:51 pm by SYSTEM
                  (modified after loading)
Additional Info : Peak(s) manually integrated
```

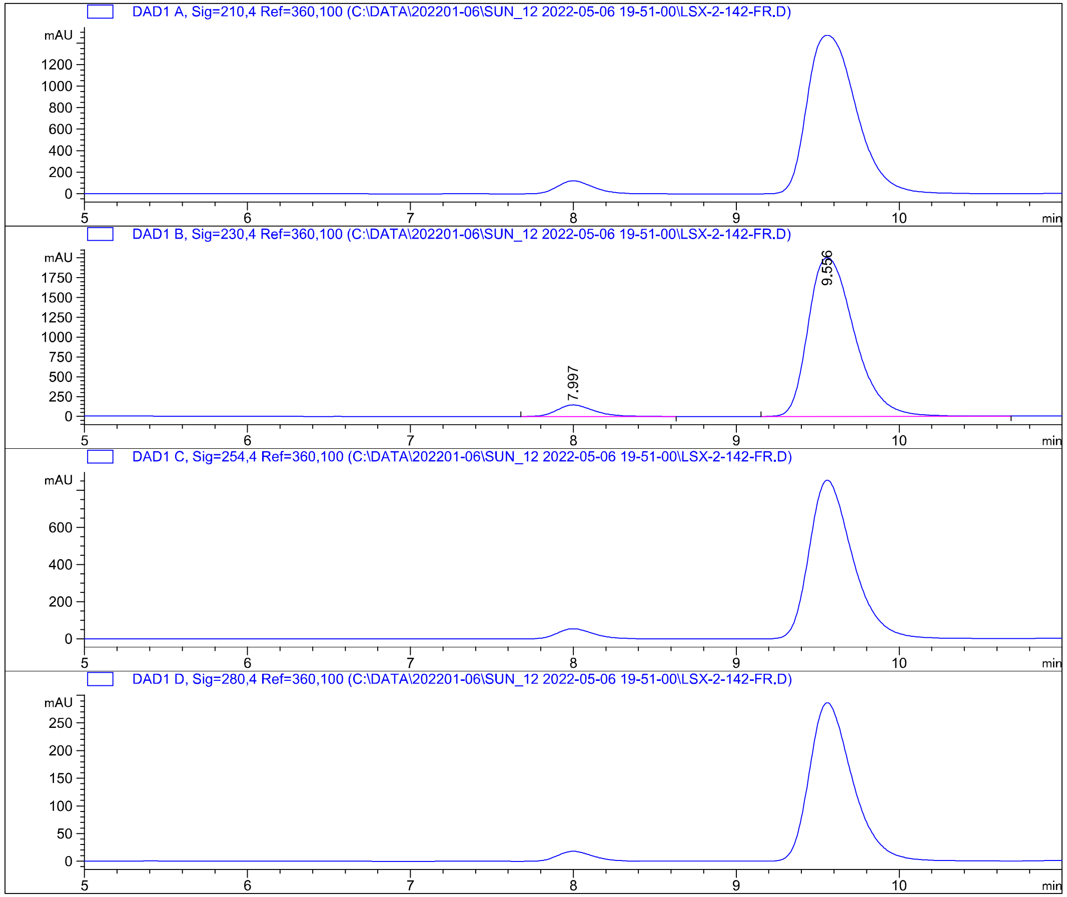

Data File C:\DATA\202201-06\SUN_12 2022-05-06 19-51-00\LSX-2-142-FR.D
Sample Name:

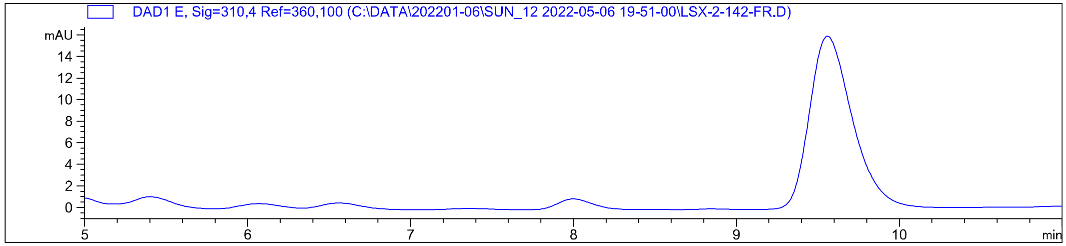


```
=====================================================================
                        Area Percent Report
=====================================================================

Sorted By             :      Signal
Multiplier            :      1.0000
Dilution              :      1.0000
Use Multiplier & Dilution Factor with ISTDs


Signal 1: DAD1 A, Sig=210,4 Ref=360,100


Signal 2: DAD1 B, Sig=230,4 Ref=360,100
```

Peak #	RetTime [min]	Type	Width [min]	Area [mAU*s]	Height [mAU]	Area %
1	7.997	BB	0.2361	2240.99194	145.19551	5.3680
2	9.556	BB	0.3080	3.95061e4	2004.24268	94.6320

```
Totals :                    4.17470e4  2149.43819


Signal 3: DAD1 C, Sig=254,4 Ref=360,100


Signal 4: DAD1 D, Sig=280,4 Ref=360,100


Signal 5: DAD1 E, Sig=310,4 Ref=360,100

=====================================================================
                        *** End of Report ***
```

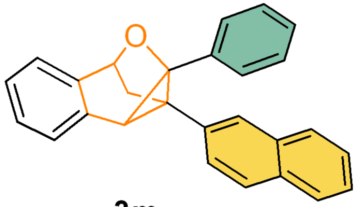

3m

===

```
Acq. Operator   :                           Seq. Line :  13
Acq. Instrument : Instrument 1               Location :   42
Injection Date  : 1/8/2022 11:58:09 am            Inj :   1
                                           Inj Volume : 5.000 µl
Acq. Method     : C:\Chem32\1\DATA\SUN_12 2022-08-01 21-33-13\AD-03-30-0.5.M
Last changed    :
Analysis Method : C:\Users\Public\Documents\ChemStation\1\Data\SUN\SUN 2024-03-13 17-26-47\OD-00
                  -20.M (Sequence Method)
Last changed    : 13/3/2024 10:41:01 pm by SYSTEM
                  (modified after loading)
Additional Info : Peak(s) manually integrated
```

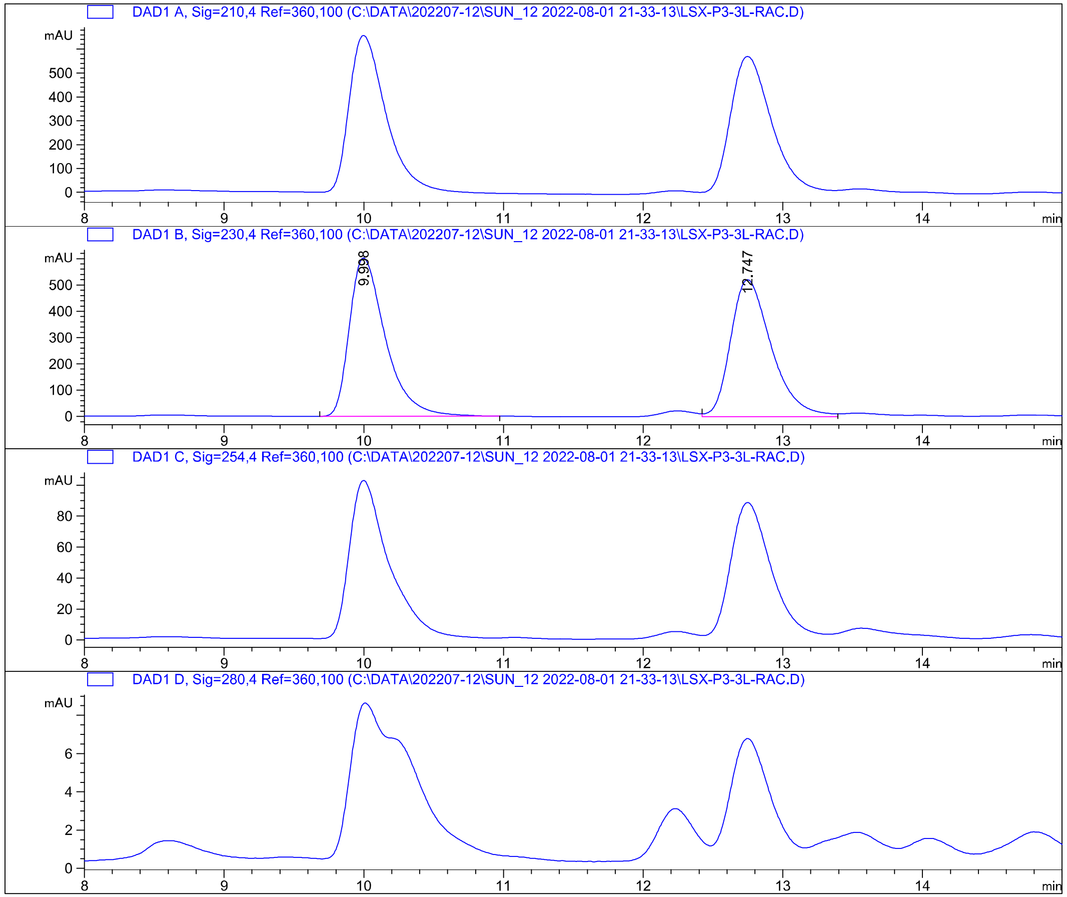

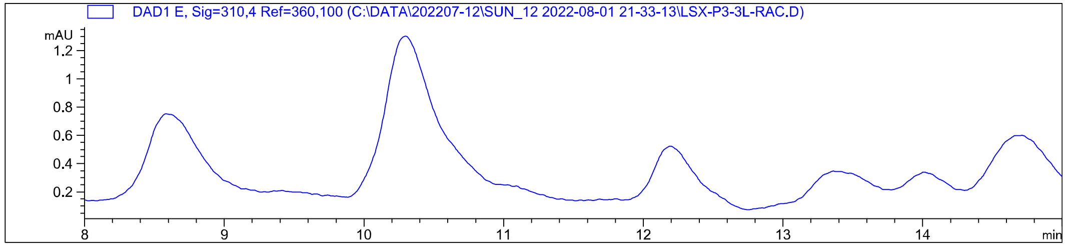

===

Area Percent Report

===

```
Sorted By             :      Signal
Multiplier            :      1.0000
Dilution              :      1.0000
Use Multiplier & Dilution Factor with ISTDs
```

Signal 1: DAD1 A, Sig=210,4 Ref=360,100

Signal 2: DAD1 B, Sig=230,4 Ref=360,100

Peak #	RetTime [min]	Type	Width [min]	Area [mAU*s]	Height [mAU]	Area %
1	9.998	BB	0.2731	1.08005e4	602.47620	50.4473
2	12.747	VV	0.3114	1.06090e4	521.29974	49.5527

Totals : 2.14095e4 1123.77594

Signal 3: DAD1 C, Sig=254,4 Ref=360,100

Signal 4: DAD1 D, Sig=280,4 Ref=360,100

Signal 5: DAD1 E, Sig=310,4 Ref=360,100

===

*** End of Report ***

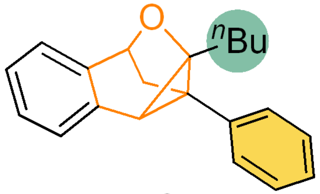

rac-3n

Data File C:\DATA\202109-1231\SUN_12 2021-12-13 22-20-33\LSX-1-147.D
Sample Name:

Acq. Operator : Seq. Line : 2
Acq. Instrument : Instrument 1 Location : 41
Injection Date : 13/12/2021 6:33:17 am Inj : 1
Inj Volume : 5.000 µl
Acq. Method : C:\Chem32\1\DATA\SUN_12 2021-12-13 22-20-33\AD-03-30-0.5.M
Last changed :
Analysis Method : C:\Users\Public\Documents\ChemStation\1\Data\SUN\SUN 2024-03-13 17-26-47\OD-00-20.M (Sequence Method)
Last changed : 13/3/2024 10:41:01 pm by SYSTEM
(modified after loading)
Additional Info : Peak(s) manually integrated

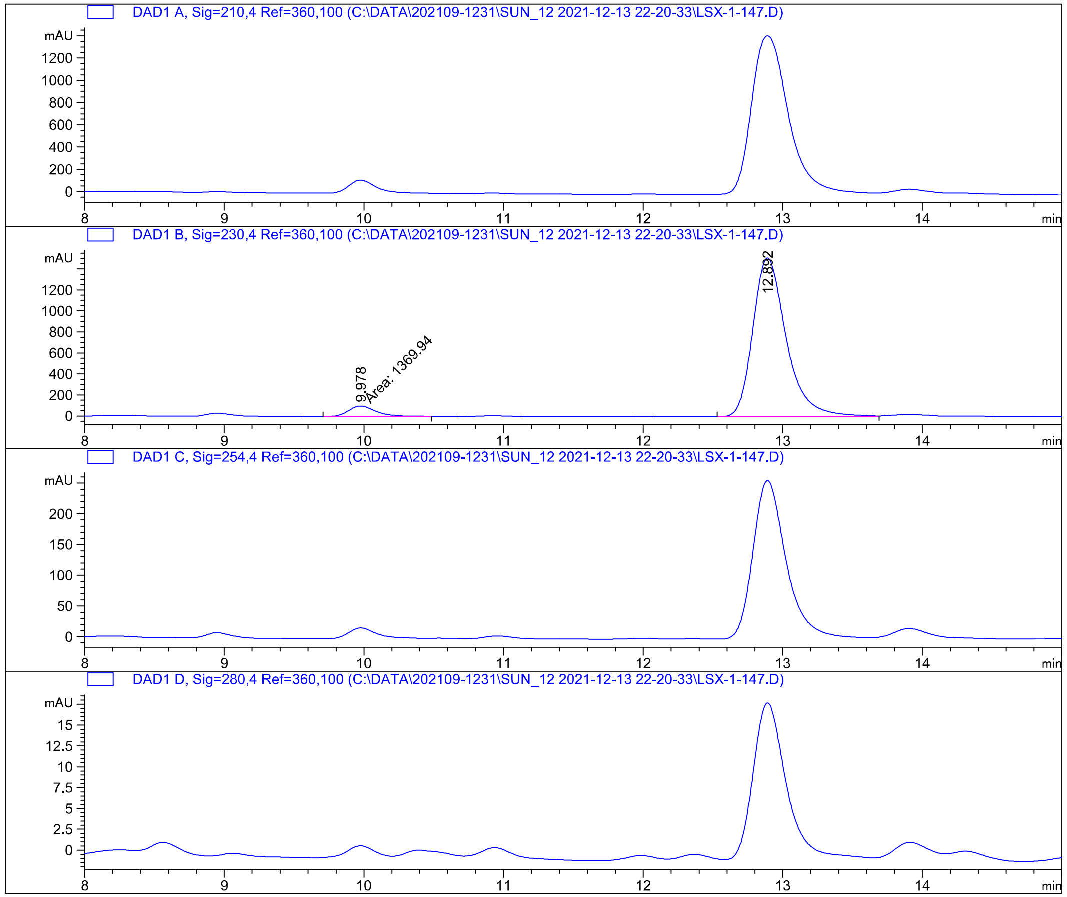

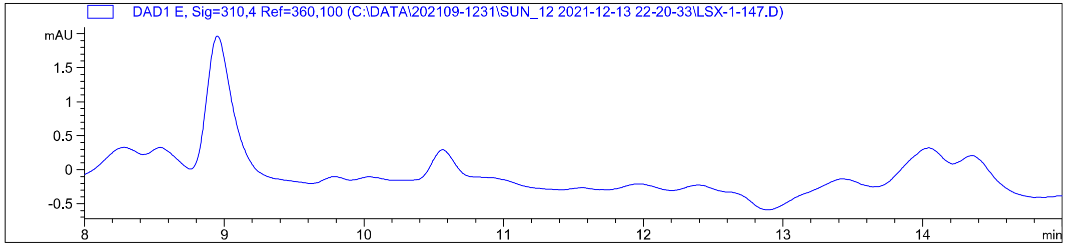

Area Percent Report

Sorted By : Signal
Multiplier : 1.0000
Dilution : 1.0000
Use Multiplier & Dilution Factor with ISTDs

Signal 1: DAD1 A, Sig=210,4 Ref=360,100

Signal 2: DAD1 B, Sig=230,4 Ref=360,100

Peak #	RetTime [min]	Type	Width [min]	Area [mAU*s]	Height [mAU]	Area %
1	9.978	MM	0.2271	1369.93945	100.54881	5.1421
2	12.892	BV	0.2551	2.52716e4	1510.90625	94.8579

Totals : 2.66416e4 1611.45506

Signal 3: DAD1 C, Sig=254,4 Ref=360,100

Signal 4: DAD1 D, Sig=280,4 Ref=360,100

Signal 5: DAD1 E, Sig=310,4 Ref=360,100

*** End of Report ***

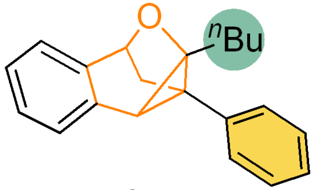

3n

Data File C:\DATA\202207-12\SUN_12 2022-08-02 10-25-51\LSX-P3-3M-RAC.D
Sample Name:

===

Acq. Operator	:	Seq. Line :	6
Acq. Instrument	: Instrument 1	Location :	42
Injection Date	: 1/8/2022 9:32:52 pm	Inj :	1
		Inj Volume :	5.000 µl

Different Inj Volume from Sample Entry! Actual Inj Volume : 10.000 µl
Acq. Method : C:\Chem32\1\DATA\SUN_12 2022-08-02 10-25-51\AD-02-30.M
Last changed :
(modified after loading)
Analysis Method : C:\Users\Public\Documents\ChemStation\1\Data\SUN\SUN 2024-03-13 17-26-47\OD-00-20.M (Sequence Method)
Last changed : 13/3/2024 10:44:59 pm by SYSTEM
(modified after loading)
Additional Info : Peak(s) manually integrated

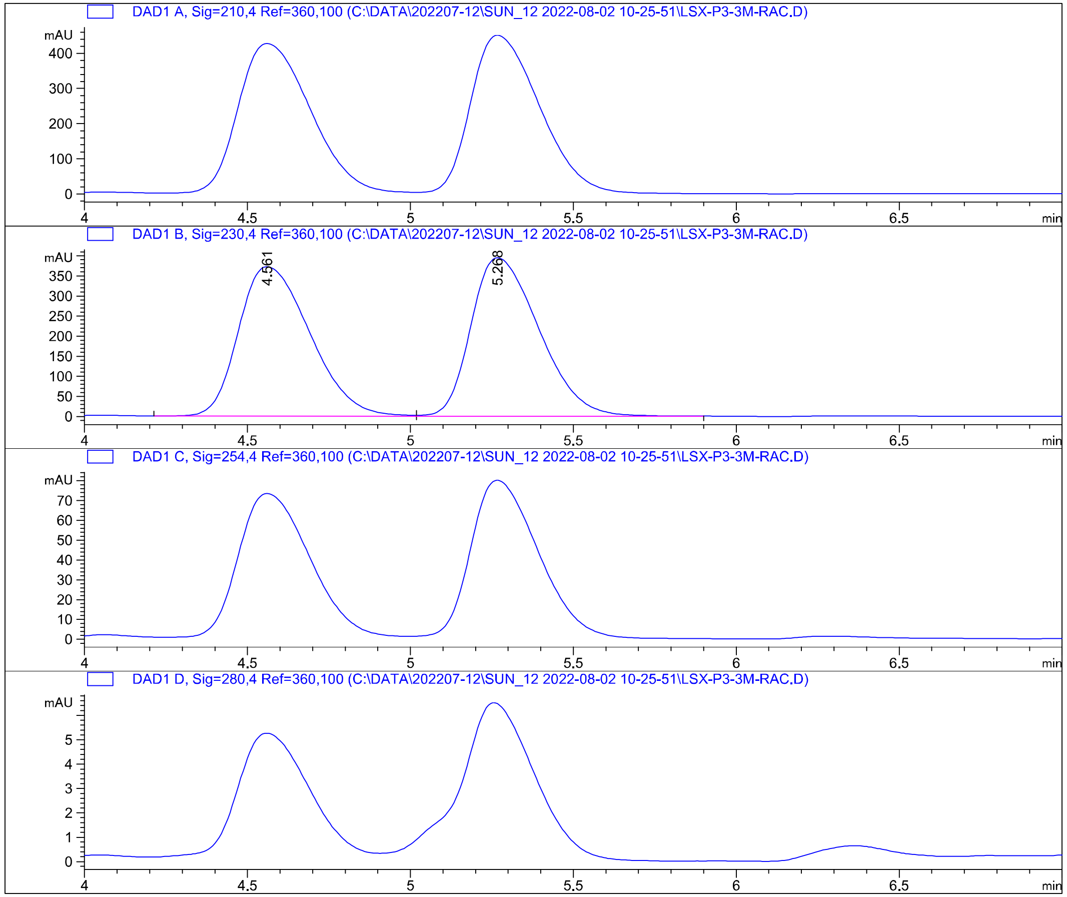

Data File C:\DATA\202207-12\SUN_12 2022-08-02 10-25-51\LSX-P3-3M-RAC.D
Sample Name:

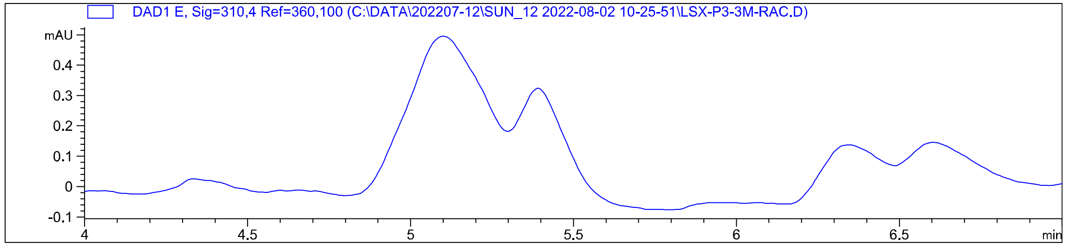

===
Area Percent Report
===

Sorted By : Signal
Multiplier : 1.0000
Dilution : 1.0000
Use Multiplier & Dilution Factor with ISTDs

Signal 1: DAD1 A, Sig=210,4 Ref=360,100

Signal 2: DAD1 B, Sig=230,4 Ref=360,100

Peak #	RetTime [min]	Type	Width [min]	Area [mAU*s]	Height [mAU]	Area %
1	4.561	VV	0.2414	5656.20166	371.97165	49.7169
2	5.268	VB	0.2292	5720.62207	394.34897	50.2831

Totals : 1.13768e4 766.32062

Signal 3: DAD1 C, Sig=254,4 Ref=360,100

Signal 4: DAD1 D, Sig=280,4 Ref=360,100

Signal 5: DAD1 E, Sig=310,4 Ref=360,100

===
*** End of Report ***

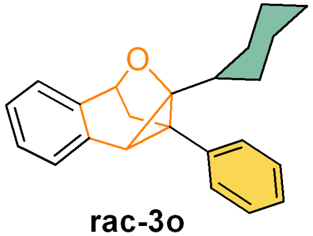

rac-3o

Data File C:\DATA\202201-06\SUN_12 2022-06-06 19-12-01\LSX-3-12-FR.D
Sample Name:

```
=====================================================================
Acq. Operator   :                                 Seq. Line :  14
Acq. Instrument : Instrument 1                     Location :   41
Injection Date  : 6/6/2022 7:43:36 am                   Inj :   1
                                                 Inj Volume : 5.000 µl
Different Inj Volume from Sample Entry!  Actual Inj Volume : 8.000 µl
Acq. Method     : C:\Chem32\1\DATA\SUN_12 2022-06-06 19-12-01\AD-02-30.M
Last changed    :
                  (modified after loading)
Analysis Method : C:\Users\Public\Documents\ChemStation\1\Data\SUN\SUN 2024-03-13 17-26-47\OD-00
                  -20.M (Sequence Method)
Last changed    : 13/3/2024 10:44:59 pm by SYSTEM
                  (modified after loading)
Additional Info : Peak(s) manually integrated
```

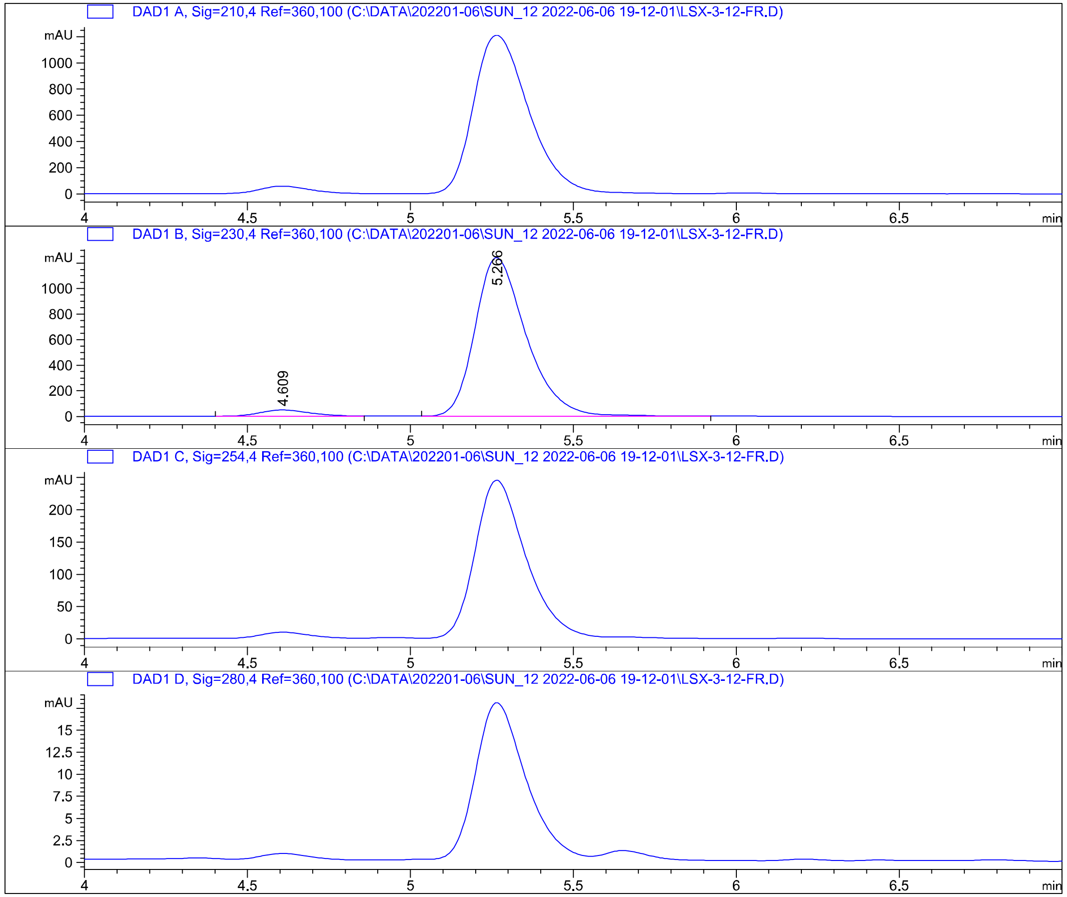

Data File C:\DATA\202201-06\SUN_12 2022-06-06 19-12-01\LSX-3-12-FR.D
Sample Name:

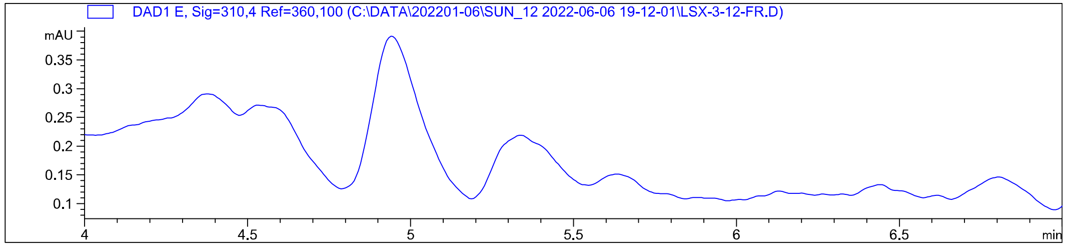


```
=====================================================================
                         Area Percent Report
=====================================================================

Sorted By             :      Signal
Multiplier            :      1.0000
Dilution              :      1.0000
Use Multiplier & Dilution Factor with ISTDs


Signal 1: DAD1 A, Sig=210,4 Ref=360,100


Signal 2: DAD1 B, Sig=230,4 Ref=360,100

Peak RetTime Type  Width     Area      Height     Area
  #   [min]        [min]   [mAU*s]     [mAU]        %
----|-------|----|-------|----------|----------|--------|
   1   4.609 BV    0.1649  530.77295   48.80680   3.7515
   2   5.266 VB    0.1660 1.36176e4  1241.16309  96.2485

Totals :                  1.41484e4  1289.96989


Signal 3: DAD1 C, Sig=254,4 Ref=360,100


Signal 4: DAD1 D, Sig=280,4 Ref=360,100


Signal 5: DAD1 E, Sig=310,4 Ref=360,100

=====================================================================
                        *** End of Report ***
```

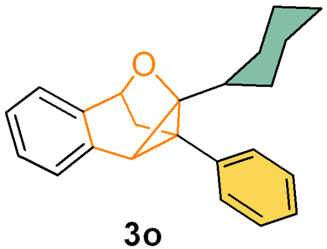

3o

Data File C:\Users\P...\ChemStation\1\Data\SUN\SUN 2024-07-21 14-34-56\OnlineEdited--007.D
Sample Name: lsx-8-143crac

===

Acq. Operator : SYSTEM   Seq. Line : 7
Sample Operator : SYSTEM
Acq. Instrument : HPLC   Location : P1-D-01
Injection Date : 21/7/2024 3:54:36 pm   Inj : 1
Inj Volume : 2.000 µl
Different Inj Volume from Sample Entry! Actual Inj Volume : 10.000 µl
Acq. Method : C:\Users\Public\Documents\ChemStation\1\Data\SUN\SUN 2024-07-21 14-34-56\IC3-2-25.M
Last changed : 2/7/2024 6:32:09 pm by SYSTEM
Analysis Method : C:\Users\Public\Documents\ChemStation\1\Data\SUN\SUN 2024-07-21 14-34-56\IC3-2-25.M (Sequence Method)
Last changed : 22/7/2024 3:53:30 pm by SYSTEM
(modified after loading)
Additional Info : Peak(s) manually integrated

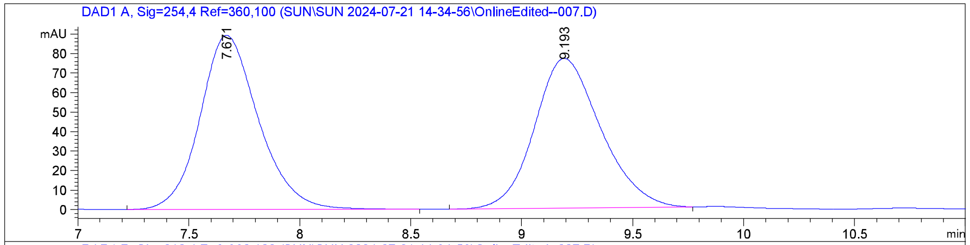

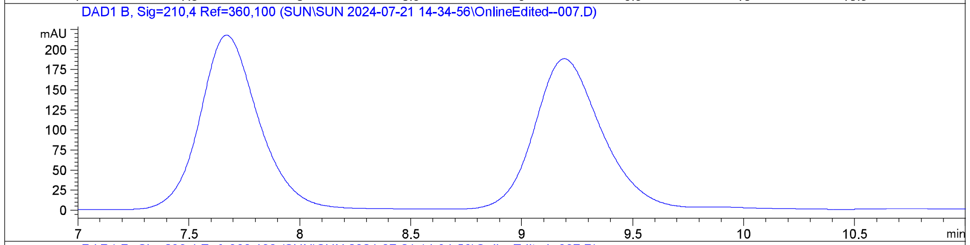

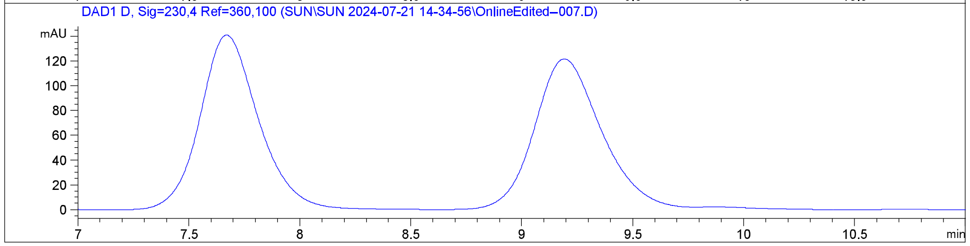

Data File C:\Users\P...\ChemStation\1\Data\SUN\SUN 2024-07-21 14-34-56\OnlineEdited--007.D
Sample Name: lsx-8-143crac

===

Area Percent Report

===

Sorted By : Signal
Multiplier : 1.0000
Dilution : 1.0000
Use Multiplier & Dilution Factor with ISTDs

Signal 1: DAD1 A, Sig=254,4 Ref=360,100

Peak #	RetTime [min]	Type	Width [min]	Area [mAU*s]	Height [mAU]	Area %
1	7.671	BB	0.2776	1639.43286	89.33002	50.6508
2	9.193	BB	0.3157	1597.30408	76.77734	49.3492
Totals :				3236.73694	166.10736	

Signal 2: DAD1 B, Sig=210,4 Ref=360,100

Signal 3: DAD1 D, Sig=230,4 Ref=360,100

===

*** End of Report ***

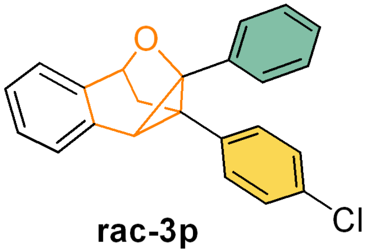

rac-3p

===

Acq. Operator : SYSTEM   Seq. Line : 3
Sample Operator : SYSTEM
Acq. Instrument : HPLC   Location : P1-D-01
Injection Date : 21/7/2024 9:44:59 pm   Inj : 1
Inj Volume : 2.000 µl
Different Inj Volume from Sample Entry! Actual Inj Volume : 10.000 µl
Acq. Method : C:\Users\Public\Documents\ChemStation\1\Data\SUN\SUN 2024-07-21 21-27-19 \IC3-2-25.M
Last changed : 2/7/2024 6:32:09 pm by SYSTEM
Analysis Method : C:\Users\Public\Documents\ChemStation\1\Data\SUN\SUN 2024-07-21 21-27-19 \IC3-2-25.M (Sequence Method)
Last changed : 22/7/2024 3:54:28 pm by SYSTEM
(modified after loading)
Additional Info : Peak(s) manually integrated

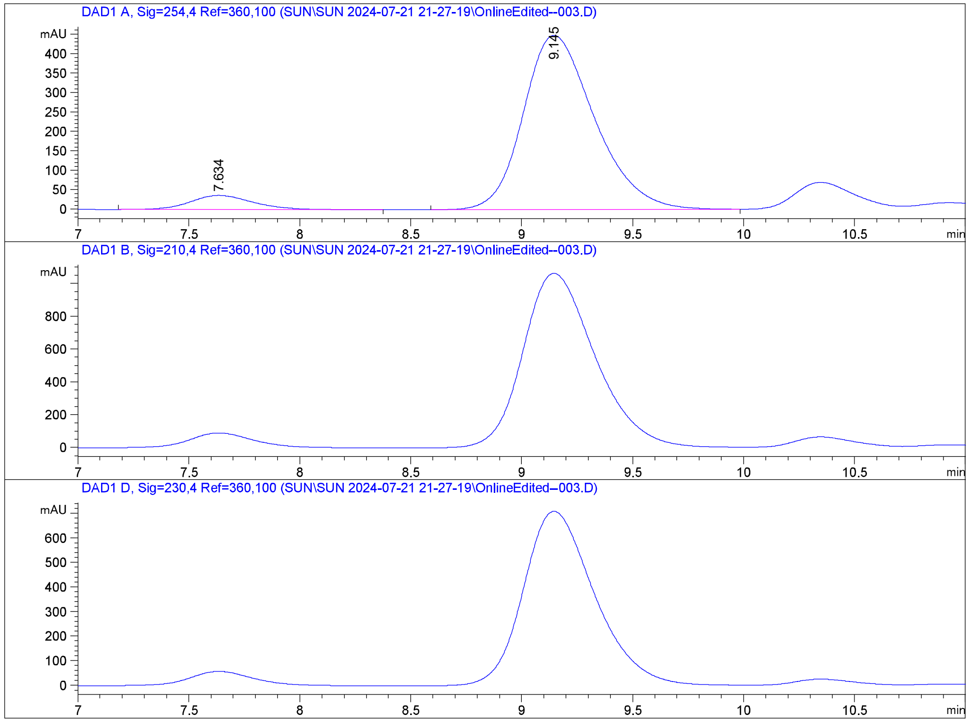

Data File C:\Users\P...\ChemStation\1\Data\SUN\SUN 2024-07-21 21-27-19\OnlineEdited--003.D
Sample Name: lsx-8-143crac

===

Area Percent Report

===

Sorted By : Signal
Multiplier : 1.0000
Dilution : 1.0000
Use Multiplier & Dilution Factor with ISTDs

Signal 1: DAD1 A, Sig=254,4 Ref=360,100

Peak #	RetTime [min]	Type	Width [min]	Area [mAU*s]	Height [mAU]	Area %
1	7.634	BB	0.2946	724.15308	36.62976	6.7459
2	9.145	BB	0.3398	1.00105e4	447.76245	93.2541

Totals : 1.07346e4 484.39222

Signal 2: DAD1 B, Sig=210,4 Ref=360,100

Signal 3: DAD1 D, Sig=230,4 Ref=360,100

===

*** End of Report ***

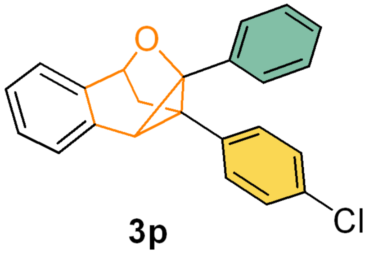

3p

===

Acq. Operator : SYSTEM Seq. Line : 9
Sample Operator : SYSTEM
Acq. Instrument : HPLC Location : P1-D-03
Injection Date : 21/7/2024 4:47:34 pm Inj : 1
Inj Volume : 2.000 µl
Different Inj Volume from Sample Entry! Actual Inj Volume : 10.000 µl
Acq. Method : C:\Users\Public\Documents\ChemStation\1\Data\SUN\SUN 2024-07-21 14-34-56\IC3-2-25.M
Last changed : 2/7/2024 6:32:09 pm by SYSTEM
Analysis Method : C:\Users\Public\Documents\ChemStation\1\Data\SUN\SUN 2024-07-21 14-34-56\IC3-2-25.M (Sequence Method)
Last changed : 22/7/2024 3:51:26 pm by SYSTEM
(modified after loading)
Additional Info : Peak(s) manually integrated

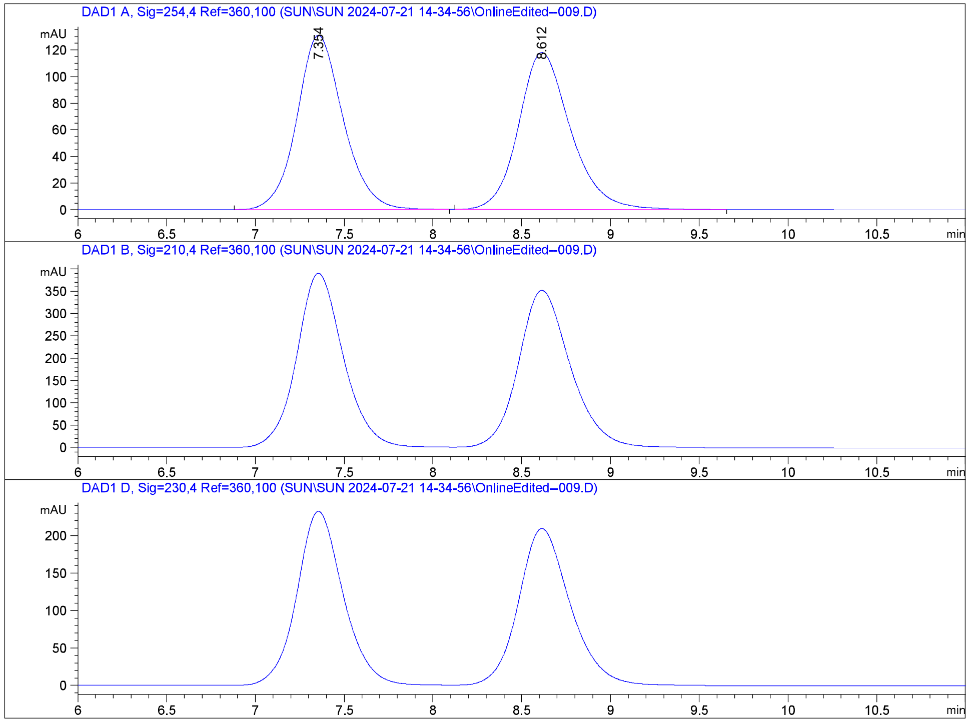

Data File C:\Users\P...\ChemStation\1\Data\SUN\SUN 2024-07-21 14-34-56\OnlineEdited--009.D
Sample Name: lsx-8-143brac

===
Area Percent Report
===

Sorted By : Signal
Multiplier : 1.0000
Dilution : 1.0000
Use Multiplier & Dilution Factor with ISTDs

Signal 1: DAD1 A, Sig=254,4 Ref=360,100

Peak #	RetTime [min]	Type	Width [min]	Area [mAU*s]	Height [mAU]	Area %
1	7.354	BB	0.2712	2371.44141	130.66690	49.6549
2	8.612	BB	0.3101	2404.39990	117.79632	50.3451

Totals : 4775.84131 248.46322

Signal 2: DAD1 B, Sig=210,4 Ref=360,100

Signal 3: DAD1 D, Sig=230,4 Ref=360,100

===
*** End of Report ***

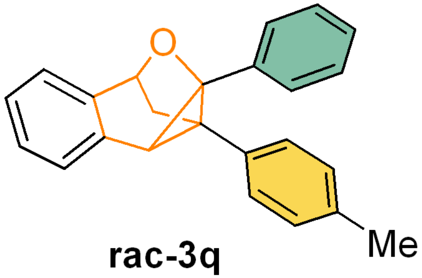

rac-3q

===

```
Acq. Operator   : SYSTEM                        Seq. Line :    2
Sample Operator : SYSTEM
Acq. Instrument : HPLC                           Location :   P1-D-01
Injection Date  : 22/7/2024 12:48:04 pm               Inj :    1
                                               Inj Volume : 2.000 µl
Different Inj Volume from Sample Entry!  Actual Inj Volume : 8.000 µl
Acq. Method     : C:\Users\Public\Documents\ChemStation\1\Data\SUN\SUN 2024-07-22 12-34-21
                  \IC3-2-25.M
Last changed    : 2/7/2024 6:32:09 pm by SYSTEM
Analysis Method : C:\Users\Public\Documents\ChemStation\1\Data\SUN\SUN 2024-07-22 12-34-21
                  \IC3-2-25.M (Sequence Method)
Last changed    : 22/7/2024 3:52:38 pm by SYSTEM
                  (modified after loading)
Additional Info : Peak(s) manually integrated
```

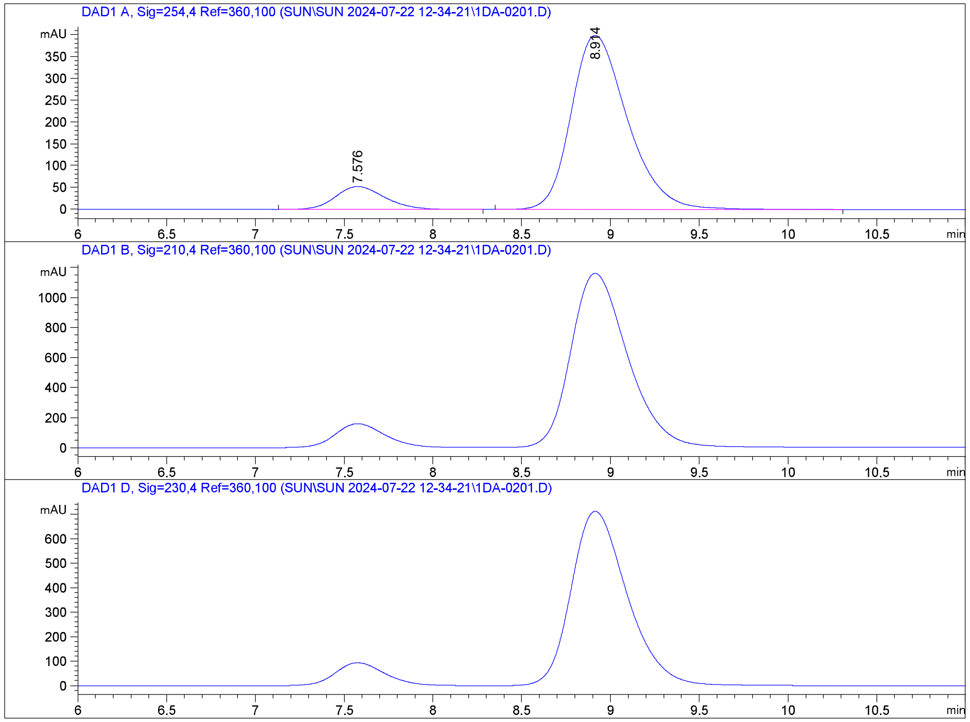

Data File C:\Users\P...Documents\ChemStation\1\Data\SUN\SUN 2024-07-22 12-34-21\1DA-0201.D
Sample Name: lsx-8-143brt

===

Area Percent Report

===

```
Sorted By          :      Signal
Multiplier         :      1.0000
Dilution           :      1.0000
Use Multiplier & Dilution Factor with ISTDs
```

Signal 1: DAD1 A, Sig=254,4 Ref=360,100

Peak #	RetTime [min]	Type	Width [min]	Area [mAU*s]	Height [mAU]	Area %
1	7.576	BB	0.2970	1029.88721	52.81315	10.4569
2	8.914	BB	0.3380	8819.01270	399.61432	89.5431

Totals : 9848.89990 452.42747

Signal 2: DAD1 B, Sig=210,4 Ref=360,100

Signal 3: DAD1 D, Sig=230,4 Ref=360,100

===

*** End of Report ***

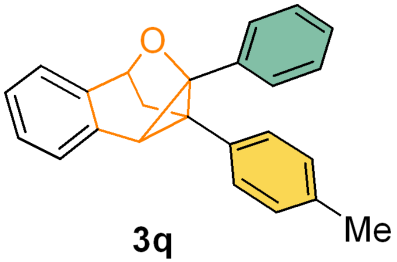

3q

Data File C:\Users\P...\ChemStation\1\Data\SUN\SUN 2024-07-07 14-44-18\OnlineEdited--015.D
Sample Name: lsx-8-120rac

===

```
Acq. Operator   : SYSTEM                    Seq. Line :  15
Sample Operator : SYSTEM
Acq. Instrument : HPLC                       Location :   P1-D-03
Injection Date  : 7/7/2024 6:11:14 pm             Inj :   1
                                       Inj Volume : 2.000 µl
Different Inj Volume from Sample Entry!  Actual Inj Volume : 8.000 µl
Acq. Method     : C:\Users\Public\Documents\ChemStation\1\Data\SUN\SUN 2024-07-07 14-44-18
                  \IC3-2-25.M
Last changed    : 2/7/2024 6:32:09 pm by SYSTEM
Analysis Method : C:\Users\Public\Documents\ChemStation\1\Data\SUN\SUN 2024-07-07 14-44-18
                  \IC3-2-25.M (Sequence Method)
Last changed    : 22/7/2024 3:46:47 pm by SYSTEM
                  (modified after loading)
Additional Info : Peak(s) manually integrated
```

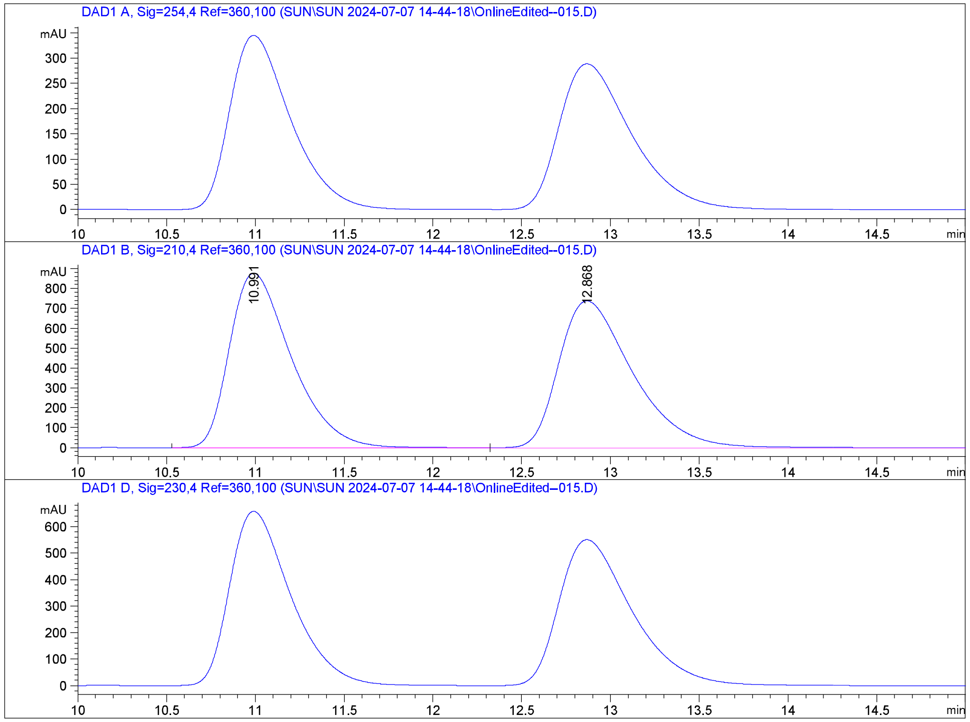

Data File C:\Users\P...\ChemStation\1\Data\SUN\SUN 2024-07-07 14-44-18\OnlineEdited--015.D
Sample Name: lsx-8-120rac

===

Area Percent Report

===

```
Sorted By            :      Signal
Multiplier           :      1.0000
Dilution             :      1.0000
Use Multiplier & Dilution Factor with ISTDs
```

Signal 1: DAD1 A, Sig=254,4 Ref=360,100

Signal 2: DAD1 B, Sig=210,4 Ref=360,100

Peak #	RetTime [min]	Type	Width [min]	Area [mAU*s]	Height [mAU]	Area %
1	10.991	BB	0.3685	2.12142e4	876.14178	49.8608
2	12.868	BB	0.4359	2.13327e4	737.73883	50.1392

Totals : 4.25469e4 1613.88062

Signal 3: DAD1 D, Sig=230,4 Ref=360,100

===

*** End of Report ***

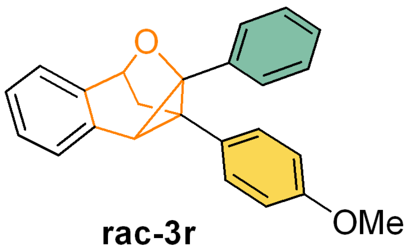

rac-3r

===

Acq. Operator	: SYSTEM	Seq. Line :	3
Sample Operator	: SYSTEM		
Acq. Instrument	: HPLC	Location :	P1-D-01
Injection Date	: 20/7/2024 3:23:26 pm	Inj :	1
		Inj Volume :	2.000 µl

Different Inj Volume from Sample Entry! Actual Inj Volume : 10.000 µl
Acq. Method : C:\Users\Public\Documents\ChemStation\1\Data\SUN\SUN 2024-07-20 14-53-17 \IC3-2-25.M
Last changed : 2/7/2024 6:32:09 pm by SYSTEM
Analysis Method : C:\Users\Public\Documents\ChemStation\1\Data\SUN\SUN 2024-07-20 14-53-17 \IC3-2-25.M (Sequence Method)
Last changed : 22/7/2024 3:39:01 pm by SYSTEM (modified after loading)
Additional Info : Peak(s) manually integrated

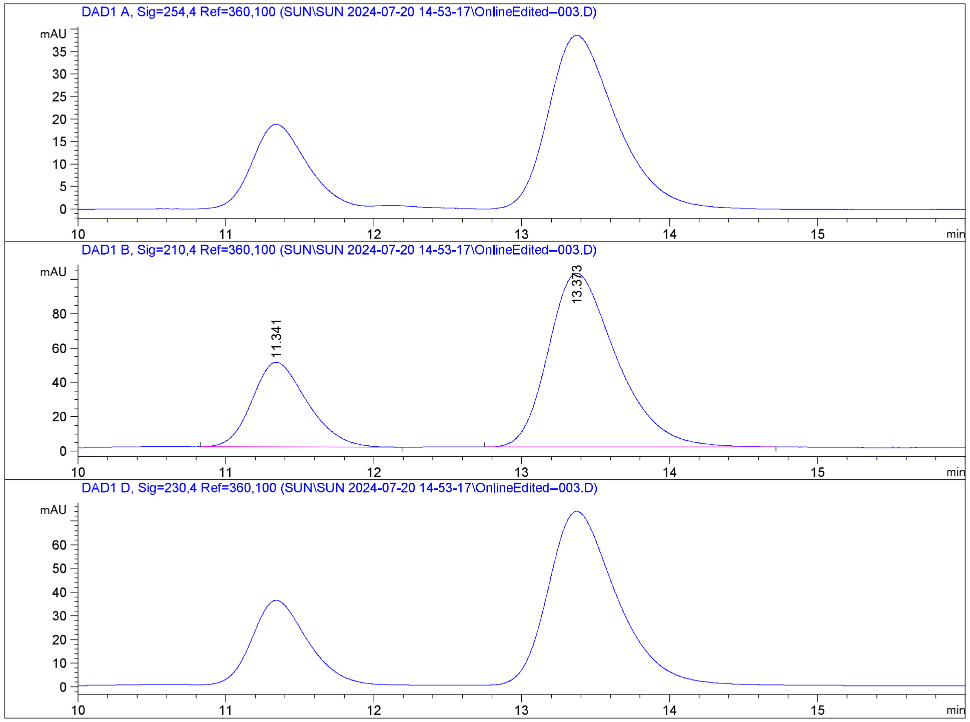

Data File C:\Users\P...\ChemStation\1\Data\SUN\SUN 2024-07-20 14-53-17\OnlineEdited--003.D
Sample Name: lsx-8-143a

===

Area Percent Report

===

Sorted By : Signal
Multiplier : 1.0000
Dilution : 1.0000
Use Multiplier & Dilution Factor with ISTDs

Signal 1: DAD1 A, Sig=254,4 Ref=360,100

Signal 2: DAD1 B, Sig=210,4 Ref=360,100

Peak #	RetTime [min]	Type	Width [min]	Area [mAU*s]	Height [mAU]	Area %
1	11.341	BB	0.3718	1274.70825	49.27628	28.2194
2	13.373	BB	0.4679	3242.43311	101.18215	71.7806

Totals : 4517.14136 150.45843

Signal 3: DAD1 D, Sig=230,4 Ref=360,100

===

*** End of Report ***

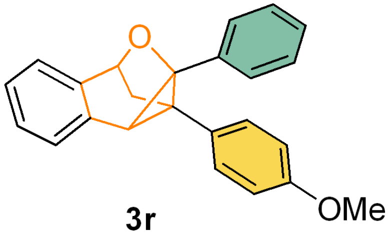

3r

Data File C:\Users\P...emStation\1\Data\SUN\SUN 2024-03-14 22-42-40\002-41-lsx-6-156-rac.D
Sample Name: lsx-6-156-rac

===

```
Acq. Operator   : SYSTEM                        Seq. Line :    2
Acq. Instrument : LC1260                         Location :   41
Injection Date  : 14/3/2024 10:55:26 pm               Inj :    1
                                               Inj Volume : 5.000 µl
Different Inj Volume from Sample Entry!  Actual Inj Volume : 3.000 µl
Acq. Method     : C:\Users\Public\Documents\ChemStation\1\Data\SUN\SUN 2024-03-14 22-42-40\OD-
                  005-30-0.5.M
Last changed    : 14/3/2024 10:54:38 pm by SYSTEM
                  (modified after loading)
Analysis Method : C:\Users\Public\Documents\ChemStation\1\Data\SUN\SUN 2024-03-14 22-42-40\OD-
                  005-30-0.5.M (Sequence Method)
Last changed    : 16/3/2024 11:03:28 am by SYSTEM
                  (modified after loading)
Additional Info : Peak(s) manually integrated
```

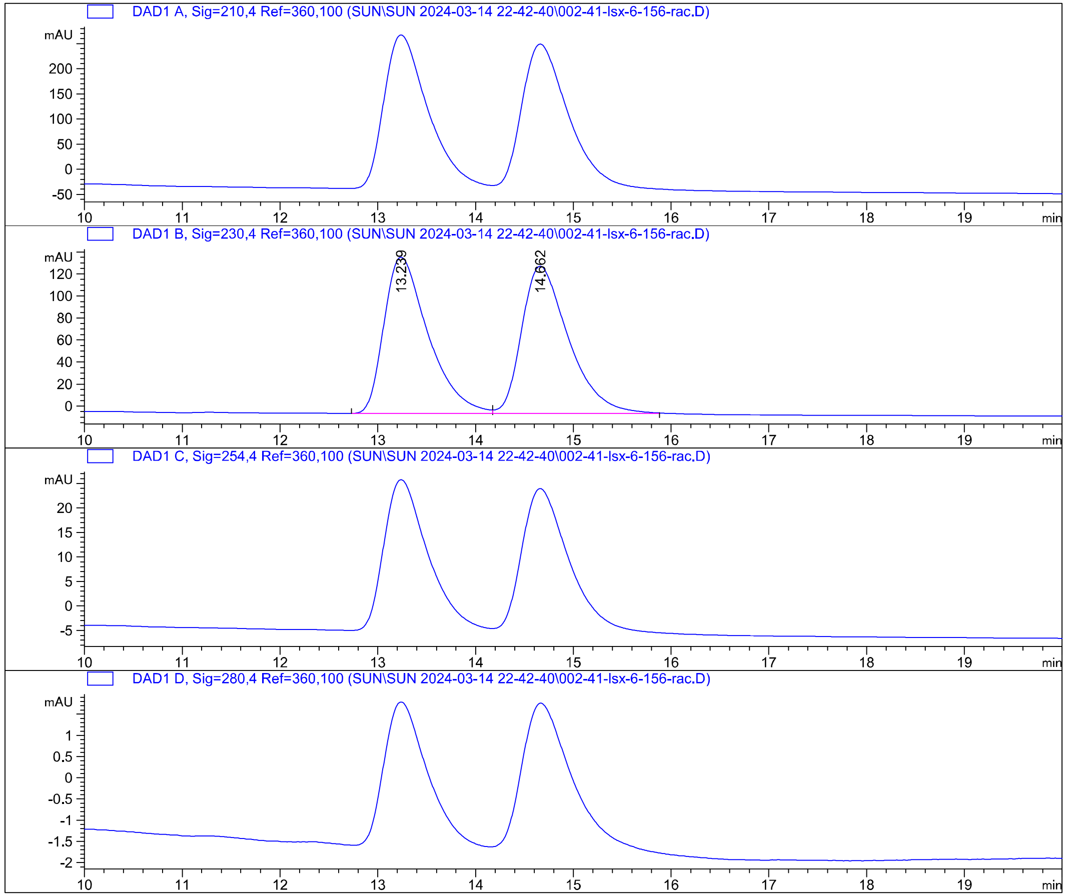

Data File C:\Users\P...emStation\1\Data\SUN\SUN 2024-03-14 22-42-40\002-41-lsx-6-156-rac.D
Sample Name: lsx-6-156-rac

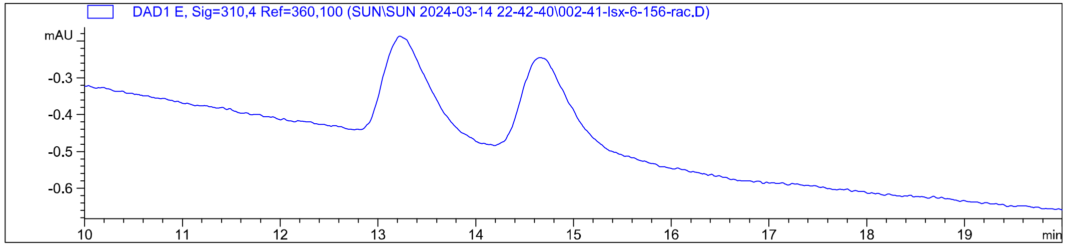

===

Area Percent Report

===

```
Sorted By             :      Signal
Multiplier            :      1.0000
Dilution              :      1.0000
Use Multiplier & Dilution Factor with ISTDs

Signal 1: DAD1 A, Sig=210,4 Ref=360,100

Signal 2: DAD1 B, Sig=230,4 Ref=360,100
```

Peak #	RetTime [min]	Type	Width [min]	Area [mAU*s]	Height [mAU]	Area %
1	13.239	BV	0.4763	4445.55176	141.59248	49.9927
2	14.662	VB	0.5044	4446.84326	133.58446	50.0073
Totals :				8892.39502	275.17694	

```
Signal 3: DAD1 C, Sig=254,4 Ref=360,100

Signal 4: DAD1 D, Sig=280,4 Ref=360,100

Signal 5: DAD1 E, Sig=310,4 Ref=360,100
```

===

*** End of Report ***

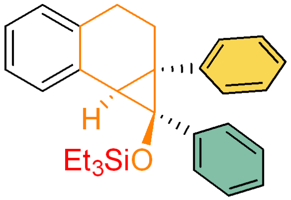

rac-4

Data File C:\DATA\202306-12\SUN_12 2023-10-23 15-11-35\LSX-6-156.D
Sample Name:

===

```
Acq. Operator   :                               Seq. Line :    2
Acq. Instrument : Instrument 1                   Location :   41
Injection Date  : 23/10/2023 12:24:27 am              Inj :    1
                                               Inj Volume : 5.000 µl
Different Inj Volume from Sample Entry!  Actual Inj Volume : 3.000 µl
Acq. Method     : C:\Chem32\1\DATA\SUN_12 2023-10-23 15-11-35\OD-005-30-0.5.M
Last changed    :
Analysis Method : C:\Users\Public\Documents\ChemStation\1\Data\SUN\SUN 2024-03-14 15-51-32\OD-00
                  -20.M (Sequence Method)
Last changed    : 14/3/2024 10:41:18 pm by SYSTEM
                  (modified after loading)
Additional Info : Peak(s) manually integrated
```

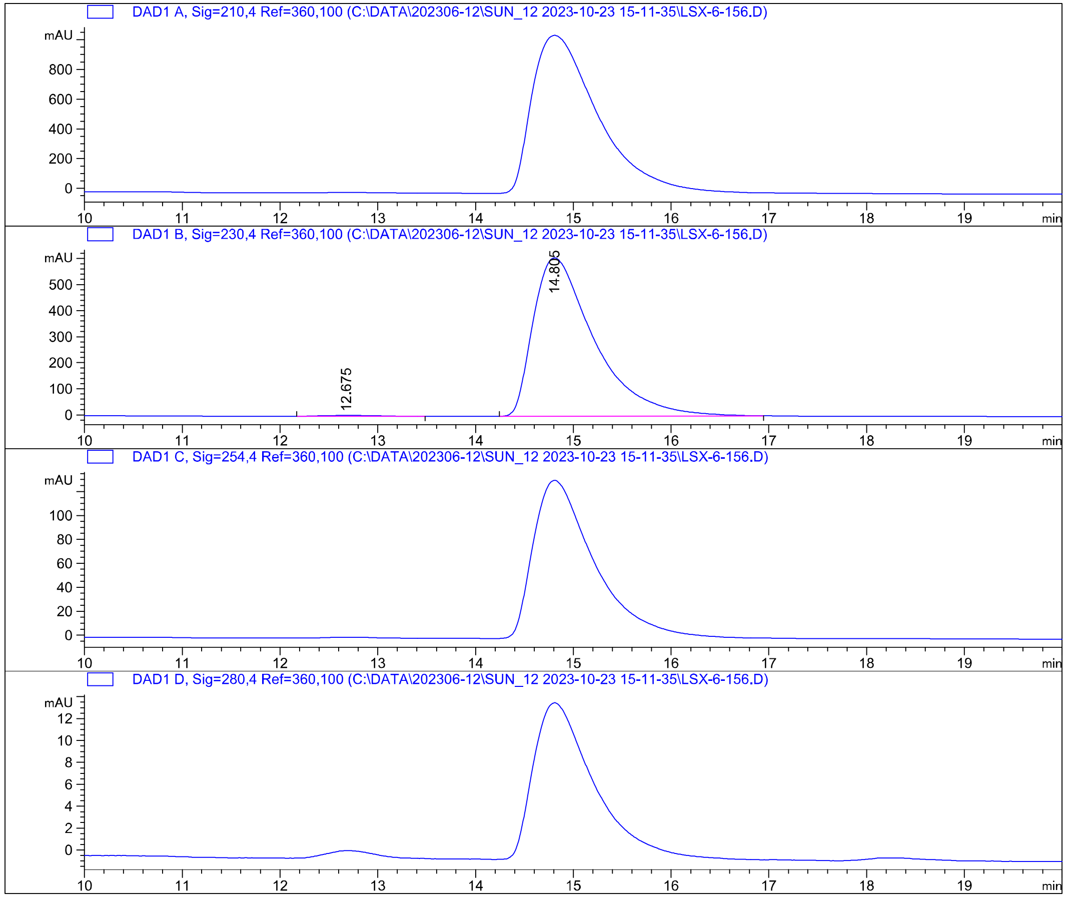

Data File C:\DATA\202306-12\SUN_12 2023-10-23 15-11-35\LSX-6-156.D
Sample Name:

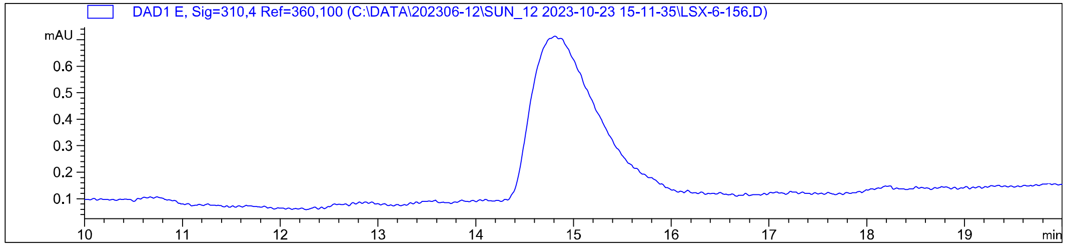

===

Area Percent Report

===

```
Sorted By             :      Signal
Multiplier            :      1.0000
Dilution              :      1.0000
Use Multiplier & Dilution Factor with ISTDs
```

Signal 1: DAD1 A, Sig=210,4 Ref=360,100

Signal 2: DAD1 B, Sig=230,4 Ref=360,100

Peak #	RetTime [min]	Type	Width [min]	Area [mAU*s]	Height [mAU]	Area %
1	12.675	BB	0.4218	114.07614	3.41453	0.4315
2	14.805	BB	0.6554	2.63224e4	607.67755	99.5685

Totals : 2.64364e4 611.09208

Signal 3: DAD1 C, Sig=254,4 Ref=360,100

Signal 4: DAD1 D, Sig=280,4 Ref=360,100

Signal 5: DAD1 E, Sig=310,4 Ref=360,100

===

*** End of Report ***

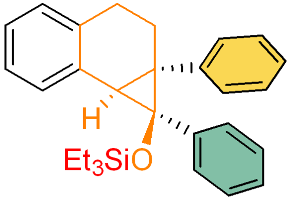

4

Data File C:\Users\P...Documents\ChemStation\1\Data\SUN\SUN 2024-07-13 22-12-06\1DA-0601.D
Sample Name: lsx-8-139tbafrac

===

Acq. Operator : SYSTEM Seq. Line : 6
Sample Operator : SYSTEM
Acq. Instrument : HPLC Location : P1-D-01
Injection Date : 13/7/2024 11:49:44 pm Inj : 1
Inj Volume : 2.000 µl
Different Inj Volume from Sample Entry! Actual Inj Volume : 5.000 µl
Acq. Method : C:\Users\Public\Documents\ChemStation\1\Data\SUN\SUN 2024-07-13 22-12-06\IC3-2-25.M
Last changed : 2/7/2024 6:32:09 pm by SYSTEM
Analysis Method : C:\Users\Public\Documents\ChemStation\1\Data\SUN\SUN 2024-07-13 22-12-06\IC3-2-25.M (Sequence Method)
Last changed : 22/7/2024 9:45:20 pm by SYSTEM
(modified after loading)
Additional Info : Peak(s) manually integrated

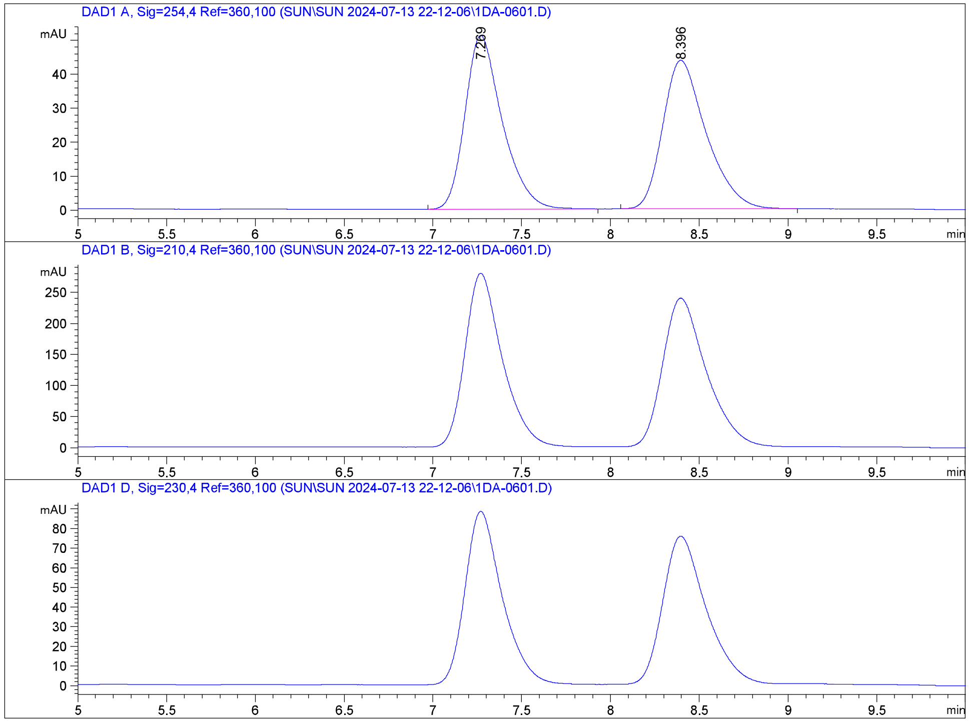

Data File C:\Users\P...Documents\ChemStation\1\Data\SUN\SUN 2024-07-13 22-12-06\1DA-0601.D
Sample Name: lsx-8-139tbafrac

===

Area Percent Report

===

Sorted By : Signal
Multiplier : 1.0000
Dilution : 1.0000
Use Multiplier & Dilution Factor with ISTDs

Signal 1: DAD1 A, Sig=254,4 Ref=360,100

Peak #	RetTime [min]	Type	Width [min]	Area [mAU*s]	Height [mAU]	Area %
1	7.269	BB	0.2164	745.83508	51.21305	50.1220
2	8.396	BB	0.2479	742.20300	43.76320	49.8780

Totals : 1488.03809 94.97625

Signal 2: DAD1 B, Sig=210,4 Ref=360,100

Signal 3: DAD1 D, Sig=230,4 Ref=360,100

===

*** End of Report ***

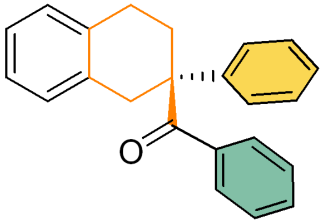

rac-5

Data File C:\Users\P...Documents\ChemStation\1\Data\SUN\SUN 2024-07-14 17-45-29\1DC-0201.D
Sample Name: lsx-8-139

===

Acq. Operator : SYSTEM Seq. Line : 2
Sample Operator : SYSTEM
Acq. Instrument : HPLC Location : P1-D-03
Injection Date : 14/7/2024 5:58:36 pm Inj : 1
Inj Volume : 2.000 µl
Different Inj Volume from Sample Entry! Actual Inj Volume : 5.000 µl
Acq. Method : C:\Users\Public\Documents\ChemStation\1\Data\SUN\SUN 2024-07-14 17-45-29 \IC3-2-25.M
Last changed : 2/7/2024 6:32:09 pm by SYSTEM
Analysis Method : C:\Users\Public\Documents\ChemStation\1\Data\SUN\SUN 2024-07-14 17-45-29 \IC3-2-25.M (Sequence Method)
Last changed : 22/7/2024 3:44:09 pm by SYSTEM
(modified after loading)
Additional Info : Peak(s) manually integrated

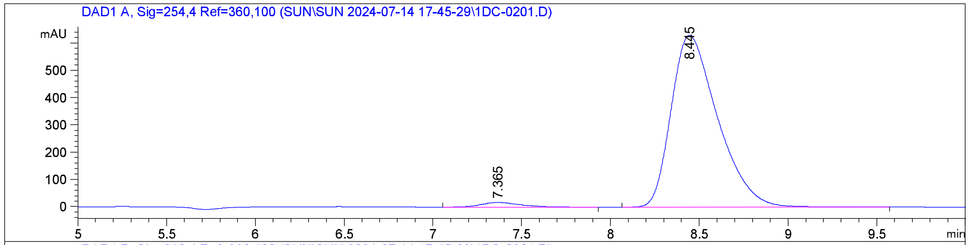

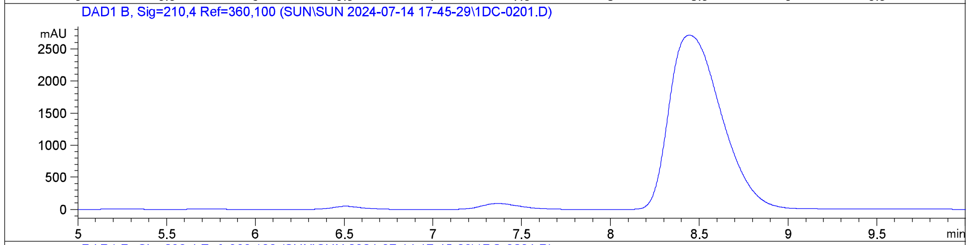

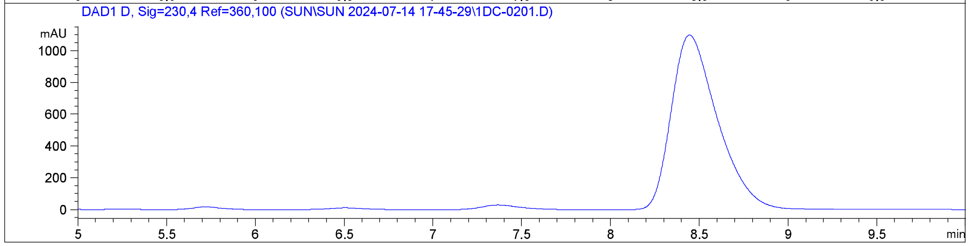

Data File C:\Users\P...Documents\ChemStation\1\Data\SUN\SUN 2024-07-14 17-45-29\1DC-0201.D
Sample Name: lsx-8-139

===

Area Percent Report

===

Sorted By : Signal
Multiplier : 1.0000
Dilution : 1.0000
Use Multiplier & Dilution Factor with ISTDs

Signal 1: DAD1 A, Sig=254,4 Ref=360,100

Peak #	RetTime [min]	Type	Width [min]	Area [mAU*s]	Height [mAU]	Area %
1	7.365	BB	0.2214	254.72859	16.99403	2.1785
2	8.445	BB	0.2745	1.14379e4	629.57275	97.8215

Totals : 1.16926e4 646.56679

Signal 2: DAD1 B, Sig=210,4 Ref=360,100

Signal 3: DAD1 D, Sig=230,4 Ref=360,100

===
*** End of Report ***

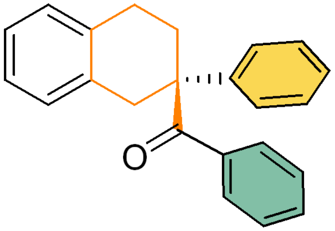

5
